# Psychosocial interventions for survivors of rape and sexual assault experienced during adulthood

**DOI:** 10.1002/14651858.CD013456.pub2

**Published:** 2023-10-05

**Authors:** Lorna O'Doherty, Maxine Whelan, Grace J Carter, Katherine Brown, Laura Tarzia, Kelsey Hegarty, Gene Feder, Sarah J Brown

**Affiliations:** Institute for Health and WellbeingCoventry UniversityCoventryUK; Department of General PracticeThe University of MelbourneMelbourneAustralia; Department of Psychology and Sports ScienceUniversity of HertfordshireHatfieldUK; The Royal Women's HospitalVictoriaAustralia; Centre for Academic Primary Care, Population Health Sciences, Bristol Medical SchoolUniversity of BristolBristolUK; Faculty of Arts, Business and Law, Law SchoolUSC: University of the Sunshine CoastSippy DownsAustralia; Faculty of Health and Applied SciencesUniversity of the West of EnglandBristolUK

**Keywords:** Adult, Female, Humans, Male, Behavior Therapy, Cognitive Behavioral Therapy, Cognitive Behavioral Therapy/methods, Psychosocial Intervention, Psychotherapy, Psychotherapy/methods, Rape

## Abstract

**Background:**

Exposure to rape, sexual assault and sexual abuse has lifelong impacts for mental health and well‐being. Prolonged Exposure (PE), Cognitive Processing Therapy (CPT) and Eye Movement Desensitisation and Reprocessing (EMDR) are among the most common interventions offered to survivors to alleviate post‐traumatic stress disorder (PTSD) and other psychological impacts. Beyond such trauma‐focused cognitive and behavioural approaches, there is a range of low‐intensity interventions along with new and emerging non‐exposure based approaches (trauma‐sensitive yoga, Reconsolidation of Traumatic Memories and Lifespan Integration). This review presents a timely assessment of international evidence on any type of psychosocial intervention offered to individuals who experienced rape, sexual assault or sexual abuse as adults.

**Objectives:**

To assess the effects of psychosocial interventions on mental health and well‐being for survivors of rape, sexual assault or sexual abuse experienced during adulthood.

**Search methods:**

In January 2022, we searched CENTRAL, MEDLINE, Embase, 12 other databases and three trials registers. We also checked reference lists of included studies, contacted authors and experts, and ran forward citation searches.

**Selection criteria:**

Any study that allocated individuals or clusters of individuals by a random or quasi‐random method to a psychosocial intervention that promoted recovery and healing following exposure to rape, sexual assault or sexual abuse in those aged 18 years and above compared with no or minimal intervention, usual care, wait‐list, pharmacological only or active comparison(s). We classified psychosocial interventions according to Cochrane Common Mental Disorders Group’s psychological therapies list.

**Data collection and analysis:**

We used the standard methodological procedures expected by Cochrane.

**Main results:**

We included 36 studies (1991 to 2021) with 3992 participants randomly assigned to 60 experimental groups (3014; 76%) and 23 inactive comparator conditions (978, 24%).

The experimental groups consisted of: 32 Cognitive Behavioural Therapy (CBT); 10 behavioural interventions; three integrative therapies; three humanist; five other psychologically oriented interventions; and seven other psychosocial interventions. Delivery involved 1 to 20 (median 11) sessions of traditional face‐to‐face (41) or other individual formats (four); groups (nine); or involved computer‐only interaction (six). Most studies were conducted in the USA (n = 26); two were from South Africa; two from the Democratic Republic of the Congo; with single studies from Australia, Canada, the Netherlands, Spain, Sweden and the UK. Five studies did not disclose a funding source, and all disclosed sources were public funding.

Participants were invited from a range of settings: from the community, through the media, from universities and in places where people might seek help for their mental health (e.g. war veterans), in the aftermath of sexual trauma (sexual assault centres and emergency departments) or for problems that accompany the experience of sexual violence (e.g. sexual health/primary care clinics). Participants randomised were 99% women (3965 participants) with just 27 men. Half were Black, African or African‐American (1889 participants); 40% White/Caucasian (1530 participants); and 10% represented a range of other ethnic backgrounds (396 participants). The weighted mean age was 35.9 years (standard deviation (SD) 9.6). Eighty‐two per cent had experienced rape or sexual assault in adulthood (3260/3992). Twenty‐two studies (61%) required fulfilling a measured PTSD diagnostic threshold for inclusion; however, 94% of participants (2239/2370) were reported as having clinically relevant PTSD symptoms at entry.

The comparison of psychosocial interventions with inactive controls detected that there may be a beneficial effect at post‐treatment favouring psychosocial interventions in reducing PTSD (standardised mean difference (SMD) ‐0.83, 95% confidence interval (CI) ‐1.22 to ‐0.44; 16 studies, 1130 participants; low‐certainty evidence; large effect size based on Cohen’s D); and depression (SMD ‐0.82, 95% CI ‐1.17 to ‐0.48; 12 studies, 901 participants; low‐certainty evidence; large effect size). Psychosocial interventions, however, may not increase the risk of dropout from treatment compared to controls, with a risk ratio of 0.85 (95% CI 0.51 to 1.44; 5 studies, 242 participants; low‐certainty evidence). Seven of the 23 studies (with 801 participants) comparing a psychosocial intervention to an inactive control reported on adverse events, with 21 events indicated. Psychosocial interventions may not increase the risk of adverse events compared to controls, with a risk ratio of 1.92 (95% CI 0.30 to 12.41; 6 studies; 622 participants; very low‐certainty evidence).

We conducted an assessment of risk of bias using the RoB 2 tool on a total of 49 reported results. A high risk of bias affected 43% of PTSD results; 59% for depression symptoms; 40% for treatment dropout; and one‐third for adverse events. The greatest sources of bias were problems with randomisation and missing outcome data. Heterogeneity was also high, ranging from I^2^ = 30% (adverse events) to I^2^ = 87% (PTSD).

**Authors' conclusions:**

Our review suggests that survivors of rape, sexual violence and sexual abuse during adulthood may experience a large reduction in post‐treatment PTSD symptoms and depressive symptoms after experiencing a psychosocial intervention, relative to comparison groups. Psychosocial interventions do not seem to increase dropout from treatment or adverse events/effects compared to controls. However, the number of dropouts and study attrition were generally high, potentially missing harms of exposure to interventions and/or research participation. Also, the differential effects of specific intervention *types* needs further investigation.

We conclude that a range of behavioural and CBT‐based interventions may improve the mental health of survivors of rape, sexual assault and sexual abuse in the short term. Therefore, the needs and preferences of individuals must be considered in selecting suitable approaches to therapy and support. The primary outcome in this review focused on the post‐treatment period and the question about whether benefits are sustained over time persists. However, attaining such evidence from studies that lack an active comparison may be impractical and even unethical. Thus, we suggest that studies undertake head‐to‐head comparisons of different intervention types; in particular, of novel, emerging therapies, with one‐year plus follow‐up periods. Additionally, researchers should focus on the therapeutic benefits and costs for subpopulations such as male survivors and those living with complex PTSD.

## Plain language summary

**How helpful to recovery and healing are support and psychological interventions after exposure to sexual violence and abuse?**

**Key messages**

• We found evidence that psychological or social (collectively known as ‘psychosocial’) interventions may reduce symptoms of post‐traumatic stress disorder (PTSD) and depression in survivors of rape, sexual assault and abuse experienced during adulthood.

• Our review suggests that interventions did not worsen symptoms or lead to unwanted effects. However, as large numbers of participants dropped out of treatments or did not complete studies’ assessments, these findings are unclear. More recent studies were better at reporting information about participant safety, and reasons why survivors did not complete treatments or health and wellbeing assessments after the interventions.

• As the studies brought diverse groups of participants together, future research is needed to improve understanding about which interventions are most suited to particular groups of survivors, including those with long‐term or complex trauma, as well as men and gender minorities. 'Emerging' interventions, which have potential to expand treatment choices for survivors, also warrant more evaluation.

**What is 'sexual violence and abuse'?**

‘Sexual violence and abuse’ means any sexual activity or act that happened without consent. It includes rape, sexual assault, sexual abuse and sexual harassment. It causes emotional and physical health problems that can be long‐lasting. The effects are often made worse by fear, shame, feelings of self‐blame and the negative responses of others.

**How is sexual violence and abuse treated?**

Survivors have a range of physical, sexual health and forensic care needs in the aftermath of rape, sexual assault or abuse. Psychosocial interventions may be offered in response to these needs at different stages in survivors’ recovery journeys. Some interventions aim to assist survivors by carefully re‐exposing them to aspects of the original trauma to ‘process’ what happened (e.g. Trauma‐focused Cognitive Behavioural Therapy (CBT)). Other treatments focus less on the traumatic memories, instead helping survivors cope with life after abuse (e.g. different forms of counselling; education about mental health; and support for a range of needs).

**What did we want to find out?**

We wanted to know whether psychosocial interventions help to relieve the mental health impact experienced by survivors as a result of rape, sexual assault or sexual abuse in adulthood. We also wanted to know if some types of interventions were more helpful than others.

**What did we do?**

We searched for studies comparing the effects of psychosocial interventions for individuals who had been subjected to rape, sexual assault or sexual abuse from the age of 18 years, with a control group (a group of participants who did not receive the intervention but instead were given their usual care, were placed on a waiting‐list for treatment, or received very minimal assistance, such as leaflets). We looked for differences between groups on trauma and depression symptoms after receiving the intervention; dropout from interventions (non‐completion); and any unwanted effects related to the intervention or research.

**About the studies and their participants**

We found 36 studies that placed consenting adult participants by chance into an intervention or a control group. Participants were invited from a range of settings: community; universities; places where people seek help for their mental health, sexual trauma (e.g. specialist sexual assault centres and emergency departments) or for problems that occur alongside the experience of sexual violence (e.g. primary care clinics); and via media requests. The studies included 3992 survivors; only 27 were men. Sixty per cent of participants were Black or from a minority ethnic or cultural background. The average age was 36 years, and nearly all had symptoms of PTSD.

Most studies were done in the USA (26); there were two from South Africa; two from the Democratic Republic of the Congo; and single studies from Australia, Canada, the Netherlands, Spain, Sweden and the UK. Five studies did not disclose a funding source; those that did reported public funding.

Over half the interventions were CBT‐based. Support was mostly delivered one‐to‐one by trained mental health professionals and varied between 1 and 20 sessions.

**What did we find?**

Survivors who participate in a psychosocial intervention may experience a large reduction in PTSD and depression symptoms soon after the intervention is completed. Non‐completion was not more common among survivors who experienced interventions compared to control groups, but this was based on a small number of studies. Psychosocial interventions may not increase the risk of unwanted effects. Only seven studies reported 21 unwanted effects, suggesting most researchers may not have actively monitored the negative impacts of interventions or participation in the studies.

**What are the limitations of the evidence?**

We have little confidence in the results because of concerns about the level of variation across the studies (e.g. types of survivor experiences, wide range of interventions and study sizes). It is possible that the allocation of survivors to one group or another may not have been entirely random. Furthermore, survivors who did not complete interventions or study assessments may have differed in important ways from survivors who did (e.g. had better/worse health problems).

**How up‐to‐date is the evidence?**

The evidence is up‐to‐date to January 2022.

## Summary of findings

**Summary of findings 1:** Summary of findings table ‐ Psychosocial interventions compared to inactive control for survivors of sexual violence and abuse

**Psychosocial interventions compared to inactive control for survivors of sexual violence and abuse**						
**Patient or population:** survivors of sexual violence and abuse **Setting:** mental health clinics; veterans affairs medical centres; sexual assault and abuse services in acute, primary care and community; and academic/experimental settings **Intervention:** psychosocial interventions **Comparison:** inactive control						
**Outcomes**	Anticipated absolute effects^*^ (95% CI)		**Relative effect (95% CI)**	**№ of participants (studies)**	**Certainty of the evidence (GRADE)**	**Comments**
	**Risk with inactive control**	**Risk with psychosocial interventions**				
PTSD symptoms, post‐treatment (self‐reported or clinician‐rated)	‐	SMD **0.83 lower** (1.22 lower to 0.44 lower)	‐	1130 (16 RCTs)	⊕⊕⊝⊝ Low^a,^^b^	Lower score means fewer PTSD symptoms. An SMD of ‐0.83 is a large effect (Cohen’s D). Subgroup analyses indicate there may be evidence of a group difference for CBT (SMD ‐0.77) and Behavioural Therapy (SMD ‐1.85), but no evidence of a difference for low‐intensity interventions (P = 0.09).
Depressive symptoms, post‐treatment (self‐reported or clinician‐rated)	‐	SMD **0.82 lower** (1.17 lower to 0.48 lower)	‐	901 (12 RCTs)	⊕⊕⊝⊝ Low^a,^^c,^^d^	Lower score means fewer depressive symptoms. An SMD of ‐0.82 is a large effect (Cohen's D). Subgroup analyses indicate there may be evidence of a group difference for CBT (SMD ‐0.73) and Behavioural Therapy (SMD ‐1.51), but no evidence of a difference for low‐intensity interventions (P = 0.39).
Dropout from treatment (a count of participants not meeting study‐defined completion threshold)	336 per 1000	**286 per 1000** (171 to 484)	**RR 0.85** (0.51 to 1.44)	242 (5 RCTs)	⊕⊕⊝⊝ Low^e,^^f^	
Adverse events (a count of reported harms or adverse events/experiences over life of study/follow‐up)	7 per 1000	**13 per 1000** (2 to 82)	**RR 1.92** (0.30 to 12.41)	622 (6 RCTs)	⊕⊝⊝⊝ Very low^g,^^h,^^i^	There were 21 adverse events reported in just 7 studies, suggesting many studies may not have actively monitored negative impacts of the treatments or being in the research.
*The risk in the intervention group (and its 95% confidence interval) is based on the assumed risk in the comparison group and the **relative effect** of the intervention (and its 95% CI). **CI:** confidence interval; **RR:** risk ratio; **SMD:** standardised mean difference						
**GRADE Working Group grades of evidence** **High certainty:** we are very confident that the true effect lies close to that of the estimate of the effect. **Moderate certainty:** we are moderately confident in the effect estimate: the true effect is likely to be close to the estimate of the effect, but there is a possibility that it is substantially different. **Low certainty:** our confidence in the effect estimate is limited: the true effect may be substantially different from the estimate of the effect. **Very low certainty:** we have very little confidence in the effect estimate: the true effect is likely to be substantially different from the estimate of effect.						
See interactive version of this table: https://gdt.gradepro.org/presentations/#/isof/isof_question_revman_web_429039861582912181.						

^a^ Sensitivity analyses removing studies at high risk of bias reduced the effect size to moderate based on Cohen's D. ^b^ Downgraded 1 level due to heterogeneity I2 = 87%, P < 0.001. ^c^ Downgraded 1 level due to 50% or more of the results receiving an overall high risk of bias judgement. ^d^ Downgraded 1 level due to heterogeneity I2 = 78%, P < 0.001. ^e^ Only includes the 5 studies that reported dropout from a psychosocial intervention vs minimal intervention; this analysis may not directly address the question about treatment completion as this requires comparing 2 active interventions. ^f^ The confidence interval includes appreciable benefit or harm. ^g^ Only 6 of 23 studies in the main comparison reported on adverse events by group, raising concerns about selective reporting bias. ^h^ Adverse events were not reported using consistent methods. ^i^ Imprecision due to too few events.

## Background

### Description of the condition

Rape, sexual assault and sexual abuse are serious public health and human rights problems (WHO 2013a). For the purpose of the review, we will use the overarching term 'sexual assault', which refers to any act of physical, psychological and emotional violation in the form of a sexual act, inflicted on someone without their consent (see review protocol for overview of definitions; Brown 2019). Sexual assault disproportionately affects the lives of women (Walby 2016). Research into men's experiences of sexual assault has been scant by comparison, and the prevalence of sexual assault perpetrated against men is largely unknown. In England and Wales, it is estimated that 4% of adults aged 16 to 74 years (1.6 million people) have experienced sexual assault by rape or penetration (including attempts) since the age of 16 years (7% for women and 0.5% for men) (ONS 2021). Social and legal marginalisation, exacerbated by gender‐defined services, stigma and discrimination, all mean that sexual assault experienced by gender and sexual minorities is hidden and poorly understood (e.g. see Wirtz 2018). There are extensive immediate and long‐term physical and mental health consequences for survivors (Jina 2013). The consequences for adult and child victims include injuries, substance misuse, eating disorders, post‐traumatic stress disorder (PTSD), anxiety, depression, self‐harm and suicidality (Oram 2017; WHO 2013a). Sexual and reproductive health implications include condom non‐use, unwanted pregnancy (Decker 2014), sexually transmitted infections (WHO 2013a), urinary tract infections, painful sex, chronic pelvic pain and vaginal bleeding (Campbell 2002), fistula (Dossa 2014) and increased risk of sexual dysfunction such as low sexual desire and difficulty with physiological and psychological sexual arousal, and low sexual satisfaction (Pulverman 2018; Pulverman 2021). For male victims, physical health consequences include genital and rectal injuries and erectile dysfunction (Tewkesbury 2007; Weare 2021). The negative effects of rape and sexual assault ripple across generations, having social and economic costs in addition to impacts on physical and mental health by affecting individuals’ capacities to work and to participate in family and community life.

The mental health burden is substantial and similar across male and female victims (Coxell 2010; Tewkesbury 2007; Walker 2005; WHO 2013a). Sexual assault was ranked among the top three most traumatic life events in the US National Epidemiologic study (Pietrzak 2011). Those with a psychiatric diagnosis of PTSD were four times more likely to report exposure to sexual assault than those that did not have a diagnosis, and 13% of women with PTSD had lifetime experience of sexual assault. PTSD is a psychiatric disorder that can follow exposure to psychological trauma and is associated with intrusive memories, nightmares, avoidance, and problems with sleep and concentration (Lerman 2019). Guina and colleagues reported no difference in PTSD symptoms and severity among men and women who had experienced sexual trauma (Guina 2016). Indirect pathways to poor long‐term health outcomes are also of concern; for example, taking lifetime PTSD as a proxy, PTSD is associated with increased risk of hypertension, cardiovascular disease (Jakubowski 2018) and gastrointestinal problems (Pietrzak 2011). Thus, the immense physical and psychological impacts of sexual violence exposure can lead to long‐term disability.

Considering what is known about the prevalence of sexual assault, the risk for developing PTSD and poor mental health after exposure to sexual violence and abuse, and the risk for re‐victimisation and further traumatisation (Finkelhor 2007; Möller 2017), identifying accessible and cost‐effective treatments for this population is of great importance.

A large body of work exists to establish the most effective psychological treatments for PTSD in mixed trauma populations such as among those affected by disasters; migration/refugee trauma; active duty and ex‐serving military service; physical abuse; medical trauma; traumatic grief; intimate partner violence; child sexual abuse; sexual assault and rape; and military sexual trauma. This work has led to trauma‐focused therapies being recommended as frontline interventions for PTSD (e.g. Centre for Posttraumatic Mental Health 2013; NICE 2018; VA/DoD 2017). Trauma‐focused therapies include behaviour‐based approaches such as Eye Movement Desensitisation Reprocessing (EMDR), and Cognitive Behavioural Therapy with a trauma focus (TF‐CBT), of which Cognitive Processing Therapy (CPT) and Prolonged Exposure (PE) have received the most attention.

Our work aims to extend this body of research by synthesising evidence focused specifically on survivors of rape, sexual assault and abuse during adulthood. Given the wide range of contexts in which survivors of sexual assault are located, and the variable needs of individuals depending on their stage of recovery and resources available, we aimed for broad scope around the nature of the interventions we included. We refer to 'psychosocial' interventions as encompassing support and advocacy interventions for survivors as well as psychological or psychotherapeutic treatments.

### Description of the intervention

Psychosocial interventions are those that involve “interpersonal or informational activities, techniques, or strategies that target biological, behavioural, cognitive, emotional, interpersonal, social, or environmental factors with the aim of improving health functioning and well‐being” (IOM 2015 p.5). For the purposes of this review, we organised psychosocial interventions according to the list of psychological therapies of the former Cochrane Depression, Anxiety and Neurosis (CCDAN) and Cochrane Common Mental Disorders (CCMD) Groups. These include the following types of interventions:

a) Cognitive Behavioural Therapy (CBT) including trauma‐focused CBT (TF‐CBT) and other CBT‐based techniques are promoted for the treatment of PTSD in best practice guidelines (Centre for Posttraumatic Mental Health 2013; Foa 2009; IOM 2008; NICE 2018; VA/DoD 2017). Cognitive‐behavioural processes can be sub‐classified into three major classes (Dobson 2009): 1) cognitive re‐structuring, which focuses on internal underlying beliefs and thoughts with the aim of challenging maladaptive thought patterns; 2) coping skills therapy, which targets the identification and alteration of cognitions and behaviours that may increase the impact of negative external events; and 3) problem‐solving therapies, which combine cognitive re‐structuring and coping skills therapy to change internal thought patterns and optimise responses to external negative events. Each of these three classes have a slightly different target for change, demonstrating the wide range of psychological interventions based upon cognitive‐behavioural principles (Dobson 2009). Cognitive Processing Therapy (CPT; Resnick 1997) and Prolonged Exposure Therapy (PET; Foa 1986) are the most common cognitive‐behavioural approaches evaluated in studies of interventions for people affected by PTSD and for those who have been subjected to sexual assault. Cognitive Processing Therapy and Prolonged Exposure are considered trauma‐focused CBT and they are also 'exposure'‐based therapies. This means they use the concept of emotional processing theory (Foa 1986), that is, they use interventions for activation of fear structure, habituation and disconfirmation of erroneous cognitions and beliefs to treat PTSD (Foa 2016). Other CBTs used with sexual assault survivors such as Stress Inoculation Therapy (SIT; Meichenbaum 1977) do not encourage imaginal exposure.

b) Behaviour therapies include relaxation techniques and Eye Movement Desensitisation and Reprocessing (EMDR; Shapiro 1995). NICE guidance recommends EMDR for adults with a diagnosis of PTSD (or clinically important symptoms of PTSD) who have presented after a (non‐combat related) trauma if the person has a preference for EMDR (NICE 2018). As with TF‐CBT, EMDR sits within a trauma‐response theoretical model (Goodman 1993; Herman 1992).

c) Third‐Wave CBT, such as Acceptance and Commitment Therapy and mindfulness, prioritises the holistic promotion of psychological and behavioural processes associated with well‐being over the reduction of psychological and emotional symptoms. It focuses on context, processes and functions of how a person relates to internal experiences over the content of the thoughts themselves.

d) Integrative Therapies include such approaches as interpersonal therapy and blend elements of different traditions or approaches.

e) Humanistic Therapies include Gestalt and experiential approaches, as well as supportive and non‐directive therapy following the work of Rogers among others. Person‐Centred Therapy focuses on providing support, and discussing and understanding in the present problems that are generated by the client.

f) Other psychologically orientated interventions such as art therapy; meditation; and hypnotherapy. A good example is Present‐Centred Therapy, initially developed as a control group for other therapies and recommended as a second‐line treatment in its own right (Hamblen 2019). The theoretical basis for Present‐Centred Therapy arises from the large literature on 'common factors' used in psychotherapy like the supportive therapeutic relationship, a rationale that explains the person's symptoms and the steps for relieving them, the experience of talking about problems in a safe environment, and creating positive expectations and hope.

g) Support and services delivered by mentors, support workers, advisors or advocates (for example, independent sexual assault advisors (ISVAs), in the UK), and support groups.

To emphasise the evidence of benefits (and harms) of psychosocial interventions across diverse adult survivor communities, we set out to include studies of sexual assault‐only samples, as well as studies where sexual assault survivors were a subset of a wider trauma sample. Despite the focus on sexual assault alone, we anticipated a high degree of heterogeneity across study populations due to the different settings in which people get help and due to the widespread contexts of abuse. Studies evaluating treatments for exposure to sexual assault involve university students (Anderson 2010); veterans and active service personnel subjected to military sexual trauma (Acierno 2021; Katz 2014; Suris 2008); acute settings such as emergency departments (Walsh 2017); survivors seeking counselling and specialist support for sexual assault (Nixon 2016; Rajan 2020) and other forms of support in the community (Bass 2013; Bass 2016); and clinical samples and mental health outpatients (Falsetti 2008; Foa 2006). Such features are important; for example, the military environment may serve to intensify trauma and impact a person’s capacity to cope following a military sexual trauma, if they have limited options for escaping the perpetrator in such a closed environment (Surís 2013). In this sense, Katz and colleagues liken it to child sexual abuse, feeling betrayed by those who are supposed to be protective, and not being able to report or get help without incurring devastating consequences (Katz 2014). The experience of intimate partner sexual violence may also produce these adverse effects. Aside from the nature and context of the assault, studies may also focus on how effectively interventions serve survivors with certain characteristics such as age (Bowland 2012); cultural and ethnicity heritage (Feske 2008); and gender (Echeburua 1996; Gray 2020).

Thus, in order to respond to the needs and circumstances of these varied samples of survivors, studies test not only different intervention types but also different modalities for delivering them. These include group (Bass 2013; Bass 2016) and individual (Foa 1991; Foa 1999; Foa 2005; Foa 2006) formats and delivery via computer (Bomyea 2015), online, by telephone (Anderson 2010), video (Miller 2015), using telemedicine or in person (Acierno 2021). A large proportion of the work has emerged from expert treatment centres and/or with clinicians highly proficient in and with strong allegiance to trauma‐focused CBT approaches used for sexual assault survivors (Nixon 2016) and interventions might involve single sessions (Rajan 2020) or a small number of sessions (Gray 2020) or multiple sessions (Belleville 2018; Falsetti 2008). It is important, therefore, to compare the effects of interventions of different intensity and duration to identify cost‐effective ways of supporting survivors. Thus, the review will extend beyond this to include non‐specialist settings and will examine these many different aspects of interventions and populations.

See Appendix 1.

### How the intervention might work

See Appendix 1.

### Why it is important to do this review

Clinical and policy guidelines inform responses to rape and sexual assault (e.g. NICE 2018; WHO 2013b), but gaps remain in our knowledge of the most effective ways of intervening to improve health outcomes and prevent further victimisation. While there is moderate evidence on the consequences of sexual trauma (Description of the condition), it is less clear what happens to people’s health and well‐being over time, including in response to different interventions. Whilst TF‐CBT and EMDR are recommended for PTSD, none has been fully effective in its treatment (Kitchiner 2019), with most studies reporting that between 60% and 72% of participants retain diagnosis (Steenkamp 2015). Dropout is also a concern in studies of interventions that involve in vivo or imaginal exposure, or both. In response, there have been calls for new and more effective approaches to the treatment of PTSD (Gray 2020; Kitchiner 2019; Steenkamp 2015).

There is good evidence for the effects of psychological treatments in reducing mental health issues in children who have experienced sexual trauma (Gillies 2016), with CBT for sexually abused children with symptoms of post‐traumatic stress showing the best evidence for reduction in mental health conditions (MacDonald 2012; MacMillan 2009). However, these conclusions cannot be extrapolated to adults who have experienced sexual trauma, and there has been no recent systematic review or meta‐analysis examining the effects of intervention on this population.

Relative to intimate partner violence (IPV), sexual violence has received less attention in the research literature, and several reviews focus on psychological interventions for IPV (Arroyo 2017; Hameed 2020; Trabold 2018). While there is certainly overlap in the populations of interest, in that many sexual assaults and rapes occur within IPV, rape and sexual assault is not exclusive to IPV and many who experience sexual trauma as adults outside a domestic abuse context require support or interventions. Those reviews that have looked at rape and sexual assault have tended to focus on women (Parcesepe 2015) and children (Gillies 2016; MacDonald 2012), indicating that the experiences of men and transgender survivors are less represented in the literature. Similarly, the representation of sexual minorities and ethnic minorities is typically minimal in intervention studies (IOM 2008), with studies rarely sufficiently powered to detect benefits and costs for specific user groups or subgroups of survivors.

Other reviews have focused on psychological therapies for PTSD in any population (Bisson 2013); in specific populations such as military personnel with PTSD (Kitchiner 2019) or those with comorbid substance misuse problems (Roberts 2015; Roberts 2016); or examined combined pharmacotherapy and psychological therapies for PTSD (Hetrick 2010). In these reviews, sexual assault and rape survivors are a subset of the population. While these reviews are helpful in understanding appropriate therapies to combat PTSD specifically, not all sexual assault or rape victims experience PTSD, and the impacts of sexual trauma are broader than PTSD. Campbell and colleagues published a review in 2009 (Campbell 2009) and Regehr and colleagues a systematic review in 2013 (Regehr 2013) on interventions to reduce distress in adult victims of sexual assault and rape. These reviews are relevant; however, they are now a decade out of date, and there have been many developments in terms of novel interventions since these publications. Contemporary approaches have been tested in several recent studies, including Reconsolidation of Traumatic Memories (Gray 2020), Lifespan Integration (Rajan 2020); trauma‐sensitive yoga (Kelly 2021); and neurofeedback and biofeedback (Bell 2019).

We believe it is important to examine the interventions that go beyond psychotherapeutic approaches. Survivors may not be able to access psychotherapy (e.g. due to long waiting lists or lack of available services) or they may not be at the appropriate stage in their recovery to discuss the traumatic experience. Among those involved in criminal justice proceedings, there may be concerns about material generated as a result of therapy being obtained by police on the basis that it represents a reasonable line of enquiry (CPS 2021). For these reasons, psychosocial interventions that avoid discussion of the trauma can be a vital source of support to rape and sexual assault victims. Although many psychosocial interventions have demonstrated effectiveness, the findings have not been synthesised well, and it can be difficult to know what treatments are effective (IOM 2015).

The current review also examines the broader range of impacts of sexual trauma for all victims who experience rape and sexual assault as adults. Hence, this review is feasible and timely and addresses an important gap in the current literature.

## Objectives

To assess the effects of psychosocial interventions on mental health and well‐being for survivors of rape and sexual assault experienced during adulthood.

## Methods

### Criteria for considering studies for this review

#### Types of studies

Any study that allocated individuals or clusters of individuals by a random or quasi‐random method (whereby the method of allocation was not truly random such as alternate allocation, allocation by birth date, day shift etc.) to a psychosocial intervention for adult victims of rape or sexual assault compared with no intervention, usual care, wait‐list, or minimal or active comparison (see 'Comparator intervention' under Types of interventions).

Studies were eligible for inclusion in the review if they used random assignment to treatment and comparison groups or employed one of the following designs: quasi‐randomised controlled trial (RCT) (non‐randomised experimental design trials); cluster‐randomised trials (instead of individuals, groups will be randomised) or cross‐over trial (longitudinal studies where the participant receives a sequence of different treatments).

#### Types of participants

Adults aged 18 years and older, of any gender, who had experienced rape or sexual assault as an adult (i.e. aged 18 years and older), irrespective of a mental health diagnosis. Types of sexual assault included rape, attempted rape, forced oral sex, anal sex, penetration with objects, touching of intimate parts and any sexual contact where consent was not given, as well as forcing or manipulating someone to witness sexual acts. We included studies of participants who screened positive for exposure to sexual violence, even if they did not report what those behaviours were. We included studies involving subsets of eligible participants provided that the subset included at least 50% of those randomised and could be analysed separately. We included studies of participants recruited in any setting (e.g. community, forensic, criminal justice and health).

We excluded samples made up entirely of individuals (adult or child) who were victims of rape, sexual assault or sexual abuse during their childhood (aged 17 years and under), as well as samples of children (i.e. those younger than 18 years of age).

#### Types of interventions

##### Experimental intervention

The experimental intervention consisted of any type of psychosocial and psychological intervention that targeted recovery from sexual assault or rape, including the following.

1. Formal CBT, TF‐CBT and CBT‐based techniques
2. Integrative therapies (e.g. interpersonal therapy)
3. Behaviour therapies (e.g. EMDR and relaxation techniques)
4. Third‐wave CBT (e.g. Acceptance and Commitment Therapy, mindfulness)
5. Humanistic therapies (e.g. supportive and non‐directive therapy)
6. Other psychologically orientated interventions (e.g. art therapy, meditation, trauma‐informed body‐based practices (such as embodied relational therapy, yoga and Tai Chi), narrative therapy)
7. Other psychosocial interventions, including support services delivered by mentors, support workers, advisors or advocates (such as independent sexual assault advisors (ISVAs) in the UK), support groups and coping interventions.

We included interventions of any duration or frequency of treatment so long as the treatment met the criteria stated above.

For all interventions, mode of intervention delivery included one or more of the following: face‐to‐face; telephone; or computer‐based delivery. We included both individual and group delivery of the intervention.

##### Comparator intervention

Comparator interventions consisted of inactive controls, such as usual care, no treatment, delayed provision of psychological interventions (or wait‐list conditions), or pharmacological treatment only, and minimal interventions such as information provision. However, we did not exclude studies on the grounds that an active control group had been used (e.g. where an intervention from one category (CBT) was compared to an intervention from another category (psychosocial intervention), or different intensities or dosages of an intervention were compared). We recognised that there will be instances where researchers employ an active comparison condition for pragmatic or ethical reasons (e.g. the importance of offering some care or treatment to a survivor and that research studies may replicate this when designing or delivering an evaluation). In our analyses, we strived to pool studies that conducted similar types of comparisons (i.e. active versus inactive or active versus active).

#### Types of outcome measures

We did not select studies based on the nature of the outcomes assessed. The review was designed to measure the effects of psychological therapies and psychosocial interventions for survivors of rape and sexual assault experienced during adulthood, based on a wide range of indicators of a person's health and well‐being, particularly mental health and well‐being. We were also mindful about evaluating harm and adverse consequences from therapies and other interventions.

Where studies used multiple measures of the same outcome within the same study (e.g. PTSD symptoms collected using an interview‐based assessment *and* a self‐report measure), we extracted all data. However, we prioritised the interview‐generated data in meta‐analyses on the basis that such assessments of symptoms are likely to be more reliable.

We extracted data arising at four time points (post‐treatment, three months, six months and 12 months). The primary time point for treatment efficacy was post‐treatment (Differences between protocol and review), encompassing an assessment period extending from immediately after the intervention up to one month; we frequently refer to post‐treatment as 'the days and weeks after intervention'.

For the purpose of interpreting the time between intervention and outcome assessments, we classified time points up to six months as short term; up to 12 months as medium‐term; and long‐term as 12 months or longer.

##### Primary outcomes

1. Treatment efficacy, PTSD symptoms: response to treatment, determined by differences in scores for PTSD symptoms, assessed by independent observer or self‐report. Validated observer‐rated instruments include the Clinician‐Administered PTSD Symptom Scale (Kulka 1988), Clinician‐Administered PTSD Scale (CAPS; Blake 1990; Blake 1995), and the PTSD Symptom Scale ‐ Interview (PSS‐I; Foa 1993). Validated self‐report measures include the PTSD Symptom Scale ‐ Self‐Report (PSS‐SR; Foa 1993), Impact of Event Scale (IES; Horowitz 1979), Impact of Event Scale ‐ Revised (IES‐R; Weiss 1997), and PCL‐5 (Bovin 2016), which is the self‐reported PTSD Checklist for the *Fifth Edition of the Diagnostic and Statistical Manual of Mental Disorders* (DSM–5; APA 2013).
2. Treatment efficacy, depressive symptoms: response to treatment, determined by differences in scores for depressive symptoms, assessed by independent observer or self‐report measures, including the Hospital Anxiety and Depression Scale (HADS; Zigmond 1983), Beck Depression Inventory (BDI; Beck 1961), Center for Epidemiologic Studies Depression Scale (CES‐D; Radloff 1977), Patient Health Questionnaire (PHQ; Spitzer 1999), and Hamilton Depression Rating Scale (HAM‐D; Hamilton 1960).
3. Treatment acceptability: the number of participants who dropped out of the intervention (as distinct from attrition), including in studies of two intervention types and other assessments of acceptability (e.g. measures of patient/client satisfaction).
4. Adverse effects, such as counts of mortality, completed suicides and attempted suicides, or worsening of symptoms (specifically, group differences on PTSD, depression, self‐harm and suicidality ‐ see below for tools), including those summarised in narrative form, or using a tool such as the Negative Effects Questionnaire (Rozental 2018). We recorded whether or not studies made reference to this outcome.

##### Secondary outcomes

1. Anxiety symptoms, assessed with self‐report scales such as the Beck Anxiety Inventory (BAI; Beck 1988), State‐Trait Anxiety Inventory (STAI; Spielberger 1970), or Generalised Anxiety Disorder ‐ Seven‐item Scale (GAD‐7; Kertz 2013; Spitzer 2006).
2. Dissociation symptoms, measured using instruments such as the Dissociative Experiences Scale (DES; Bernstein 1986), or the Dissociative Experiences Scale‐II (DES‐II; Bernstein 1986; Carlson 1993).
3. Global mental health functioning/distress, which is frequently measured by either the Global Severity Index (GSI), Positive Symptom Distress Index (PSDI) and Positive Symptom Total (PST) of the SCL‐90‐R (Derogatis 1983), or by the Behavior And Symptom Identification Scale (BASIS‐32; Eisen 1999).
4. Feelings of guilt or self‐blame (or both; hereon described as trauma‐related beliefs) experienced by survivors, measured by self‐report tools such as the Trauma‐Related Guilt Inventory (TRGI; Kubany 1996), Rape Attribution Questionnaire (RAQ; Frazier 2003), South African Stigma Scale (Singh 2011), Social Support Appraisal (SSA) scale (Vaux 1986), Rape Aftermath Symptom Test (RAST; Kilpatrick 1988), or Inventory of Interpersonal Problems (IPP; Horowitz 1988).
5. Substance use, measured by a number of established scales, including the Michigan Alcoholism Screening Test (MAST; Selzer 1971), Drug Abuse Screening Test (DAST; Skinner 1982), Addiction Severity Index (ASI; McLellan 1980: McLellan 1992), Alcohol Use Inventory (AUI; Chang 2001), Drug Use Disorders Identification Test (DUDIT; Berman 2005), or the Alcohol Use Disorders Identification Test (AUDIT; Pradhan 2012).
6. Quality of life, which is commonly measured by self‐report measures such as the WHO Quality of Life scale ‐ Abbreviated Version (WHOQOL‐BREF; Skevington 2004) and EuroQol‐5 Dimensions (EQ‐5D; Brooks 1996).
7. Self‐harming or suicidality often measured by the Deliberate Self‐Harm Inventory (DSHI; Gratz 2001), Self‐Harm Behaviour Questionnaire (SHBQ; Guttierez 2001), or the Self‐Injury Questionnaire (SIQ; Santa Mina 2006).
8. Sexual violence assessment, measured by instruments such as the Sexual Experiences Survey (SES; Koss 1987b) and the Abuse Assessment Screen (AAS) (Basile 2007; NSVRC 2011). These tools differ in terms of their method of delivery; their appropriateness for screening for females, males, or both; the setting in which screening is to occur; the total number of questions they contain; and the number of questions that are specific to sexual violence (Basile 2007; NSVRC 2011).

### Search methods for identification of studies

We identified RCTs of psychological interventions for survivors of rape and sexual assault experienced during adulthood from key bibliographic databases listed in Electronic searches. We ran the first searches in July 2019, and updated them in March 2021 and again in January 2022.

#### Electronic searches

We searched the databases and trials registers listed below for published and unpublished studies. We adapted the MEDLINE strategy in Appendix 2 for the other sources using appropriate indexing terms and syntax. We did not apply any limitations on publication date, place or language of any research; we did not exclude any potentially relevant studies and we included research from different backgrounds and disciplines. The Information Specialist for Cochrane Developmental Psychosocial and Learning Problems searched all of the databases listed below, with the exception of the Common Mental Disorders Controlled Trials Register, which was searched by the Information Specialist for Cochrane Common Mental Disorders.

1. Cochrane Central Register of Controlled Trials (CENTRAL 2021, Issue 12) in the Cochrane Library. Searched 10 January 2022.
2. Cochrane Common Mental Disorders Controlled Trials Register (CCMDCTR; current to June 2016). Searched 2 July 2019. No new content added after this date. See Appendix 3 for one of the core strategies (MEDLINE) used to populate CCMDCTR.
3. MEDLINE Ovid (1946 to December Week 5 2021).
4. MEDLINE In‐Process & Non‐Indexed Citations Ovid (7 January 2022).
5. MEDLINE Epub Ahead of Print Ovid (7 January 2022).
6. Embase Ovid (1974 to 7 January 2022).
7. CINAHL Plus EBSCOhost (Cumulative Index to Nursing and Allied Health Literature; 1937 to 11 January 2022).
8. PsycINFO Ovid (1806 to January Week 1 2022).
9. ERIC EBSCOhost (Education Resources Information Center; 1966 to 11 January 2022).
10. Social Policy and Practice Ovid (last updated 202110). Searched 11 January 2022.
11. PTSDpubs Proquest (previously known as PILOTS; 1871 to 11 January 2022).
12. *Cochrane Database of Systematic Reviews* (CDSR 2022, Issue 1), in the Cochrane Library. Searched 10 January 2022.
13. Web of Science Core Collection: Citation Indexes Clarivate (Science Citation Index, Social Sciences Citation Index, Conference Proceedings Citation Index ‐ Science and Conference Proceedings Citation Index ‐ Social Science & Humanities; 1970 to 11 January 2022).
14. Epistemonikos (www.epistemonikos.org; searched 12 January 2022).
15. ClinicalTrials.gov (www.ClinicalTrials.gov; searched 12 January 2022).
16. WHO International Clinical Trials Registry Platform (ICTRP; apps.who.int/trialsearch; searched 12 January 2022).
17. Be Part of Research (replaced UK Clinical Trials Gateway; www.bepartofresearch.nihr.ac.uk; searched 12 January 2022).

#### Searching other resources

##### Personal communication

We contacted a wide range of triallists and experts in the field regarding published, unpublished and ongoing research and to ask for further trial data where applicable.

##### Reference lists

We examined the reference lists of all included studies and relevant systematic reviews to identify additional studies from the electronic searches (for example, unpublished or in‐press citations).

##### Supplementary searches

Supplementary searches were conducted through to February 2022. We conducted a forward citation search of included studies using Web of Science.

### Data collection and analysis

#### Selection of studies

Titles and abstracts of all records identified through the searches were each assessed against the inclusion criteria (Criteria for considering studies for this review) by two of five authors/researchers (NK, KB, SB, GC, LOD) working independently, and were coded as 'yes' (eligible), 'no' (not eligible) or 'maybe' (potentially eligible or unclear).

We retrieved full‐text reports for those titles and abstracts identified as eligible or potentially eligible and two review authors (SB and LOD) independently assessed each report against the inclusion criteria (Criteria for considering studies for this review). Studies were identified for either inclusion or exclusion. We contacted study authors, as required, to decide whether the inclusion criteria were met. We recorded reasons for excluding studies. In the event of disagreements, the authors discussed the papers and reasons for the decisions, with final decisions being made by consensus and with input from a third author when needed (GF, KH, KB, MW).

We identified and excluded duplicate records and collated multiple reports that related to the same study, so that each study, rather than each report, is the unit of interest in the review. We recorded the selection process in sufficient detail to complete a four‐phase (identification, screening, eligibility and included) PRISMA flow diagram for study collection (Moher 2009).

#### Data extraction and management

We used Covidence as a platform to upload the included studies and extract data (Covidence 2018), and export data into Review Manager 5 (RevMan 5) (Review Manager 2014). After the review was checked into RevMan 5, Review Manager Web (RevMan Web) allowed us to analyse the data and build the text, tables and figures for presenting the review (Review Manager Web 2019). We generated a PRISMA diagram report.

We piloted and refined the data collection form using the first five studies included in the review. Two authors (LOD with KB, SB, GC), working independently, extracted all data on key characteristics, methods and outcomes from each included study, and compared their results to identify differences. Where differences were identified, we resolved them by consensus or by referral to another member of the review team (KB, SB, GC). When further clarification or missing data were needed from study authors, we made all reasonable attempts to contact the study authors and obtain the relevant information.

Specifically, we extracted data on the following characteristics from each included study.

1. Methods: brief description of study design and randomisation method; dates or total duration of study; location of study.
2. Participants: baseline characteristics, including gender, age, ethnicity, disability, markers of opportunity/deprivation; recruitment setting; inclusion and exclusion criteria; group differences; number of eligible people recruited and assigned; attrition; numbers analysed.
3. Interventions: number of intervention groups and sessions; type of psychosocial intervention; mode of delivery; frequency and duration of delivery; format (i.e. group, individual or a blend); level of training of person delivering the intervention; relevant comparator intervention characteristics; treatment completion rates.
4. Outcomes: primary and secondary outcomes; outcome measures used; timing of outcome measurement (i.e. post‐treatment, 3 months, 6 months or 12 months); mean, standard deviation, number of events and sample size.
5. Notes: funding for trial; notable conflicts of interest of trial authors.

One review author (MW) transferred the data into RevMan 5 (Review Manager 2014). Another review author (LOD) independently checked the data extraction forms for accuracy and completeness.

#### Assessment of risk of bias in included studies

##### Randomised parallel‐group trials

We undertook our risk of bias assessment using RevMan Web (Review Manager Web 2019) and according to Cochrane's revised risk of bias tool for randomised trials (RoB 2) (Higgins 2021a; Sterne 2019) and using the suite of templates and tools available online (Higgins 2019). The review aimed to assess the effect of assignment to intervention ‐ the 'intention‐to‐treat' effect. We assessed the risk of bias for each result arising from studies that reported our primary outcomes (i.e. treatment efficacy based on PTSD and depression, treatment acceptability and adverse effects). We applied RoB 2 to any result involving our primary outcome at post‐treatment. Three review authors (LOD with LT and MW) independently undertook these assessments. Any disagreement was resolved by discussion and involving a third review author (LT and MW).

For a single trial result, we responded to a series of 'signalling' questions covering five domains.

1. Risk of bias arising from the randomisation process.
2. Risk of bias due to deviations from the intended interventions (effect of assignment to intervention).
3. Risk of bias due to missing outcome data.
4. Risk of bias in measurement of the outcome.
5. Risk of bias in the selection of the reported result.

We selected one of the five response options to each question (‘yes’, ‘probably yes’, ‘probably no’, ‘no’ and ‘no information’). We used these responses to reach a judgement of low, some or high concerns. The final step was to combine these responses for the five domains to reach an overall rating of low risk of bias, some or high risk of bias for the result. When considering treatment effects, we took into account the risks of bias of the results contributing to that effect.

##### Cluster‐randomised parallel‐group trials

We assessed the risk of bias of cluster‐randomised trials in line with Section 23.1.2 of the *Cochrane Handbook for Systematic Reviews of Interventions* (Higgins 2021b), assessing each study for risk of bias across the five domains listed below.

1. Bias arising from the randomisation process.
2. Bias due to deviation arising from intended interventions.
3. Bias due to missing outcome data.
4. Bias in the measurement of outcome.
5. Bias in the selection of the reported outcome.

We also examined bias arising from identification or recruitment of individual participants within clusters.

##### Quasi‐experimental

In assessing the risk of bias in quasi‐randomised studies, we applied the same methods as those recommended for randomised trials, in line with Cochrane guidance (Reeves 2021) and new guidance from Sterne and colleagues (Sterne 2019). Generally, we judged such studies to be at high risk of bias arising from the randomisation process.

#### Measures of treatment effect

We imported the data for each study and outcome entered into Covidence (Covidence 2018) into RevMan 5 (Review Manager 2014). We used RevMan Web to perform meta‐analyses and present results in graph form (Review Manager Web 2019).

##### Dichotomous data

While primary and secondary outcomes are usually assessed with continuous measures, we expected that some investigators would have presented dichotomous data on these outcomes. We required counts and percentages by trial arm for each study that reported dichotomous outcomes (e.g. dropout or adverse events). Using the summary data, we calculated the pooled risk ratio (RR) and 95% confidence intervals (CI) across the studies for each outcome.

##### Continuous data

We required means and standard deviations by study arm for studies that reported continuous outcomes. When studies used different outcome measures to assess the same construct, we calculated standardised mean differences (SMD) and 95% CI as the measure of effect (Schünemann 2011). We expected outcomes to be measured with a range of tools (see Types of outcome measures) across studies, and that we would largely be calculating SMD. We used Cohen's general rule of thumb to interpret effect sizes computed using the SMD, where 0.2 represents a small effect, 0.5 represents a medium effect, and 0.8 or larger represents a large effect (Cohen 1988).

#### Unit of analysis issues

##### Studies with multiple treatment groups

When studies compared multiple eligible experimental interventions with a single control group, we split the control group to enable pairwise comparisons. This led to having more comparisons than studies. For continuous outcomes, we split the sample size by the number of eligible experimental conditions but kept the mean and standard deviation consistent. For dichotomous outcomes, we split the sample size and the number of events by the number of eligible experimental conditions. If studies used multiple control groups, we combined the control groups to compare them to the experimental intervention group.

#### Dealing with missing data

Where data were missing, we followed the recommendations outlined in the *Cochrane Handbook for Systematic Reviews of Interventions* (Higgins 2021b). We classified data as either 'missing at random' or 'not missing at random'. Where we considered data to be missing at random, we analysed the available data. For data that we considered not missing at random, we made every effort to contact study authors to gather the missing information. We asked questions in an open‐ended manner to prevent the skewing of responses (Higgins 2021b). We documented all correspondence with study authors. It was not possible to use analytical methods to handle missing data as we only collected summary data from the studies; we did not source individual level data from the study authors (Egger 2001). We highlighted any suppositions that we made during our analysis when data were unavailable.

#### Assessment of heterogeneity

Clinical heterogeneity refers to variability in the participants, setting, interventions and outcomes studied; methodological heterogeneity refers to variability in study design and risk of bias; and statistical heterogeneity refers to variability in the effects reported in the different studies. Statistical heterogeneity is a consequence of clinical or methodological heterogeneity, or both, among the studies and manifests in the observed intervention effects being more different from each other than one would expect due to random error (chance) alone.

We identified sources of clinical heterogeneity by constructing tables to summarise studies in terms of participants, setting, type of intervention, intervention delivery (e.g. group or individual, number of sessions) and outcomes examined. Where studies were similar, we conducted further analyses, initially by reviewing the consistency of the results across studies using graphical representations (Egger 1997). To initially identify the heterogeneity/inconsistency of the whole network, we used the Q statistic, separating the studies based on whether they shared the same design or not. We assessed statistical heterogeneity with the Chi^2^ test, which provided us with evidence of variation in effects, disregarding the effect of chance. The Chi^2^ test is ineffective for analysing heterogeneity in studies with only a small number of participants or trials, so we set our P value at 0.10 (Deeks 2021), and assessed heterogeneity using the I^2^ statistic, which found the percentage of variability due to heterogeneity outside of the effect of chance (Higgins 2003).

We interpreted the observed value of I^2^ using the guide given in Section 10.10.2 of the *Cochrane Handbook for Systematic Reviews of Interventions* (Deeks 2021), where 0% to 40% might not be important, 30% to 60% may represent moderate heterogeneity, 50% to 90% may represent substantial heterogeneity, and 75% to 100% shows considerable heterogeneity. We took into consideration the size and direction of effects and the strength of evidence for heterogeneity using the Chi^2^ test and the 95% CI for I^2^. With a small number of studies (< 20), both the I^2^ confidence interval and the Q test should be interpreted very cautiously (Huedo‐Medina 2006).

Where there was evidence for statistical heterogeneity, we used the strategies outlined in Section 10.10.3 of the *Cochrane Handbook for Systematic Reviews of Interventions* (Deeks 2021), to identify potential sources of heterogeneity among the results of the studies. In particular, we explored differences in the characteristics of the studies or other factors as possible explanations for heterogeneity in the results. We summarised any differences identified in the narrative summary. The significance of the I^2^ statistics observed will rely upon the effects of treatment and the quality of evidence suggesting heterogeneity.

We used RevMan 5 (Review Manager 2014) to produce forest plots and calculate Tau^2^, the between‐study variance in a random‐effects meta‐analysis (Deeks 2021; Review Manager 2014). To understand the intervention effects, we used Tau^2^ to identify a range for the primary outcome. We used the *Cochrane Handbook for Systematic Reviews of Interventions* as a guideline throughout this process (Deeks 2021).

#### Assessment of reporting biases

We attempted to locate the protocols or study records (or both) in trial registries of the RCTs included in the review. Where the protocol was available, we compared its outcomes against the published report; and where the protocol could not be found, we compared the outcomes included in the methods section of the trial report to the reported results. We identified outcome reporting bias where outcomes were included in the methods but not reported (Pocock 1987; Tannock 1996).

If there were 10 or more studies, we constructed funnel plots to investigate associations between effect size and study precision (which is closely related to sample size) (Egger 1997). We also applied Egger's regression asymmetry test to funnel plots to test for funnel plot asymmetry (Egger 1997). Such an association could be due to publication or related biases, or due to systematic differences between small and large studies. If we identified an association, we examined the clinical diversity of the studies as a possible explanation. If appropriate, we also conducted a sensitivity analysis to determine whether assumptions about the effect of the bias impact the estimated treatment effect and the conclusions of the review.

#### Data synthesis

We performed a meta‐analysis if there were sufficient data (three or more studies was selected as a threshold given the potential for a very large number of analyses if we pooled two or more studies and issues relating to statistical power when examining an outcome with a small number of studies) (Borenstein 2011). It also had to be meaningful to pool the data across studies; for instance, the treatments, comparisons, participants and the underlying measures needed to be similar enough for pooling to be appropriate. Our decision to perform a meta‐analysis was determined by the comparability of populations, denominators and interventions (clinical heterogeneity); the comparability of the duration of follow‐up (methodological heterogeneity); and the comparability of outcomes. We used a random‐effects model to analyse the data across the studies. The Mantel‐Haenszel method, a default program in RevMan 5 (Review Manager 2014), can take account of few events or small study sizes and can be used with random‐effects models. Studies were excluded from the meta‐analysis for the dichotomous outcomes when no events were reported in either arm of a trial as per Cochrane guidance Chapter 10.4.4.1 (Deeks 2021). We used the inverse‐variance method, another default program in RevMan 5 (Review Manager 2014), for continuous outcomes. This approach assumes that the different studies were estimating different, yet related, intervention effects.

We stratified results for the main comparison (psychosocial interventions versus inactive controls) by type of therapy (see Subgroup analysis and investigation of heterogeneity and Types of interventions), where there were sufficient numbers of studies of the same intervention type, comparison arm and reporting the same outcome. For other comparisons, comparing two experimental interventions (i.e. an intervention from one category against an intervention from another category), we required three or more studies comparing similar experimental interventions using similar outcomes.

#### Subgroup analysis and investigation of heterogeneity

We were keen to investigate intervention effects for subsets of interventions. To do this, we performed subgroup analyses on the category of intervention (i.e. Cognitive Behavioural Therapies, Behavioural Therapies and low‐intensity psychosocial interventions). We used a simple approach, described in Chapter 9.6.3 of the *Cochrane Handbook for Systematic Reviews of Interventions* (Deeks 2021), to investigate whether there was a difference in the intervention effect between the subgroups.

If we identified a considerable degree of heterogeneity (75% to 100%), we first checked the data for errors. If the data were correct, we conducted a sensitivity analysis by excluding certain studies from the existing meta‐analysis, assessing the influence of the studies on the degree of heterogeneity (see Sensitivity analysis).

#### Sensitivity analysis

We based our primary analyses on available data from all included studies relevant to the comparison of interest. In order to examine any effects of methodological decisions on the overall outcome, we performed the following sensitivity analyses, provided there were sufficient numbers of studies.

1. Re‐analysis of the data excluding studies with results at high risk of bias.
2. Re‐analysis of the data excluding studies with a high degree of heterogeneity.

#### Summary of findings and assessment of the certainty of the evidence

We created our summary of findings table(s) using GRADEpro GDT (GRADEpro GDT 2015) in RevMan Web (Review Manager Web 2019), and followed standard methods described in the *Cochrane Handbook for Systematic Reviews of Interventions* (Schünemann 2017). The table provides key information concerning the quality of evidence, the magnitude of effect of the interventions examined, and the sum of available data on primary outcomes. The table includes details relating to the participants, interventions, comparisons, outcomes, settings, length of the follow‐up and outcome measurement.

The key comparison for the summary of findings table is impact of psychosocial interventions versus inactive controls on treatment efficacy. For each outcome, we presented standardised effect size estimates and 95% CI. The primary outcomes for the review were: treatment efficacy measured by group differences on PTSD symptoms and on depressive symptoms, treatment acceptability (dropout from treatment) and adverse effects. We have reported treatment efficacy at post‐treatment, which constitutes a change in time point from the original protocol (Differences between protocol and review).

It was recognised that the main comparison combined all intervention types in one, which may lead to high levels of heterogeneity, and also that it may be more useful to stakeholders to understand effects by type. Thus, we conducted subgroup analyses to accompany Comparison 1 and added the result as a comment in the summary of findings table.

Two authors (LOD, KB) independently assessed the certainty of the evidence using the GRADE approach and included the results of this assessment in the summary of findings table. The level of certainty was defined by five factors: risk of bias; indirectness of factors (such as evidence, population, control, intervention and outcomes); inconsistency of results; imprecision of results (and large CI); and a high likelihood of publication bias. We downgraded all evidence by one level for a single factor up to a maximum of three levels for all factors. The final grade was determined by how likely the effect can be predicted. We assessed the certainty of the evidence on a four‐point scale, ranging from high (the real effect is close to what will be predicted) to very low (what actually happens is significantly varied from the predicted effect) (Schünemann 2017). Differences of opinion between the two authors were resolved through discussion and consulting a third author (SB).

We created the summary of findings table after data entry into RevMan 5 (Review Manager 2014), writing up our results and conducting the risk of bias assessment, but before writing our abstract, discussion and conclusions, to allow the opportunity to consider the impact of risk of bias in the studies contributing to each outcome on the mean treatment effect and our confidence in these findings.

## Results

### Description of studies

See: Included studies; Excluded studies; Studies awaiting classification; Ongoing studies.

#### Results of the search

Our electronic searches retrieved a total of 22,162 records. We found an additional 35 records from other sources. Once we had identified and deleted duplicate citations, the searches identified 15,250 records that were potentially relevant to the review. After screening title and abstracts, we retrieved the full texts for 243 records for further investigation. A total of 149 reports were excluded (See section on Excluded studies). In total, 36 studies (from 83 reports) met the inclusion criteria for the current review, and nine (from 10 reports) were categorised as ongoing studies that had not yet published outcomes (See Ongoing studies table), and one study was categorised as awaiting classification (See Studies awaiting classification table), see Figure 1. All were available in English and published in peer‐reviewed journals.

**1:**
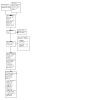
PRISMA flow diagram for selection of studies

#### Included studies

We included 36 studies in this review. Below, we summarise the key characteristics of the included studies.

##### Study design

Thirty‐five studies used a randomised controlled trial and one employed a cluster‐randomised controlled trial design (Bass 2013). The 36 studies included a range of active and comparative groups (Table 2).

**1:** List of studies by number of active and comparative arms

**Design**	**Number of studies**	**Studies**
2‐arm trial of intervention vs control	16	Abrahams 2010; Anderson 2010; Bass 2016; Bell 2019; Bomyea 2015; Bowland 2012; Brady 2021; Creech 2021; Falsetti 2008; Gray 2020; Krakow 2001; Littleton 2016; Miller 2015; Rajan 2020; Rothbaum 1997; Sikkema 2018
2‐ or 3‐active arm trial vs control group	7	Foa 1991; Foa 1999; Foa 2005; Foa 2006; Resick 2002; Rothbaum 2005; Walsh 2017
2‐ or 3‐active arm trial	13	Acierno 2021; Bass 2013; Belleville 2018; Covers 2021; Echeburua 1996; Feske 2008; Galovski 2016; Katz 2014; Kelly 2021; Nixon 2016; Resick 2008a; Schnurr 2007; Surís 2013

##### Sample sizes

The included studies randomised a total of 3992 individuals. The number of individuals randomised within the included studies ranged from 16 (Belleville 2018) to 405 (Bass 2013). Fifteen studies randomised more than 100 participants (Abrahams 2010; Acierno 2021; Bass 2013; Bass 2016; Creech 2021; Foa 2005; Galovski 2016; Kelly 2021; Krakow 2001; Miller 2015; Resick 2002; Resick 2008a; Schnurr 2007; Surís 2013; Walsh 2017). The number of individuals approached and randomised in each of the studies is described in the Included studies table.

##### Setting

Most studies (n = 26, 76%) were conducted in the USA; two were from South Africa (Abrahams 2010; Sikkema 2018), two were conducted in the Democratic Republic of the Congo (Bass 2013; Bass 2016) and there were single studies in Australia (Nixon 2016), Canada (Belleville 2018), the Netherlands (Covers 2021), Spain (Echeburua 1996), Sweden (Rajan 2020), and the UK (Brady 2021). Studies were published over a 30‐year period (1991 to 2021).

##### Characteristics of participants

###### Participants’ experience of violence

All 36 studies included participants who had experienced rape and sexual assault in adulthood. All but seven (Bell 2019; Bowland 2012; Brady 2021; Falsetti 2008; Feske 2008; Gray 2020; Schnurr 2007) had exposure to rape or sexual assault as an eligibility criterion. Overall, 82% of the participants counted in this review reported an experience of rape or sexual assault in adulthood (3260/3992). Seven studies with 887 participants included survivors of military sexual trauma (MST; 22% of all those randomised) (Acierno 2021; Creech 2021; Gray 2020; Katz 2014; Kelly 2021; Schnurr 2007; Surís 2013). The time since the index trauma ranged from < 72 hours (Miller 2015) to a mean of 16 years (SD 14 years) for a range of index traumas, which included sexual assault in adulthood (Galovski 2016).

###### Participants’ experience of PTSD

A diagnosis of PTSD was specifically indicated as an inclusion criterion in 21 studies. In the 26 studies that reported on proportions of the sample with probable PTSD at baseline, 94% of participants (2239/2370) had a clinician‐derived PTSD diagnosis or clinically relevant symptoms based on cut‐off for the Clinician‐Administered PTSD Scale (CAPS; Blake 1990) (10 studies); the PTSD Symptom Scale‐Interview (PSS‐I; Foa 1993) (six studies); the PCL‐5 (Blevins 2015) (four studies); or other assessments (six studies).

###### Participant socio‐demographic characteristics

Whilst 13 studies specified female gender in their inclusion criteria (Abrahams 2010; Bass 2013; Bass 2016; Bomyea 2015; Creech 2021; Feske 2008; Foa 2005; Galovski 2016; Krakow 2001; Rothbaum 1997; Schnurr 2007; Sikkema 2018; Walsh 2017), 99% (3965) of all participants were female. Just 27 male survivors, derived from four studies, were included (Brady 2021; Covers 2021; Nixon 2016; Surís 2013).

The average age of participants across all those randomised was 35.9 years (SD 9.6) (based on the data of 3467 participants). Overall, participant mean age ranged from 19.3 years (Anderson 2010) to 61.3 years old (Bowland 2012).

Half the randomised participants in studies furnishing data on cultural or ethnic background were Black, African or African‐American (1889/3815). Several studies had a majority of African‐American participants (Acierno 2021; Creech 2021; Feske 2008; Foa 2006; Kelly 2021) or were studies located in African countries (Abrahams 2010; Bass 2013; Bass 2016). Forty per cent of participants had White or Caucasian ethnicity (1530/3815) and 10% (396/3815) were from mixed backgrounds or other ethnicities. The one UK study included only migrant or asylum‐seeking people, representing nine nations (Brady 2021).

Five studies explicitly reported on disability. The way in which disability was reported varied across these five studies, and there was little detail about the nature of the disability. One study reported that one‐third of participants received disability payments (33% in Feske 2008). Two studies reported the proportion of participants who were disabled in the context of reporting about employment status (i.e. those that were unemployed and unable to work or find work (7.5% in Foa 2005; 53% in Katz 2014)). Two studies reported on approved disability status in the context of Veterans Affairs (21.8% 'PTSD disability' status approved in Schnurr 2007; 62% 'disability' status approved, 21% 'PTSD disability' status approved in Surís 2013). Based on these indicators alone, 180 individuals were reported to have a disability. It should be noted that 34 studies reported that they excluded participants from participating in the study if they had cognitive impairments, severe medical conditions or mental health difficulties that would prevent individuals from providing informed consent to take part. Thus, opportunities for diversity (including those affected by disabilities) may have been lost on account of rigorous inclusion criteria.

The socioeconomic status of participants was mixed. Several studies reported a high proportion of participants on low incomes (e.g. Bomyea 2015; Feske 2008; Foa 2005; Galovski 2016; Walsh 2017). Others reported attainment of college education (e.g. Creech 2021; Foa 1999; Foa 2005; Foa 2006; Gray 2020; Krakow 2001; Littleton 2016; Resick 2008a; Rothbaum 1997; Rothbaum 2005; Surís 2013). Two studies reported 100% of participants currently engaged in university education (Anderson 2010; Littleton 2016). In the 12 studies reporting employment status, between 9.6% and 77% of participants self‐reported being employed at study entry (Falsetti 2008; Feske 2008; Foa 1991; Foa 1999; Foa 2005; Gray 2020; Rothbaum 1997; Rothbaum 2005; Schnurr 2007; Sikkema 2018; Surís 2013; Walsh 2017).

Many of the participants were not partnered at study entry. Of the 22 studies reporting partner status, 21 reported 13.6% to 87.5% of participants having a partner (three exceeded 50%: Bass 2016; Bowland 2012; Sikkema 2018) and one reported that 4.8% of participants were married (Feske 2008). Studies did not address participants' sexual identities except for one reporting that 14% of the women in the study were bisexual, lesbian or other minority sexual orientation (Creech 2021).

The number of participants reporting comorbid conditions ranged from 52.4% (Bomyea 2015) to 95.2% (Feske 2008). Some studies reported specific prevalence of major depressive disorder (e.g. Bomyea 2015; Feske 2008; Foa 2005; Resick 2008a), anxiety disorders (e.g. Bomyea 2015; Falsetti 2008) and hazardous drinking (Creech 2021; Sikkema 2018).

###### Participant recruitment settings

Settings for recruitment of participants and delivery of the intervention were not always the same.

Recruitment settings were diverse: for 15 studies, this was mainly community settings (Bass 2013; Bass 2016; Bell 2019; Belleville 2018; Bomyea 2015; Bowland 2012; Echeburua 1996; Falsetti 2008; Foa 1991; Foa 2005; Foa 2006; Galovski 2016; Krakow 2001; Rajan 2020; Resick 2008a); five recruited through health and forensic sexual assault services or medico‐legal environments (Abrahams 2010; Covers 2021; Miller 2015; Nixon 2016; Walsh 2017); seven were clinics for veterans and active service people in the USA (Acierno 2021; Creech 2021; Gray 2020; Katz 2014; Kelly 2021; Schnurr 2007; Surís 2013); two were purely university student samples (Anderson 2010; Littleton 2016); two were outpatients/clinical settings (Feske 2008; Rothbaum 1997); one was a clinic for delivery of HIV medicine and primary care (Sikkema 2018) and one was a charity providing support for survivors of human rights abuses (Brady 2021). Three were unclear in terms of how or where participants were invited (Foa 1999; Resick 2002; Rothbaum 2005).

It was not uncommon for studies to utilise multiple recruitment strategies that included police (Foa 2006); victim agencies (Belleville 2018; Foa 1991; Foa 2006; Krakow 2001; Resick 2008a); counselling centres (Schnurr 2007); community therapists (Resick 2008a) and other professionals (Brady 2021); media (Foa 1991; Foa 2006); healthcare settings such as hospitals (Bowland 2012; Miller 2015; Schnurr 2007) and emergency departments (Foa 2006; Walsh 2017); mental health clinics (Gray 2020; Krakow 2001); and outpatient clinics (Feske 2008; Surís 2013).

##### Interventions

###### Types of interventions

We classified the experimental interventions according to the list of psychological therapies of the former Cochrane Depression, Anxiety and Neurosis (CCDAN) Group.

Across the 36 studies, there were a total of 60 active experimental conditions (3014 participants; 76%) and 23 inactive or minimally active comparator conditions (978 participants; 24%). The 60 experimental groups consisted of: 32 cognitive behavioural therapy (CBT); 10 behavioural interventions; three integrative therapies; three humanist; five other psychologically oriented interventions; and seven other psychosocial interventions.

See Table 2 for a list of studies according to the number of active and comparative trial arms. Sixteen studies consisted of a traditional two‐arm trial of an active intervention versus a control group. In relation to the active arm of these studies, four were CBT interventions (Anderson 2010; Falsetti 2008; Krakow 2001; Littleton 2016); and six were behavioural interventions ‐ EMDR (Rothbaum 1997); High Interference Control (Bomyea 2015); Narrative Exposure Therapy (Brady 2021); Reconciliation of Traumatic Memories study (Gray 2020); Lifespan Integration (Rajan 2020); biofeedback (Bell 2019). The remaining six were classified as ‘other psychosocial interventions’ or 'other psychologically oriented interventions': telephonic psychosocial support (Abrahams 2010); village savings and loans association (Bass 2016); spiritually focused group therapy (Bowland 2012); psychoeducation and coping video (Miller 2015); a brief motivational interviewing and psychoeducation‐based computerised intervention (Creech 2021) and an intervention to improve AIDS care after trauma (Sikkema 2018).

Seven studies compared either two (Foa 2005; Foa 2006; Resick 2002; Rothbaum 2005; Walsh 2017) or three (Foa 1991; Foa 1999) active interventions and included a control group. These included 16 active intervention groups, of which 11 were CBT; two were behavioural interventions ‐ EMDR (Rothbaum 2005) and pleasant imagery/relaxation video (Walsh 2017); two were humanist (supportive counselling) (Foa 1991; Foa 2006); and one was grouped as ‘other psychosocial interventions’ ‐ a prevention of post‐rape stress video (Walsh 2017).

The remaining 13 studies did not have a control condition; rather, they compared two (Acierno 2021; Bass 2013; Belleville 2018; Covers 2021; Echeburua 1996; Feske 2008; Galovski 2016; Kelly 2021; Nixon 2016; Schnurr 2007; Surís 2013) or three (Katz 2014; Resick 2008a) active conditions.

###### Comparisons

Of the 23 studies that included a control group, two used a no‐treatment control (Anderson 2010; Bowland 2012); two used an assessment condition (Creech 2021; Foa 2006); four compared usual or standard care (Abrahams 2010; Miller 2015; Sikkema 2018; Walsh 2017); four compared the active intervention(s) to a minimal intervention (Bell 2019; Bomyea 2015; Brady 2021; Littleton 2016) and the remaining 11 used wait‐list controls.

Occasionally, a study indicated 'usual care' or 'standard care' typical of the delivery setting, which we classified as sufficiently active to warrant placing it in an active category for analysis. For example, Nixon 2016 compared Cognitive Processing Therapy to treatment as usual in a sexual assault services setting; since the latter encompassed a range of methods, including psychoeducation, supportive counselling, problem‐solving, interpersonal therapy, elements of mindfulness, acceptance and value‐based techniques, and discussion of thoughts and feelings, we believed it was better analysed as active and classified it as 'integrative' (where therapists select models and methods from across orientations to best suit a particular client and context). Another study, also located in a sexual assault services setting, compared the experimental group to treatment‐as‐usual (TAU) at participating sexual assault centres (Covers 2021). This TAU consisted of two telephone contacts of approximately 30 minutes with a case manager, who provided psychoeducation and emotional support in accordance with a watchful waiting protocol (NICE 2018); thus, we defined the comparison as 'Other psychosocial intervention'. Lastly, we classified the treatment as usual condition in Feske 2008 as integrative therapy; it consisted of 9 to 12 sessions of the standard treatment provided at the clinic and included one weekly, hour‐long individual counselling session and a weekly, 90‐minute group treatment session (e.g. anger management).

####### Inactive controls

Twenty‐three studies had an inactive group.

- Abrahams 2010: usual care
- Anderson 2010: no treatment control
- Bass 2016: wait‐list
- Bell 2019: minimal intervention
- Bomyea 2015: minimal intervention
- Bowland 2012: no treatment control
- Brady 2021: minimal intervention
- Creech 2021: assessment control
- Falsetti 2008: wait‐list
- Foa 1991: wait‐list
- Foa 1999: wait‐list
- Foa 2005: wait‐list
- Foa 2006: assessment condition
- Gray 2020: wait‐list
- Krakow 2001: wait‐list
- Littleton 2016: minimal intervention
- Miller 2015: usual care
- Rajan 2020: wait‐list
- Resick 2002: wait‐list
- Rothbaum 1997: wait‐list
- Rothbaum 2005: wait‐list
- Sikkema 2018: usual care
- Walsh 2017: usual care

####### Active treatment

For the 13 studies that tested the performance of different interventions against one another, these involved a total of 28 different intervention groups.

- Acierno 2021: CBT (Prolonged Exposure via home‐based telemedicine) versus CBT (Prolonged Exposure via in‐person)
- Bass 2013 CBT (Cognitive Processing Therapy contained no in vivo exposure) versus other psychosocial interventions (individual support)
- Belleville 2018: CBT (Image Rehearsal Therapy + Prolonged Exposure) versus CBT (Prolonged Exposure only)
- Covers 2021: Behavioural (Eye Movement Desensitisation and Reprocessing) versus other psychosocial interventions (Telephonic psychosocial support)
- Echeburua 1996: CBT (Cognitive Restructuring + Coping Skills) versus behavioural (Progressive Relaxation)
- Feske 2008: CBT (Cognitive Processing Therapy) versus Integrative Therapy
- Galovski 2016: CBT (Cognitive Processing Therapy + Hypnosis) versus CBT (Cognitive Processing Therapy)
- Katz 2014: CBT (Prolonged Exposure) versus humanist (Person‐centred Therapy) versus Integrative Therapy (Holographic Reprocessing)
- Kelly 2021: Other psychologically oriented interventions (Trauma‐sensitive Yoga) versus CBT (Cognitive Processing Therapy)
- Nixon 2016: CBT (Cognitive Processing Therapy) versus Integrative Therapy
- Resick 2008a: CBT (Cognitive Processing Therapy) versus CBT (Cognitive Therapy) versus CBT (Written Exposure)
- Schnurr 2007: CBT (Prolonged Exposure) versus other psychologically oriented interventions (Present‐centred Therapy)
- Surís 2013: CBT (Cognitive Processing Therapy) versus other psychologically oriented interventions (Present‐centred Therapy)

###### Formats of interventions

For the 60 active intervention groups, 41 interventions used individual and face‐to‐face delivery; nine used a group and face‐to‐face approach (in Bass 2013; Bass 2016; Bowland 2012; Falsetti 2008; Feske 2008; the two groups in Kelly 2021; Krakow 2001; Sikkema 2018); six involved computer use and no interpersonal element (in Bell 2019; Bomyea 2015; Creech 2021; Miller 2015; two in Walsh 2017); and the final four were individual and used telephone (Abrahams 2010); teleconference (Acierno 2021); or online (Littleton 2016) modalities.

To deliver the intervention, 25 studies ensured the intervention delivery personnel were qualified either via qualifications, training or prior experience (Abrahams 2010; Anderson 2010; Bass 2013; Bass 2016; Bowland 2012; Brady 2021;Covers 2021; Echeburua 1996; Falsetti 2008; Feske 2008; Foa 1991; Foa 1999; Foa 2005; Foa 2006; Gray 2020; Katz 2014; Kelly 2021; Krakow 2001; Nixon 2016; Rajan 2020; Resick 2002; Resick 2008a; Rothbaum 1997; Schnurr 2007; Surís 2013). For three studies, this detail was not applicable due to video or computerised delivery (Creech 2021; Miller 2015; Walsh 2017), whilst eight studies did not provide sufficient information (Acierno 2021; Bell 2019; Belleville 2018; Bomyea 2015; Galovski 2016; Littleton 2016; Rothbaum 2005; Sikkema 2018).

###### Number of sessions and duration of interventions

The median number of *planned* sessions across the 60 interventions was 11, and this ranged from 1 session to 20 sessions. In studies that reported the mean number of sessions *completed*, this ranged from 1 to 17 (Brady 2021) completed sessions. It was possible to compare the number of sessions planned with the number of sessions completed. For example, in both active arms of the Acierno 2021 study, 12 to 15 sessions were planned whilst 6.80 (SD 4.14) sessions were completed in the prolonged exposure via home‐based telemedicine group and 6.28 (SD 4.33) sessions were completed in the group that received face‐to‐face prolonged exposure. The most sessions delivered in any study was 26 (Brady 2021).

The length of time over which interventions were delivered ranged from one week (e.g. Walsh 2017) to 40 weeks (Bass 2016). In 29 studies, interventions were delivered over 12 or fewer weeks. In seven studies, intervention sessions were delivered over 13 to 40 weeks (Acierno 2021; Bass 2013; Bass 2016; Belleville 2018; Brady 2021; Galovski 2016; Littleton 2016).

The reported total amount of time that individuals spent in an intervention ranged from nine minutes (Miller 2015; Walsh 2017) to 27 hours and 30 minutes (Belleville 2018). Some studies reported that individuals were asked to do 'homework' or activities outside of the intervention. For example, Rothbaum 2005 reported how individuals participating in the prolonged exposure intervention were asked to listen to tape recordings of their trauma narratives (which lasted 45 to 60 minutes) on a daily basis over five weeks. Resick 2002 reported the number of hours in which the intervention involved homework and this ranged from 22.6 hours to 44.8 hours in the cognitive processing therapy and prolonged exposure interventions respectively.

###### Delivery setting

In addition to the recruitment settings, we considered the settings where the interventions took place. The majority of studies (11/36) delivered interventions in academic settings (Belleville 2018; Bomyea 2015; Bowland 2012; Foa 1991; Foa 1999; Galovski 2016; Krakow 2001; Resick 2002; Resick 2008a; Rothbaum 1997; Rothbaum 2005). Feske 2008 used a university‐affiliated outreach clinic. Of the seven that recruited veterans and active service individuals (Acierno 2021; Creech 2021; Gray 2020; Katz 2014; Kelly 2021; Schnurr 2007; Surís 2013), six delivered interventions in American Veterans Affairs settings, such as medical centres, and one used a dedicated private office space for delivery of the intervention (Gray 2020). Five studies provided the interventions in an acute setting for forensic and medical care after rape or sexual assault (Abrahams 2010; Covers 2021; Miller 2015; Nixon 2016; Walsh 2017). Counselling services and clinics were used by three studies (Echeburua 1996; Foa 2006; Rajan 2020). Sikkema 2018 offered their intervention in a primary care setting for those with HIV. The two studies by Bass and colleagues were delivered in the community (Bass 2013; Bass 2016). The university cohorts received their interventions in the university setting (Anderson 2010; Littleton 2016). Foa 2005 used a combination of a community clinic for rape survivors and a centre dedicated to treatment and study of anxiety. Similarly, Falsetti 2008 delivered their intervention at a centre dedicated to the care and support of individuals who have been victims of crime. Brady 2021 delivered their intervention in charity‐based therapeutic services. Although not entirely clear, Bell 2019 likely used an experimental setting for their study of neurofeedback versus biofeedback.

##### Outcomes

###### Primary outcomes

All 36 studies reported on one or more of our primary outcomes. A summary is provided in Table 3 of which studies assessed the four primary outcomes (PTSD symptoms, depressive symptoms, treatment dropout and adverse events). Most studies measured PTSD symptoms using PSS‐I (e.g. Foa 1999; Gray 2020; Littleton 2016), CAPS (e.g. Bomyea 2015; Resick 2002) and PCL‐5 (e.g. Bell 2019; Sikkema 2018). Studies reported a variety of validated questionnaires, including BDI‐I (e.g. Bomyea 2015; Falsetti 2008; Foa 1999; Resick 2002); BDI‐II (e.g. Feske 2008); GDS (e.g. Bowland 2012); PHQ‐9 (e.g. Brady 2021) and CES‐D (e.g. Littleton 2016). Outcomes were either self‐reported by participants or observer‐rated by clinicians. We only extracted outcomes reported at the end of treatment and at three, six and 12 months follow‐up. Studies used a variety of scales to assess the primary and secondary outcomes; where appropriate, we chose the measure that was used most often, to reduce heterogeneity of outcomes.

**2:** List of included studies reporting primary outcome data

**Primary outcome**	**Number of studies**	**Studies**
PTSD symptoms	32	Acierno 2021; Anderson 2010; Bass 2013; Bass 2016; Bell 2019; Belleville 2018; Bomyea 2015; Covers 2021; Creech 2021; Echeburua 1996; Falsetti 2008; Feske 2008; Foa 1991; Foa 1999; Foa 2005; Foa 2006; Galovski 2016; Gray 2020; Katz 2014; Kelly 2021; Krakow 2001; Littleton 2016; Miller 2015; Nixon 2016; Rajan 2020; Resick 2002; Resick 2008a; Rothbaum 1997; Schnurr 2007; Sikkema 2018; Surís 2013; Walsh 2017
Depressive symptoms	23	Abrahams 2010; Acierno 2021; Bass 2016; Bomyea 2015; Bowland 2012; Covers 2021; Echeburua 1996; Falsetti 2008; Feske 2008; Foa 1991; Foa 1999; Foa 2005; Foa 2006; Galovski 2016; Katz 2014; Littleton 2016; Nixon 2016; Resick 2002; Resick 2008a; Rothbaum 1997; Rothbaum 2005; Schnurr 2007; Surís 2013
Dropout	32	Abrahams 2010; Acierno 2021; Bass 2013; Bell 2019; Bomyea 2015; Bowland 2012; Covers 2021; Creech 2021; Echeburua 1996; Falsetti 2008; Feske 2008; Foa 1991; Foa 1999; Foa 2005; Foa 2006; Galovski 2016; Gray 2020; Katz 2014; Kelly 2021; Krakow 2001; Littleton 2016; Miller 2015; Nixon 2016; Rajan 2020; Resick 2002; Resick 2008a; Rothbaum 1997; Rothbaum 2005; Schnurr 2007; Sikkema 2018; Surís 2013; Walsh 2017
Adverse events	16	Abrahams 2010; Acierno 2021;Brady 2021; Covers 2021; Foa 2005; Feske 2008; Gray 2020; Katz 2014; Kelly 2021; Krakow 2001; Littleton 2016; Nixon 2016; Rajan 2020; Resick 2008a; Schnurr 2007; Surís 2013

We included studies in adverse events if they reported a count of adverse events affecting participants. PTSD: post‐traumatic stress disorder

###### Secondary outcomes

Twenty‐four of the 36 included studies addressed one or more of the secondary outcomes defined in this review. There was a large variety of validated questionnaires used to capture anxiety, including STAI‐state (e.g. Rothbaum 2005), STAI‐trait (e.g. Foa 1999; Rothbaum 1997) and BAI (e.g. Bowland 2012; Feske 2008; Foa 2006). Studies reported global mental health functioning/distress using several different questionnaires, including Brief Symptom Inventory (e.g. Feske 2008) and GHQ12 (e.g. Rajan 2020). Trauma‐related beliefs such as feelings of guilt, shame or self‐blame in the aftermath of sexual assault were assessed using measures like Rape Aftermath Symptom Scale (e.g. Rothbaum 1997) and the Post‐traumatic Cognitions Inventory (e.g. Katz 2014). Substance use was measured in a small number of studies using tools such as the AUDIT (e.g. Creech 2021). In Table 4, we provide a summary of which studies assessed the secondary outcomes. No studies assessed self‐harm or suicidality or sexual violence outcomes.

**3:** List of included studies reporting secondary outcome data

**Outcome**	**Number of studies**	**Studies**
Anxiety symptoms	17	Bass 2016; Bell 2019; Bomyea 2015; Bowland 2012; Covers 2021; Echeburua 1996; Feske 2008; Foa 1991; Foa 1999; Foa 2006; Katz 2014; Littleton 2016; Miller 2015; Resick 2008a; Rothbaum 1997; Rothbaum 2005; Schnurr 2007
Dissociation symptoms	2	Covers 2021; Rothbaum 2005
Global mental health functioning/distress	10	Anderson 2010; Bass 2013; Bass 2016; Belleville 2018; Covers 2021; Feske 2008; Katz 2014; Rajan 2020; Rothbaum 1997; Schnurr 2007
Trauma‐related beliefs	9	Bass 2013; Bass 2016; Covers 2021; Echeburua 1996; Foa 1991; Foa 2006; Katz 2014; Resick 2002; Resick 2008a
Substance use	3	Creech 2021; Schnurr 2007; Walsh 2017
QoL	1	Schnurr 2007

QoL: quality of life

##### Study funding sources

The 36 included studies were funded by a variety of sources, including research funding bodies such as National Institute of Mental Health (Acierno 2021; Bomyea 2015; Falsetti 2008; Feske 2008; Foa 1991; Foa 1999; Foa 2005; Foa 2006; Galovski 2016; Krakow 2001; Littleton 2016; Resick 2002; Resick 2008a; Rothbaum 2005; Sikkema 2018), South African Medical Research Council (Abrahams 2010), Blue Angels Foundation (Gray 2020), Australian Rotary Health Research Fund (Nixon 2016), the Oak Foundation (Brady 2021) and National Institute on Drug Abuse (Walsh 2017). Four studies reported university financial support (Echeburua 1996; Krakow 2001; Rothbaum 1997; Sikkema 2018); one reported a particular centre (Miller 2015) and a recent trial of EMDR (Covers 2021) reported several sources in the charity and private sectors ‐ Achmea Association Victims & Society, Innovatiefonds Zorgverzekeraars, EMDR Research Foundation, Vereniging EMDR Nederland, and PAOS fonds. Governmental bodies were reported by seven studies (Acierno 2021; Bass 2013; Bass 2016; Creech 2021; Kelly 2021; Rajan 2020; Schnurr 2007; Surís 2013). Five studies did not disclose their sponsorship source (Anderson 2010; Bell 2019; Belleville 2018; Bowland 2012; Katz 2014).

#### Excluded studies

In summary, 149 reports were excluded as irrelevant. The reasons for exclusion were grouped as 'studies not randomised' (57); 'ineligible populations' (73); and 'ineligible interventions' (19). Of these, we selected 31 studies that required closer examination before being excluded on the basis of ineligible population. These are reported in the Excluded studies table.

#### Studies awaiting classification

One study, Dutton 2021, is awaiting classification as more information is required from the researchers to make a decision about inclusion (see Studies awaiting classification table).

#### Ongoing studies

Nine ongoing studies were identified in the searches. A list of these studies is provided below, with more comprehensive details of the studies outlined in the Ongoing studies table.

- NCT02808468: Brief Cognitive Therapy versus assessment only
- NCT04124380: Imaginal Exposure then alcohol skills training versus alcohol skills training then Imaginal Exposure versus alcohol skills training, no additional treatment versus Imaginal Exposure, no additional treatment versus supportive telehealth
- NCT03429166: Skills Training in affective and interpersonal regulation versus Present‐centred Therapy
- NCT03794986: Motivational Interviewing versus Motivational Interviewing with trauma‐informed sexual gender minorities affirmative care
- NCT03703258: app‐based Cognitive Behavioural Intervention versus assessment only
- ISRCTN16806208: online Cognitive Therapy versus internet‐delivered Stress Management versus wait‐list
- NCT03019497: Cognitive Behavioural Therapy with specific modules about a specific related problem versus Cognitive Behavioural Therapy without specific modules
- NCT04582695: Written Exposure Therapy integrated with CBT versus Written Exposure Therapy
- IRCT20120619010063N8: Mindfulness‐based art therapy versus wait‐list

### Risk of bias in included studies

We performed risk of bias assessment using the RoB 2 tool for all primary outcomes (where data were provided) and summarised the results of this assessment in the results level RoB 2 tables. Note that some studies produced more than one result for the same outcome in instances where there were multiple experimental arms that could be individually compared to the control arm. Most studies provided sufficient information to allow for potential risk of bias assessment with regard to PTSD (for details, see link), depression (for details, see link) and, to a lesser degree, treatment completion (for details, see link) and adverse events (for details, see link).

For PTSD at post‐treatment, there were 21 results to assess from 16 studies. We assumed an overall risk of bias with some concerns: half the overall results were judged to be of some concern, 43% (9 results from 6 studies) were at high risk (Bell 2019; Falsetti 2008; Foa 1999; Foa 2005; Miller 2015; Rothbaum 1997) and two studies at low risk (Creech 2021; Gray 2020). Studies performed relatively well on the measurement of PTSD (86% low risk), on deviation from the intervention (71% low risk) and on the selection of the reported result (33% low risk). However, seven out of 21 PTSD results were at high risk of bias with regard to randomisation (Bell 2019; Falsetti 2008; three comparisons from Foa 1999; Miller 2015; Rothbaum 1997) and four for missing data (Falsetti 2008; two comparisons from Foa 2005; Miller 2015).

For depression at post‐treatment, we assessed 17 results from 12 studies. We assumed an overall high risk of bias for depression on the basis that 59% of results were judged at high risk (Abrahams 2010; Falsetti 2008; three comparisons from Foa 1999; two from Foa 2005; Rothbaum 1997; two comparisons from Rothbaum 2005) and 41% raised some concerns (Bomyea 2015; Bowland 2012; Brady 2021; Foa 2006; Littleton 2016; two from Resick 2002). Measurement of depression was at low risk for all 17 results. Also, selection of the reported result was at low risk in 18% of results (Brady 2021; and the two results arising from Resick 2002) and of some concern for the remaining 82%. For randomisation, the level of risk of bias was similar to the PTSD outcome with seven results from four studies being at high risk (Falsetti 2008; Foa 1999; Rothbaum 1997; Rothbaum 2005). For deviations from the intended intervention, two results were at high risk (Abrahams 2010; Falsetti 2008). For missing data, three results were at high risk (Falsetti 2008; two comparisons from Foa 2005).

For treatment dropout, we assessed five results from five studies. We assumed an overall risk of bias with some concerns: 60% of studies generated some concerns (Bomyea 2015; Brady 2021; Foa 2006) and 40% were high risk (Bell 2019; Littleton 2016). Treatment dropout generally worked more effectively as an outcome in comparisons of active interventions, given the need for intervention completion data; it was less meaningful to assess dropout from usual care, wait‐lists or no‐treatment controls. Therefore, only included in this assessment are those five studies with comparators that we considered minimal interventions and for which studies reported dropout. Measurement issues led to the high risk assessment for Littleton 2016, whilst a lack of information about randomisation led to the high risk assessment for Bell 2019. Treatment dropout should be relatively straightforward to report and assess, as merely a count of those who completed the intervention expressed as a proportion of the numbers randomised. This outcome was at low risk for deviations from the intended interventions and bias due to missing data. However, it was complicated by poor reporting of data in flow diagrams and lack of clarity around thresholds for completion (e.g. how many sessions needed to be completed to be regarded as treatment completion?). Where these cut‐offs have not been stated prior to commencing the study, researchers may select thresholds that suit the conclusions they hope to demonstrate (be that favourable completion rates or tolerance of the intervention or to, in some way, influence who gets included in per protocol analyses).

For adverse events, there were six studies in Comparison 1 reporting results for adverse events/effects quantitatively by group. We assumed an overall risk of bias with some concerns with 50% of studies generating some concerns (Abrahams 2010; Brady 2021; Gray 2020). We assessed Rajan 2020 as being at low risk. Two studies were at high risk due to missing outcome data, which meant we could not assess why people had exited studies or if negative outcomes or experiences correlated with high attrition (Krakow 2001; Littleton 2016). On the other hand, these six were the only studies out of 23 studies in the main comparison that reported adverse events by arm.

### Effects of interventions

See: Table 1

See: Table 1 and forest plots. We undertook two sets of comparisons. The first set compares psychosocial interventions with inactive control arms only and includes subgroup analyses by intervention types (versus controls).

The second set of comparisons represents head‐to‐head comparison of active interventions, incorporating the set of studies that compared trauma‐focused interventions to any other intervention that did not involve exposure to the trauma (i.e. non‐trauma‐focused psychosocial interventions).

#### Comparison 1. Psychosocial interventions versus inactive controls

##### Primary outcomes

###### PTSD symptoms

A total of 16 studies reported PTSD symptoms for this comparison post‐treatment (Bell 2019; Bomyea 2015; Brady 2021; Creech 2021; Falsetti 2008; Foa 1999; Foa 2005; Foa 2006; Gray 2020; Littleton 2016; Miller 2015; Rajan 2020; Resick 2002; Rothbaum 1997; Sikkema 2018; Walsh 2017). Seven studies also compared PTSD symptoms at three months, two studies at six months and only one study at 12 months follow‐up. These studies consistently reported means and standard deviations, which enabled pooling.

We pooled all available studies post‐treatment into a random‐effects meta‐analysis. In the pooled analysis, there was a standardised mean difference (SMD) of −0.83 (95% CI −1.22 to –0.44; P < 0.001, I^2^ = 87%; 16 studies, 1130 participants; low‐certainty evidence; Analysis 1.1), favouring the experimental group. Using Cohen’s D as a basis for interpreting the SMD, this suggests a large effect. The three‐month time point revealed a SMD of –0.13 (95% CI ‐0.42 to 0.17; P = 0.41, I^2^ = 72%; 7 studies, 770 participants; Analysis 1.2), suggesting there may be no difference between groups at three months. An insufficient number of studies was available for the six‐month and 12‐month time points to conduct analyses.

We observed a tendency towards symmetrical funnel plots in Figure 2, disregarding possible reporting and publication biases.

**2:**
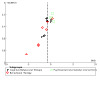
Funnel plot for the comparison of psychosocial interventions versus inactive control for PTSD symptoms at post‐treatment

Sensitivity analyses for PTSD symptoms

For the post‐treatment time point, we performed separate sensitivity analyses removing high risk of bias studies to reveal a SMD of −0.66 (95% CI −1.21 to ‐0.12; P = 0.02, I^2^ = 90%; 10 studies, 710 participants). In another sensitivity analysis, given the high heterogeneity amongst all studies reporting PTSD symptoms at post‐treatment (I^2^ = 87%; P < 0.001), we removed studies that were clinically diverse in terms of being low‐intensity interventions (i.e. they did not involve psychotherapy); this revealed a SMD of −1.13 (95% CI −1.57 to −0.70; P < 0.001, I^2^ = 84%; 13 studies, 808 participants; analysis not shown). Thus, there was a large effect size but no detectable improvement in heterogeneity, suggesting it may arise from additional methodological and clinical characteristics.

###### Depressive symptoms

Depressive symptoms were reported by 12 studies at post‐treatment for this comparison (Abrahams 2010; Bomyea 2015; Bowland 2012; Brady 2021; Falsetti 2008; Foa 1999; Foa 2005; Foa 2006; Littleton 2016; Resick 2002; Rothbaum 1997; Rothbaum 2005). We pooled depressive symptom scores into a random‐effects meta‐analysis. The result of the analysis at post‐treatment favoured the experimental group with a SMD of −0.82 (95% CI −1.17 to −0.48; P < 0.001, I^2^ = 78%; 12 studies, 901 participants; low‐certainty evidence; Analysis 1.3). Using Cohen’s D as a basis for interpreting the SMD, this suggests a large effect. For the three‐month time point, we calculated a SMD (SMD –0.05, 95% CI −0.39 to 0.29; P = 0.77, I^2^ = 38%; 3 studies, 376 participants; Analysis 1.4), which suggested no group difference. There was an insufficient number of studies to pool data for the other two time points.

We observed a tendency towards symmetrical funnel plots in Figure 3, disregarding possible reporting and publication biases.

**3:**
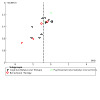
Funnel plot for the comparison of psychosocial interventions versus inactive control for depressive symptoms at post‐treatment

Sensitivity analyses for depressive symptoms

We performed separate sensitivity analyses removing high risk of bias studies for the post‐treatment time point to reveal a SMD of ‐0.65 (95% CI −1.16 to ‐0.13; P = 0.01, I^2^ = 79%; 6 studies, 360 participants). For the second sensitivity analysis we removed studies that were clinically diverse in terms of being low‐intensity interventions. This revealed a SMD of −0.90 (95% CI −1.28 to −0.52; P < 0.001, I^2^ = 76%; 10 studies, 718 participants; analysis not shown). There was no detectable improvement in heterogeneity, suggesting it arises from additional methodological and clinical characteristics.

###### Dropout from treatment

Dropout from treatment could be assessed in the five studies (242 participants) that compared psychosocial interventions to control groups that were defined as minimal interventions (Bell 2019; Brady 2021; Bomyea 2015; Foa 2006; Littleton 2016). We conducted a random‐effects meta‐analysis and revealed a risk ratio of 0.85 (95% CI 0.51 to 1.44; P = 0.55, I^2^ = 35%; low‐certainty evidence; Analysis 1.5), suggesting there was a lack of evidence about how exposure to the groups may affect dropout from treatment.

###### Adverse events

We considered adverse events within the 23 studies comparing a psychosocial intervention to an inactive control. Twenty‐one adverse events were reported in seven studies (801 participants) (Abrahams 2010; Brady 2021; Foa 2005; Gray 2020; Krakow 2001; Littleton 2016; Rajan 2020). Only six of the studies (622 participants) presented data by condition: three had zero adverse events in either condition (Abrahams 2010; Gray 2020; Rajan 2020) and three had minimal adverse events (Brady 2021; Krakow 2001; Littleton 2016). Upon pooling the six studies, a RR of 1.92 was revealed (95% CI 0.30 to 12.41; P = 0.49, I^2^ = 30%; 6 studies; 622 participants; very low‐certainty evidence; Analysis 1.6), suggesting there was a lack of evidence of a group difference.

In addition to counts of adverse events, and as Table 5 shows, studies considered harm experiences by examining rates of non‐completion of the intervention, and reporting the reasons for this. However, studies lacked feedback from large numbers of non‐completers and those that dropped out from follow‐up assessments, thus potentially missing harms of exposure to interventions and/or research participation. It was more common for studies to report on participation bias by comparing treatment completers and non‐completers on baseline factors, which is a related but distinct issue. The third approach to considering harm was to report worsening of symptoms based on outcome measures (usually PTSD). Finally, a small number of studies observed levels of distress or negative affect following therapy sessions; however, a temporary increase in negative mood was not necessarily viewed as problematic, rather, being part of a pathway to improved health.

**4:** List of included studies in Comparison 1 reporting adverse events data

**Study**	**Approach to reporting adverse events**	**Count**
Abrahams 2010	Study explicitly states that there were no recorded adverse events.	0/136 telephonic psychosocial support vs 0/138 minimal intervention
Anderson 2010	Study highlights that, in terms of emotional processes at the session level, women in the CBT (CAED) group reported significantly greater levels of negative affect following each of the sessions (except Session 3), relative to the control group; however, it does not provide the numbers. It states, "Hence, overall the CAED sessions appeared to achieve the expected increase in negative affect." Beyond this, the study lacks specific information about recording of or actual adverse events and harms.	—
Bass 2016	There was no reference to any methods around assessing adverse events or harms. Authors state, "We learned that it is possible to implement targeted strategies to include sexual violence survivors in VSLA groups in a safe and ethical manner, and that sexual violence survivors even those with severe trauma symptoms, can benefit from this participation."	—
Bell 2019	The study lists a range of stressors that participants were exposed to during the study but does not provide numbers for these. They make the point that "many participants who were exposed to triggers and stressors reported feeling lower levels of stress reactivity and enhanced levels of self‐regulation in response to these stressors than they had prior to the study." The study refers to how stressors may confound the findings and acknowledges triggering experiences occurring in participants' lives but does not give attention to harms arising from its interventions. Notwithstanding this, there was just 1 withdrawal, and that was in the control (or minimal intervention) group.	—
Bomyea 2015	"No participants experienced clinically significant deterioration." The authors acknowledge the high rate of treatment dropout but did not gather systematic data or reasons for this. Overall, the study does not report a systematic approach to recording adverse events and harms.	—
Bowland 2012	Treatment dropout was low: "One did not attend any groups and was unable to be reached after the randomization procedure". Overall, the study does not report a systematic approach to recording adverse events and harms.	—
Brady 2021	"Overall, retention in both arms of the trial was acceptable. Within the NET group, one individual discovered that she was in the advanced stages of pregnancy soon after allocation. It was not feasible for her to receive a full course of treatment, so she was withdrawn from the study. One individual dropped out of therapy after eight sessions, stating that they no longer wanted to engage in trauma‐focused therapy. All other participants in the NET group (n = 13) completed NET. In the wait‐list group, one participant was removed from the trial before the end of the waiting period owing to a significant increase in their risk of suicide. The remaining participants in the wait‐list group completed the waiting period. However, two participants dropped out of the study at the point of post‐wait assessment, owing to a deterioration in their physical health, and were therefore lost to follow‐up. The remaining participants in the wait‐list group were offered the opportunity to receive NET, and all took up this offer." The researchers make reference to deterioration in scores – for example, in the NET group, two people had worse scores on the CAPS‐5 – but this was not clinically significant, and we did not count it.	0/15 narrative exposure therapy vs 1/10 minimal intervention (psychoeducation)
Falsetti 2008	It is indicated that "Additionally, 16 participants dropped out of the study, including six individuals in the treatment group who attended only one session and 10 participants who dropped out at a later point in the study (two from the treatment group and eight from the control group)." However, reasons for treatment noncompletion are not furnished. There is no other reference to assessment of adverse events or harms in the study.	—
Foa 1991	No specific reference to adverse events or harm is made.	—
Foa 1999	The study provides an analysis of the differences in baseline characteristics between treatment completers and noncompleters but does not offer any explanations as to why people elected to exit the intervention. "More participants dropped out from SIT and PE‐SIT (27%) than from the PE and WL conditions (5%)… There were no significant differences between dropouts and completers on any of the pretreatment measures of psychopathology. A significant difference on one demographic variable emerged: nonworking participants (30%) were more likely to drop out than participants who were working full or part time (10%)." No specific reference to adverse events or harm is made.	—
Foa 2005	Greater detail in this subsequent study by Foa and colleagues on the nature of events: "Twelve serious adverse events led to termination in the study, six of which are included in the postrandomization removal category in Figure 1 (4 participants reassaulted, 1 developing a life‐threatening illness, and 1 death). The remaining six serious adverse events were classified as drop‐outs (4 had severe depression and suicidal ideation that required immediate intervention, 2 of which were hospitalized, and 2 exhibited extreme dissociative symptoms)." This study also examined symptom worsening. Whilst this study has provided detail at the individual level not seen in others, it is limited by not providing events by group and thus cannot be meta‐analysed.	12/179
Foa 2006	"Twenty‐four participants (26.7%) dropped out of the intervention, leaving 66 completers. Dropouts were distributed as follows: 9 (29%) in the B‐CBT condition, 10 (33.3%) in the AC, and 5 (17.2%) in the SC condition. The dropout rate did not differ across conditions…No significant differences between completers and dropouts emerged on pre‐intervention psychopathology and demographic variables." The authors state, "Finally, we did not collect systematic information about how many people were excluded from the study or the reasons why." Overall, this study lacks detail on adverse events and harms.	—
Gray 2020	Dropout from this study was low – one person exited the post‐wait intervention group. The study states that "No reportable adverse events occurred" across the 30 randomised participants.	0/15 RTM vs 0/15 waiting list
Krakow 2001	Four participants were lost from the intervention. The study states, "Imagery rehearsal therapy produces imagery adverse effects; 4 patients reported increased negative imagery and eventually withdrew, and 12 of 66 who completed treatment did not complete follow‐up for unknown reasons." Some limitations around the potential for wider harms to be detected.	4/88 CBT/IRT vs 0/80 waiting list
Littleton 2016	This study was explicit about any increases in symptoms (PTSD, anxiety and depression) at the individual level, e.g. "No participants reported a clinically significant increase in PTSD symptoms from pre‐treatment to follow‐up. Two participants in the interactive program reported continued clinically significant increases in depression symptoms from pre‐treatment to follow‐up…One participant assigned to the psycho‐educational website also experienced a clinically significant increase in depressive symptoms from pre‐treatment to follow‐up. Finally, one additional participant in the interactive program reported a clinically significant increase in anxiety at follow‐up." This study shows positive attention to individual journeys through the treatment; however, it confines its assessment to the outcome measures.	3/46 interactive CBT vs 1/41 minimal intervention website
Miller 2015	Only 1 person is reported to not have tolerated the psychoeducation video; however, attrition was very high in this study at nearly 60%. "Participants were questioned about whether the examination or participating in research increased their distress. Overall, they responded that neither increased distress so this is unlikely to relate to the attrition." There is a lack of information about the qualitative component of this research. There are many unknowns in regard to the reasons why attrition was so elevated.	—
Rajan 2020	Treatment completion was high, and attrition was low. "No harms were detected (i.e., there were no elevated scores on IES‐R, or NSESSS at time point two or three)." The authors explain, "Because this was the first randomized controlled treatment study conducted for the method, we followed the results in order to be able to detect adverse effects (i.e., elevated scores on self‐rating at time point two)." This study showed an awareness of monitoring adverse effects, although it appears to confine this to the outcome measures.	0/21 modified lifespan integration vs 0/17 waiting list
Resick 2002	This study makes some attempt to provide reasons for early postrandomisation withdrawals, though it is not systematic about this: "Of 181 women randomized into the trial, 10 were terminated from the study as a result of meeting exclusion criteria subsequent to new violence (women had to be at least 3 months posttrauma), changes in medication, or substance dependence relapse. Therefore, the intent‐to‐treat (ITT) sample included 171 women, among whom 13 never returned for the first session." The study provides an analysis on the differences in baseline characteristics between treatment completers and noncompleters but does not offer any explanations as to why a further 37 women left the treatment", "Thirty‐seven women dropped out of treatment, and 121 women completed treatment along with at least the posttreatment assessment: 41 CPT clients, 40 PE clients, and 40 MA clients. Dropout rates for the two active treatment groups were similar: 26.8% for CPT and 27.3% for PE. In the MA condition, 14.9% did not return for the second assessment. There were no significant differences between women who dropped out of therapy and those who completed therapy with regard to their initial PTSD or depression scores." This study lacks a systematic approach to recording adverse events and harms.	—
Rothbaum 1997	No specific statement on adverse events or harm is offered; however, reasons for attrition of 3 people in the study are provided, and none raise concerns.	—
Rothbaum 2005	"Of the 74 women enrolled in the study, 1 dropped out during the assessment phase, 1 was terminated and referred during treatment for not meeting treatment criteria, 12 dropped out during treatment, and 60 women (83.3%) completed the protocol. The dropout rate across the three groups was not significantly different, PE: 13.0% (n = 3, 2 before MID); EMDR: 20.0% (n = 5, 4 before MID); and WAIT: 16.7% (n = 4)." This study lacks a systematic approach to recording adverse events and harms.	—
Sikkema 2018	This study highlights the barriers to gaining reasons for treatment noncompletion when people are not contactable: of the 13 who left the treatment, 12 were not reachable and 1 had scheduling issues; of the 6 who exited the standard care condition, 4 were not reachable and 2 had scheduling issues. In terms of attrition, "Participants lost to follow‐up at the 6‐month assessment were significantly more likely than those retained to have reported hazardous drinking (69.2% vs. 37.3%, p = 0.04) and recent physical intimate partner violence at baseline (46.2% vs. 17.65%, p = 0.03)." This study lacks a systematic approach to recording adverse events and harms.	—
Walsh 2017	"The PANAS was administered pre‐exam as a measure of potential differences in distress across groups as well as post‐exam as a validity check regarding intervention condition." We did not see any reporting of scores beyond as a check when a change in length of the video was introduced. This study lacks a systematic approach to recording adverse events and harms.	—

AC: assessment control; B‐CBT: brief cognitive behaviour therapy; CAED: Clinician‐Assisted Emotional Disclosure; CAPS‐5: Clinician‐Administered PTSD Scale for DSM‐5; CBT: cognitive behavioural therapy; CPT: cognitive processing therapy; EMDR: eye movement desensitisation and reprocessing; IES‐R: Impact of Event Scale – Revised; IRT: imagery rehearsal therapy; MA: minimal attention condition; MID: minimally important difference; NET: narrative exposure therapy; NSESSS: National Stressful Events Survey PTSD Short Scale; PANAS: Positive and Negative Affect Schedule; PE: prolonged exposure; PE‐SIT: prolonged exposure combined with stress inoculation therapy; PTSD: post‐traumatic stress disorder; RTM: reconsolidation of traumatic memories; SC: supportive counselling; SIT: stress inoculation therapy; VSLA: Village Savings and Loans Associations; WAIT: wait‐list control; WL: wait‐list

##### Secondary outcomes

###### Anxiety symptoms

A total of 10 studies (436 participants) reported a measure of anxiety symptoms post‐treatment (Bell 2019; Bomyea 2015; Bowland 2012; Brady 2021; Foa 1999; Foa 2006; Littleton 2016; Miller 2015; Rothbaum 1997; Rothbaum 2005).

We entered all data for anxiety symptoms in a random‐effects meta‐analysis, which showed a SMD of −0.84 at post‐treatment (95% CI –1.26 to −0.42, P < 0.001, I^2^ = 73%; 10 studies, 436 participants; Analysis 1.7). This showed a large effect size favouring the experimental group. For the three‐month time point, we pooled four studies and revealed a SMD of ‐0.26 (95% CI −0.44 to –0.07; P = 0.007, I^2^ = 0%; 449 participants; Analysis 1.8); this was a small effect favouring the experimental group. An insufficient number of studies reported anxiety symptoms at 6 and 12 months. We observed a tendency towards symmetrical funnel plots in Figure 4, disregarding possible reporting and publication biases.

**4:**
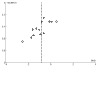
Funnel plot for the comparison of psychosocial interventions versus inactive control for anxiety symptoms at post‐treatment

###### Global mental health functioning/distress

Three studies reported global mental health/distress symptoms (Anderson 2010; Rajan 2020; Rothbaum 1997). Post‐treatment, a pooled meta‐analysis revealed a SMD of −0.92 (95% CI −1.70 to −0.13; P = 0.02, I^2^ = 61%; 3 studies, 80 participants; Analysis 1.9). We interpreted the SMD as a large effect favouring the experimental condition. There were insufficient studies to pool for the other time points.

###### Trauma‐related beliefs, substance misuse, dissociation and quality of life

An insufficient number of studies reported on these outcomes at post‐treatment, three months, six months and 12 months to conduct analyses.

##### Subgroup analyses

Where data permitted and sufficient studies were available to pool, we conducted our planned subgroup analyses.

###### PTSD symptoms

The test for subgroup differences found evidence of a difference between different types of psychosocial interventions (Analysis 1.1, test for subgroup differences: Chi² = 27.45, df = 2 (P < 0.001, I² = 92.7%)). This analysis suggests that while both CBT (SMD –0.77, 95% CI –1.12 to –0.42; 6 studies, 575 participants) and Behavioural Therapy (SMD –1.85, 95% CI −3.00 to –0.70; 7 studies, 233 participants) may result in a reduction in PTSD symptoms compared to inactive control, there may be no evidence of a difference between psychosocial (low‐intensity) interventions and inactive controls (SMD 0.26, 95% CI −0.04 to 0.55; 4 studies, 322 participants).

###### Depressive symptoms

The test for subgroup differences found no evidence of a difference between different types of psychosocial interventions for this outcome (Analysis 1.3, test for subgroup differences: Chi² = 3.00, df = 2 (P = 0.22), I² = 33.4%). This analysis suggests that while both CBT (SMD –0.73, 95% CI –1.13 to –0.33; 7 studies, 595 participants) and Behavioural Therapy (SMD –1.51, 95% CI ‐2.58 to –0.44; 4 studies, 123 participants) may result in a reduction in depressive symptoms compared to inactive control, there may be no evidence of a difference between psychosocial (low‐intensity) interventions and inactive control (−0.34, 95% CI −1.12 to 0.44; 2 studies, 183 participants).

#### Comparison 2. Trauma‐focused versus non‐trauma‐focused interventions

##### Primary outcomes

###### PTSD symptoms

We pooled studies in a random‐effects meta‐analysis to reveal a SMD at post‐treatment (SMD −0.18, 95% CI −0.48 to 0.13; P = 0.26, I^2^ = 67%; 10 studies, 727 participants; Analysis 2.1), suggesting there may be no group difference in PTSD symptoms at post‐treatment. Interpreting the result at three months using Cohen's D, there was evidence of a small effect favouring trauma‐focused interventions, with a SMD of −0.33 (95% CI −0.49 to –0.16; P < 0.001, I^2^ = 30%; 8 studies, 584 participants; Analysis 2.2). There may be no effect at six months (SMD −0.21, 95% CI −0.43 to 0.01; P = 0.06, I^2^ = 26%; 5 studies, 533 participants; Analysis 2.3). Fewer than three studies were available for pooling at 12 months.

###### Depressive symptoms

Pooling of studies for depressive symptoms at post‐treatment revealed a SMD of −0.21 (95% CI −0.54 to 0.12; P = 0.21, I^2^ = 69%; 9 studies, 673 participants; Analysis 2.4), suggesting there may be no difference between groups at post‐treatment. At three months, a SMD of −0.56 was revealed (95% CI −0.97 to –0.15; P = 0.008, I^2^ = 72%; 7 studies, 535 participants; Analysis 2.5), suggesting there may be a moderate effect favouring trauma‐focused interventions. At six months, the SMD was ‐0.68 (95% CI −1.49 to 0.13; P = 0.10, I^2^ = 94%; 5 studies, 532 participants; Analysis 2.6), suggesting there may be no group difference at six months. An insufficient number of studies was available for pooling at 12 months.

###### Dropout from treatment

We pooled 10 studies (859 participants) in a random‐effects meta‐analysis to reveal a risk ratio of 1.43 (95% CI 1.08 to 1.87; P = 0.01, I^2^ = 18%; Analysis 2.7). The results favoured non‐trauma‐focused interventions; those exposed to trauma‐focused interventions may have an increased risk of dropping out from treatment.

###### Adverse events

We pooled five studies (591 participants) and revealed a risk ratio of 0.63 (95% CI 0.29 to 1.37; P = 0.24, I^2^ = 4%; Analysis 2.8), suggesting there may be no group difference in adverse events. Table 6 outlines additional data reported by the included studies.

**5:** List of included studies in Comparison 2 reporting adverse events data

**Study**	**Approach to reporting adverse events**	**Count**
Covers 2021	"Only one participant discontinued participation due to increased suicide ideation. No other (serious) adverse events were found."	1/29 behavioural (EMDR) vs 0/28 other psychosocial interventions (telephonic psychosocial support)
Feske 2008	"Two PE clients and 1 TAU client were withdrawn because they changed their medication. Two PE clients dropped out for unknown reasons; one TAU client dropped out because her depression worsened after the third TAU session."	0/13 CBT/PE and 1/14 integrative (TAU) (non‐trauma focused)
Foa 1991	No specific reference to adverse events or harm is made.	—
Foa 1999	No specific reference to adverse events or harm is made.	—
Katz 2014	"The study was monitored by the local IRB and no adverse events were reported."	0/17 PE vs 0/17 integrative/holographic reprocessing (non‐trauma focused) vs 0/17 PCT (non‐trauma focused)
Kelly 2021	"There were no safety problems or unanticipated adverse events reported during this study. There were no study‐related physical injuries. Two participants in the CPT group withdrew due to increased psychological distress and were referred for individual therapy. The study included a Data Safety Monitoring Board (DSMB) in the final year with the addition of the second site. The DSMB met with the study Principal Investigator and had no concerns regarding safety of participants or data."	2/46 CBT/CPT vs 0/59 trauma‐sensitive yoga (non‐trauma focused)
Nixon 2016	"There were no significant adverse events in either condition." Based on available data and using a change of > 12 points on the CAPS as an indicator of reliable change, 2 participants in CPT demonstrated worsening of symptoms at different periods. Two participants in the group that received integrative care (as part of TAU) had an increase of exactly 12 points on the CAPS at 6‐month follow‐up. This worsening of symptoms was not seen on participants' PCL scores.	2/25 CPT vs 2/22 integrative (non‐trauma focused)
Resick 2008a	"Of 162 women randomized into the trial, 12 were terminated from the study, by design, for meeting exclusion criteria subsequent to new violence (women had to be at least 3 months posttrauma), changes in medication, or psychosis. Among them, 1 WA participant was terminated from the trial when the therapist stopped the protocol because of increased suicidal ideation. These terminations were evenly distributed across groups. Therefore, the intent‐to‐treat (ITT) sample included 150 women. There was one other unrelated adverse event during the trial." We had already excluded the WE group from Comparison 2, and it is not clear which group the other person with an adverse event was assigned to.	—
Schnurr 2007	"There were 5 serious adverse events in prolonged exposure (4 psychiatric hospitalizations and 1 suicide attempt) and 14 in present‐centered therapy (2 deaths [nonsuicidal], 9 psychiatric hospitalizations, and 3 suicide attempts). No events were regarded as study‐related; the suicide attempt in prolonged exposure was coded as possibly related."	5/141 CBT/PE vs 14/143 PCT (non‐trauma focused)
Surís 2013	"During the course of the study there were three adverse events in the CPT condition (one suicide attempt by overdose and two psychiatric hospitalizations) and two adverse events in the PCT condition (one suicide attempt by overdose and one psychiatric hospitalization). No events were deemed definitely study‐related; however, one psychiatric hospitalization in the CPT condition was deemed possibly related."	3/72 CBT/CPT vs 2/57 PCT (non‐trauma focused)

CAPS: Clinician Administered PTSD Scale; CBT: cognitive behavioural therapy; CPT: cognitive processing therapy; EMDR: eye movement desensitisation and reprocessing; IRB: institutional review board; PCL: Post‐Traumatic Stress Disorder Checklist; PCT: present‐centred therapy; PE: prolonged exposure; TAU: treatment as usual; WA: written account; WE: written exposure

##### Secondary outcomes

###### Anxiety symptoms

We pooled seven studies for the post‐treatment time point comparing trauma‐focused and non‐trauma‐focused interventions. This meta‐analysis revealed a SMD of –0.15 (95% CI −0.42 to 0.13; P = 0.29, I^2^ = 38%; 7 studies, 540 participants; Analysis 2.9), suggesting a lack of evidence for a group difference in anxiety. The three‐month and six‐month time points also revealed SMDs (three months: SMD −0.23, 95% CI −0.49 to 0.02; P = 0.07, I^2^ = 11%; 5 studies, 403 participants; Analysis 2.10; six months: SMD −0.05, 95% CI −0.37 to 0.28; P = 0.79, I^2^ = 47%; 3 studies, 399 participants; Analysis 2.11), suggesting there may be no group differences in anxiety at three and six months.

###### Trauma‐related beliefs

At post‐treatment, a SMD of 0.16 was revealed after pooling three studies (95% CI −0.17 to 0.50; 152 participants; P = 0.34, I^2^ = 0%; Analysis 2.12), suggesting there may be a lack of evidence for a group difference in trauma‐related beliefs. There was an insufficient number of studies for pooling at three, six and 12 months.

###### Global mental health functioning/distress

Three studies reported global mental health/distress at post‐treatment. This meta‐analysis revealed a SMD of 0.07 (95% CI ‐0.36 to 0.51; P = 0.74, I^2^ = 35%; 3 studies, 342 participants; Analysis 2.13), and at three months we identified a SMD of ‐0.35 (95% CI ‐1.00 to 0.31; P = 0.30, I^2^ = 73%; 3 studies, 341 participants; Analysis 2.14). This suggests that there may be no group difference in global mental health or distress.

## Discussion

### Summary of main results

We identified 36 studies (1991 to 2021) that were randomised controlled trials of psychosocial interventions (ranging from intensive psychotherapy to low‐intensity, social, psychoeducation and other interventions) following rape, sexual assault and sexual abuse. These studies included 3992 people with exposure to trauma, assigned to 60 experimental groups (3014; 76%) and 23 inactive comparator conditions (978, 24%). Eighty‐two per cent of people had sexual trauma during adulthood; 94% had a post‐traumatic stress disorder (PTSD) diagnosis at baseline based on a clinical interview or clinically important symptoms based on self‐report thresholds; 99% of participants were women and 60% identified as Black or as an ethnic minority.

#### Comparison 1. Psychosocial interventions versus controls

Taken together, and when compared to control conditions, the evidence suggests that psychosocial interventions may reduce PTSD and depression symptoms at post‐treatment (in the days and weeks following intervention). Overall, there was a low level of certainty, mainly due to heterogeneity in the studies (for example, psychosocial interventions vary considerably in their aims and mechanisms) and risk of bias. Psychosocial interventions may not decrease treatment acceptability (i.e. completion of treatment) relative to comparison groups; however, this outcome was based on a small number of studies and there was low certainty in the evidence due to not fully addressing the question about treatment acceptability. Our fourth outcome in our main comparison was adverse events/effects, with 21 events identified in seven studies. We had a very low level of certainty about this outcome; there was high non‐completion across groups and attrition from studies without explanation, suggesting potential for missing wider harms of exposure to the intervention or comparison conditions and/or research participation.

Beyond post‐treatment, there may be no difference between groups in PTSD and depressive symptoms at three months. However, studies included at that time point were relatively low‐intensity interventions including: an economic scheme (Bass 2016); brief (Foa 2006) and online (Littleton 2016) cognitive behavioural therapy (CBT); a psychoeducation video (Miller 2015); and an intervention primarily to improve AIDS care post‐sexual trauma (Sikkema 2018). Establishing benefits beyond three months was not possible due to a lack of control data ‐ many wait‐list groups were offered active interventions after the post‐treatment assessment, excluding the option of longer‐term comparison.

##### Secondary outcomes

The evidence suggests that there may be large effects for post‐treatment anxiety and global mental health/distress favouring psychosocial interventions among survivors of sexual violence and abuse. At three months, a small effect persisted for anxiety. An insufficient number of studies reported global mental health/distress beyond post‐treatment, and for trauma‐related beliefs, dissociation and substance misuse at any time point, to conduct analyses.

##### Subgroup of comparison 1: analysis of three types of psychosocial intervention

i) The CBT subgroup combined common approaches such as Prolonged Exposure and Cognitive Processing Therapy, with CBT blends (Foa 2005) and alternatives like Multiple Channel Exposure Therapy (Falsetti 2008), Imagery Rehearsal Therapy (Krakow 2001) and Clinician‐Assisted Emotional Disclosure (Anderson 2010) versus inactive controls.

ii) The behavioural interventions subgroup included a mixture of traditional EMDR studies (Rothbaum 1997; Rothbaum 2005) and Narrative Exposure Therapy (Brady 2021) and new approaches such as Reconsolidation of Traumatic Memories (Gray 2020), High Interference Control (Bomyea 2015), neurofeedback (Bell 2019) and Lifespan Integration (Rajan 2020), and a pleasant imagery and relaxation instruction video provided at an acute medical setting (Walsh 2017) versus inactive controls.

iii) Psychosocial interventions of low intensity combined low‐intensity (largely non‐psychotherapeutic) interventions grouped as 'other psychosocial interventions' and 'other psychologically oriented interventions' versus inactive controls. Relative to CBT and behavioural interventions, these interventions tended to be less manualised and did not require the same level of training for staff, which was an important part of making them accessible in acute and primary care healthcare and in low‐ and middle‐income settings. They included brief video interventions delivered in acute settings (Miller 2015; Walsh 2017) and interventions that prioritised other health or social issues such as care for HIV/AIDS (Sikkema 2018), economic difficulties (Bass 2016) or used computerised screening and responses for multiple issues, e.g. substance misuse and intimate partner violence (Creech 2021).

For post‐treatment PTSD, the test for subgroup differences found evidence of a difference between different types of psychosocial interventions and suggested that both CBT and behavioural interventions may result in a reduction in PTSD symptoms compared to inactive controls. However, there may be no evidence of a difference between psychosocial (low‐intensity) interventions and inactive controls. The evidence was less conclusive for depressive symptoms at post‐treatment, though it similarly suggested that CBT and behavioural interventions, but not low‐intensity psychosocial interventions, may result in reductions in depression.

#### Comparison 2. Trauma‐focused interventions compared to non‐trauma‐focused interventions

The next comparison was a head‐to‐head analysis of active interventions, addressing a key question concerning whether interventions that involved exposure to the feared memory of the traumatic event (or to cues that became associated with fear at the time of a traumatic event) would outperform interventions that did not include such exposure. The evidence suggests that trauma‐focused interventions may result in little to no difference in PTSD symptoms and depressive symptoms at post‐treatment compared to non‐trauma‐focused interventions. Further, there may be no group difference in adverse events. However, participants who underwent trauma‐focused therapies had increased risk of not completing the treatment compared to those who received interventions that did not involve confronting feared memories and stimuli such as Holographic Reprocessing (Katz 2014), Stress Inoculation Therapy (Foa 1999), Present‐Centred Therapy (Schnurr 2007; Surís 2013) and telephonic psychosocial support (Covers 2021), along with a range of other novel and emerging non‐trauma‐focused interventions (e.g. trauma‐sensitive yoga; Kelly 2021). Studies generally matched the intensity of the interventions (i.e. number and duration of therapy sessions in each group).

All interventions in both groups reported changes at post‐treatment from baseline of 1 to 2 standard deviations, which is within the recommendations for establishing clinically meaningful changes (e.g. based on the clinician‐assessed PTSD scale (CAPS) and self‐report PTSD checklist (PCL) severity and change z scores ranging between 0.5 to 0.8 standard deviations in Stefanovics 2018).

At three months, trauma‐focused interventions may result in a small important effect, with a slight reduction in PTSD, and a moderate effect for depressive symptoms. There may be no group differences in these primary outcomes at six months and there were insufficient studies to pool at 12 months. There was a lack of evidence on group differences for the secondary outcomes.

The improvement in mental health at three months favouring trauma‐focused intervention needs to be balanced against treatment acceptability. It suggests that among those who are safe and no longer exposed to the trauma, have achieved stabilisation and express a preference for such approaches, offering treatment with a trauma focus may be appropriate. For others, and those that opt to exit trauma‐focused treatments early, structured non‐trauma‐focused approaches provide a viable alternative.

### Overall completeness and applicability of evidence

Our review considers randomised controlled trials of a wide range of psychosocial interventions for survivors of sexual violence and abuse. These included TF‐CBT, EMDR and other behavioural approaches, non‐trauma‐focused CBT and other psychotherapeutic and psychosocial interventions, using mainly individual, face‐to‐face delivery (see Included studies). Studies included participants from different countries and backgrounds, who had been exposed to sexual violence and abuse across a range of contexts and circumstances, reflected in the approach to recruitment and settings of intervention delivery (see Included studies). The majority of studies reported on the use of qualified and experienced therapists, and an encouraging proportion included assessment of adherence to a treatment protocol (see Included studies table).

Starting with country of origin, trials of interventions for PTSD generally (Bisson 2013) and for specific populations such as military‐related trauma survivors (Steenkamp 2015) and sexual violence and abuse survivors, derive mainly from the USA (72% of studies in this review). Interventions tested in the USA tend to be trauma‐exposure based, have strong links with responses to military‐based trauma and involved clinical settings and treatment‐seeking populations. This contrasts with the four studies (12%) that came from the African continent (South Africa and the Democratic Republic of the Congo (DRC)), notably more 'psychosocial' than psychotherapeutic, with a primary focus on promoting HIV care (Sikkema 2018), post‐exposure prophylaxis after rape (Abrahams 2010) and economic factors (Bass 2016); the one study that did consist of Cognitive Processing Therapy, Bass 2013, omitted the trauma narrative in a bid to enhance feasibility and reach. These studies highlighted co‐morbidities and other major health concerns for rape, sexual assault and sexual abuse. All included efforts to use culturally adapted measures, local‐language versions of well‐established measures, or translated and adapted interventions to meet the needs of the local women. No studies in the current review arose from Asia although one UK study of migrant people seeking care after exposure to trafficking did include people from parts of Asia (Brady 2021). Three additional studies derived from Europe ‐ Sweden (Rajan 2020), the Netherlands (Covers 2021) and Spain (Echeburua 1996). The remaining two studies were from Australia (Nixon 2016) and Canada (Belleville 2018). To summarise, there were 32 studies from high‐income countries, two from middle‐income countries and two from low‐income countries.

Studies were more balanced on ethnicity than country; over 60% of participants had ethnicities other than White/Caucasian, demonstrating an important shift away from ethnocentrism common to this field of enquiry (IOM 2008). Just two studies focused specifically on recruiting a minority sample: low‐income, African‐American women (Feske 2008) and international survivors of people trafficking (Brady 2021). Evidence from our review, however, is strongly weighted towards the experiences of women, with just four studies engaging men (making up only 1% of the total randomised sample). Whilst the global burden of sexual violence and abuse is shouldered by women and girls, men's experiences in this sphere remain under‐represented. Related to this is the lack of emphasis on wider aspects of gender and sexual identities: with few exceptions (Creech 2021), transgender and non‐binary people's experiences were omitted, as was reference to sexual identity or orientation in participant characteristics across studies.

There were generally no upper limits on age across studies: mean ages ranged from 19 years among a university sample (Anderson 2010) to 61 years in a study that specifically recruited older women (Bomyea 2015), with a weighted mean age of 35.9 years across all participants. Despite the levels of sexual violence and abuse experienced by migrant and refugee populations, these groups were under‐represented in this evidence, the recent UK study being an exception (Brady 2021). This study attempted to overcome barriers of language by engaging interpreters in the research. Similarly, those with learning and communication disabilities, people affected by substance misuse, suicidality and severe mental health difficulties, and those currently at risk of domestic abuse were routinely excluded. Whilst the rationale for some of these exclusions is understood in the context of conducting a RCT (e.g. safety and for establishing mechanisms of change), it is important to point out the gaps in this evidence as regards who it does and does not apply to and the risk of excluding individuals whose complex needs make treatment more difficult (Bisson 2013). In particular, we draw attention to the exclusion of individuals with more complex trauma histories in intervention trials. Complex PTSD (CPTSD; ICD‐11; WHO 2021) can arise for individuals who have experienced chronic, repeated and prolonged traumas, such as childhood sexual abuse or domestic abuse. CPTSD is associated with the experience of complex reactions extending beyond those typically observed in PTSD in three key domains: emotion regulation, self‐identity and relational capacities (WHO 2021).

To enhance the overall completeness of the evidence base, future research needs to be cautious about what subgroups of survivors are excluded from trials. Recognising the challenges related to involving survivors of rape, sexual assault and sexual abuse in research, we are cautious to recommend that studies need to solely focus on minority groups as this may not be feasible. However, the evidence could be enhanced by taking steps to report results for different subgroups to allow meta‐analyses related to participant characteristics.

### Quality of the evidence

We judged certainty of the evidence for all four primary outcomes using GRADE. Heterogeneity was one of the main problems in the main comparison but it was not unanticipated given that we drew such a wide range of interventions together in one analysis. In any case, since we detected substantial heterogeneity (Deeks 2021) for PTSD and depression, we downgraded on this basis; dropout (treatment acceptability) and adverse events/effects were not affected to the same extent and therefore we did not downgrade these for inconsistency. Given the wide range of intervention types, we carried out a sensitivity analysis to exclude low‐intensity psychosocial interventions. Whilst this led to increases in effect sizes, it did not improve heterogeneity significantly. This suggests the heterogeneity may have arisen from additional methodological and clinical characteristics, such as sample sizes and time since trauma.

The threat of bias was another reason for downgrading the evidence. We observed i) poor reporting or execution of the randomisation process and ii) the potential for systematic bias relating to missing outcome data and the associated risk that attrition from studies was associated with trauma burden, thus affecting primary outcomes for mental health but also adverse events. We additionally observed instances of including treatment completers only in analyses and in summaries of baseline characteristics. However, intention‐to‐treat analyses were commonplace in the more recently published reports. Some problems such as lack of blinding to the intervention were more enduring but the RoB 2 tool does not penalise study results if it can be demonstrated that there is a low likelihood that the lack of blinding of personnel and participants influenced the outcome in the groups. We had concerns that adverse events may suffer a reporting bias as it was addressed by so few studies overall. Sensitivity analyses that excluded high‐risk results showed a reduced PTSD and depression effect size, from high to medium.

We also had concerns that treatment acceptability (dropout) may suffer from indirectness, not quite addressing a key question about whether psychosocial interventions are associated with higher treatment non‐completion than other conditions. Dropout also suffered from a high level of imprecision. Similarly, the adverse events/effects outcome was affected by imprecision due to very few events being reported/included in the analysis, and there was inconsistency in the approach to measuring this outcome. Thus, we downgraded these outcomes twice.

Although not formally reported using GRADE, the second comparison of trauma‐focused interventions compared to non‐trauma‐focused interventions provided a much more robust picture about treatment acceptability and adverse events. It addresses whether or not available research evidence can be directly used to answer the question about whether interventions for sexual violence and abuse lead to harm and non‐completion. Risk of bias was less frequent in the more recent studies and studies in the head‐to‐head comparison were more recent; further, the evidence suffered neither inconsistency (reporting I^2^ mainly in a range unlikely to be important (Deeks 2021)) nor imprecision (e.g. narrow confidence intervals). Thus, although not included in our summary of findings table, Comparison 2 delivered a promising level of certainty about the evidence.

### Potential biases in the review process

We believe the various strategies described in the Methods section will have effectively minimised the potential biases in the review process. The most important among them has been the engagement of authors from several different institutions across aspects of the review and consultation with people with lived experience of sexual violence or abuse. We have also engaged widely with authors of included studies to try and gather missing/subgroup data and gain input on categorising novel interventions. We have indicated where we successfully accessed data from authors in the Included studies table. On rare occasions, we were unable to access disaggregated data to check if a study population contained a sufficient proportion of sexual violence survivors to be included in our review. There are also many RCTs of interventions targeting domestic abuse survivors where participants will have been subjected to rape, sexual assault and sexual abuse by partners or ex‐partners that could be relevant to our review both in terms of population and intervention. However, we took the decision to exclude domestic abuse interventions due to another Cochrane Review (Hameed 2020). There are many elements of the review that rely on authors' judgement, including interpretation of the certainty of the evidence, so it is possible that a different review team may not have agreed with all our assessments or decisions.

### Agreements and disagreements with other studies or reviews

Several previous reviews of interventions for PTSD have included sexual violence and abuse survivors as a subset of the overall trauma population (Hetrick 2010; Kitchiner 2019; Roberts 2015; Roberts 2016; Steenkamp 2015), and several have called for a focus on specific populations (Bisson 2013; Regehr 2013). Our review has succeeded in increasing the representation of survivors of adulthood sexual violence and abuse to 81% of the total number of people randomised.

A Cochrane Review of psychological interventions for chronic PTSD in any population underscored the need for reviews of trauma interventions within specific populations given the degree of clinical and methodological diversity observed across studies (Bisson 2013). That review included 13 studies of sexual violence and abuse survivors, of which three compared TF‐CBT to other therapies and identified no differences in PTSD at follow‐up. Many relevant studies have been added to the literature since 2013; we incorporated 10 studies of TF‐CBT compared to other therapies, and noted clinically relevant improvements for both TF‐CBT and non‐exposure treatments at post‐treatment, and a small effect favouring the trauma‐exposed group at three months. We also identified increased likelihood of dropout in the TF‐CBT group. These findings are consistent with the Steenkamp 2015 review, which found that TF‐CBT (CPT and Prolonged Exposure) was marginally superior to non‐trauma focused psychotherapies among military personnel and veterans experiencing PTSD (Steenkamp 2015). Our review diverges from previous findings in respect of EMDR (Kitchiner 2019), where no benefit was detected for reducing PTSD in active duty and ex‐serving military personnel with PTSD. This is consistent with guidance that recommends EMDR as an option for sexual violence and abuse survivors, but not for military‐related trauma (e.g. NICE 2018).

Our review supports and strengthens findings from another review involving adult sexual violence and abuse survivors (Regehr 2013). Based on six studies, Regehr 2013 reported that cognitive and behavioural interventions had “a statistically significant effect” on PTSD and depressive symptoms in comparison to the control groups.

A contribution of the current review is the synthesis of several novel treatments across a range of areas including Lifespan Integration (LI), neurofeedback, Reconsolidation of Traumatic Memories (RTM) and trauma‐sensitive yoga. Some of these interventions have been shown to be effective among survivors of a range of types of trauma not including sexual abuse and violence experienced as an adult (e.g. see Gray 2019 for a study of RTM; Panish 2020 for a review of neurofeedback), whilst the others have very little robust evidence in any population, with research limited by methodological problems such as lack of control groups or randomisation (Kelly 2021; Panish 2020). We identified four studies, one for each type of novel intervention discussed. Studies examining the effectiveness of LI (Rajan 2020), neurofeedback (Bell 2019) and RTM (Gray 2020) were included in our main comparison (i.e. compared to inactive controls). A study of trauma‐sensitive yoga was included in Comparison 2 (Kelly 2021), a head‐to‐head comparison of active treatments, which performed similarly to the gold standard treatment, CPT. Our review provides further support for the promise of these interventions, some of which (LI and RTM) are shorter than traditional approaches such as CPT and PE, with implications for the reduction of treatment dropout and more effective use of limited available resources. However, we also support the conclusions of the authors of the studies (Bell 2019; Gray 2020; Kelly 2021; Rajan 2020) and review (Panish 2020) that further RCTs are required to more firmly establish the evidence base for these more recently developed interventions.

Taken together, these finding indicate the value of our review in terms of filling a gap in relation to the specific needs of sexual violence and abuse survivors and increasing overall certainty of the evidence about what works in this context.

## Authors' conclusions

**Implications for practice**

This synthesis of findings from 36 studies represents the most comprehensive analysis to date on the efficacy of psychotherapies and other psychosocial interventions for survivors of sexual violence and abuse in adulthood.

Our review suggests adult survivors of rape, sexual violence and sexual abuse may experience a large reduction in post‐traumatic stress disorder (PTSD) symptoms in the days and weeks following psychosocial interventions compared to controls. Post‐treatment reduction in PTSD exceeded an effect size criterion for clinical significance (standardised mean difference (SMD) ≥ 0.8) suggested by Kitchiner 2019. Psychosocial interventions may also reduce depressive symptoms. The evidence suggests that psychosocial interventions may not reduce treatment completion or increase adverse events when compared to controls. However, since treatment non‐completion and study attrition were high in both groups, the potential wider harms of exposure to different interventions and/or research participation itself may be missed. The estimates of effect may be biased because of poor randomisation processes and dropout from studies. There was also much variation in the studies (design, sample sizes and, importantly, the aims/nature of interventions). An observational test for subgroup differences found evidence of a difference between types of psychosocial interventions: cognitive behavioural therapy (CBT) and behavioural interventions may result in reductions in PTSD and depression, whilst psychosocial interventions of low intensity may result in little or no difference in mental health burden.

Based on our second comparison, the current review suggests that CBT with a trauma focus such as Prolonged Exposure and Cognitive Processing Therapy and other exposure‐based therapies (e.g. eye movement desensitisation and reprocessing (EMDR)) probably benefit survivors of sexual violence and abuse. However, trauma‐focused interventions may result in higher treatment non‐completion and may leave some survivors with a high symptom load post‐treatment. For example, despite achieving minimal clinically important differences (Stefanovics 2018) over inactive controls at post‐treatment and over other active interventions at three months, endpoint group means reported in several studies still exceeded thresholds for probable PTSD, e.g. 50 on the Clinician‐Administered PTSD Scale (CAPS) and 20 on the PTSD Scale‐Interview (PSS‐I) (Steenkamp 2015); 34 on the Post‐Traumatic Stress Disorder Checklist for DSM‐5 (PCL‐5) and 46 on Impact of Events Scale‐Revised (IES‐R) (Murphy 2017).

In response to the limits of trauma‐focused interventions, there have been calls for more effective approaches to the management of PTSD (Bisson 2013; Kitchiner 2019; Steenkamp 2015), and for sexual violence and abuse exposure specifically (Regehr 2013). One contribution of the current review is the synthesis of several novel treatments across a range of promising new areas such as Reconsolidation of Traumatic Memories (RTM); trauma‐sensitive or trauma‐informed yoga; Lifespan Integration (LI); and neurofeedback. Some of these have been shown to be effective among survivors of other types of trauma (e.g. RTM; Gray 2019), whilst others have little evidence in any population.

Our review consolidates evidence on mainstay treatments for PTSD, supporting the continued use of trauma‐focused psychotherapies such as EMDR, Cognitive Processing Therapy and Prolonged Exposure as first‐line treatments for PTSD (NICE 2018; VA/DoD 2017). It extends the evidence base by finding these PTSD treatments to be applicable to survivors of sexual violence and abuse. It acknowledges the limitations associated with trauma‐focused (exposure‐based) approaches. Non‐exposure‐based approaches, including several emerging areas, may offer opportunities for second‐line options to practitioners and service users. These variously include features of shorter duration; minimal or no focus on details of the trauma or associated cognitive and emotional effects; computer‐based or minimal interpersonal contact with a therapist; and somatic practice/movement as the main modality. Our analyses suggest that interventions do not necessarily need to involve a large number of sessions to be effective; however, they do require well‐trained, qualified therapists delivering interventions based on manualised, standardised treatment protocols. Low‐intensity psychosocial interventions (e.g. psychoeducation alone/videos; community interventions where the emphasis on sexual violence and abuse was secondary to other social or health concerns) may not reduce PTSD and depression, but that conclusion is based on low‐certainty evidence. Further, there are other important outcomes not measured in this review that may arise from such interventions; these include perceived support, advocacy, access to health services and legal advice.

It is the responsibility of practitioners and therapists to make decisions about treatments appropriate to the circumstances of their clients, in consultation with them and their families, carers or guardians. Further insight into factors that shape the treatment preferences of survivors, their families and professionals can be found in our related Cochrane Review (Brown 2020). The need to distinguish between PTSD and complex PTSD (CPTSD) has been argued since Judith Herman first proposed the diagnoses in the 1980s, with CPTSD added to the International Classification of Diseases 11th revision (ICD‐11) (WHO 2021). Recognising the distinction when considering suitable trauma treatment is important, as CPTSD may be less amenable to trauma‐focused approaches (Bradley 2005; Gray 2020). It underscores the value of a wide range of treatment options and alternatives to first line therapies. Many such alternative treatments could have relevance to clinical and policy decisions because they are often shorter and therefore less costly, easier to deliver, may be deliverable online, more feasible for survivors to access and scalable.

**Implications for research**

Some participants in the included studies had PTSD scores above a clinical threshold following treatment; low‐intensity psychosocial interventions may not have reduced mental health problems; and a third of those receiving exposure‐based interventions did not complete treatment. Thus, there is a need for further evaluation of new interventions to improve mental health in this population. We propose that future research includes high‐quality trials with regard to these intervention types and other novel approaches such that, in the future, survivors can be offered a range of evidence‐based interventions in line with their varying needs and preferences. Such interventions can benefit from comparisons with inactive (wait‐lists, no‐treatment control) conditions, as well as head‐to‐head comparisons, with the gold standard trauma‐focused treatments, or suitable non‐trauma‐focused comparators, such as Present‐Centred Therapy. Comparison with active standard care (for example, in Nixon 2016) is also feasible, though researchers need to be cautious about how they bring together different comparators because usual care in a specialist sexual assault setting will look very different to usual care in health settings (e.g. mental health services or primary care).

Many of the contemporary intervention types did not map easily to the psychological therapies listed by former Cochrane groups CCDAN and CCMD based on our experience and feedback from experts/researchers; thus, we recommend updating classification systems to allow greater confidence, for example, in standardising how interventions are grouped for subgroup analyses.

The majority of interventions we examined were delivered individually and face‐to‐face. However, the alternative modalities have never been more important and relevant, given the magnitude of change precipitated by the COVID‐19 pandemic (WHO 2020). This period saw the therapeutic milieu necessarily re‐invent itself online and through telephonic and video‐based support in order to initiate or maintain support to survivors. The remote delivery of care is likely to continue, and thus growing evidence on the efficacy of treatments delivered using diverse modalities is a priority in a post‐COVID‐19 era.

We classified nine studies as ongoing and of potential interest in updates to the current review (Ongoing studies). These are studies of both trauma‐focused psychotherapies (e.g. imaginal exposure (NCT04124380)), and non‐trauma‐focused approaches (e.g. skills training in affective and interpersonal regulation, which is compared to present‐centred therapy in a Canadian trial (NCT03429166)). Several of the studies examine different versions of CBT and target comorbid alcohol misuse (NCT04124380; NCT03703258; NCT04582695) and a range of other health problems (NCT03019497) associated with sexual violence and abuse. What is notable about these studies is a clear shift in emphasis to testing new modalities of delivery. These studies employ web‐based apps, online delivery and telehealth approaches. For example, a UK trial potentially meeting the inclusion criteria for the review (ISRCTN16806208), compares internet‐delivered trauma‐focused cognitive therapy for PTSD with internet‐delivered (non‐trauma‐focused) stress management therapy. These trials have strong potential to further strengthen the level of certainty about the effectiveness of a wide range of interventions.

To improve the certainty of the evidence, larger samples are needed. It is acknowledged that survivors of sexual violence and abuse are often minoritised and hard to reach; multi‐site trials, which have been rare, may alleviate some of these difficulties. Over four‐fifths of participants in our review represented the target group; however, we believe that heterogeneity would improve by incorporating sexual violence and abuse suffered in adulthood as an inclusion criterion, which is more common in recently published and ongoing trials. The participants were diverse in age; cultural and ethnic background; indicators of deprivation; education; and employment. However, the research largely represents North America.

Given the female bias in the studies included in this Review, we encourage greater gender diversity across recruited samples, and for studies to report the findings for minorities separately to aid meta‐analyses in different population subgroups. There is a clear need for the field to acknowledge male and gender diverse survivors of sexual violence and abuse in adulthood. In one ongoing study (NCT03794986), motivational interviewing is being used to engage male survivors. Reducing the barriers to access that men face is assisted by ensuring the inclusion of male survivors in developing the evidence base to treat PTSD and other impacts from sexual violence and abuse. Furthermore, strict study eligibility criteria may reduce the applicability of the research of those affected by complex PTSD and by severe mental health difficulties. It is understandable that safety and stabilisation of circumstances are important for commencing PTSD treatments. On the other hand, efforts need to be made to avoid this leading to the exclusion of certain groups in need of intervention. The health inequality that faces people in this context with complex PTSD, from migrant backgrounds, those who are homeless and people with learning difficulties, is reflected here in the exclusion of these groups from the research.

PTSD, depression and/or anxiety were included in nearly every study, and several measures of distress or mental wellbeing were employed, which we combined as 'global mental health/distress'. There were also quite a few studies that assessed trauma‐related cognitions, mainly using the Post‐Traumatic Cognitions Inventory (PTCI), which assesses negative beliefs about the self, the world and self‐blame. Other domains of importance to survivors may be overlooked but trials and systematic reviews are often necessarily restricted in the number of primary and secondary outcomes they can include. This highlights the importance of conducting process evaluations of trials and building the qualitative evidence base to allow understanding of the broader dimensions of people's experiences during and following interventions (Brown 2020). Ideally studies would: undertake clinical interviews using the Clinician‐Administered PTSD Checklist for DSM‐5 (CAPS‐5) or PTSD Symptom Scale – Interview (PSS‐I) at baseline and follow‐up; report clearly on proportions meeting clinical thresholds before and after treatments; employ validated self‐report PTSD assessments, reporting baseline and follow‐up means and standard deviations. There also needs to be improved reporting of the number of adverse events by group, or for studies to pre‐define the approach to harm assessment at the outset. Also, intention‐to‐treat analyses need to be given precedence, and researchers should follow strict protocols around randomisation and be clear in study protocols about selected thresholds for treatment completion. Such approaches will help standardise future research and increase the certainty of evidence by reducing methodological diversity and risk of bias.

Whilst the question about whether benefits are sustained over time persists, attaining such evidence in studies that lack an active comparison group may be impractical and even unethical. Thus, in searching for evidence of long‐term benefit, we recommend studies of head‐to‐head comparisons of different intervention types, with follow‐ups of over a year and longer‐term cohort studies.

Sexual violence and abuse has devastating physical, social and mental health impacts across the lifespan. No community is free of sexual assault, rape and abuse. As well as enabling access to justice, governments and societies have a duty to enable access to safe and effective treatments that preserve the well‐being of victims and survivors, address mental health sequelae, and minimise long‐term disability and the costs associated with sexual violence and abuse. We hope this review will provide useful insights on the state of the art in psychosocial interventions for sexual violence and abuse.

## History

Protocol first published: Issue 10, 2019

## Risk of bias

**Table:** Risk of bias for analysis 1.1 Psychosocial interventions versus inactive control; outcome 1: PTSD symptoms, post‐treatment

**Study**	**Bias**											
	**Randomisation process**		**Deviations from intended interventions**		**Missing outcome data**		**Measurement of the outcome**		**Selection of the reported results**		**Overall**	
	**Authors' judgement**	**Support for judgement**	**Authors' judgement**	**Support for judgement**	**Authors' judgement**	**Support for judgement**	**Authors' judgement**	**Support for judgement**	**Authors' judgement**	**Support for judgement**	**Authors' judgement**	**Support for judgement**
**Subgroup 1.1.1 Cognitive Behavioural Therapy**												
Falsetti 2008	High risk of bias	There was a lack of information on the process of randomisation and a greater than 10‐point difference on the means for PTSD at baseline with control group having the higher mean and 7 people who were assigned to the intervention group but did not take up the intervention were excluded from the pre‐treatment reported rates on PTSD.	High risk of bias	Participants were aware of assigned intervention. Per‐protocol analyses were used. Even in the imputed analyses the authors excluded the 7 people who had no treatment.	High risk of bias	52% of data were absent in the CBT‐group (M‐CET; 15/29) and 25.8% (8/31) of data were missing in the comparison group. We did not entirely agree with the authors that drop out was balanced. The authors omitted 7 individuals assigned to the intervention group but received no treatment.	Low risk of bias	The Modified PTSD Symptom Scale Self Report (MPSS‐SR; Falsetti, Resnick, Resick, & Kilpatrick, 1993) "is a modification of the PTSD Symptom Scale developed by Foa, Riggs, Dancu, and Rothbaum (1993) and allows for the assessment of both the frequency and severity of symptoms. The scale is composed of 17 items that correspond to PTSD symptom criteria in DSM‐IV. Falsetti et al. (1993) investigated the validity and reliability of this scale and reported high internal consistency (.96) as well as good concurrent validity with the SCID." "All assessments were completed in person by an independent evaluator unaware of treatment condition." However, the MPSS is a self report measure.	Some concerns	No protocol identified.	High risk of bias	Major concerns related to the approach to assignment and estimating the effect of assignment to intervention.
Foa 1999	High risk of bias	There is a lack of information on the process of assignment.	Some concerns	Blinding to intervention is not possible here. Fidelity in the study was high for example we are told that in addition to precise treatment guidelines and regular supervision, 9% of sessions were videotaped and assessed for presence of 52 intervention components across the treatments. On average, therapists completed 93% of the components prescribed for a given session in the corresponding protocol. On the other hand, it is unclear what was planned for the analyses and those who did not complete the intervention were excluded from analyses (N=8/30). Although we are told that there were no differences on pre‐treatment psychopathology measures between those who dropped out and those who completed, there is a lack of data to check this. We are told: "A significant difference on one demographic variable emerged: nonworking participants (30%) were more likely to drop out than participants who were working full or part time (10%)[...]." There was greater dropout from the intervention than from the waiting‐list.	Low risk of bias	In terms of those who did complete interventions, there was a good presence of data at post‐treatment.	Low risk of bias	Interview‐based assessment of PTSD by those qualified and trained and unaware of assignments.	Some concerns	We do not have much information on the planned analyses from a protocol or trial registry, most likely due to the lack of such standards at the time.	High risk of bias	The main concerns were the 'completer' analysis and lack of information on the full randomised sample and process.
Foa 1999	High risk of bias	There is a lack of information on the process of assignment.	Some concerns	Blinding to intervention is not possible here. Fidelity in the study was high for example we are told that in addition to precise treatment guidelines and regular supervision, 9% of sessions were videotaped and assessed for presence of 52 intervention components across the treatments. On average, therapists completed 93% of the components prescribed for a given session in the corresponding protocol. On the other hand, it is unclear what was planned for the analyses and those who did not complete the intervention were excluded from analyses (N=8/30). Although we are told that there were no differences on pre‐treatment psychopathology measures between those who dropped out and those who completed, there is a lack of data to check this. We are told: "A significant difference on one demographic variable emerged: nonworking participants (30%) were more likely to drop out than participants who were working full or part time (10%)[...]." There was greater dropout from the intervention than from the waiting‐list.	Low risk of bias	In terms of those who did complete interventions, there was a good presence of data at post‐treatment.	Low risk of bias	Interview‐based assessment of PTSD by those qualified and trained and unaware of assignments.	Some concerns	We do not have much information on the planned analyses from a protocol or trial registry, most likely due to the lack of such standards at the time.	High risk of bias	The main concerns were the 'completer' analysis and lack of information on the full randomised sample and process.
Foa 1999	High risk of bias	There is a lack of information on the process of assignment.	Some concerns	Blinding to intervention is not possible here. Fidelity in the study was high for example we are told that in addition to precise treatment guidelines and regular supervision, 9% of sessions were videotaped and assessed for presence of 52 intervention components across the treatments. On average, therapists completed 93% of the components prescribed for a given session in the corresponding protocol. On the other hand, it is unclear what was planned for the analyses and those who did not complete the intervention were excluded from analyses (N=8/30). Although we are told that there were no differences on pre‐treatment psychopathology measures between those who dropped out and those who completed, there is a lack of data to check this. We are told: "A significant difference on one demographic variable emerged: nonworking participants (30%) were more likely to drop out than participants who were working full or part time (10%)[...]." There was greater dropout from the intervention than from the waiting‐list.	Low risk of bias	In terms of those who did complete interventions, there was a good presence of data at post‐treatment.	Low risk of bias	Interview‐based assessment of PTSD by those qualified and trained and unaware of assignments.	Some concerns	We do not have much information on the planned analyses from a protocol or trial registry, most likely due to the lack of such standards at the time.	High risk of bias	The main concerns were the 'completer' analysis and lack of information on the full randomised sample and process.
Foa 2005	Some concerns	There is a lack of information about the how the allocation sequence was generated: "The study statistician assigned participants who provided informed consent to one of the three conditions using a weighted randomisation procedure such that participants were assigned to one of the active treatment conditions at a greater rate than to wait‐list (WL)." On the other hand, no significant differences between treatment arms were reported: "We first examined possible pretreatment differences on PSS‐I, BDI, SAS‐Work (SAS‐W), and SAS‐Social (SAS‐S) scores across treatment groups and sites using a series of separate single factor analyses of variance (ANOVAs)."	Low risk of bias	Participants and those delivering the intervention were aware of assignment but fidelity was high.	High risk of bias	Risk of bias may have been introduced due to level of missing data in intervention compared to control group and suggestion that there were important differences between those who provided data at the end (here, equivalent to intervention completers) and those who did not (here, equivalent to those who dropped out of the intervention).	Low risk of bias	The method of measuring the outcome was not inappropriate.	Some concerns	There was a lack of information, but unlikely to have been inappropriately selected/reported.	High risk of bias	Risk of bias may have been introduced due to systematic differences in the rates of missing data.
Foa 2005	Some concerns	There is a lack of information about the how the allocation sequence was generated: "The study statistician assigned participants who provided informed consent to one of the three conditions using a weighted randomisation procedure such that participants were assigned to one of the active treatment conditions at a greater rate than to wait‐list (WL)." On the other hand, no significant differences between treatment arms were reported: "We first examined possible pretreatment differences on PSS‐I, BDI, SAS‐Work (SAS‐W), and SAS‐Social (SAS‐S) scores across treatment groups and sites using a series of separate single factor analyses of variance (ANOVAs)."	Low risk of bias	Participants and those delivering the intervention were aware of assignment but fidelity was high.	High risk of bias	Risk of bias may have been introduced due to level of missing data in intervention compared to control group and suggestion that there were important differences between those who provided data at the end (here, equivalent to intervention completers) and those who did not (here, equivalent to those who dropped out of the intervention).	Low risk of bias	The method of measuring the outcome was not inappropriate.	Some concerns	There was a lack of information, but unlikely to have been inappropriately selected/reported.	High risk of bias	Risk of bias may have been introduced due to systematic differences in the rates of missing data.
Foa 2006	Some concerns	There was a lack of information about randomisation. "At the start of the study, participants were randomly assigned to either four sessions weekly of B‐CBT or AC." On the other hand, participants in the assessment condition and in the CBT (Brief CBT) did not seem to differ in terms of initial psychopathology or demographic variables.	Low risk of bias	Participants and intervention deliverers aware of assignment. Fidelity measures were comprehensive. Various analyses are presented: "..given our primary interest in testing the efﬁcacy of a brief, four session intervention, only data for completers will be presented here". Analyses were conducted using the last observation carried forward method (LOCF), with pre intervention scores for drop outs included in all analyses. Accordingly, in LOCF analyses, for missing data due to drop out, the last observation for that participant was used as their data for all subsequent analyses.	Some concerns	Missing data rates were fairly balanced: 9 (29%) in the B‐CBT condition, 10 (33.3%) in the AC. "No signiﬁcant differences between completers and drop outs emerged on pre‐intervention psychopathology and demographic variables. Therefore, given our primary interest in testing the efficacy of a brief, four session intervention, only data for completers will be presented here." However, the data on pre‐treatment are not fully reported.	Low risk of bias	"The pre intervention interview included the SCID and the PSS‐I." "Interviewers blind to treatment assignment conducted independent evaluations."	Some concerns	They refer to pre planned comparisons but we have not located where those planned comparisons were reported.	Some concerns	A lack of information on how the allocation sequence was generated and concealed and not enough information on pre planning of analyses and on the blinding of those conducting analyses.
Littleton 2016	Low risk of bias	"Participants were randomized to the interactive program or psychoeducational website based on a computerized coin flip."	Low risk of bias	Participants and intervention deliverers were likely aware of assignment but appropriate analysis appears to have been used. There is evidence that "External ratings of the competence of the feedback provided by therapists were uniformly high."	Some concerns	20/46 randomised (43%) to the intervention did not provide data at post‐treatment and 12/41 (29%) in the comparison did not provide data. The numbers for the PTSD outcome were even lower with 23/46 and 28/41 people self reporting PTSD in the intervention and comparison conditions, respectively. The figures look slightly better if 73 (N who completed a baseline survey and logged into the system) is used as the denominator. "There were no differences between drop‐outs and completers on demographics, child abuse history, and pre‐treatment PTSD, depression, or general anxiety." However, this refers to non‐completers of the intervention rather than non responders to the assessments.	Some concerns	Method used not inappropriate. Possible assessment of outcome influenced by knowledge of intervention received.	Some concerns	No information.	Some concerns	Concerns related to missing data and lack of information about blinding for PTSD interview assessment.
Resick 2002	Some concerns	"The design of the project involved random assignment of participants to CPT, PE, or MA [minimal attention group]." In the ITT sample, there were no significant differences in demographic characteristics among the three groups although not supplied. The CPT and wait list ITT groups were fairly balanced on scores on the PTSD outcome measure at baseline.	Low risk of bias	Fidelity checks were effectively built into the trial: "Independent raters who were not otherwise involved in the project conducted assessments of treatment adherence and therapist competence. All therapy sessions were videotaped and were available at random for rating." There was an effort "to assess therapist competence as well as adherence to the protocols by outside evaluators who were experts with the particular therapies being studied. The fidelity of the treatments was excellent, and the competence of the therapists was evaluated as satisfactory or better in the sample that was rated." "ITT analyses with LOCF were conducted on 171 participants, including the 13 women who never attended a session but had been accepted into the study."	Some concerns	Although imputation was used for analyses, 21/62 (34%) were lost from CPT and 7/47 (15%) lost from the control arm suggesting that there was not equivalence in dropout numbers. However, it is stated: "There were no significant differences between women who dropped out of therapy and those who completed therapy with regard to their initial PTSD or depression scores."	Low risk of bias	"The CAPS (Blake et al., 1990) is an interviewer‐administered diagnostic instrument that measures PTSD. It has been found to have excellent psychometric properties (Blake et al., 1995)." This is relevant as it was a clinical interview. Though it was unclear how much knowledge the assessors had about allocation.	Some concerns	Numerical result probably not selected on basis of results.	Some concerns	Some concerns mainly related to randomisation process.
Resick 2002	Some concerns	"The design of the project involved random assignment of participants to CPT, PE, or MA [minimal attention group]." In the ITT sample, there were no significant differences in demographic characteristics among the three groups although not supplied. The CPT and wait list ITT groups were fairly balanced on scores on the PTSD outcome measure at baseline.	Low risk of bias	Fidelity checks were effectively built into the trial: "Independent raters who were not otherwise involved in the project conducted assessments of treatment adherence and therapist competence. All therapy sessions were videotaped and were available at random for rating." There was an effort "to assess therapist competence as well as adherence to the protocols by outside evaluators who were experts with the particular therapies being studied. The fidelity of the treatments was excellent, and the competence of the therapists was evaluated as satisfactory or better in the sample that was rated." "ITT analyses with LOCF were conducted on 171 participants, including the 13 women who never attended a session but had been accepted into the study."	Some concerns	Although imputation was used for analyses, 21/62 (34%) were lost from CPT and 7/47 (15%) lost from the control arm suggesting that there was not equivalence in dropout numbers. However, it is stated: "There were no significant differences between women who dropped out of therapy and those who completed therapy with regard to their initial PTSD or depression scores."	Low risk of bias	"The CAPS (Blake et al., 1990) is an interviewer‐administered diagnostic instrument that measures PTSD. It has been found to have excellent psychometric properties (Blake et al., 1995)." This is relevant as it was a clinical interview. Though it was unclear how much knowledge the assessors had about allocation.	Some concerns	Numerical result probably not selected on basis of results.	Some concerns	Some concerns mainly related to randomisation process.
**Subgroup 1.1.2 Behavioural Therapy**												
Bell 2019	High risk of bias	It is indicated that "Twenty‐four eligible adults were enrolled on a first‐come, first‐served basis and alternately assigned between the LZNF group and HRVB group according to the order in which they returned their prescreening materials." Thus, this was quasi‐randomisation and there was a lack of allocation concealment. "The largest baseline differences were in the HRV measures, for which the LZNF group had higher initial levels."	Low risk of bias	There is little explanation of what participants were actually told about the intervention. It is indicated that "participants were unblinded to the training they were undergoing, which is a complication inherent to the use of active controls." However, little bias in this regard was anticipated.	Low risk of bias	Only one person was lost‐to‐follow‐up.	Some concerns	The study used the PCL‐5 which "closely correlates with the symptoms outlined in the Diagnostic and Statistical Manual of Mental Disorders (5th ed.; DSM‐V; American Psychiatric Association, 2013; Blevins, Weathers, Davis, Witte, & Domino, 2015) and assesses the frequency and severity of PTSD symptoms using a Likert‐type scale." there was little information on the blinding of assessors or the assessment process.	Some concerns	We did not locate a protocol.	High risk of bias	It is possible bias was introduced during the randomisation process.
Bomyea 2015	Low risk of bias	Individuals who completed all baseline assessments were randomly assigned to the HIC condition or the LIC condition based on a computer‐generated random number system prior to attending the first training session. Conditions were assigned by an independent third party using computer software, so that participants and research personnel remained blind to subjects’ conditions. Participants in the two groups did not differ on age or measures of clinical characteristics and no differences were identified in participants' ethnic background, education, income, or marital status.	Low risk of bias	Participants, carers and people delivering intervention not aware.	Some concerns	Half the sample did not return for the post‐treatment assessment. It was reported that there were no statistically significant differences in demographic characteristics, baseline clinical characteristics, or initial perceived treatment acceptability between women who completed the study and those who dropped out of treatment prematurely.	Low risk of bias	Not inappropriate.	Some concerns	There was no indication that those conducting the analysis were blind to the allocation of participants.	Some concerns	Main concern is that 50% of the sample did not return for the post‐treatment assessment but it was balanced across the two groups and there is no evidence to suggest that missingness on the PTSD outcome was related to the true value of the outcome.
Brady 2021	Some concerns	“After diagnostic assessment and completion of baseline outcome measures, a research assistant randomised participants to either trial condition using a virtual coin toss programme.” “Randomisation did not yield equal allocation across the two groups. There were also some notable group differences in the distribution of other participant characteristics.”	Low risk of bias	Participants may have been aware of their assignment. However, providers had a high level of training, a protocol was used and there was some reporting on fidelity.	Some concerns	Numbers were low in both groups for this feasibility study but retention was acceptable. However, when we obtained data from the authors, there were additional missing data relative to that reported in the article; we estimated the missing data to be around 30% at post‐treatment.	Low risk of bias	The choice of outcome measures was appropriate. Assessors were aware of the allocations which could be problematic however the measure was self report rather than clinician rating.	Low risk of bias	The lack of blinding around data raised some concerns however in the context of this feasibility trial we did not believe it increased risk around bias in reporting (i.e. considering the aims of the study).	Some concerns	This trial was a feasibility trial that encountered many barriers to recruitment, intervention completion and blinding and there was baseline imbalance with a tendency to better health in the intervention group which could have biased the PTSD result.
Gray 2020	Low risk of bias	"Participants were admitted to the study in cohorts of 10 and randomly assigned to treatment or control groups by the site manager. This assignment was based on a list of random numbers, previously generated at an independent location using Microsoft Excel 2016’s random number function."	Low risk of bias	Same therapist for all clients could introduce bias. "To ensure that our results reflected the most conservative and unbiased interpretation of the data, all computations were based on intent‐to‐treat analysis."	Low risk of bias	At the two week mark, there had been no loss‐to‐follow‐up.	Low risk of bias	"The PSS‐I is a 17‐item clinical interview for evaluating DSM–IV PTSD symptom severity, which is regularly used by the United States Department of Defense and VA." "Independent psychometricians, blinded to treatment condition, evaluated PTSD symptoms at intake, postwait (study week 5).."	Low risk of bias	We could not locate a trial protocol but previous studies were replicated and we checked the design in Gray et al. (2019). "The waitlist RCT design and methods followed previous studies of RTM (Gray et al., 2019; Tylee et al., 2017)."	Low risk of bias	Whilst there would be concerns about the longer term follow up, at 2 weeks post‐treatment, confidence in the the result is high.
Rajan 2020	Low risk of bias	There was a large proportion of individuals excluded post randomisation and prior to allocation which raised some concerns. Also some disrepancy with the trial record for example according to the trial record, 63 were enrolled. However, several strengths in this domain, including: "Preparation of the trial material and randomization was conducted by the independent data and safety monitoring board Karolinska Trial Alliance (KTA) via sequenced computer‐generated simple randomization with 1:1 allocation. Participants received sequentially numbered trial materials with concealed allocation. Allocation envelopes were kept and handled by the trial staff. Due to initial misunderstandings, the prepared sealed envelopes were initially not always picked in strict numeric order. However, the study was monitored and periodically reviewed by the KTA and the misunderstanding was corrected." No differences were reported around baseline characteristics.	Low risk of bias	"Participants could not be blinded to their treatment allocation due to the nature of this nonpharmacological intervention. Therapists involved in delivery of the intervention were also unblinded." "An intention to treat (ITT) approach, in which all participants are analyzed in the study arm to which they were randomly allocated, was applied to the analyses for primary and secondary outcomes. Differences in mean outcomes between intervention and comparison arms at time point two were analyzed using analyses of covariance (ANCOVAs), using baseline scores as covariates. "	Low risk of bias	For those allocated, presence of outcome data was satisfactory.	Some concerns	"The primary outcome was the difference between the two trial arms in mean PTSD symptoms as measured by Impact of Event Scale Revised (IES‐R) at time point two. The IES‐R has shown high internal consistency and the correlation between the IES‐R and the PTSD checklist (PCL5) has been shown to be high (Creamer et al., 2003; Murphy et al., 2017)." We therefore had few concerns despite lack of blinding to intervention and assessment. Due to a lack of resources, both the continuous monitoring of results and the analysis were done within the research team, without blinding.	Low risk of bias	It appears the recruitment stopped prematurely due to resource problems but the study detected an effect early on.	Some concerns	Being a newer study, it benefits from available guidelines and conventions which have been applied fairly well notwithstanding the usual challenges of blinding of providers and service users. There are some concerns related to not continuing to the target sample size (i.e. stopping at 36 participants or reporting interim results).
Rothbaum 1997	High risk of bias	No information on randomisation process was available. PTSD but not depression at baseline appeared balanced. There was a lack of information on other potential differences (e.g. on socio‐demographic variables).	Low risk of bias	There were steps to build in fidelity checks through independent evaluators. Most of those randomised remained in study and exclusions were very minimal (n=3). Two of these individuals could have been asked to complete the post‐treatment despite not receiving the intervention but were not.	Low risk of bias	Data for this outcome available for all, or nearly all, participants.	Low risk of bias	Method of measuring outcome not inappropriate.	Some concerns	No information.	High risk of bias	Concerns arose from the lack of information related to the randomisation process.
Walsh 2017	Some concerns	"The study was designed as a parallel trial with an allocation ratio of 1:1:1. A computerized random numbers generator was used to randomly assign participants to one of three conditions via a stratified blocked randomization procedure with variable block sizes of 9 or 12. Nurses who enrolled participants immediately after the study commenced (phase 1) accessed videos for participants via a secure internet link and administered videos prior to the medical exam. Following an approved change of scope, those enrolled in phase 2 (n = 126) were administered videos on CDs following the medical exam that were stored in envelopes prepared by a study coordinator and labelled only with a participant subject number until opened by the nurse, who was blind to study condition to that point." No baseline data for PTSD reported though in the (acute) context of the study, this is understandable.	Some concerns	Participants who received access to a video may have recognised they were in an intervention group and staff who assigned them may have recognised the different research conditions. Researchers altered how the videos were provided to participants at a later time though any effects were equivalent across groups: "A small proportion (n = 28; 18%) of participants received longer (18‐min) versions of the intervention and active control videos that were shown prior to the medical examination and included either the PPRS or PIRI video plus information about the examination. There were no differences among those who received the shorter and longer versions of the intervention and active control in pre‐examination". Completer analyses only and there were no data for PTSD at baseline to impute.	Some concerns	Data were available for around 70% across the groups. Because of lack of baseline data on PTSD or other mental health problems we could not determine if trauma levels influenced dropout. Potentially those with higher (or lower) trauma were lost, it is not possible to say.	Low risk of bias	PTSD Symptom Scale Self‐Report (PSS‐SR); validity not as high as PTSD clinical interview but acceptable properties. Self‐report measures subject to bias as participants may have recognised they were in an intervention or usual care group. We are told in a 2020 paper by Walsh et al. (https://doi.org/10.1016/j.addbeh.2019.106121) that "nurses administering the protocol and doctoral students conducting the follow‐ups were blind to study condition".	Low risk of bias	Published study protocol not available however records from the trial registry on ClinicalTrials.gov (NCT01430624) suggest PTSD was planned as a secondary outcome from the outset. It is unclear if those conducting the analysis were blinded to conditions. Low risk of bias was assigned as the researchers used just one PTSD assessment and we selected their post‐treatment data and their timepoints did not deviate from the records in the registry.	Some concerns	Unable to assess for baseline imbalance as no baseline on symptoms gathered and difficult to know if missing data correlated with trauma burden.
**Subgroup 1.1.3 Psychosocial (low‐intensity) interventions**												
Creech 2021	Low risk of bias	"Participants completed a self report baseline assessment and were randomized to the intervention or control condition using a standard randomization procedure within the computerized software. After completion of the baseline assessment, the (computer) narrator “flipped a coin” and women (N=153) were randomized into the control or SHE intervention. The randomization sequence was known only to the computer program and optimized for balanced assignment over time between the two conditions. This procedure resulted in n = 76 assigned to the intervention and n = 77 assigned to the control condition"	Low risk of bias	There were no concerns as it was a computerised intervention which minimised risk of bias.	Low risk of bias	Study effectively reported on missing data which was minimal at around 13% and did not differ between groups.	Low risk of bias	Appropriate measurement for the PTSD outcome done on an iPad.	Low risk of bias	The result was consistent with the trial protocol.	Low risk of bias	Good retention at post‐treatment data collection and generally well applied trial processes.
Miller 2015	High risk of bias	Little information provided. More individuals in the standard care (usual care) group reported rape (67.1%) compared to the video intervention condition (46.8%). Otherwise groups seems balanced on psychopathology and on other socio‐demographic factors though data on the latter are not provided by group.	Low risk of bias	Those in the video group would likely have recognised they were receiving an intervention. However, overall we detected a low risk of bias on this domain.	High risk of bias	There was high but equivalent attrition at the post‐treatment assessment in both groups: 47/85 were lost to follow up in the standard care group and 48/79 in the video intervention group. There is no information to allow comparison of any differences on baseline characteristics of those who were lost at follow up and those retained. It is possible that people with higher levels of trauma were less likely to participate in follow ups. There is just not enough information provided to evaluate this. The authors comment: "the participants who participated in the follow‐up assessments may be characteristically different than those who dropped out. "	Low risk of bias	Method of measuring outcome not inappropriate.	Some concerns	No information. The authors highlight that their analyses were limited by the high loss‐to‐follow‐up.	High risk of bias	Lack of information provided about generation of the allocation sequence and allocation concealment. This raised concerns especially given that there appeared to be a higher proportion of severe sexual violence (rape over sexual assault) in the standard care group. High attrition and lack of information to evaluate if people who were lost to follow up were different to those who were retained in the study.
Sikkema 2018	Low risk of bias	"Patients who met eligibility criteria and gave written informed consent to the trial were scheduled to complete the baseline assessment (typically within 1 – 5 days following screening), prior to receiving the standard adherence counseling sessions. Participants were randomly assigned (1:1) to the standard of care control condition (SoC: 3 adherence “readiness” sessions) or the experimental intervention condition (SoC, plus ImpACT) using a small block (size 8 or 10) randomization procedure. Condition assignments were placed in sealed envelopes, blinded to study staff until assignment." "No significant differences for any of these sample characteristics were observed between treatment conditions." However, there were differences on the outcome measure at baseline with higher PTSD in the intervention group.	Low risk of bias	"Due to the nature of the intervention conditions, neither participants nor staff could be blinded to condition assignment." "Fidelity to the ImpACT intervention protocol for individual sessions was high. An independent coder estimated level of fidelity to the ImpACT intervention for the individual sessions, with 97.8% coverage specific to each session’s protocol. Due to challenges in group session attendance, limited quality assurance data for group sessions were available, although for the group sessions that were implemented, intervention fidelity was also high."	Some concerns	13/32 lost from the interventon group and 6/32 from the control arm at the post‐treatment point. Retention does appear to favour the control condition however authors state: "Retention in the 3‐ and/or 6‐month assessment was 85.9% and no significant differences by study condition in the percentage of participants lost to follow‐up were observed." Reasons for dropout were provided and were mainly related to people being unreachable. However, given that the intervention group had higher PTSD, it is possible this influenced dropout.	Low risk of bias	Method of measuring outcome not inappropriate.	Low risk of bias	Reporting was done in line with the trial registration information (NCT02223390).	Some concerns	Retention was improved at the 6 month follow up however this assessment focuses on the first post‐treatment assessment and there was higher loss of data in the intervention group relative to comparison, and not enough information to assess if those lost had higher levels of PTSD.
Walsh 2017	Some concerns	"The study was designed as a parallel trial with an allocation ratio of 1:1:1. A computerized random numbers generator was used to randomly assign participants to one of three conditions via a stratified blocked randomization procedure with variable block sizes of 9 or 12. Nurses who enrolled participants immediately after the study commenced (phase 1) accessed videos for participants via a secure internet link and administered videos prior to the medical exam. Following an approved change of scope, those enrolled in phase 2 (n = 126) were administered videos on CDs following the medical exam that were stored in envelopes prepared by a study coordinator and labelled only with a participant subject number until opened by the nurse, who was blind to study condition to that point." No baseline data for PTSD reported though in the (acute) context of the study, this is understandable.	Some concerns	Participants who received access to a video may have recognised they were in an intervention group and staff who assigned them may have recognised the different research conditions. Researchers altered how the videos were provided to participants at a later time though any effects were equivalent across groups: "A small proportion (n = 28; 18%) of participants received longer (18‐min) versions of the intervention and active control videos that were shown prior to the medical examination and included either the PPRS or PIRI video plus information about the examination. There were no differences among those who received the shorter and longer versions of the intervention and active control in pre‐examination". Completer analyses only and there were no data for PTSD at baseline to impute.	Some concerns	Data were available for around 70% across the groups. Because of lack of baseline data on PTSD or other mental health problems we could not determine if trauma levels influenced dropout. Potentially those with higher (or lower) trauma were lost, it is not possible to say.	Low risk of bias	PTSD Symptom Scale Self‐Report (PSS‐SR); validity not as high as PTSD clinical interview but acceptable properties. Self‐report measures subject to bias as participants may have recognised they were in an intervention or usual care group. We are told in a 2020 paper by Walsh et al. (https://doi.org/10.1016/j.addbeh.2019.106121) that "nurses administering the protocol and doctoral students conducting the follow‐ups were blind to study condition".	Low risk of bias	Published study protocol not available however records from the trial registry on ClinicalTrials.gov (NCT01430624) suggest PTSD was planned as a secondary outcome from the outset. It is unclear if those conducting the analysis were blinded to conditions. Low risk of bias was assigned as the researchers used just one PTSD assessment and we selected their post‐treatment data and their timepoints did not deviate from the records in the registry.	Some concerns	Unable to assess for baseline imbalance as no baseline on symptoms gathered and difficult to know if missing data correlated with trauma burden.

**Table:** Risk of bias for analysis 1.3 Psychosocial interventions versus inactive control; outcome 2: Depressive symptoms, post‐treatment

**Study**	**Bias**											
	**Randomisation process**		**Deviations from intended interventions**		**Missing outcome data**		**Measurement of the outcome**		**Selection of the reported results**		**Overall**	
	**Authors' judgement**	**Support for judgement**	**Authors' judgement**	**Support for judgement**	**Authors' judgement**	**Support for judgement**	**Authors' judgement**	**Support for judgement**	**Authors' judgement**	**Support for judgement**	**Authors' judgement**	**Support for judgement**
**Subgroup 1.3.1 Cognitive Behavioural Therapy**												
Falsetti 2008	High risk of bias	There was a lack of information on the process of randomisation and a greater than 10‐point difference on the means for PTSD at baseline with control group having the higher mean and 7 people who were assigned to the intervention group but did not take up the intervention were excluded from the pre‐treatment reported rates on PTSD.	High risk of bias	The approach to estimating the effect of assignment to intervention (per‐protocol) may have introduced bias.	High risk of bias	13/29 people did not complete the intervention or assessment and a further two did not complete the follow‐up assessment at post‐treatment; 8 people did not complete the assessment in the control arm. Dropout was double in the intervention arm and the data provided on differences between those retained and lost is not reliable as it excludes the 7 who had no sessions. We do not know baseline scores on depression for those who dropped out but it is possible those that dropped out had poorer health.	Low risk of bias	"All assessments were completed in person by an independent evaluator unaware of treatment condition."	Some concerns	No information.	High risk of bias	High likelihood of bias related to randomisation and/or handling of intervention non‐completers, dropouts, and missing data.
Foa 1999	High risk of bias	There is a lack of information on the process of assignment.	Some concerns	Participants would have known if they were on a wait‐list or in a treatment group. Fidelity measures were comprehensive. Some concerns arose from per protocol analysis.	Low risk of bias	It appears proportions of missing outcome data were low (at least for those included in analyses (i.e. intervention completers)).	Low risk of bias	The BDI was used which is a self‐report measure. It has been widely used across trials. The authors state: "The BDI (Beck, Ward, Mendelsohn, Mock, & Erbaugh, 1961) is a 21‐item inventory measuring depression. Split‐half reliability was .93. Correlations with clinician ratings of depression ranged from .62 to.66." It is not inconceivable participants provided responses in the expected direction but overall seems unlikely. "Independent evaluators were female clinicians with at least a master's degree who received extensive training in administration of the instruments and were unaware of treatment assignment."	Some concerns	We do not have much information on the planned analyses from a protocol or trial registry most likely due to the lack of such standards at the time.	High risk of bias	Concerns arose from a comparison of a wait‐list group to intervention completers and lack of information on randomisation/concealment.
Foa 1999	High risk of bias	There is a lack of information on the process of assignment.	Some concerns	Participants would have known if they were on a wait‐list or in a treatment group. Fidelity measures were comprehensive. Some concerns arose from per protocol analysis.	Low risk of bias	It appears proportions of missing outcome data were low (at least for those included in analyses (i.e. intervention completers)).	Low risk of bias	The BDI was used which is a self‐report measure. It has been widely used across trials. The authors state: "The BDI (Beck, Ward, Mendelsohn, Mock, & Erbaugh, 1961) is a 21‐item inventory measuring depression. Split‐half reliability was .93. Correlations with clinician ratings of depression ranged from .62 to.66." It is not inconceivable participants provided responses in the expected direction but overall seems unlikely. "Independent evaluators were female clinicians with at least a master's degree who received extensive training in administration of the instruments and were unaware of treatment assignment."	Some concerns	We do not have much information on the planned analyses from a protocol or trial registry most likely due to the lack of such standards at the time.	High risk of bias	Concerns arose from a comparison of a wait‐list group to intervention completers and lack of information on randomisation/concealment.
Foa 1999	High risk of bias	There is a lack of information on the process of assignment.	Some concerns	Participants would have known if they were on a wait‐list or in a treatment group. Fidelity measures were comprehensive. Some concerns arose from per protocol analysis.	Low risk of bias	It appears proportions of missing outcome data were low (at least for those included in analyses (i.e. intervention completers)).	Low risk of bias	The BDI was used which is a self‐report measure. It has been widely used across trials. The authors state: "The BDI (Beck, Ward, Mendelsohn, Mock, & Erbaugh, 1961) is a 21‐item inventory measuring depression. Split‐half reliability was .93. Correlations with clinician ratings of depression ranged from .62 to.66." It is not inconceivable participants provided responses in the expected direction but overall seems unlikely. "Independent evaluators were female clinicians with at least a master's degree who received extensive training in administration of the instruments and were unaware of treatment assignment."	Some concerns	We do not have much information on the planned analyses from a protocol or trial registry most likely due to the lack of such standards at the time.	High risk of bias	Concerns arose from a comparison of a wait‐list group to intervention completers and lack of information on randomisation/concealment.
Foa 2005	Some concerns	There is a lack of information about the how the allocation sequence was generated: "The study statistician assigned participants who provided informed consent to one of the three conditions using a weighted randomisation procedure such that participants were assigned to one of the active treatment conditions at a greater rate than to wait‐list (WL)." On the other hand, no significant differences between treatment arms were reported: "We first examined possible pretreatment differences on PSS‐I, BDI, SAS‐Work (SAS‐W), and SAS‐Social (SAS‐S) scores across treatment groups and sites using a series of separate single factor analyses of variance (ANOVAs)."	Low risk of bias	"Therapists made contact with the participants and arranged initial therapy appointments with those assigned to active treatment, and they also informed them of the specific treatment condition at the first session. WL participants were informed by phone that they had been assigned to the WL condition." Fidelity was high in the intervention arm. "Therapists completed 97% of the components prescribed in the protocol." They conducted both ITT analysis and a per protocol analysis.	High risk of bias	There were differences between completers and non completers and there was greater dropout in the intervention groups relative to the wait‐list. For example, 66% of those those in the CBT (PE) group returned data at post‐treatment and nearly all those in the waiting list group did (22/26). The use of imputed data was not assumed to correct for bias.	Low risk of bias	Use of BDI was appropriate and there is ample support for validity and reliability. "Independent evaluations were conducted at pretreatment and posttreatment and 3‐, 6‐, and 12‐month posttreatment. All evaluations were conducted by trained doctoral or master’s level CTSA clinicians who were blind to study condition."	Some concerns	No clear analysis plan available nor indication of blind analyses.	High risk of bias	Differences detected on baseline characteristics between completers and non‐completers of the interventions, and there was differential drop‐out and missing data across intervention and wait‐list.
Foa 2005	Some concerns	There is a lack of information about the how the allocation sequence was generated: "The study statistician assigned participants who provided informed consent to one of the three conditions using a weighted randomisation procedure such that participants were assigned to one of the active treatment conditions at a greater rate than to wait‐list (WL)." On the other hand, no significant differences between treatment arms were reported: "We first examined possible pretreatment differences on PSS‐I, BDI, SAS‐Work (SAS‐W), and SAS‐Social (SAS‐S) scores across treatment groups and sites using a series of separate single factor analyses of variance (ANOVAs)."	Low risk of bias	"Therapists made contact with the participants and arranged initial therapy appointments with those assigned to active treatment, and they also informed them of the specific treatment condition at the first session. WL participants were informed by phone that they had been assigned to the WL condition." Fidelity was high in the intervention arm. "Therapists completed 97% of the components prescribed in the protocol." They conducted both ITT analysis and a per protocol analysis.	High risk of bias	There were differences between completers and non completers and there was greater dropout in the intervention groups relative to the wait‐list. For example, 66% of those those in the CBT (PE) group returned data at post‐treatment and nearly all those in the waiting list group did (22/26). The use of imputed data was not assumed to correct for bias.	Low risk of bias	Use of BDI was appropriate and there is ample support for validity and reliability. "Independent evaluations were conducted at pretreatment and posttreatment and 3‐, 6‐, and 12‐month posttreatment. All evaluations were conducted by trained doctoral or master’s level CTSA clinicians who were blind to study condition."	Some concerns	No clear analysis plan available nor indication of blind analyses.	High risk of bias	Differences detected on baseline characteristics between completers and non‐completers of the interventions, and there was differential drop‐out and missing data across intervention and wait‐list.
Foa 2006	Some concerns	There was a lack of information about randomisation. "At the start of the study, participants were randomly assigned to either four sessions weekly of B‐CBT or AC." On the other hand, participants in the assessment condition and in the CBT (Brief CBT) did not seem to differ in terms of initial psychopathology or demographic variables.	Some concerns	Removal of those who did not complete the intervention from primary analyses raises concerns although also presents data based on last observation carried forward method.	Some concerns	Dropouts were distributed as follows: 9 (29%) in the B‐CBT condition, 10 (33.3%) in the AC ‐ high but equivalent. "No signiﬁcant differences between completers and drop outs emerged on pre‐intervention psychopathology and demographic variables. Therefore, given our primary interest in testing the efficacy of a brief, four session intervention, only data for completers will be presented here." However, the data on pre‐treatment are not fully reported.	Low risk of bias	"The Beck Depression Inventory (BDI; Beck, Ward, Mendelson, Mock, & Erbaugh, 1961). The BDI is a 21‐item self‐report measure that assesses the severity of depression in adolescents and adults with scores ranging from 0 to 63. Test‐retest reliability (.60) and internal consistency (.81) are good." "Interviewers blind to treatment assignment conducted independent evaluations" however it was self‐report for the BDI.	Some concerns	No protocol declared.	Some concerns	The randomisation process is not described and an intervention completer analysis is done.
Littleton 2016	Low risk of bias	"Participants were randomized to the interactive program or psychoeducational website based on a computerized coin flip."	Low risk of bias	There is evidence that "External ratings of the competence of the feedback provided by therapists were uniformly high."	Some concerns	20/46 randomised (43%) to the intervention did not provide data at post‐treatment and 12/41 (29%) in the comparison did not provide data. The numbers for the depression outcome were even lower with 18/46 and 24/41 people self reporting depression in the intervention and comparison conditions, respectively. The figures look slightly better if 73 (N who completed a baseline survey and logged into the system) is used as the denominator. In reference to those who experienced the programs, it is indicated that "There were no differences between drop‐outs and completers on demographics, child abuse history, and pre‐treatment PTSD, depression, or general anxiety." It is less clear what the differences were between those who provided post treatment data and those that did not.	Low risk of bias	"The Center for Epidemiological Studies‐Depression Scale was administered to assess current depressive symptoms (CES‐D; Radloff, 1977)." This is a self‐reported measure.	Some concerns	No information.	Some concerns	Some concerns around missing outcomes and selection of reporting results .
Resick 2002	Some concerns	"The design of the project involved random assignment of participants to CPT, PE, or MA [minimal attention group]." In the ITT sample, there were no significant differences in demographic characteristics among the three groups although not supplied. The CPT and wait list ITT groups were fairly balanced on scores on the PTSD outcome measure at baseline.	Low risk of bias	Independent raters who were not otherwise involved in the project conducted assessments of treatment adherence and therapist competence. There was a high level of treatment adherence and competence. "ITT analyses with LOCF were conducted on 171 participants, including the 13 women who never attended a session but had been accepted into the study."	Some concerns	Although imputation was used, 21/62 (34%) were lost from CPT and 7/47 (15%) lost from the control arm. "There were no significant differences between women who dropped out of therapy and those who completed therapy with regard to their initial PTSD or depression scores."	Low risk of bias	Method of outcome assessment not inappropriate.	Low risk of bias	There were several analyses but we drew on the ITT data and the authors were quite transparent and justificed their approach.	Some concerns	Beyond some concerns about the randomisation process, this study was relatively well‐designed for this comparison up to post‐treatment.
Resick 2002	Some concerns	"The design of the project involved random assignment of participants to CPT, PE, or MA [minimal attention group]." In the ITT sample, there were no significant differences in demographic characteristics among the three groups although not supplied. The CPT and wait list ITT groups were fairly balanced on scores on the PTSD outcome measure at baseline.	Low risk of bias	Independent raters who were not otherwise involved in the project conducted assessments of treatment adherence and therapist competence. There was a high level of treatment adherence and competence. "ITT analyses with LOCF were conducted on 171 participants, including the 13 women who never attended a session but had been accepted into the study."	Some concerns	Although imputation was used, 21/62 (34%) were lost from CPT and 7/47 (15%) lost from the control arm. "There were no significant differences between women who dropped out of therapy and those who completed therapy with regard to their initial PTSD or depression scores."	Low risk of bias	Method of outcome assessment not inappropriate.	Low risk of bias	There were several analyses but we drew on the ITT data and the authors were quite transparent and justificed their approach.	Some concerns	Beyond some concerns about the randomisation process, this study was relatively well‐designed for this comparison up to post‐treatment.
Rothbaum 2005	High risk of bias	Information about imbalances (e.g. "Participants in the EMDR condition exhibited significantly higher overall PTSD symptoms, higher levels of intrusive symptoms on the PSS and higher levels of depression, dissociation and trait anxiety than the PE group." ) raised concerns about the process of randomisation for which there was a lack of information.	Some concerns	Measures were put in place to ensure fidelity of interventions. A per protocol approach was used. There was a lack of information on characteristics of those excluded from analyses.	Some concerns	Dropout was moderately high for post‐treatment evaluation. However, the dropout rate across the groups was not signiﬁcantly different: PE: 13.0% (n=3); EMDR: 20.0% (n =5); WAIT: 16.7% (n=4).	Low risk of bias	Self report used for the BDI.	Some concerns	No information.	High risk of bias	There was a lack of information about randomisation and imbalance on the outcome measure at baseline.
**Subgroup 1.3.2 Behavioural Therapy**												
Bomyea 2015	Low risk of bias	Individuals who completed all baseline assessments were randomly assigned to the HIC condition or the LIC condition based on a computer‐generated random number system prior to attending the first training session. Conditions were assigned by an independent third party using computer software, so that participants and research personnel remained blind to subjects’ conditions. Participants in the two groups did not differ on age or measures of clinical characteristics and no differences were identified in participants' ethnic background, education, income, or marital status.	Low risk of bias	"Conditions were assigned by an independent third party using computer software, so that participants and research personnel remained blind to subjects’ conditions." Given that it was a computer based intervention it is unlikely that personnel became aware.	Some concerns	Large proportion of missing responses; however, this was equal across two groups. Imputing outcomes cannot be assumed to correct bias associated with missing data.	Low risk of bias	BDI‐II is widely used as a self‐report measure of depressive symptoms.	Some concerns	It is not clear if the analysis was conducted by a blinded statistician.	Some concerns	Main concern is that 50% of the sample did not return for the post‐treatment assessment but it was balanced across the two groups and there is no evidence to suggest that missingness on the PTSD outcome was related to the true value of the outcome.
Brady 2021	Some concerns	“After diagnostic assessment and completion of baseline outcome measures, a research assistant randomised participants to either trial condition using a virtual coin toss programme.” “Randomisation did not yield equal allocation across the two groups. There were also some notable group differences in the distribution of other participant characteristics.”	Low risk of bias	Protocols were followed and this part of the research was well‐reported.	Some concerns	We missed around 30% of data at the post‐treatment timepoint.	Low risk of bias	The tool was appropriate.	Low risk of bias	We had some concerns about lack of blinding on analysis. On balance, given aims of this study, we reached a judgement of low risk.	Some concerns	The imbalance at baseline was the main concern though it did not appear to affect the depression outcome at baseline. Numbers were low to begin with and we had high missing data.
Rothbaum 1997	High risk of bias	No information on randomisation process was available. PTSD but not depression at baseline appeared balanced. There was a lack of information on other potential differences (e.g. on socio‐demographic variables).	Low risk of bias	Some efforts to build in fidelity checks through independent evaluators. Most of those randomised remained in the study and exclusions were very minimal (n=3). Two of these individuals could have been asked to complete the post‐treatment despite not receiving the intervention but were not.	Low risk of bias	Dropout at post‐treatment was minimal and similar across the groups	Low risk of bias	Self‐report is used for the BDI.	Some concerns	Not analysed in accordance with a pre‐specified analysis plan.	High risk of bias	The randomisation process introduced high risk of bias, with no detail on how it was done and there were some concerns about the difference on the outcome measure (depression) between the two groups at baseline with the control arm having higher scores.
Rothbaum 2005	High risk of bias	Information about imbalances (e.g. "Participants in the EMDR condition exhibited significantly higher overall PTSD symptoms, higher levels of intrusive symptoms on the PSS and higher levels of depression, dissociation and trait anxiety than the PE group." ) raised concerns about the process of randomisation for which there was a lack of information.	Some concerns	Measures were put in place to ensure fidelity of interventions. A per protocol approach was used. There was a lack of information on characteristics of those excluded from analyses.	Some concerns	Dropout was moderately high for post‐treatment evaluation. However, the dropout rate across the groups was not signiﬁcantly different: PE: 13.0% (n=3); EMDR: 20.0% (n =5); WAIT: 16.7% (n=4).	Low risk of bias	Self report used for the BDI.	Some concerns	No information.	High risk of bias	There was a lack of information about randomisation and imbalance on the outcome measure at baseline.
**Subgroup 1.3.3 Psychosocial (low‐intensity) interventions**												
Abrahams 2010	Some concerns	It is indicated that a computer‐generated randomisation list was generated by the study statistician and random block of four, six and eight were used to ensure balance in the two arms. Participants were then allocated to a group by the study coordinator after the initial data had been collected. The contact information of intervention group participants was sent to the counsellors who commenced the intervention within 12 hours.	High risk of bias	The authors highlight a concern of their own: "We cannot exclude the possibility that there were weaknesses in intervention delivery such as variations in the number of telephone calls, which could have attenuated the differences between the intervention and the control group." It is indicated that in delivering the intervention: "The mean number of total calls differed between the two study sites with more calls made to Cape Town participants compared to those in Mthatha. Many more calls at the Cape Town site were made to a third party or were unanswered."	Some concerns	We communicated with the author to obtain depression data on continuous scale and received 140 cases of the 274 randomised. Proportions of missing data do not vary by group.	Low risk of bias	CES‐D is widely used measure of depression and in the setting. It is unlikely that participants' own knowledge of allocation influenced responses. Also "Fieldworkers who did the final interview were blinded to study arm, but this may have been disclosed by the participants during the final interviews."	Some concerns	We did not locate protocol or registry record to examine planned analyses and a per protocol analysis was undertaken on the depression outcome.	High risk of bias	Differences within the intervention sites and deviations from protocol in delivery raised concerns.
Bowland 2012	Some concerns	The following approach may have introduced bias: "Women were paired on scores from a spiritual distress scale (SDS) and then randomised into treatment or control groups using a random number table." However, it is indicated that "No differences were found between the treatment and control groups at pretest."	Low risk of bias	Participants and intervention deliverers likely aware of assignment but it appears an appropriate analysis was used.	Low risk of bias	The study gathered post‐treatment data from all but one of those randomised.	Low risk of bias	"The Geriatric Depression Scale (GDS) is used extensively with older adults (Yesavage et al., 1982–1983)." "The testers were blind to the treatment conditions of the participants". Much of the data collection was self‐report: "Nearly all participants completed paper and pencil tests at group testing sessions. Occasionally tests were completed in individual sessions with the testers or by phone."	Some concerns	No information.	Some concerns	There was a lack of information on some domains like the randomisation process.

**Table:** Risk of bias for analysis 1.5 Psychosocial interventions versus inactive control; outcome 3: Dropout from treatment

**Study**	**Bias**											
	**Randomisation process**		**Deviations from intended interventions**		**Missing outcome data**		**Measurement of the outcome**		**Selection of the reported results**		**Overall**	
	**Authors' judgement**	**Support for judgement**	**Authors' judgement**	**Support for judgement**	**Authors' judgement**	**Support for judgement**	**Authors' judgement**	**Support for judgement**	**Authors' judgement**	**Support for judgement**	**Authors' judgement**	**Support for judgement**
Bell 2019	High risk of bias	It is indicated that "Twenty‐four eligible adults were enrolled on a first‐come, first‐served basis and alternately assigned between the LZNF group and HRVB group according to the order in which they returned their prescreening materials." Thus, this was quasi‐randomisation and there was a lack of allocation concealment. "The largest baseline differences were in the HRV measures, for which the LZNF group had higher initial levels."	Low risk of bias	Participants and intervention deliverers probably knew assignment but analysis likely appropriate.	Low risk of bias	Data available for all or nearly all.	Low risk of bias	Method of measuring outcome not inappropriate.	Low risk of bias	Probably analysed in accordance with pre‐specified plan.	High risk of bias	Although this outcome is about dropout from the trial and many features lead to confidence about that result, there remains the issue that the randomisation process was susceptible to bias.
Bomyea 2015	Low risk of bias	Individuals who completed all baseline assessments were randomly assigned to the HIC condition or the LIC condition based on a computer‐generated random number system prior to attending the first training session. Conditions were assigned by an independent third party using computer software, so that participants and research personnel remained blind to subjects’ conditions. Participants in the two groups did not differ on age or measures of clinical characteristics and no differences were identified in participants' ethnic background, education, income, or marital status.	Low risk of bias	Unaware of assignment.	Low risk of bias	7/22 (intervention) v 10/20 (control) ‐ based on flowchart we selected the Ns that did not receive the intervention and started but discontinued and use the randomised ns as the denominators.	Some concerns	However it is not clear if treatment completion meant all 8 sessions were attended and this was important to state.	Some concerns	For dropout, it is fairly straightforward.	Some concerns	This study adequately measures and reports treatment dropout though more clarity on required number of sessions (definition of completion) would have been useful.
Brady 2021	Some concerns	“After diagnostic assessment and completion of baseline outcome measures, a research assistant randomised participants to either trial condition using a virtual coin toss programme.” “Randomisation did not yield equal allocation across the two groups. There were also some notable group differences in the distribution of other participant characteristics.”	Low risk of bias	The interventions were delivered effectively to the best of our knowledge.	Low risk of bias	Dropout from the study interventions was low (as distinct from missing data on outcomes).	Low risk of bias	We had no concerns that the researchers were able to accurately report on the numbers who exited the intervention which was 2/15 (intervention) and 3/10 (minimally active control)	Low risk of bias	No concerns that there was biased reporting on dropout.	Some concerns	It is conceivable that the imbalance at baseline on characteristics of participants could have interacted with outcomes including susceptibility to leaving the intervention.
Foa 2006	Some concerns	There was a lack of information about randomisation. "At the start of the study, participants were randomly assigned to either four sessions weekly of B‐CBT or AC." On the other hand, participants in the assessment condition and in the CBT (Brief CBT) did not seem to differ in terms of initial psychopathology or demographic variables.	Low risk of bias	Completer analysis was undertaken however the current outcome is not so much affected by this	Low risk of bias	No obvious risk to ascertaining the dropout rates.	Some concerns	9/31 v 10/30, however did not detail how many sessions required to consider completion. It was unclear what thresholds were used for brief CBT v assessment control condition.	Some concerns	Numerical result here not assessed based on a selection made by the researchers.	Some concerns	Information on number of sessions lacking by group and what precisely constituted dropout from treatment.
Littleton 2016	Low risk of bias	"Participants were randomized to the interactive program or psychoeducational website based on a computerized coin flip."	Low risk of bias	Participants and intervention deliverers were likely aware of assignment but appropriate analysis appears to have been used. There is evidence that "External ratings of the competence of the feedback provided by therapists were uniformly high."	Low risk of bias	Data available for all or nearly all participants.	High risk of bias	Some concern that there was a low threshold for treatment completion, suggesting the 'dose' ended up as low for most participants. e.g. just "Six participants (15.8%) completed the entire program." A higher expectation about 'completion level' would have meant greater dropout from treatment. It is difficult to align what is in text with the numbers in the flow diagram for the study and so we selected the Ns reported the study's Figure 1 for non initiation and discontinued. It is not entirely clear how dropout was ascertained in the control (minimal intervention).	Some concerns	There are several ways of expressing treatment dropout and the authors may have selected a perspective that made the result look more favourable.	High risk of bias	The study reports a favourable rate of treatment adherence when in fact only 15.8% of people in the treatment group went through the full program.

**Table:** Risk of bias for analysis 1.6 Psychosocial interventions versus inactive control; outcome 4: Adverse events

**Study**	**Bias**											
	**Randomisation process**		**Deviations from intended interventions**		**Missing outcome data**		**Measurement of the outcome**		**Selection of the reported results**		**Overall**	
	**Authors' judgement**	**Support for judgement**	**Authors' judgement**	**Support for judgement**	**Authors' judgement**	**Support for judgement**	**Authors' judgement**	**Support for judgement**	**Authors' judgement**	**Support for judgement**	**Authors' judgement**	**Support for judgement**
Abrahams 2010	Some concerns	It is indicated that a computer‐generated randomisation list was generated by the study statistician and random block of four, six and eight were used to ensure balance in the two arms. Participants were then allocated to a group by the study coordinator after the initial data had been collected. The contact information of intervention group participants was sent to the counsellors who commenced the intervention within 12 hours.	Some concerns	"We cannot exclude the possibility that there were weaknesses in intervention delivery such as variations in the number of telephone calls, which could have attenuated the differences between the intervention and the control group."	Low risk of bias	Low loss to follow up suggesting there was good opportunity to observe adverse events.	Low risk of bias	There was low risk as the approach to measuring adverse effects seemed appropriate (monitoring worsening of outcome results)	Some concerns	"We also initially planned blinded external statistical analysis. Due to a lack of resources, both the continuous monitoring of results and the analysis were done within the research team, without blinding."	Some concerns	Low loss‐to‐follow‐up suggesting there was good opportunity to observe adverse events and the team was clearly attuned to importance of monitoring for adverse events, however, there were some problems with blinding of results.
Brady 2021	Some concerns	“After diagnostic assessment and completion of baseline outcome measures, a research assistant randomised participants to either trial condition using a virtual coin toss programme.” “Randomisation did not yield equal allocation across the two groups. There were also some notable group differences in the distribution of other participant characteristics.”	Low risk of bias	We did not detect any bias related to the intervention that could have influenced adverse events.	Low risk of bias	Care was taken to consider and report reasons for people leaving the study and there was high level of contact with participants through the study giving opportunity to identify adverse events.	Low risk of bias	We found no indication of measurement bias.	Low risk of bias	Reasons were provided for withdrawals.	Some concerns	It is conceivable that the imbalance at baseline on characteristics of participants could have interacted with outcomes including susceptibility to adverse events.
Gray 2020	Low risk of bias	"Participants were admitted to the study in cohorts of 10 and randomly assigned to treatment or control groups by the site manager. This assignment was based on a list of random numbers, previously generated at an independent location using Microsoft Excel 2016’s random number function."	Low risk of bias	No indication of deviation from planned intervention.	Low risk of bias	There was high completion of the intervention and responses rates in the post‐treatment phase were high so opportunity for adverse event reporting existed. There was greater loss over time however it is expected adverse events would have occurred during or soon after the intervention.	Some concerns	"No reportable adverse events occurred." However, there was not a clear description of how adverse events were monitored or recorded.	Some concerns	Not entirely clear how they planned to measure and report adverse events.	Some concerns	Whilst risk of bias is minimal given general reporting of this trial, it was difficult to assess in regards to this outcome due to lack of methodological information.
Krakow 2001	Low risk of bias	"To mask treatment assignment, patients mailed back a postcard after intake to complete entry into the protocol. The postcard’s time and date were logged into a computer and entered into a previously generated list of numbers that randomly assigned participants to treatment and control groups. All numbers and group assignments were generated at the start of the protocol."	Low risk of bias	This study gave no concern for deviation from interventions affecting the outcome	High risk of bias	44/88 lost in the intervention group and 28/80 lost in the control arm. "Imagery rehearsal therapy produces imagery adverse effects; 4 patients reported increased negative imagery and eventually withdrew, and 12 of 66 who completed treatment did not complete follow‐up for unknown reasons."	Low risk of bias	No concerns.	Some concerns	No known pre‐specified analysis plan.	High risk of bias	Adverse outcomes may have been the reason for drop‐out and drop‐out was high.
Littleton 2016	Low risk of bias	"Participants were randomized to the interactive program or psychoeducational website based on a computerized coin flip."	Low risk of bias	Participants and intervention deliverers probably aware of assignment.	High risk of bias	There was a high rate of attrition so some information about adverse events may not have been gathered.	Low risk of bias	There was a high rate of attrition so some information about adverse events may not have been gathered.	Some concerns	There are several ways of expressing treatment drop out and the authors may have selected a perspective that made the result look more favourable.	High risk of bias	The study reports a favourable rate of treatment adherence when in fact only 15.8% of people in the treatment group went through the full program.
Rajan 2020	Low risk of bias	There was a large proportion of individuals excluded post randomisation and prior to allocation which raised some concerns. Also some disrepancy with the trial record for example according to the trial record, 63 were enrolled. However, several strengths in this domain, including: "Preparation of the trial material and randomization was conducted by the independent data and safety monitoring board Karolinska Trial Alliance (KTA) via sequenced computer‐generated simple randomization with 1:1 allocation. Participants received sequentially numbered trial materials with concealed allocation. Allocation envelopes were kept and handled by the trial staff. Due to initial misunderstandings, the prepared sealed envelopes were initially not always picked in strict numeric order. However, the study was monitored and periodically reviewed by the KTA and the misunderstanding was corrected." No differences were reported around baseline characteristics.	Low risk of bias	There was no cause for concern in how the intervention was delivered.	Low risk of bias	"No reportable adverse events occurred." The difficulty with adverse events is only knowing about what gets reported however the domain of assessment was given due consideration by the researchers. "Because this was the first randomized controlled treatment study conducted for the method, we followed the results in order to be able to detect adverse effects (i.e., elevated scores on self‐rating at time point two)."	Low risk of bias	"Since this was the first study of MLI in this context, we were concerned about risk for adverse effects and therefore planned external continuous monitoring of the results."	Low risk of bias	No concerns arose about the selection of the reported result.	Low risk of bias	The brevity of the intervention promoted completion and reduced risk of harm and dropout. Overall, data are a complete and reliable assessment of adverse events.

## Appendices

### Appendix 1. Appendix 1. How the intervention might work

Cognitive behavioural interventions are based on the proposition that behaviours are cognitively mediated (Butler 2006). Mental health and social problems may be influenced by cognitions and resulting behaviours. Because cognitive activity may be monitored and altered, behaviours may be changed through cognitive changes (Dobson 2009). Therefore, interventions that address certain thinking patterns and beliefs may result in positive changes in symptoms, problems and behaviours, which may reduce some of the negative trauma‐related outcomes of rape or sexual assault (Butler 2006). In the case of trauma, theorists believe that the appraisal of fear involves the activation of trauma‐induced schema that lead the survivor to pay attention to information that is consistent with the schema and to ignore evidence that is inconsistent (Resick 1992). This means that benign or ambiguous events can trigger a fear appraisal in trauma survivors (Beck 1985). Hence, cognitive theory, as applied to the process of post‐traumatic stress disorder (PTSD) (Veronen 1983), focuses on two processes: 1) changing a person’s cognitive appraisal of the traumatic event, or changing the process by which an individual attaches meaning to an event, and 2) changing a person’s attribution of the event. Coping skill treatments are designed to equip survivors with an array of skills to manage their trauma. Some interventions are designed to be delivered within a short period of time following the assault or rape (less than three months), whereas others are used for survivors over the longer term. The former is an attempt to provide prophylactic treatment to prevent chronic problems, while others intend to facilitate faster recovery (Vickerman 2009).

Interventions for sexual assault and rape survivors typically employ a combination of approaches, for example, in the case of stress inoculation therapy, prolonged exposure therapy and cognitive processing therapy, as outlined below.

Stress inoculation therapy (SIT) was adapted (Veronen 1983) from anxiety management procedures (Meichenbaum 1977). It incorporates three elements: 1) behaviourally based psychoeducation so that survivors can understand and normalise fear and avoidance behaviours; 2) guided hierarchical in vivo assignments to target rape‐related phobias (e.g. darkness); and 3) training in six behavioural and cognitive behavioural coping strategies, which are thought‐stopping, guided self‐dialogue, muscle relaxation, controlled breathing, covert modelling and role‐playing. The goal of SIT is to increase the survivor’s awareness of conditioned stimuli to improve early detection of anxiety‐provoking cues, which facilitates the use of coping skills early in the stress response to reduce anxiety (Sherman 1998).

Prolonged exposure therapy (PET) was developed from earlier treatments using flooding exposure techniques and emotion processing theory with patients with anxiety disorders (Foa 1986). These techniques were extended by Foa and colleagues (Foa 1986; Foa 1994), who argued that it is the encoding of memories under extreme distress that leads to disjointed and disorganised memories, which impede natural recovery and lead to PTSD. The aim of PET is to decrease the anxiety associated with rape memories. PET begins with psychoeducation, breathing training and the development of a fear and avoidance hierarchy for in vivo exposures. In therapy, people are asked to relive the rape scene and describe it aloud as they are imagining it, using present tense and vivid detail, which may be done several times in one therapy session. The survivor’s narrative is recorded, and daily homework requires them to listen to their recorded account for further exposure (Foa 1991).

Cognitive processing therapy (CPT) was developed from emotional processing theory to identify a rape survivor’s 'stuck points', which are the parts of the traumatic narratives that cause them the greatest conflict (Resick 1992; Resick 1993). These are manifestations of unsuccessful attempts to accommodate information in relation to the trauma into pre‐existing memory and belief structures. The goal of CPT is to help individuals to integrate their trauma into pre‐existing schemas, to decrease avoidance and intrusions of unintegrated aspects of the trauma. Unlike PET, CPT seeks to directly correct misconceptions or misinformation about trauma (for example, 'I’m not safe anywhere' or 'I can't trust anyone'). CPT also includes psychoeducation, exposure and cognitive methods. Exposure is achieved via the individual writing accounts of the rape and its meaning, which they re‐read between sessions, and writing about the impact of the trauma multiple times, in order to incorporate new understandings and evaluation. Therapy then addresses one of five themes (safety, trust, power and control, esteem and intimacy) in each of the last five sessions, via the use of cognitive‐restructuring worksheets, Socratic questioning and discussion.

Behavioural therapies are based on the premise that all behaviours are learnt and, therefore, that unhealthy behaviours can be changed. Techniques such as systematic desensitisation and flooding are often used with this population, which emphasise the importance of extinguishing anxiety and reducing avoidant behaviours. For example, Foa and colleagues believe that exposure to the trauma allows mistaken evaluations and faulty stimulus‐response associations to be corrected (Foa 1986; Foa 1994). Survivors are taught to replace a fear response with relaxation responses. This can be done gradually, with systematic desensitisation, or more quickly via flooding.

Eye movement desensitisation and reprocessing (EMDR) was developed by Shapiro 1995 for the treatment of PTSD. It involves exposure elements and cognitive techniques. In EMDR, a scene is used to represent the entire trauma. The survivor imagines the scene and recites words related to the scene, while the therapist moves their fingers back and forth in front of the survivor, so that the survivor performs rhythmic, multi saccadic eye movements (quick, simultaneous movements of both eyes between two or more phases of fixation in the same direction) by watching the therapist’s fingers. This movement is argued to facilitate the processing of trauma memory through the dual attention required to focus on attending to the therapist’s finger movement (external stimulus) and the trauma scene (internal stimulus). When the survivor’s anxiety to the scene has decreased, a new adaptive belief is rehearsed until this new belief feels true (Rothbaum 1997). EMDR is similar to the behavioural techniques of flooding and systematic desensitisation (Boudewyns 1996a), and studies comparing EMDR with and without eye movements suggest that EMDR without eye movements leads to equivalent outcomes as EDMR with eye movements (Boudewyns 1996b; Pitman 1996).

Third‐wave cognitive behavioural therapies (e.g. acceptance and commitment therapy and mindfulness) act on changing the function of psychological events and the individuals' relationship to them through acceptance, being present and committed action (Hayes 2006).

Counselling encompasses a range of interventions that may be employed by, for example, rape crisis centres (Cryer 1980; Foa 1991; Resick 1988). Counselling can be premised on a number of approaches (e.g. humanist, psychodynamic) and may be delivered as an intervention in itself or in combination with other approaches. Counselling is likely to be very individually focused in order to discuss issues raised by the survivor, and the necessary variation makes it difficult to specify exactly what is included in each session.

Humanistic and supportive therapies include an eclectic mix of therapeutic techniques. Supportive therapy is almost always non‐directive, that is, the survivor is empowered to guide the content and the therapist avoids offering direct advice (Cohen 2005; Deblinger 2001). The focus is on developing a supportive relationship between the therapist and participant (Cohen 2005). Supportive therapy can be conducted in either an individual or group format.

Other psychologically orientated interventions include a diverse range of therapies that aim to help survivors cope with, express and work through trauma through, for example, expressive writing (Harte 2013) or mindfulness (Brotto 2012). For instance, equine‐assisted therapy for anxiety and post‐traumatic stress symptoms has been shown to reduce symptoms of post‐traumatic stress, severe emotional responses to trauma, generalised anxiety, symptoms of depression and alcohol use, as well as increasing the use of mindfulness strategies (Earles 2015).

Psychosocial interventions include a wide range of interventions that target interpersonal, social and environmental factors that relate to recovery from the trauma of rape and sexual assault in addition to, or instead of, the individual factors that are the focus of psychological therapies. The way in which the interventions might work will be dependent on the factors that are targeted. Psychoeducation elements aim to provide information, modelling and training, for example, to explain maladaptive and adaptive coping strategies and to encourage the use of the latter (e.g. see Sikkema 2018). Group programmes and the provision of advisors or mentors provide social support; these may be important given the stigma and shame associated with rape and sexual assault and which can lead to social isolation. Psychosocial interventions may increase self‐esteem (Sikkema 2018) and provide practical assistance and emotional support (Home Office 2017).

### Appendix 2. Search strategies

#### Cochrane Central Register of Controlled Trials (CENTRAL)

Searched 5 July 2019 (1494 records) Searched 8 March 2021 (439 records) Searched 10 January 2022 (160 records) #1 [mh ^"sex offenses"] #2 [mh Incest] #3 [mh "intimate partner violence"] #4 [mh "human trafficking"] #5 [mh rape] #6 [mh "spouse abuse"] #7 (sex* NEAR/5 (abuse* or assaul* or attack* or aggress* or coer* or exploit* or force* or molest* or offen* or traffick* or trauma* or unlawful* or unwanted or violen*)):ti,ab,kw #8 (intercourse NEAR/5 (coer* or force* or unwanted)):ti,ab,kw #9 ("intimate partner violence" or "intimate partner abuse"):ti,ab,kw #10 (rape or raped or incest*):ti,ab,kw #11 (sex* NEAR/3 (victim* or revictim* or re‐victim* or survivor*)):ti,ab,kw #12 {or #1‐#11} #13 MeSH descriptor: [Anxiety Disorders] explode all trees and with qualifier(s): [therapy ‐ TH] #14 [mh "Adaptation, Psychological"] #15 [mh "Behavior Therapy"] #16 [mh "Combined Modality Therapy"] #17 [mh ^"community networks"] #18 [mh "Complementary therapies"] #19 [mh Counseling] #20 MeSH descriptor: [Depression] this term only and with qualifier(s): [therapy ‐ TH] #21 MeSH descriptor: [Depressive Disorder, Major] explode all trees and with qualifier(s): [therapy ‐ TH] #22 [mh Exercise] #23 [mh "Exercise therapy"] #24 [mh "Health Education"] #25 [mh "Health Knowledge, Attitudes, Practice"] #26 [mh "Interview, Psychological"] #27 [mh "mind body therapies"] #28 [mh "Psychological adjustment"] #29 MeSH descriptor: [Psychological Trauma] explode all trees and with qualifier(s): [prevention & control ‐ PC, rehabilitation ‐ RH, therapy ‐ TH] #30 [mh "psychosocial support systems"] #31 [mh "psychotherapy"] #32 [mh "Referral and Consultation"] #33 [mh "Self‐Help Groups"] #34 [mh "Social Support"] #35 MeSH descriptor: [Stress Disorders, Post‐Traumatic] explode all trees and with qualifier(s): [prevention & control ‐ PC, rehabilitation ‐ RH, therapy ‐ TH] #36 MeSH descriptor: [Video Recording] this term only #37 MeSH descriptor: [Videotape Recording] this term only #38 [mh Writing] #39 ((abreaction or desensitization or exposure or implosive) NEAR/3 therap*):ti,ab,kw #40 "acceptance and commitment therapy":ti,ab,kw #41 (advisor* or advocate* or advocacy):ti,ab,kw #42 ((animal* or art or colo?r or creative* or dance or dancing or drama or equine or experiential or music or narrative or play* or sensory or singing) NEAR/3 (program* or intervention* or therap*)):ti,ab,kw #43 (autogenic or autosuggestion* or auto next suggestion* or breathing next exercise* or hypnosis or hypno next therapy or hypnotherapy):ti,ab,kw #44 behavio* next activation:ti,ab,kw #45 (behavio* NEAR/3 (intervention* or program* or therap* or training or treatment*)):ti,ab,kw #46 ((biofeedback or feedback or imagery) NEAR/3 (intervention* or therap* or train* or treatment*)):ti,ab,kw #47 ((brief or combination or compass* next focus* or integrated or integrative or time next limited) NEAR/3 (intervention* or therap* or treatment*)):ti,ab,kw #48 ((client next focus* or non next direct* or nondirect* or solution next focus* or trauma* or talking) NEAR/3 therap*) #49 (cognitiv* or cognition):ti,ab,kw #50 CBT:ti,ab,kw #51 ((cope or coping) NEAR/1 (intervention* or mechanism* or skill* or technique*)):ti,ab,kw #52 counsel*ing:ti,ab,kw #53 ((couple* or family or group or systemic* or multimodal* or multi next modal*) NEAR/3 (program* or intervention* or therap* or treat*)):ti,ab,kw #54 dialectical next behavio*r* next therap*:ti,ab,kw #55 (exercise* or physical next training):ti,ab,kw #56 ((existential or gestalt or humanistic or interpersonal or milieu or person‐centred or residential or socioenvironmental or socio next environmental) NEXT therap*):ti,ab,kw #57 expressive next writing:ti,ab,kw #58 ("Eye Movement Desensitization and Reprocessing" or EMDR):ti,ab,kw #59 (meditat* or mental next training or mindfulness* or mind next training or brain next training or yoga):ti,ab,kw #60 motivational next interview*:ti,ab,kw #61 (reality next therap* or problem next solving):ti,ab,kw #62 (psycho* next therap* or psychotherap*) #63 (psychoanalytic* or psycho next analytic* or psychodynamic* or psycho next dynamic*):ti,ab,kw #64 (psychodrama or psycho next drama or acting next out or role next play):ti,ab,kw #65 (psychosocial or psycho next social or psychoeducation* or psycho next education*):ti,ab,kw #66 rational next emotive:ti,ab,kw #67 (Relax* NEAR/3 (training* or treatment* or therap*)):ti,ab,kw #68 (Service* NEAR/3 (refer* or use*)):ti,ab,kw #69 (stress next inoculation next training or SIT or prolonged next exposure next therapy or PET or cognitive next processing next therapy or CPT):ti,ab,kw #70 ((support or advice or advisor*) NEAR/1 (centre* or center* or community or group* or network* or social or staff*)):ti,ab,kw #71 (therapeutic next allianc* or therapeutic next relationship* or therapeutic next communit*):ti,ab,kw #72 Third next wave:ti,ab,kw #73 {or #13‐#72} #74 #12 and #73 #75 (rape near/1 (centre* or center* or service* or support)):ti,ab,kw #76 ((sex* next assault NEAR/3 centre) or (sex* next assault NEAR/3 center) or (sex* next assault NEAR/3 service) or (sex* next assault NEAR/3 support)):ti,ab,kw #77 ((sex* next abuse* NEAR/3 centre) or (sex* next abuse* NEAR/3 center) or (sex* next abuse* NEAR/3 service) or (sex* next abuse* NEAR/3 support)):ti,ab,kw #78 {or #74‐#77}  #79 (IRCT* OR RBR* OR JPRN* OR TCTR* OR SLCTR* OR CTRI* OR EUCT* OR NCT* OR ISRCTN* OR ACTRN* OR DRKS* OR PACT*):AU #80 #78 and #7 #81 #78 not #80 (Note: final line 2019. Trial registry records were excluded from the CENTRAL search. A separate search for trials was conducted via trials registry websites)  #82 #78 in Trials Limited by Added between 05/07/2019 and 08/03/2021 (Note: final line 2021) #83 #78 in Trials Limited by Added between 08/03/2021 and 10/01/2022 (Note: final line 2022)

#### CCMDCTR‐Studies register

Searched 2 July 2019 (308 records) Not searched in 2021 as no further records had been added

The CCMDCTR‐Studies register was searched using the following controlled vocabulary terms:

("sexual abuse" or "sexual assault victim" or rape):SCM,SCO,STC AND (adolescent or adult or aged or "not stated" or unclear):XAGE AND STUDY:CRSTYPE AND INREGISTER n=14 studies (24 refs) [Key to study field codes. SCO: Health Care Condition; SCM: Co‐morbid Health Care Condition; STC: Target Condition; XAGE: Age of participants]

The CCMDCTR‐References register was also searched using a more sensitive set of keywords (all fields) to find additional uncoded/untagged references:

(rape or raped or (sex* adj3 (abuse* or assault* or victim* or violen*))) not (sex* adj3 (child or children)):ti n=304

Total from both searches = 328 Duplicates removed= 20 Records to screen = 308

#### Ovid MEDLINE(R)

Searched 2 July 2019 (2699 records) Searched 8 March 2021 (322 records) Searched 10 January 2022 (244 records)

1 sex offenses/  2 Incest/  3 intimate partner violence/  4 human trafficking/ 5 rape/  6 spouse abuse/  7 (sex$ adj5 (abuse$ or assaul$ or attack$ or aggress$ or coer$ or exploit$ or force$ or molest$ or offen$ or traffick$ or trauma$ or unlawful$ or unwanted or violen$)).tw,kf.  8 (intercourse adj5 (coer$ or force$ or unwanted)).tw,kf.  9 intimate partner violence.tw,kf. 10 (rape or raped or incest$).tw,kf. 11 (sex$ adj3 (victim$ or revictim$ or re‐victim$ or survivor$)).tw,kf.  12 or/1‐11  13 Anxiety/th  14 Anxiety Disorders/th  15 Adaptation, Psychological/  16 exp Behavior Therapy/ 17 Combined Modality Therapy/ 18 community networks/  19 exp Complementary therapies/ 20 exp Counseling/  21 Depression/th  22 Depressive Disorder/th ) 23 Depressive Disorder, Major/th  24 Exercise/  25 Exercise therapy/  26 Health Education/  27 Health Knowledge, Attitudes, Practice/  28 Interview, Psychological/  29 exp mind body therapies/ 30 Psychological adjustment/  31 Psychological Trauma/pc, rh, th  32 psychosocial support systems/  33 exp psychotherapy/  34 "Referral and Consultation"/  35 Self‐Help Groups/  36 Social Support/  37 Stress Disorders, Post‐Traumatic/pc, rh, th  38 video recording/ or videotape recording/  39 Writing/  40 ((abreaction or desensitization or exposure or implosive) adj3 therap$).tw,kf.  41 "acceptance and commitment therapy".tw,kf. 42 (advisor$ or advocate$ or advocacy).tw,kf. 43 ((animal$ or art or colo?r or creative$ or dance or dancing or drama or equine or experiential or music or narrative or play$ or sensory or singing) adj3 (program$ or intervention$ or therap$)).tw,kf.  44 (autogenic or autosuggestion$ or auto‐suggestion$ or breathing exercise$ or hypnosis or hypno‐therapy or hypnotherapy).tw,kf.  45 behavio$ activation.tw,kf.  46 (behavio?r$ adj3 (intervention$ or program$ or therap$ or training or treatment$)).tw,kf.  47 ((biofeedback or feedback or imagery) adj3 (intervention$ or therap$ or train$ or treatment$)).tw,kf.  48 ((brief or combination or compass$ focus$ or integrated or integrative or time‐limited) adj3 (intervention$ or therap$ or treatment$)).tw,kf.  49 ((client focus$ or non‐direct$ or nondirect$ or solution focus$ or trauma$ or talking) adj3 therap$).tw,kf. 50 (cognitiv$ or cognition).tw,kf.  51 CBT.tw,kf.  52 ((cope or coping) adj1 (intervention$ or mechanism$ or skill$ or technique$)).tw,kf.  53 counsel?ing.tw,kf.  54 ((couple$ or family or group or systemic$ or multimodal$ or multi‐modal$) adj3 (program$ or intervention$ or therap$ or treat$)).tw,kf.  55 dialectical behavio?r$ therap$.tw,kf. 56 (exercise$ or physical training).tw,kf.  57 ((existential or gestalt or humanistic or interpersonal or milieu or person‐centred or residential or socioenvironmental or socio‐environmental) adj therap$).tw,kf.  58 expressive writing.tw,kf.  59 ("Eye Movement Desensitization and Reprocessing" or EMDR).tw,kf.  60 (meditat$ or mental training or mindfulness$ or mind training or brain training or yoga).tw,kf.  61 motivational interview$.tw,kf.  62 (reality therap$ or problem solving).tw,kf. 63 (psycho$ therap$ or psychotherap$).tw,kf.  64 (psychoanalytic$ or psycho‐analytic$ or psychodynamic$ or psycho‐dynamic$).tw,kf.  65 (psychodrama or psycho‐drama or acting out or role play).tw,kf.  66 (psychosocial or psycho‐social or psychoeducation$ or psycho‐education$).tw,kf.  67 rational emotive.tw,kf.  68 (Relax$ adj3 (training$ or treatment$ or therap$)).tw,kf.  69 (Service$ adj3 (refer$ or use$)).tw,kf. 70 (stress inoculation training or SIT or prolonged exposure therapy or PET or cognitive processing therapy or CPT).tw,kf.  71 ((support or advice or advis$1) adj1 (centre$1 or center$1 or community or group$ or network$ or social or staff$)).tw,kf.  72 (therapeutic allianc$ or therapeutic relationship$ or therapeutic communit$).tw,kf.  73 Third wave.tw,kf.  74 or/13‐73  75 12 and 74 76 (rape adj3 (centre$ or center$ or service$ or support)).tw,kf.  77 ((sex$ assault adj3 centre) or (sex$ assault adj3 center) or (sex$ assault adj3 service) or (sex$ assault adj3 support)).tw,kf.  78 ((sex$ abuse$ adj3 centre) or (sex$ abuse$ adj3 center) or (sex$ abuse$ adj3 service) or (sex$ abuse$ adj3 support)).tw,kf.  79 or/76‐78  80 75 or 79  81 randomized controlled trial.pt.  82 controlled clinical trial.pt.  83 randomi#ed.ab.  84 placebo$.ab.  85 drug therapy.fs. 86 randomly.ab.  87 trial.ab.  88 groups.ab.  89 or/81‐88  90 exp animals/ not humans.sh.  91 89 not 90  92 80 and 91  93 (201907* or 201908* or 201909* or 201910* or 201911* or 201912* or 2020* or 2021*).dt,ez,da.  94 92 and 93 95 (202103* or 202104* or 202105* or 202106* or 202107* or 202108* or 202109* or 202110* or 202111* or 202112* or 2022*).dt,ez,da.  96 92 and 95

Ovid MEDLINE In‐process and other nonindexed citations

Searched 2 July 2019 (490 records) Searched 8 March 2021 (252 records) Searched 10 January 2022 (46 new records)

1 (sex$ adj5 (abuse$ or assaul$ or attack$ or aggress$ or coer$ or exploit$ or force$ or molest$ or offen$ or traffick$ or trauma$ or unlawful$ or unwanted or violen$)).tw,kf.  2 (intercourse adj5 (coer$ or force$ or unwanted)).tw,kf.  3 intimate partner violence.tw,kf. 4 (rape or raped or incest$).tw,kf.  5 (sex$ adj3 (victim$ or revictim$ or re‐victim$ or survivor$)).tw,kf. 6 or/1‐5  7 therapy.ti.  8 ((abreaction or desensitization or exposure or implosive) adj3 therap$).ab,kf.  9 "acceptance and commitment therapy".ab,kf.  10 (advisor$ or advocate$ or advocacy).tw,kf.  11 ((animal$ or art or colo?r or creative$ or dance or dancing or drama or equine or experiential or music or narrative or play$ or sensory or singing) adj3 (program$ or intervention$ or therap$)).tw,kf.  12 (autogenic or autosuggestion$ or auto‐suggestion$ or breathing exercise$ or hypnosis or hypno‐therapy or hypnotherapy).tw,kf.  13 behavio$ activation.tw,kf.  14 (behavio?r$ adj3 (intervention$ or program$ or therap$ or training or treatment $)).tw,kf.  15 ((biofeedback or feedback or imagery) adj3 (intervention$ or therap$ or train$ or treatment$)).tw,kf.  16 ((brief or combination or compass$ focus$ or integrated or integrative or time‐ limited) adj3 (intervention$ or therap$ or treatment$)).tw,kf.  17 ((client focus$ or non‐direct$ or nondirect$ or solution focus$ or trauma$ or talking) adj3 therap$).tw,kf.  18 (cognitiv$ or cognition or CBT).tw,kf.  19 ((cope or coping) adj1 (intervention$ or mechanism$ or skill$ or technique $)).tw,kf. 20 counsel?ing.tw,kf.  21 ((couple$ or family or group or systemic$ or multimodal$ or multi‐modal$) adj3 (program$ or intervention$ or therap$ or treat$)).tw,kf.  22 dialectical behavio?r$ therap$.tw,kf. 23 (exercise$ or physical training).tw,kf. 24 ((existential or gestalt or humanistic or interpersonal or milieu or person‐ centred or residential or socioenvironmental or socio‐environmental) adj therap$).tw,kf.  25 expressive writing.tw,kf. 26 ("Eye Movement Desensitization and Reprocessing" or EMDR).tw,kf. 27 (meditat$ or mental training or mindfulness$ or mind training or brain training or yoga).tw,kf. 28 motivational interview$.tw,kf.  29 (reality therap$ or problem solving).tw,kf.  30 (psycho$ therap$ or psychotherap$).tw,kf.  31 (psychoanalytic$ or psycho‐analytic$ or psychodynamic$ or psycho‐dynamic$).tw,kf.  32 (psychodrama or psycho‐drama or acting out or role play).tw,kf.  33 (psychosocial or psycho‐social or psychoeducation$ or psycho‐education$).tw,kf.  34 rational emotive.tw,kf.  35 (Relax$ adj3 (training$ or treatment$ or therap$)).tw,kf.  36 (Service$ adj3 (refer$ or use$)).tw,kf.  37 (stress inoculation training or SIT or prolonged exposure therapy or PET or cognitive processing therapy or CPT).tw,kf.  38 ((support or advice or advis$1) adj1 (centre$1 or center$1 or community or group$ or network$ or social or staff$)).tw,kf.  39 (therapeutic allianc$ or therapeutic relationship$ or therapeutic communit$).tw,kf. 40 Third wave.tw,kf. 41 or/7‐40  42 6 and 41  43 (rape adj3 (centre$ or center$ or service$ or support)).tw,kf.  44 ((sex$ assault adj3 centre) or (sex$ assault adj3 center) or (sex$ assault adj3 service) or (sex$ assault adj3 support)).tw,kf.  45 ((sex$ abuse$ adj3 centre) or (sex$ abuse$ adj3 center) or (sex$ abuse$ adj3 service) or (sex$ abuse$ adj3 support)).tw,kf.  46 or/42‐45 47 (random$ or control$ or group$ or cluster$ or placebo$ or trial$ or assign$ or prospectiv$ or meta‐analysis or systematic review or longitudinal$).tw,kf.  48 46 and 47  49 limit 48 to ("in data review" or in process or "pubmed not medline")

#### Ovid MEDLINE Epub Ahead of Print

Searched 2 July 2019 (247 records) Searched 8 March 2021 (279 records) Searched 10 January 2022 (229 records)

1 (sex$ adj5 (abuse$ or assaul$ or attack$ or aggress$ or coer$ or exploit$ or force$ or molest$ or offen$ or traffick$ or trauma$ or unlawful$ or unwanted or violen$)).tw,kf.  2 (intercourse adj5 (coer$ or force$ or unwanted)).tw,kf.  3 intimate partner violence.tw,kf. 4 (rape or raped or incest$).tw,kf.  5 (sex$ adj3 (victim$ or revictim$ or re‐victim$ or survivor$)).tw,kf. 6 or/1‐5  7 therapy.ti.  8 ((abreaction or desensitization or exposure or implosive) adj3 therap$).ab,kf.  9 "acceptance and commitment therapy".ab,kf.  10 (advisor$ or advocate$ or advocacy).tw,kf.  11 ((animal$ or art or colo?r or creative$ or dance or dancing or drama or equine or experiential or music or narrative or play$ or sensory or singing) adj3 (program$ or intervention$ or therap$)).tw,kf.  12 (autogenic or autosuggestion$ or auto‐suggestion$ or breathing exercise$ or hypnosis or hypno‐therapy or hypnotherapy).tw,kf.  13 behavio$ activation.tw,kf.  14 (behavio?r$ adj3 (intervention$ or program$ or therap$ or training or treatment $)).tw,kf.  15 ((biofeedback or feedback or imagery) adj3 (intervention$ or therap$ or train$ or treatment$)).tw,kf.  16 ((brief or combination or compass$ focus$ or integrated or integrative or time‐ limited) adj3 (intervention$ or therap$ or treatment$)).tw,kf.  17 ((client focus$ or non‐direct$ or nondirect$ or solution focus$ or trauma$ or talking) adj3 therap$).tw,kf.  18 (cognitiv$ or cognition or CBT).tw,kf.  19 ((cope or coping) adj1 (intervention$ or mechanism$ or skill$ or technique $)).tw,kf. 20 counsel?ing.tw,kf.  21 ((couple$ or family or group or systemic$ or multimodal$ or multi‐modal$) adj3 (program$ or intervention$ or therap$ or treat$)).tw,kf.  22 dialectical behavio?r$ therap$.tw,kf. 23 (exercise$ or physical training).tw,kf. 24 ((existential or gestalt or humanistic or interpersonal or milieu or person‐ centred or residential or socioenvironmental or socio‐environmental) adj therap$).tw,kf.  25 expressive writing.tw,kf. 26 ("Eye Movement Desensitization and Reprocessing" or EMDR).tw,kf. 27 (meditat$ or mental training or mindfulness$ or mind training or brain training or yoga).tw,kf. 28 motivational interview$.tw,kf.  29 (reality therap$ or problem solving).tw,kf.  30 (psycho$ therap$ or psychotherap$).tw,kf.  31 (psychoanalytic$ or psycho‐analytic$ or psychodynamic$ or psycho‐dynamic$).tw,kf.  32 (psychodrama or psycho‐drama or acting out or role play).tw,kf.  33 (psychosocial or psycho‐social or psychoeducation$ or psycho‐education$).tw,kf.  34 rational emotive.tw,kf.  35 (Relax$ adj3 (training$ or treatment$ or therap$)).tw,kf.  36 (Service$ adj3 (refer$ or use$)).tw,kf.  37 (stress inoculation training or SIT or prolonged exposure therapy or PET or cognitive processing therapy or CPT).tw,kf.  38 ((support or advice or advis$1) adj1 (centre$1 or center$1 or community or group$ or network$ or social or staff$)).tw,kf.  39 (therapeutic allianc$ or therapeutic relationship$ or therapeutic communit$).tw,kf. 40 Third wave.tw,kf. 41 or/7‐40  42 6 and 41  43 (rape adj3 (centre$ or center$ or service$ or support)).tw,kf.  44 ((sex$ assault adj3 centre) or (sex$ assault adj3 center) or (sex$ assault adj3 service) or (sex$ assault adj3 support)).tw,kf.  45 ((sex$ abuse$ adj3 centre) or (sex$ abuse$ adj3 center) or (sex$ abuse$ adj3 service) or (sex$ abuse$ adj3 support)).tw,kf.  46 or/42‐45 47 (random$ or control$ or group$ or cluster$ or placebo$ or trial$ or assign$ or prospectiv$ or meta‐analysis or systematic review or longitudinal$).tw,kf.  48 46 and 47

#### Embase Ovid

Searched 2 July 2019 (2335 records) Searched 8 March 2021 (878 records) Searched 10 January 2022 (515 records)

1 sexual assault/  2 sexual abuse/  3 sexual violence/  4 sexual coercion/  5 sexual exploitation/  6 incest/  7 partner violence/  8 human trafficking/  9 exp rape/  10 (sex$ adj5 (abuse$ or assaul$ or attack$ or aggress$ or coer$ or exploit$ or force$ or molest$ or offen$ or traffick$ or trauma$ or unlawful$ or unwanted or violen$)).tw,kw.  11 (intercourse adj5 (coer$ or force$ or unwanted)).tw,kw.  12 intimate partner violence.tw,kw.  13 (rape or raped or incest$).tw,kw. 14 (sex$ adj3 (victim$ or revictim$ or re‐victim$ or survivor$)).tw,kw.  15 or/1‐14  16 adaptive behavior/  17 exp alternative medicine/  18 anxiety/th [Therapy]  19 exp anxiety disorder/th [Therapy]  20 exp behavior therapy/  21 community care/  22 exp coping behavior/  23 exp counseling/  24 exp depression/th [Therapy]  25 exp exercise/  26 exp kinesiotherapy/ 27 health education/ or psychoeducation/  28 health education/  29 psychoeducation/  30 psychological interview/  31 Psychological adjustment/  32 psychotrauma/rh, th 33 psychosocial care/  34 exp psychotherapy/  35 patient referral/  36 self help/ or exp self care/  37 social support/  38 exp videorecording/ 39 writing/  40 ((abreaction or desensitization or exposure or implosive) adj3 therap$).tw,kw.  41 "acceptance and commitment therapy".tw,kw. 42 (advisor$ or advocate$ or advocacy).tw,kw. 43 ((animal$ or art or colo?r or creative$ or dance or dancing or drama or equine or experiential or music or narrative or play$ or sensory or singing) adj3 (program$ or intervention$ or therap$)).tw,kw.  44 (autogenic or autosuggestion$ or auto‐suggestion$ or breathing exercise$ or hypnosis or hypno‐therapy or hypnotherapy).tw,kw.  45 behavio$ activation.tw,kw.  46 (behavio?r$ adj3 (intervention$ or program$ or therap$ or training or treatment $)).tw,kw.  47 ((biofeedback or feedback or imagery) adj3 (intervention$ or therap$ or train$ or treatment$)).tw,kw.  48 ((brief or combination or compass$ focus$ or integrated or integrative or time‐ limited) adj3 (intervention$ or therap$ or treatment$)).tw,kw.  49 ((client focus$ or non‐direct$ or nondirect$ or solution focus$ or trauma$ or talking) adj3 therap$).tw,kw. 50 (cognitiv$ or cognition).tw,kw. 51 CBT.tw,kw. 52 ((cope or coping) adj1 (intervention$ or mechanism$ or skill$ or technique $)).tw,kw. 53 counsel?ing.tw,kw. 54 ((couple$ or family or group or systemic$ or multimodal$ or multi‐modal$) adj3 (program$ or intervention$ or therap$ or treat$)).tw,kw.  55 dialectical behavio?r$ therap$.tw,kw.  56 (exercise$ or physical training).tw,kw. 57 ((existential or gestalt or humanistic or interpersonal or milieu or person‐ centred or residential or socioenvironmental or socio‐environmental) adj therap$).tw,kw.  58 expressive writing.tw,kw.  59 ("Eye Movement Desensitization and Reprocessing" or EMDR).tw,kw.  60 (meditat$ or mental training or mindfulness$ or mind training or brain training or yoga).tw,kw 61 motivational interview$.tw,kw. 62 (reality therap$ or problem solving).tw,kw. 63 (psycho$ therap$ or psychotherap$).tw,kw. 64 (psychoanalytic$ or psycho‐analytic$ or psychodynamic$ or psycho‐dynamic$).tw,kw. 65 (psychodrama or psycho‐drama or acting out or role play).tw,kw.  66 (psychosocial or psycho‐social or psychoeducation$ or psycho‐education$).tw,kw. 67 rational emotive.tw,kw. 68 (Relax$ adj3 (training$ or treatment$ or therap$)).tw,kw.  69 (Service$ adj3 (refer$ or use$)).tw,kw.  70 (stress inoculation training or SIT or prolonged exposure therapy or PET or cognitive processing therapy or CPT).tw,kw.  71 ((support or advice or advis$1) adj1 (centre$1 or center$1 or community or group$ or network$ or social or staff$)).tw,kw. 72 (therapeutic allianc$ or therapeutic relationship$ or therapeutic communit $).tw,kw.  73 Third wave.tw,kw.  74 or/16‐73  75 15 and 74  76 (rape adj3 (centre$ or center$ or service$ or support)).tw,kw.  77 ((sex$ assault adj3 centre) or (sex$ assault adj3 center) or (sex$ assault adj3 service) or (sex$ assault adj3 support)).tw,kw. 78 ((sex$ abuse$ adj3 centre) or (sex$ abuse$ adj3 center) or (sex$ abuse$ adj3 service) or (sex$ abuse$ adj3 support)).tw,kw.  79 or/76‐78  80 75 or 79  81 Randomized controlled trial/  82 Controlled clinical study/  83 random$.ti,ab.  84 randomization/  85 intermethod comparison/  86 placebo.ti,ab.  87 (compare or compared or comparison).ti.  88 ((evaluated or evaluate or evaluating or assessed or assess) and (compare or compared or comparing or comparison)).ab. 89 (open adj label).ti,ab.  90 ((double or single or doubly or singly) adj (blind or blinded or blindly)).ti,ab. 91 double blind procedure/  92 parallel group$1.ti,ab.  93 (crossover or cross over).ti,ab.  94 ((assign$ or match or matched or allocation) adj5 (alternate or group$1 or intervention$1 or patient$1 or subject$1 or participant$1)).ti,ab.  95 (assigned or allocated).ti,ab.  96 (controlled adj7 (study or design or trial)).ti,ab.  97 (volunteer or volunteers).ti,ab.  98 human experiment/ 99 trial.ti.  100 or/81‐99 101 (random$ adj sampl$ adj7 ("cross section$" or questionnaire$1 or survey$ or database$1)).ti,ab. not (comparative study/ or controlled study/ or randomi?ed controlled.ti,ab. or randomly assigned.ti,ab.) (8480) 102 Cross‐sectional study/ not (randomized controlled trial/ or controlled clinical study/ or controlled study/ or randomi?ed controlled.ti,ab. or control group$1.ti,ab.) (262553) 103 (((case adj control$) and random$) not randomi?ed controlled).ti,ab.  104 (Systematic review not (trial or study)).ti.  105 (nonrandom$ not random$).ti,ab.  106 "Random field$".ti,ab.  107 (random cluster adj3 sampl$).ti,ab. 108 (review.ab. and review.pt.) not trial.ti. (873499) 109 "we searched".ab. and (review.ti. or review.pt.)  110 "update review".ab.  111 (databases adj4 searched).ab. 112 (rat or rats or mouse or mice or swine or porcine or murine or sheep or lambs or pigs or piglets or rabbit or rabbits or cat or cats or dog or dogs or cattle or bovine or monkey or monkeys or trout or marmoset$1).ti. and animal experiment/  113 Animal experiment/ not (human experiment/ or human/)  114 or/101‐113  115 100 not 114  116 80 and 115 ( Note: Final line 2019) 117 limit 116 to yr="2019 ‐Current"  118 limit 116 to dc=20190701‐20210305  119 117 or 118 ( Note: Final line 2021) 120 limit 116 to dc=20210305‐20220107 121 limit 116 to yr="2021 ‐Current" 122 120 or 121 (Note: Final line 2022)

#### PsycINFO (Ovid)

Searched 3 July 2019 (3087) Searched 8 March 2021 (275 records) Searched 10 January 2022 (145 records)

1 exp sexual abuse/  2 Incest/  3 intimate partner violence/  4 exp Human Trafficking/  5 (sex$ adj5 (abuse$ or assaul$ or attack$ or aggress$ or coer$ or exploit$ or force$ or molest$ or offen$ or traffick$ or trauma$ or unlawful$ or unwanted or violen$)).tw,id.  6 (intercourse adj5 (coer$ or force$ or unwanted)).tw,id.  7 intimate partner violence.tw,id. 8 (rape or raped or incest$).tw,id. 9 (sex$ adj3 (victim$ or revictim$ or re‐victim$ or survivor$)).tw,id. 10 or/1‐9 11 adaptive behavior/  12 exp alternative medicine/  13 exp behavior therapy/  14 biofeedback training/  15 exp community services/  16 exp counseling/  17 exp creative arts therapy/  18 Creative Writing/ or journal writing/  19 exp exercise/  20 movement therapy/  21 exp Health Education/  22 health knowledge/ 23 psychological assessment/ 24 exp Mind Body Therapy/  25 exp Emotional Adjustment/  26 exp psychotherapy/  27 professional referral/ or self‐referral/  28 self‐expression/ 29 exp Self‐Help Techniques/ 30 exp Social Support/  31 exp Support Groups/  32 ((abreaction or desensitization or exposure or implosive) adj3 therap$).tw,id. 33 "acceptance and commitment therapy".tw,id.  34 (behavio?r$ adj3 (intervention$ or program$ or therap$ or training or treatment$)).tw,id.  35 ((biofeedback or feedback or imagery) adj3 (intervention$ or therap$ or train$ or treatment$)).tw,id.  36 ((brief or combination or compass$ focus$ or integrated or integrative or time‐limited) adj3 (intervention$ or therap$ or treatment$)).tw,id.  37 ((client focus$ or non‐direct$ or nondirect$ or solution focus$ or trauma$ or talking) adj3 therap$).tw,id. 38 ((cognitiv$ or cognition) adj5 (therap$ or treatment$)).tw,id. 39 CBT.tw,id. 40 ((cope or coping) adj1 (intervention$ or mechanism$ or skill$ or technique$)).tw,id.  41 counsel?ing.tw,id. 42 ((couple$ or family or group or systemic$ or multimodal$ or multi‐modal$) adj3 (program$ or intervention$ or therap$ or treat$)).tw,id.  43 dialectical behavio?r$ therap$.tw,id.  44 (exercise$ or physical training).tw,id.  45 ((existential or gestalt or humanistic or interpersonal or milieu or person‐centred or residential or socioenvironmental or socio‐environmental) adj therap$).tw,id. 46 expressive writing.tw,id.  47 ("Eye Movement Desensitization and Reprocessing" or EMDR).tw,id. 48 (meditat$ or mental training or mindfulness$ or mind training or brain training or yoga).tw,id.  49 motivational interview$.tw,id.  50 (psycho$ therap$ or psychotherap$).tw,id.  51 (psychoanalytic$ or psycho‐analytic$ or psychodynamic$ or psycho‐dynamic$).tw,id.  52 (psychodrama or psycho‐drama or acting out or role play).tw,id.  53 (psychosocial or psycho‐social or psychoeducation$ or psycho‐education$).tw,id.  54 rational emotive.tw,id.  55 (reality therap$ or problem solving).tw,id.  56 (Relax$ adj3 (training$ or treatment$ or therap$)).tw,id.  57 (Service$ adj3 (refer$ or use$)).tw,id.  58 (stress inoculation training or SIT or prolonged exposure therapy or PET or cognitive processing therapy or CPT).tw,id.  59 ((support or advice or advis$1) adj1 (centre$1 or center$1 or community or group$ or network$ or social or staff$)).tw,id.  60 (therapeutic allianc$ or therapeutic relationship$ or therapeutic communit$).tw,id.  61 Third wave.tw,id.  62 or/11‐61  63 10 and 62  64 (rape adj3 (centre$ or center$ or service$ or support)).tw,id.  65 ((sex$ assault adj3 centre) or (sex$ assault adj3 center) or (sex$ assault adj3 service) or (sex$ assault adj3 support)).tw,id.  66 ((sex$ abuse$ adj3 centre) or (sex$ abuse$ adj3 center) or (sex$ abuse$ adj3 service) or (sex$ abuse$ adj3 support)).tw,id.  67 or/64‐66  68 63 or 67  69 clinical trials/  70 random sampling/ 71 placebo/  72 Experiment controls/  73 (control$ adj3 (study or trial$ or experiment$)).tw,id. 74 ((compar$ or control$ or experiment$ or treat$) adj3 (subjects or group$)).ab,id.  75 ("treat$ as usual" or "usual treatment" or tau or "wait$ list").ab.  76 ((singl$ or doubl$ or trebl$ or tripl$) adj3 (blind$ or mask$)).tw.  77 (random$ or RCT).ti.  78 (randomiz$ or randomis$).ab,id. 79 (assigned or allocated).ab.  80 exp program evaluation/  81 exp treatment outcomes/  82 exp treatment effectiveness evaluation/  83 ((effectiveness or evaluat$) adj3 (stud$ or research$)).tw,id.  84 or/69‐83 85 68 and 84  86 limit 85 to up=20190701‐20210309 (Note: final line 2021) 87 limit 85 to up=20210309‐20220103 (Note: final line 2022)

#### CINAHL Plus (EBSCOhost)

Searched 3 July 2019 (1259 records) Searched 9 March 2021 (226 records) Searched 11 January 2022 (133 records)

S1 (MH "Sexual Abuse")  S2 (MH "Incest")  S3 (MH "Rape")  S4 (MH "Intimate Partner Violence")  S5 (MH "Human Trafficking")  S6 TI(sex* N5 (abuse* or assaul* or attack* or aggress* or coer* or exploit* or force* or molest* or offen* or traffick* or trauma* or unlawful* or unwanted or violen*)) OR AB(sex* N5 (abuse* or assaul* or attack* or aggress* or coer* or exploit* or force* or molest* or offen* or traffick* or trauma* or unlawful* or unwanted or violen*))  S7 TI (intercourse N5 (coer* or force* or unwanted)) OR AB (intercourse N5 (coer* or force* or unwanted))  S8 ti(intimate partner violence) OR AB(intimate partner violence)  S9 TI(rape or raped or incest*) OR AB(rape or raped or incest*) S10 (sex* N3 (victim* or revictim* or re‐victim* or survivor*)) S11 S1 OR S2 OR S3 OR S4 OR S5 OR S6 OR S7 OR S8 OR S9 OR S10  S12 (MH "Anxiety/TH")  S13 (MH "Adaptation, Physiological")  S14 (MH "Combined Modality Therapy")  S15 (MH "Community Mental Health Services")  S16 (MH "Community Networks")  S17 (MH "Alternative Therapies+")  S18 (MH "Counseling+")  S19 (MH "Depression+/TH")  S20 (MH "Exercise+")  S21 (MH "Therapeutic Exercise+")  S22 (MH "Health Education+")  S23 (MH "Mind Body Techniques+")  S24 (MH "Psychological Trauma/TH")  S25 (MH "Psychotherapy+")  S26 (MH "Referral and Consultation")  S27 (MH "Support Groups")  S28 (MH "Support, Psychosocial")  S29 (MH "Stress Disorders, Post‐Traumatic+/TH")  S30 (MH "Videorecording")  S31 (MH "Writing") S32 TI((abreaction or desensitization or exposure or implosive) N3 therap*) OR AB((abreaction or desensitization or exposure or implosive) N3 therap*)  S33 TI (advisor* or advocate* or advocacy) OR AB (advisor* or advocate* or advocacy)  S34 TI ("acceptance and commitment therapy") OR AB("acceptance and commitment therapy")  S35 TI((animal* or art or colo*r or creative* or dance or dancing or drama or equine or experiential or music or narrative or play* or sensory or singing) N3 (program* or intervention* or therap*) OR AB((animal* or art or colo*r or creative* or dance or dancing or drama or equine or experiential or music or narrative or play* or sensory or singing) N3 (program* or intervention* or therap*))  S36 TI(autogenic or autosuggestion* or auto‐suggestion* or "breathing exercise*" or hypnosis or hypno‐therapy or hypnotherapy) OR AB(autogenic or autosuggestion* or auto‐suggestion* or "breathing exercise*" or hypnosis or hypno‐therapy or hypnotherapy)  S37 TI("behavio* activation") OR AB("behavio* activation")  S38 TI(behavio*r* N3 (intervention* or program* or therap* or training or treatment*)) OR AB(behavio*r* N3 (intervention* or program* or therap* or training or treatment*))  S39 TI(((biofeedback or feedback or imagery) N3 (intervention* or therap* or train* or treatment*) or AB(((biofeedback or feedback or imagery) N3 (intervention* or therap* or train* or treatment*)))  S40 TI((brief or combination or "compass* focus*" or integrated or integrative or time‐limited) N3 (intervention* or therap* or treatment*)) OR AB((brief or combination or "compass* focus*" or integrated or integrative or time‐limited) N3 (intervention* or therap* or treatment*))  S41 TI (("client focus*" or non‐direct* or nondirect* or "solution focus*" or trauma* or talking) N3 therap*) OR AB (("client focus*" or non‐direct* or nondirect* or "solution focus*" or trauma* or talking) N3 therap*)  S42 TI(cognitiv* or cognition OR CBT) OR AB(cognitiv* or cognition OR CBT)  S43 TI((cope or coping) N1 (intervention* or mechanism* or skill* or technique*)) OR AB((cope or coping) N1 (intervention* or mechanism* or skill* or technique*))  S44 TI(counsel*ing) OR AB (counsel*ing)  S45 TI((couple* or family or group or systemic* or multimodal* or multi‐modal*) N3 (program* or intervention* or therap* or treat*)) OR AB((couple* or family or group or systemic* or multimodal* or multi‐modal*) N3 (program* or intervention* or therap* or treat*))  S46 TI("dialectical behavio*r* therap*") OR AB("dialectical behavio*r* therap*")  S47 TI(exercise* or "physical training") OR AB(exercise* or "physical training")  S48 TI((existential or gestalt or humanistic or interpersonal or milieu or person‐centred or residential or socioenvironmental or socio‐environmental) N1 therap*) OR AB((existential or gestalt or humanistic or interpersonal or milieu or person‐centred or residential or socioenvironmental or socio‐environmental) N1 therap*)  S49 TI("expressive writing") OR AB("expressive )  S50 TI("Eye Movement Desensitization and Reprocessing" or EMDR) OR AB("Eye Movement Desensitization and Reprocessing" or EMDR)  S51 TI(meditat* or "mental training" or mindfulness* or "mind training" or "brain training" or yoga) OR AB(meditat* or "mental training" or mindfulness* or "mind training" or "brain training" or yoga)  S52 TI("motivational interview") OR AB(" motivational interview")  S53 TI("reality therap*" or "problem solving") OR AB("reality therap*" or "problem solving")  S54 TI ("psycho* therap*" or psychotherap*) OR AB ("psycho* therap*" or psychotherap*)  S55 TI(psychoanalytic* or psycho‐analytic* or psychodynamic* or psycho‐dynamic*) OR AB(psychoanalytic* or psycho‐analytic* or psychodynamic* or psycho‐dynamic*)  S56 TI(psychodrama or psycho‐drama or "acting out" or "role play") OR AB(psychodrama or psycho‐drama or "acting out" or "role play")  S57 TI (psychosocial or psycho‐social or psychoeducation* or psycho‐education*) OR AB (psychosocial or psycho‐social or psychoeducation* or psycho‐education*)  S58 TI("rational emotive") OR AB("rational emotive")  S59 TI(Relax* N3 (training* or treatment* or therap*)) OR AB(Relax* N3 (training* or treatment* or therap*))  S60 TI(Service* N3 (refer* or use*)) OR AB(Service* N3 (refer* or use*))  S61 TI("stress inoculation training" or SIT or "prolonged exposure therapy" or PET or "cognitive processing therapy" or CPT) OR AB("stress inoculation training" or SIT or "prolonged exposure therapy" or PET or "cognitive processing therapy" or CPT)  S62 TI((support or advice or advis*) N1 (centre* or center* or community or group* or network* or social or staff*)) OR AB((support or advice or advis*) N1 (centre* or center* or community or group* or network* or social or staff*))  S63 TI("therapeutic allianc*" or "therapeutic relationship*" or "therapeutic communit*") OR AB("therapeutic allianc*" or "therapeutic relationship*" or "therapeutic communit*")  S64 TI("Third wave") OR AB("Third wave")  S65 S12 OR S13 OR S14 OR S15 OR S16 OR S17 OR S18 OR S19 OR S20 OR S21 OR S22 OR S23 OR S24 OR S25 OR S26 OR S27 OR S28 OR S29 OR S30 OR S31 OR S32 OR S33 OR S34 OR S35 OR S36 OR S37 OR S38 OR S39 OR S40 OR S41 OR S42 OR S43 OR S44 OR S45 OR S46 OR S47 OR S48 OR S49 OR S50 OR S51 OR S52 OR S53 OR S54 OR S55 OR S56 OR S57 OR S58 OR S59 OR S60 OR S61 OR S62 OR S63 OR S64  S66 S11 AND S65  S67 TI(rape N3 (centre* or center* or service* or support)) OR AB(rape N3 (centre* or center* or service* or  S68 TI((sex* assault N3 centre) or (sex* assault N3 center) or (sex* assault N3 service) or (sex* assault N3 support)) OR AB((sex* assault N3 centre) or (sex* assault N3 center) or (sex* assault N3 service) or (sex* assault N3 support))  S69 TI((sex* abuse* N3 centre) or (sex* abuse* N3 center) or (sex* abuse* N3 service) or (sex* abuse* N3 support)) OR AB((sex* abuse* N3 centre) or (sex* abuse* N3 center) or (sex* abuse* N3 service) or (sex* abuse* N3 support))  S70 S67 OR S68 OR S69  S71 S66 OR S70  S72 MH ("Randomized Controlled Trials")  S73 (MH "Double‐Blind Studies")  S74 (MH "Single‐Blind Studies")  S75 (MH "Random Assignment")  S76 (MH "Pretest‐Posttest Design")  S77 MH ("Cluster Sample")  S78 TI (randomised OR randomized)  S79 AB (random*)  S80 TI (trial)  S81 (MH "Sample Size") AND AB (assigned OR allocated OR control)  S82 MH (Placebos)  S83 PT (Randomized Controlled Trial)  S84 AB (control W5 group)  S85 MH ("Crossover Design") OR MH ("Comparative Studies")  S86 AB (cluster W3 RCT)  S87 (MH "Animals+")  S88 MH ("Animal Studies")  S89 TI (animal model*)  S90 S87 OR S88 OR S89  S91 MH ("Human")  S92 S90 NOT S91  S93 S72 OR S73 OR S74 OR S75 OR S76 OR S77 OR S78 OR S79 OR S80 OR S81 OR S82 OR S83 OR S84 OR S85 OR S86  S94 S93 NOT S92  S95 S71 AND S94 [Note: Final line 2019]  S96 EM 20190701‐ S97 S95 AND S96 [Note: Final line 2021]  S98 EM 20210301‐ S99 S95 AND S98 [Note: Final line 2022]

#### ERIC (EBSCOhost)

Searched 3 July 2019 (729 records) Searched 9 March 2021 (83 records) Searched 11 January 2022 (2 records)

S1 DE "Sexual Abuse"  S2 DE "Rape"  S3 DE "Family Violence" AND sex*  S4 TI(sex* N5 (abuse* or assaul* or attack* or aggress* or coer* or exploit* or force* or molest* or offen* or traffick* or trauma* or unlawful* or unwanted or violen*)) OR AB(sex* N5 (abuse* or assaul* or attack* or aggress* or coer* or exploit* or force* or molest* or offen* or traffick* or trauma* or unlawful* or unwanted or violen*))  S5 TI (intercourse N5 (coer* or force* or unwanted)) OR AB (intercourse N5 (coer* or force* or unwanted))  S6 ti(intimate partner violence) OR AB(intimate partner violence)  S7 TI(rape or raped or incest*) OR AB(rape or raped or incest*)  S8 TI(sex* N3 (victim* or revictim* or re‐victim* or survivor*)) OR AB(sex* N3 (victim* or revictim* or re‐victim* or survivor*))  S9 S1 OR S2 OR S3 OR S4 OR S5 OR S6 OR S7 OR S8  S10 (DE "Psychotherapy" OR DE "Milieu Therapy"DE "Relaxation Training")  S11 DE "Relaxation Training") OR (DE "Art Therapy" OR DE "Behavior Modification" OR DE "Contingency Management" OR DE "Desensitization" OR DE "Positive Behavior Supports" OR DE "Bibliotherapy" OR DE "Cognitive Restructuring" OR DE "Counseling" OR DE "Career Counseling" OR DE "Cocounseling" OR DE "Educational Counseling" OR DE "Family Counseling" OR DE "Group Counseling" OR DE "Individual Counseling" OR DE "Marriage Counseling" OR DE "Nondirective Counseling" OR DE "Parent Counseling" OR DE "Peer Counseling" OR DE "Rehabilitation Counseling" OR DE "School Counseling" OR DE "Counseling Psychology" OR DE "Crisis Intervention" OR DE "Emotional Adjustment" OR DE "Group Therapy" OR DE "Hypnosis" OR DE "Individual Psychology" OR DE "Music Therapy" OR DE "Occupational Therapy" OR DE "Rehabilitation" OR DE "Correctional Rehabilitation" OR DE "Drug Rehabilitation" OR DE "Vocational Rehabilitation" OR DE "Role Playing" OR DE "Dramatic Play" OR DE "Therapeutic Environment" OR DE "Therapeutic Recreation" OR DE "Play Therapy") S12 DE "Crisis Intervention"  S13 (DE "Self Help Programs")  S14 DE "Positive Behavior Supports" OR DE "Biofeedback"  S15 TI("support group*") OR AB("support group*") S16 DE "Social Support Groups"  S17 (DE "Health Education")  S18 DE "Video Technology"  S19 TI((abreaction or desensitization or exposure or implosive) N3 therap*) OR AB((abreaction or desensitization or exposure or implosive) N3 therap*)  S20 TI (advisor* or advocate* or advocacy) OR AB (advisor* or advocate* or advocacy)  S21 TI ("acceptance and commitment therapy") OR AB("acceptance and commitment therapy")  S22 TI((animal* or art or colo*r or creative* or dance or dancing or drama or equine or experiential or music or narrative or play* or sensory or singing) N3 (program* or intervention* or therap*) OR AB((animal* or art or colo*r or creative* or dance or dancing or drama or equine or experiential or music or narrative or play* or sensory or singing) N3 (program* or intervention* or therap*))  S23 TI(autogenic or autosuggestion* or auto‐suggestion* or "breathing exercise*" or hypnosis or hypno‐therapy or hypnotherapy) OR AB(autogenic or autosuggestion* or auto‐suggestion* or "breathing exercise*" or hypnosis or hypno‐therapy or hypnotherapy) S37 TI("behavio* activation") OR AB("behavio* activation")  S24 TI(behavio*r* N3 (intervention* or program* or therap* or training or treatment*)) OR AB(behavio*r* N3 (intervention* or program* or therap* or training or treatment*))  S25 TI(((biofeedback or feedback or imagery) N3 (intervention* or therap* or train* or treatment*) or AB(((biofeedback or feedback or imagery) N3 (intervention* or therap* or train* or treatment*)))  S26 TI((brief or combination or "compass* focus*" or integrated or integrative or time‐limited) N3 (intervention* or therap* or treatment*)) OR AB((brief or combination or "compass* focus*" or integrated or integrative or time‐limited) N3 (intervention* or therap* or treatment*))  S27 TI (("client focus*" or non‐direct* or nondirect* or "solution focus*" or trauma* or talking) N3 therap*) OR AB (("client focus*" or non‐direct* or nondirect* or "solution focus*" or trauma* or talking) N3 therap*)  S28 TI(cognitiv* or cognition OR CBT) OR AB(cognitiv* or cognition OR CBT)  S29 TI((cope or coping) N1 (intervention* or mechanism* or skill* or technique*)) OR AB((cope or coping) N1 (intervention* or mechanism* or skill* or technique*))  S30 TI(counsel*ing) OR AB (counsel*ing)  S31 TI((couple* or family or group or systemic* or multimodal* or multi‐modal*) N3 (program* or intervention* or therap* or treat*)) OR AB((couple* or family or group or systemic* or multimodal* or multi‐modal*) N3 (program* or intervention* or therap* or treat*))  S32 TI("dialectical behavio*r* therap*") OR AB("dialectical behavio*r* therap*")  S33 TI(exercise* or "physical training") OR AB(exercise* or "physical training")  S34 TI((existential or gestalt or humanistic or interpersonal or milieu or person‐centred or residential or socioenvironmental or socio‐environmental) N1 therap*) OR AB((existential or gestalt or humanistic or interpersonal or milieu or person‐centred or residential or socioenvironmental or socio‐environmental) N1 therap*)  S35 TI("expressive writing") OR AB("expressive writing")  S36 TI("Eye Movement Desensitization and Reprocessing" or EMDR) OR AB("Eye Movement Desensitization and Reprocessing" or EMDR)  S37 TI(meditat* or "mental training" or mindfulness* or "mind training" or "brain training" or yoga) OR AB(meditat* or "mental training" or mindfulness* or "mind training" or "brain training" or yoga)  S38 TI("motivational interview") OR AB(" motivational interview")  S39 TI("reality therap*" or "problem solving") OR AB("reality therap*" or "problem solving")  S40 TI ("psycho* therap*" or psychotherap*) OR AB ("psycho* therap*" or psychotherap*)  S41 TI(psychoanalytic* or psycho‐analytic* or psychodynamic* or psycho‐dynamic*) OR AB(psychoanalytic* or psycho‐analytic* or psychodynamic* or psycho‐dynamic*)  S42 TI(psychodrama or psycho‐drama or "acting out" or "role play") OR AB(psychodrama or psycho‐drama or "acting out" or "role play")  S43 TI (psychosocial or psycho‐social or psychoeducation* or psycho‐education*) OR AB (psychosocial or psycho‐social or psychoeducation* or psycho‐education*)  S44 TI("rational emotive") OR AB("rational emotive")  S45 TI(Relax* N3 (training* or treatment* or therap*)) OR AB(Relax* N3 (training* or treatment* or therap*))  S46 TI(Service* N3 (refer* or use*)) OR AB(Service* N3 (refer* or use*))  S47 TI("stress inoculation training" or "prolonged exposure therapy" or "inoculation training" or "prolonged exposure therapy" or "cognitive processing therapy" or CPT) or AB("stress inoculation training" or "prolonged exposure therapy" or "inoculation training" or "prolonged exposure therapy" or "cognitive processing therapy" or CPT)  S48 TI((support or advice or advis*) N1 (centre* or center* or community or group* or network* or social or staff*)) OR AB((support or advice or advis*) N1 (centre* or center* or community or group* or network* or social or staff*))  S49 TI("therapeutic allianc*" or "therapeutic relationship*" or "therapeutic communit*") OR AB("therapeutic allianc*" or "therapeutic relationship*" or "therapeutic communit*")  S50 TI("Third wave") OR AB("Third wave")  S51 S10 OR S11 OR S12 OR S13 OR S14 OR S15 OR S16 OR S17 OR S18 OR S19 OR S20 OR S21 OR S22 OR S23 OR S24 OR S25 OR S26 OR S27 OR S28 OR S29 OR S30 OR S31 OR S32 OR S33 OR S34 OR S35 OR S36 OR S37 OR S38 OR S39 OR S40 OR S41 OR S42 OR S43 OR S44 OR S45 OR S46 OR S47 OR S48 OR S49 OR S50  S52 TI(rape N3 (centre* or center* or service* or support)) OR AB(rape N3 (centre* or center* or service* or support))  S53 TI((sex* assault N3 centre) or (sex* assault N3 center) or (sex* assault N3 service) or (sex* assault N3 support)) OR AB((sex* assault N3 centre) or (sex* assault N3 center) or (sex* assault N3 service) or (sex* assault N3 support))  S54 TI((sex* abuse* N3 centre) or (sex* abuse* N3 center) or (sex* abuse* N3 service) or (sex* abuse* N3 support)) OR AB((sex* abuse* N3 centre) or (sex* abuse* N3 center) or (sex* abuse* N3 service) or (sex* abuse* N3 support))  S55 S52 OR S53 OR S54  S56 S9 AND S51  S57 S55 OR S56  S58 DE "Meta Analysis" OR DE "Evaluation Research" OR DE "Control Groups" OR DE "Experimental Groups" OR DE "Longitudinal Studies" OR DE "Followup Studies" OR DE "Program Effectiveness" OR DE "Program Evaluation"  S59 TI (random* or trial* or experiment* or PROSPECTIVE* OR longitudinal or BLIND* or CONTROL*) OR AB (random* or trial* or experiment* or PROSPECTIVE* OR longitudinal or BLIND* or CONTROL* )  S60 S58 OR S59  S61 S57 AND S60  S62 S57 AND S60 Limiters ‐ Date Published: 20190101‐20211231 (Note: Final line 2021) S63 S57 AND S60 Limiters ‐ Date Published: 20210101‐20221231 (Note: Final line 2022)

#### Social Policy and Practice Ovid

Searched 3 July 2019 (1021 records) Searched 9 March 2021 (297 records) Searched 11 January 2022 (14 records)

1 (sex$ adj5 (abuse$ or assaul$ or attack$ or aggress$ or coer$ or exploit$ or force$ or molest$ or offen$ or traffick$ or trauma$ or unlawful$ or unwanted or violen$)).ti,ab,de.  2 (intercourse adj5 (coer$ or force$ or unwanted)).ti,ab,de.  3 (intimate partner adj (abuse or violence)).ti,ab,de.  4 (rape or raped or incest$).ti,ab,de.  5 (sex$ adj3 (victim$ or revictim$ or re‐victim$ or survivor$)).ti,ab,de.  6 (spouse adj (abuse or violence)).ti,ab,de.  7 (human trafficking adj10 sex$).ti,ab,de.  8 or/1‐7 9 ((abreaction or desensitization or exposure or implosive) adj3 therap$).ti,ab,de.  10 "acceptance and commitment therapy".ti,ab,de.  11 (advisor$ or advocate$ or advocacy).ti,ab,de.  12 ((animal$ or art or colo?r or creative$ or dance or dancing or drama or equine or experiential or music or narrative or play$ or sensory or singing) adj3 (program$ or intervention$ or therap$)).ti,ab,de.  13 (autogenic or autosuggestion$ or auto‐suggestion$ or breathing exercise$ or hypnosis or hypno‐therapy or hypnotherapy).ti,ab,de.  14 behavio$ activation.ti,ab,de. 15 (behavio?r$ adj3 (intervention$ or program$ or therap$ or training or treatment$)).ti,ab,de. 16 ((biofeedback or feedback or imagery) adj3 (intervention$ or therap$ or train$ or treatment$)).ti,ab,de.  17 ((brief or combination or compass$ focus$ or integrated or integrative or time‐limited) adj3 (intervention$ or therap$ or treatment$)).ti,ab,de.  18 ((client focus$ or non‐direct$ or nondirect$ or solution focus$ or trauma$ or talking) adj3 therap$).ti,ab,de.  19 (cognitiv$ or cognition or CBT).ti,ab,de.  20 ((cope or coping) adj1 (intervention$ or mechanism$ or skill$ or technique$)).ti,ab,de.  21 counsel?ing.ti,ab,de.  22 ((couple$ or family or group or systemic$ or multimodal$ or multi‐modal$) adj3 (program$ or intervention$ or therap$ or treat$)).ti,ab,de.  23 dialectical behavio?r$ therap$.ti,ab,de.  24 (exercise$ or physical training).ti,ab,de.  25 ((existential or gestalt or humanistic or interpersonal or milieu or person‐centred or residential or socioenvironmental or socio‐environmental) adj therap$).ti,ab,de.  26 expressive writing.ti,ab,de.  27 ("Eye Movement Desensitization and Reprocessing" or EMDR).ti,ab,de. 28 (meditat$ or mental training or mindfulness$ or mind training or brain training or yoga).ti,ab,de.  29 motivational interview$.ti,ab,de.  30 (reality therap$ or problem solving).ti,ab,de.  31 (psycho$ therap$ or psychotherap$).ti,ab,de.  32 (psychoanalytic$ or psycho‐analytic$ or psychodynamic$ or psycho‐dynamic$).ti,ab,de.  33 (psychodrama or psycho‐drama or acting out or role play).ti,ab,de.  34 (psychosocial or psycho‐social or psychoeducation$ or psycho‐education$).ti,ab,de.  35 rational emotive.ti,ab,de.  36 (Relax$ adj3 (training$ or treatment$ or therap$)).ti,ab,de.  37 (Service$ adj3 (refer$ or use$)).ti,ab,de.  38 (stress inoculation training or SIT or prolonged exposure therapy or PET or cognitive processing therapy or CPT).ti,ab,de.  39 ((support or advice or advis$1) adj1 (centre$1 or center$1 or community or group$ or network$ or social or staff$)).ti,ab,de.  40 (therapeutic allianc$ or therapeutic relationship$ or therapeutic communit$).ti,ab,de.  41 Third wave.ti,ab,de.  42 or/9‐41  43 8 and 42  44 (rape adj3 (centre$ or center$ or service$ or support)).ti,ab,de.  45 ((sex$ assault adj3 centre) or (sex$ assault adj3 center) or (sex$ assault adj3 service) or (sex$ assault adj3 support)).ti,ab,de.  46 ((sex$ abuse$ adj3 centre) or (sex$ abuse$ adj3 center) or (sex$ abuse$ adj3 service) or (sex$ abuse$ adj3 support)).ti,ab,de.  47 or/44‐46  48 43 or 47  49 (random* or RCT or control* or experimental* or trial* or placebo* or group* or blind* or longitudinal study or prospective study or "follow* up" or TAU or "usual care" or "treatment as usual").ti,ab,de.  50 48 and 49

#### Web of Science Core Collection Clarivate (SCI,SSCI, CPCI‐S, CPCI‐SSH)

Searched 4 July 2019 (1452 records) Searched 9 March 2021 (297 records) Searched 11 January 2022 (48 records)

#46 #42 AND #41 Indexes=SCI‐EXPANDED, SSCI, CPCI‐S, CPCI‐SSH Timespan=2021‐2022 (Note: Final line 2022) # 45 #42 AND #41 Indexes=SCI‐EXPANDED, SSCI, CPCI‐S, CPCI‐SSH Timespan=2019‐2021 (Note: Final line 2021) # 44 #42 AND #41 Refined by: [excluding] WEB OF SCIENCE CATEGORIES: ( PLANT SCIENCES OR HORTICULTURE OR LANGUAGE LINGUISTICS OR ENVIRONMENTAL SCIENCES OR TROPICAL MEDICINE OR AGRONOMY OR VIROLOGY OR PUBLIC ENVIRONMENTAL OCCUPATIONAL HEALTH OR AGRICULTURAL ENGINEERING OR AGRICULTURE DAIRY ANIMAL SCIENCE OR INFORMATION SCIENCE LIBRARY SCIENCE OR BUSINESS OR AGRICULTURE MULTIDISCIPLINARY OR ENERGY FUELS OR ENTOMOLOGY OR PEDIATRICS OR PATHOLOGY OR FOOD SCIENCE TECHNOLOGY OR FORESTRY OR ECOLOGY OR HISTORY OR ENDOCRINOLOGY METABOLISM OR HOSPITALITY LEISURE SPORT TOURISM OR HUMANITIES MULTIDISCIPLINARY OR MEDICINE RESEARCH EXPERIMENTAL OR LITERATURE GERMAN DUTCH SCANDINAVIAN OR MATERIALS SCIENCE MULTIDISCIPLINARY OR METEOROLOGY ATMOSPHERIC SCIENCES OR MICROBIOLOGY OR VETERINARY SCIENCES OR MYCOLOGY OR PHILOSOPHY OR UROLOGY NEPHROLOGY OR PUBLIC ADMINISTRATION OR EDUCATION SPECIAL OR ENVIRONMENTAL STUDIES OR REGIONAL URBAN PLANNING OR PHARMACOLOGY PHARMACY OR SOIL SCIENCE OR GEOGRAPHY OR IMMUNOLOGY ) #43 #42 AND #41 Indexes=SCI‐EXPANDED, SSCI, A&HCI, CPCI‐S, CPCI‐SSH, ESCI Timespan=All years # 42 TS=(random* or RCT or control* or experimental* or trial* or placebo* or group* or blind* or longitudinal or "follow* up" or TAU or "usual care" or "treatment as usual") Indexes=SCI‐EXPANDED, SSCI, A&HCI, CPCI‐S, CPCI‐SSH, ESCI Timespan=All years # 41 #40 OR #39 Indexes=SCI‐EXPANDED, SSCI, A&HCI, CPCI‐S, CPCI‐SSH, ESCI Timespan=All years #40 #37 OR #36 OR #35 Indexes=SCI‐EXPANDED, SSCI, A&HCI, CPCI‐S, CPCI‐SSH, ESCI Timespan=All years  #39 #38 AND #3  Indexes=SCI‐EXPANDED, SSCI, A&HCI, CPCI‐S, CPCI‐SSH, ESCI Timespan=All years #38 #34 OR #33 OR #32 OR #31 OR #30 OR #29 OR #28 OR #27 OR #26 OR #25 OR #24 OR #23 OR #22 OR #21 OR #20 OR #19 OR #18 OR #17 OR #16 OR #15 OR #14 OR #13 OR #12 OR #11 OR #10 OR #9 OR #8 OR #7 OR #6 OR #5 OR #4  Indexes=SCI‐EXPANDED, SSCI, A&HCI, CPCI‐S, CPCI‐SSH, ESCI Timespan=All years #37 ts= (rape NEAR/1 (centre* or center* or service* or support))  Indexes=SCI‐EXPANDED, SSCI, A&HCI, CPCI‐S, CPCI‐SSH, ESCI Timespan=All years #36 TS= (("sex* abuse*" NEAR/1 centre) or ("sex* abuse*" NEAR/1 center) or ("sex* abuse*" NEAR/1 service*) or ("sex* abuse*" NEAR/1 support))  Indexes=SCI‐EXPANDED, SSCI, A&HCI, CPCI‐S, CPCI‐SSH, ESCI Timespan=All years #35 TS= (("sex* assault" NEAR/1 centre) or ("sex* assault" NEAR/1 center) or ("sex* assault" NEAR/1 service*) or ("sex* assault" NEAR/1 support))  Indexes=SCI‐EXPANDED, SSCI, A&HCI, CPCI‐S, CPCI‐SSH, ESCI Timespan=All years #34 TS= ("therapeutic allianc*" or "therapeutic relationship*" or "therapeutic communit*" or "Third wave")  Indexes=SCI‐EXPANDED, SSCI, A&HCI, CPCI‐S, CPCI‐SSH, ESCI Timespan=All years #33 TS=((support or advice or advis* ) NEAR/1 (centre* or center* or communit* or group* or network* or social or staff*))  Indexes=SCI‐EXPANDED, SSCI, A&HCI, CPCI‐S, CPCI‐SSH, ESCI Timespan=All years #32 TS= ("stress inoculation training" or SIT or "prolonged exposure therapy" or PET or "cognitive processing therapy" or CPT)  Indexes=SCI‐EXPANDED, SSCI, A&HCI, CPCI‐S, CPCI‐SSH, ESCI Timespan=All years #31 TS=(Service* NEAR/1 (refer* or use*))  Indexes=SCI‐EXPANDED, SSCI, A&HCI, CPCI‐S, CPCI‐SSH, ESCI Timespan=All years #30 TS= (Relax* NEAR/1 (training* or treatment* or therap*))  Indexes=SCI‐EXPANDED, SSCI, A&HCI, CPCI‐S, CPCI‐SSH, ESCI Timespan=All years #29 TS=("rational emotive")  Indexes=SCI‐EXPANDED, SSCI, A&HCI, CPCI‐S, CPCI‐SSH, ESCI Timespan=All years #28 (TS=(psychosocial or "psycho‐social" or psychoeducation* or "psycho‐education*"))  Indexes=SCI‐EXPANDED, SSCI, A&HCI, CPCI‐S, CPCI‐SSH, ESCI Timespan=All years #27 TS= (psychodrama or "psycho‐drama" or "acting out" or "role play")  Indexes=SCI‐EXPANDED, SSCI, A&HCI, CPCI‐S, CPCI‐SSH, ESCI Timespan=All years #26 TS=(psychoanalytic* or "psycho‐analytic*" or psychodynamic* or "psycho‐dynamic*")  Indexes=SCI‐EXPANDED, SSCI, A&HCI, CPCI‐S, CPCI‐SSH, ESCI Timespan=All years #25 TS= ("psycho* therap*" or psychotherap*)  Indexes=SCI‐EXPANDED, SSCI, A&HCI, CPCI‐S, CPCI‐SSH, ESCI Timespan=All years #24 TS=("reality therap*" or "problem solving")  Indexes=SCI‐EXPANDED, SSCI, A&HCI, CPCI‐S, CPCI‐SSH, ESCI Timespan=All years #23 TS=("motivational interview*")  Indexes=SCI‐EXPANDED, SSCI, A&HCI, CPCI‐S, CPCI‐SSH, ESCI Timespan=All years #22 TS= (meditat* or "mental training" or mindfulness* or "mind training" or "brain training" or yoga)  Indexes=SCI‐EXPANDED, SSCI, A&HCI, CPCI‐S, CPCI‐SSH, ESCI Timespan=All years #21 TS=("Eye Movement Desensitization and Reprocessing" or EMDR)  Indexes=SCI‐EXPANDED, SSCI, A&HCI, CPCI‐S, CPCI‐SSH, ESCI Timespan=All years #20 ts=("expressive writing")  Indexes=SCI‐EXPANDED, SSCI, A&HCI, CPCI‐S, CPCI‐SSH, ESCI Timespan=All years #19 TS=((existential or gestalt or humanistic or interpersonal or milieu or "person‐centred" or residential or socioenvironmental or "socio‐environmental") NEAR/0 ( therap*))  Indexes=SCI‐EXPANDED, SSCI, A&HCI, CPCI‐S, CPCI‐SSH, ESCI Timespan=All years #18 TS=(exercise* or physical training)  Indexes=SCI‐EXPANDED, SSCI, A&HCI, CPCI‐S, CPCI‐SSH, ESCI Timespan=All years #17 TS=("dialectical behav* therap*")  Indexes=SCI‐EXPANDED, SSCI, A&HCI, CPCI‐S, CPCI‐SSH, ESCI Timespan=All years #16 (TS=((couple* or family or group or systemic* or multimodal* or multi‐modal*) near/3 (program* or intervention* or therap* or treat*)))  Indexes=SCI‐EXPANDED, SSCI, A&HCI, CPCI‐S, CPCI‐SSH, ESCI Timespan=All years #15 TS=((cope or coping) near/1 (intervention* or mechanism* or skill* or technique*))  Indexes=SCI‐EXPANDED, SSCI, A&HCI, CPCI‐S, CPCI‐SSH, ESCI Timespan=All years #14 TS=(cognitiv* or cognition or CBT)  Indexes=SCI‐EXPANDED, SSCI, A&HCI, CPCI‐S, CPCI‐SSH, ESCI Timespan=All years #13 TS=(("client focus*" or non‐direct* or nondirect* or "solution focus*" or trauma* or talking) near/3 therap*)  Indexes=SCI‐EXPANDED, SSCI, A&HCI, CPCI‐S, CPCI‐SSH, ESCI Timespan=All years #12 TS=((brief or combination or "compass* focus*" or integrated or integrative or "time limited") near/3 (intervention* or therap* or treatment*))  Indexes=SCI‐EXPANDED, SSCI, A&HCI, CPCI‐S, CPCI‐SSH, ESCI Timespan=All years #11 TS=((biofeedback or feedback or imagery) near/3 (intervention* or therap* or train* or treatment*))  Indexes=SCI‐EXPANDED, SSCI, A&HCI, CPCI‐S, CPCI‐SSH, ESCI Timespan=All years #10 TS= (behavio* near/1 (intervention* or program* or therap* or training or treatment*))  Indexes=SCI‐EXPANDED, SSCI, A&HCI, CPCI‐S, CPCI‐SSH, ESCI Timespan=All years #9 TS=("behavio* activation")  Indexes=SCI‐EXPANDED, SSCI, A&HCI, CPCI‐S, CPCI‐SSH, ESCI Timespan=All years #8 TS=(autogenic or autosuggestion* or auto‐suggestion* or "breathing exercise*" or hypnosis or hypno‐therapy or hypnotherapy)  Indexes=SCI‐EXPANDED, SSCI, A&HCI, CPCI‐S, CPCI‐SSH, ESCI Timespan=All years #7 TS=((animal* or art or colo*r or creative* or dance or dancing or drama or equine or experiential or music* or narrative or play* or sensory or singing) near/3 (program* or intervention* or therap*))  Indexes=SCI‐EXPANDED, SSCI, A&HCI, CPCI‐S, CPCI‐SSH, ESCI Timespan=All years #6 TS=(advisor* or advocate* or advocacy)  Indexes=SCI‐EXPANDED, SSCI, A&HCI, CPCI‐S, CPCI‐SSH, ESCI Timespan=All years #5 TS="acceptance and commitment therapy"  Indexes=SCI‐EXPANDED, SSCI, A&HCI, CPCI‐S, CPCI‐SSH, ESCI Timespan=All years #4 TS= ((abreaction or desensitization or exposure or implosive) near/1 therap* )  Indexes=SCI‐EXPANDED, SSCI, A&HCI, CPCI‐S, CPCI‐SSH, ESCI Timespan=All years #3 (#1 OR #2)  Indexes=SCI‐EXPANDED, SSCI, A&HCI, CPCI‐S, CPCI‐SSH, ESCI Timespan=All years #2 TI=(((rape or raped or incest*) OR (intercourse near/3 coerc*)) NOT offender*)  Indexes=SCI‐EXPANDED, SSCI, A&HCI, CPCI‐S, CPCI‐SSH, ESCI Timespan=All years #1 TI=((sex* near/0 (abuse* or assault* or attack* or aggress* or coer* or exploit* or force* or molest* or offen* or traffick* or trauma* or unlawful* or unwanted or violen*)) NOT (offender*))  Indexes=SCI‐EXPANDED, SSCI, A&HCI, CPCI‐S, CPCI‐SSH, ESCI Timespan=All years

Searched 5 July 2019 (4 records) Searched 11 March 2021 (125 records added between 5 July 2019 to 11 March 2021) Searched 12 January 2022 (No records added between 11 March 2021 to 12 January 2022)

(title:(title:(( sex OR sexual ) AND (abuse* OR assaul* OR attack* OR aggress* OR coer* OR exploit* OR force* OR molest* OR offen* OR traffick* OR trauma* OR unlawful* OR unwanted OR violen*)) OR title:(intimate partner violence OR intimate partner abuse) OR title:(rape OR raped OR incest*)) OR abstract:(title:(( sex OR sexual ) AND (abuse* OR assaul* OR attack* OR aggress* OR coer* OR exploit* OR force* OR molest* OR offen* OR traffick* OR trauma* OR unlawful* OR unwanted OR violen*)) OR title:(intimate partner violence OR intimate partner abuse) OR title:(rape OR raped OR incest*))) OR abstract:((( sex OR sexual ) AND (abuse* OR assaul* OR attack* OR aggress* OR coer* OR exploit* OR force* OR molest* OR offen* OR traffick* OR trauma* OR unlawful* OR unwanted OR violen*)) OR (intimate partner violence OR intimate partner abuse) OR title:(rape OR raped OR incest*)) NOT title:(offender* OR offending OR offended) NOT title:(child*)

Limited to publication type: systematic review

#### Cochrane Database of Systematic Reviews (CDSR)

Searched 5 July 2019 (17 records) Searched 8 March 2021 (4 records) Searched 10 January 2022 (2 records)

#1 [mh ^"sex offenses"]  #2 [mh Incest]  #3 [mh "intimate partner violence"]  #4 [mh "human trafficking"]  #5 [mh rape]  #6 [mh "spouse abuse"]  #7 (sex* NEAR/5 (abuse* or assaul* or attack* or aggress* or coer* or exploit* or force* or molest* or offen* or traffick* or trauma* or unlawful* or unwanted or violen*)):ti NOT (offender* or offended or offen*es):ti  #8 (intercourse NEAR/5 (coer* or force* or unwanted)):ti,ab,kw  #9 ("intimate partner violence" or "intimate partner abuse"):ti  #10 (rape or raped or incest*):ti 115 #11 (sex* NEAR/3 (victim* or revictim* or re‐victim* or survivor*)):ti,ab,kw  #12 {or #1‐#11}  #13 MeSH descriptor: [Anxiety Disorders] explode all trees and with qualifier(s): [therapy ‐ TH] #14 [mh "Adaptation, Psychological"]  #15 [mh "Behavior Therapy"]  #16 [mh "Combined Modality Therapy"]  #17 [mh ^"community networks"]  #18 [mh "Complementary therapies"]  #19 [mh Counseling]  #20 MeSH descriptor: [Depression] this term only and with qualifier(s): [therapy ‐ TH]  #21 MeSH descriptor: [Depressive Disorder, Major] explode all trees and with qualifier(s): [therapy ‐ TH]  #22 [mh Exercise]  #23 [mh "Exercise therapy"]  #24 [mh "Health Education"] #25 [mh "Health Knowledge, Attitudes, Practice"]  #26 [mh "Interview, Psychological"]  #27 [mh "mind body therapies"] #28 [mh "Psychological adjustment"]  #29 MeSH descriptor: [Psychological Trauma] explode all trees and with qualifier(s): [prevention & control ‐ PC, rehabilitation ‐ RH, therapy ‐ TH] 20 #30 [mh "psychosocial support systems"] 19 #31 [mh "psychotherapy"] 21875 #32 [mh "Referral and Consultation"] 2157 #33 [mh "Self‐Help Groups"] 743 #34 [mh "Social Support"] 3164 #35 MeSH descriptor: [Stress Disorders, Post‐Traumatic] explode all trees and with qualifier(s): [prevention & control ‐ PC, rehabilitation ‐ RH, therapy ‐ TH]  #36 MeSH descriptor: [Video Recording] this term only #37 MeSH descriptor: [Videotape Recording] this term only  #38 [mh Writing]  #39 ((abreaction or desensitization or exposure or implosive) NEAR/3 therap*):ti,ab,kw  #40 "acceptance and commitment therapy":ti,ab,kw  #41 (advisor* or advocate* or advocacy):ti,ab,kw #42 ((animal* or art or colo?r or creative* or dance or dancing or drama or equine or experiential or music or narrative or play* or sensory or singing) NEAR/3 (program* or intervention* or therap*)):ti,ab,kw  #43 (autogenic or autosuggestion* or auto next suggestion* or breathing next exercise* or hypnosis or hypno next therapy or hypnotherapy):ti,ab,kw  #44 behavio* next activation:ti,ab,kw  #45 (behavio* NEAR/3 (intervention* or program* or therap* or training or treatment*)):ti,ab,kw  #46 ((biofeedback or feedback or imagery) NEAR/3 (intervention* or therap* or train* or treatment*)):ti,ab,kw  #47 ((brief or combination or compass* next focus* or integrated or integrative or time next limited) NEAR/3 (intervention* or therap* or treatment*)):ti,ab,kw #48 ((client next focus* or non next direct* or nondirect* or solution next focus* or trauma* or talking) NEAR/3 therap*)  #49 (cognitiv* or cognition):ti,ab,kw  #50 CBT:ti,ab,kw  #51 ((cope or coping) NEAR/1 (intervention* or mechanism* or skill* or technique*)):ti,ab,kw #52 counsel*ing:ti,ab,kw  #53 ((couple* or family or group or systemic* or multimodal* or multi next modal*) NEAR/3 (program* or intervention* or therap* or treat*)):ti,ab,kw  #54 dialectical next behavio*r* next therap*:ti,ab,kw  #55 (exercise* or physical next training):ti,ab,kw  #56 ((existential or gestalt or humanistic or interpersonal or milieu or person‐centred or residential or socioenvironmental or socio next environmental) NEXT therap*):ti,ab,kw  #57 expressive next writing:ti,ab,kw  #58 ("Eye Movement Desensitization and Reprocessing" or EMDR):ti,ab,kw  #59 (meditat* or mental next training or mindfulness* or mind next training or brain next training or yoga):ti,ab,kw  #60 motivational next interview*:ti,ab,kw  #61 (reality next therap* or problem next solving):ti,ab,kw  #62 (psycho* next therap* or psychotherap*)  #63 (psychoanalytic* or psycho next analytic* or psychodynamic* or psycho next dynamic*):ti,ab,kw #64 (psychodrama or psycho next drama or acting next out or role next play):ti,ab,kw  #65 (psychosocial or psycho next social or psychoeducation* or psycho next education*):ti,ab,kw  #66 rational next emotive:ti,ab,kw  #67 (Relax* NEAR/3 (training* or treatment* or therap*)):ti,ab,kw  #68 (Service* NEAR/3 (refer* or use*)):ti,ab,kw  #69 (stress next inoculation next training or SIT or prolonged next exposure next therapy or PET or cognitive next processing next therapy or CPT):ti,ab,kw  #70 ((support or advice or advisor*) NEAR/1 (centre* or center* or community or group* or network* or social or staff*)):ti,ab,kw #71 (therapeutic next allianc* or therapeutic next relationship* or therapeutic next communit*):ti,ab,kw  #72 Third next wave:ti,ab,kw  #73 {or #13‐#72} #74 #12 and #73  #75 (rape NEAR/1 (centre* or center* or service* or support)):ti,ab,kw  #76 ((sex* next assault NEAR/3 centre) or (sex* next assault NEAR/3 center) or (sex* next assault NEAR/3 service) or (sex* next assault NEAR/3 support)):ti,ab,kw  #77 ((sex* next abuse* NEAR/3 centre) or (sex* next abuse* NEAR/3 center) or (sex* next abuse* NEAR/3 service) or (sex* next abuse* NEAR/3 support)):ti,ab,kw  #78 {or #74‐#77} in Cochrane Reviews, Cochrane Protocols #79 {or #74‐#77} with Cochrane Library publication date from Mar 2021 to Jan 2022

#### ClinicalTrials.gov

Searched 5 July 2019 (182 records after 17 studies about canola oil, or rapeseed were removed) Searched 11 March 2021 (30 records. First posted from 7 June 2019 to 3 November 2021) Searched 12 January 2022 (2 records. First posted from 3 November 2021 to 1 December 2022)

Interventional Studies | (Sexual Abuse OR SEXUAL ASSAULT OR RAPE OR INCEST OR "INTIMATE PARTNER") | Adult, Older Adult APPLIED FILTERS: INTERVENTIONAL ADULT 18‐64  OLDER ADULT (65+)

#### WHO ICTRP

Searched 8 July 2019 (229 records) Search attempted 11 March 2021 but website did not respond  Searched 12 January 2022 (43 records. Date of registration between 8 July 2019 to 12 January 2022)

CONDITION| Sexual Abuse OR SEXUAL ASSAULT OR RAPE OR INCEST OR "INTIMATE PARTNER"

#### UK Clinical Trials Gateway https://bepartofresearch.nihr.ac.uk/

Searched 5 July 2019 (3 records)  Searched 11 March 2021 (3 records)  Searched 12 January 2022 (no new records)

Searched for single terms and phrases:

violence; IPV; incest; sex; sexual; Post‐traumatic stress disorder (PTSD)

### Appendix 3. Cochrane Common Mental Disorders Controlled Trials Register

#### Core MEDLINE search

The search strategy below is the weekly OVID MEDLINE search, which was used to inform the Group’s specialised register. It is based on a list of terms for all conditions within the scope of the Cochrane Common Mental Disorders Group plus a sensitive RCT filter.

##### 1. [MeSH Headings]:

eating disorders/ or anorexia nervosa/ or binge‐eating disorder/ or bulimia nervosa/ or female athlete triad syndrome/ or pica/ or hyperphagia/ or bulimia/ or self‐injurious behavior/ or self mutilation/ or suicide/ or suicidal ideation/ or suicide, attempted/ or mood disorders/ or affective disorders, psychotic/ or bipolar disorder/ or cyclothymic disorder/ or depressive disorder/ or depression, postpartum/ or depressive disorder, major/ or depressive disorder, treatment‐resistant/ or dysthymic disorder/ or seasonal affective disorder/ or neurotic disorders/ or depression/ or adjustment disorders/ or exp antidepressive agents/ or anxiety disorders/ or agoraphobia/ or neurocirculatory asthenia/ or obsessive‐compulsive disorder/ or obsessive hoarding/ or panic disorder/ or phobic disorders/ or stress disorders, traumatic/ or combat disorders/ or stress disorders, post‐traumatic/ or stress disorders, traumatic, acute/ or anxiety/ or anxiety, castration/ or koro/ or anxiety, separation/ or panic/ or exp anti‐anxiety agents/ or somatoform disorders/ or body dysmorphic disorders/ or conversion disorder/ or hypochondriasis/ or neurasthenia/ or hysteria/ or munchausen syndrome by proxy/ or munchausen syndrome/ or fatigue syndrome, chronic/ or obsessive behavior/ or compulsive behavior/ or behavior, addictive/ or impulse control disorders/ or firesetting behavior/ or gambling/ or trichotillomania/ or stress, psychological/ or burnout, professional/ or sexual dysfunctions, psychological/ or vaginismus/ or Anhedonia/ or Affective Symptoms/ or *Mental Disorders/

##### 2. [Title/ Author Keywords]:

(eating disorder* or anorexia nervosa or bulimi* or binge eat* or (self adj (injur* or mutilat*)) or suicide* or suicidal or parasuicid* or mood disorder* or affective disorder* or bipolar i or bipolar ii or (bipolar and (affective or disorder*)) or mania or manic or cyclothymic* or depression or depressive or dysthymi* or neurotic or neurosis or adjustment disorder* or antidepress* or anxiety disorder* or agoraphobia or obsess* or compulsi* or panic or phobi* or ptsd or posttrauma* or post trauma* or combat or somatoform or somati#ation or medical* unexplained or body dysmorphi* or conversion disorder or hypochondria* or neurastheni* or hysteria or munchausen or chronic fatigue* or gambling or trichotillomania or vaginismus or anhedoni* or affective symptoms or mental disorder* or mental health).ti,kf.

##### 3. [RCT filter]:

(controlled clinical trial.pt. or randomised controlled trial.pt. or (randomi#ed or randomi#ation).ab,ti. or randomly.ab. or (random* adj3 (administ* or allocat* or assign* or class* or control* or determine* or divide* or distribut* or expose* or fashion or number* or place* or recruit* or subsitut* or treat*)).ab. or placebo*.ab,ti. or drug therapy.fs. or trial.ab,ti. or groups.ab. or (control* adj3 (trial* or study or studies)).ab,ti. or ((singl* or doubl* or tripl* or trebl*) adj3 (blind* or mask* or dummy*)).mp. or clinical trial, phase ii/ or clinical trial, phase iii/ or clinical trial, phase iv/ or randomised controlled trial/ or pragmatic clinical trial/ or (quasi adj (experimental or random*)).ti,ab. or ((waitlist* or wait* list* or treatment as usual or TAU) adj3 (control or group)).ab.)

##### 4. (1 and 2 and 3)

Records were screened for reports of RCTs within the scope of the Cochrane Common Mental Disorders Group. Secondary reports of RCTs were tagged to the appropriate study record. The CCMD‐CTR is current to June 2016 only.

### Appendix 4. Unused methods

#### Randomised cross‐over trials

Had we found cross‐over trials, we would have applied the same approach as recommended in Section 23.2.3 of the *Cochrane Handbook for Systematic Reviews of Interventions* (Higgins 2021b). As discussed in section 23.2.3 in the Handbook, this would involve using the variant of the Cochrane risk of bias tool designed specifically for cross‐over trials. This would have helped address some of the key issues when assessing risk of bias in cross‐over trials, including 1) bias arising from the randomisation process, 2) bias due to deviations from intended interventions, 3) bias due to missing outcome data, 4) bias in measurement of the outcome and 5) bias in selection of the reported outcome.

#### Continuous data

Had studies used the same continuous outcome measure, we would have calculated the mean difference (MD) with 95% confidence interval (CI). We would have presented conceptually distinct outcomes in separate forest plots. Had it been necessary to combine dichotomous data and continuous data in a meta‐analysis, we would have needed estimates of the standard error. Standard errors could have been computed for all studies by entering the data into RevMan 5 as dichotomous and continuous outcome type data, as appropriate, and converting the CI for the resulting log odds ratios and standardised mean differences (SMD) into standard errors (Lefebvre 2021). Once we had computed the SMD (or log odds ratios) and their standard errors for all studies in the meta‐analysis, we would have combined them using the generic inverse‐variance method in RevMan 5 (Review Manager 2014).

#### Cluster‐randomised trials

There was one included cluster trial (Bass 2013); however, this was not included in the meta‐analysis due to the nature of the comparison. Had we identified cluster‐randomised trials eligible for the meta‐analysis, we would have adjusted the standard errors or sample sizes using the method described in the *Cochrane Handbook for Systematic Reviews of Interventions* (Higgins 2021b). As described in section 23.1.4 in the Handbook, the adjustment method would have required the following information from the study: the number of clusters or groups randomised to each intervention group and the total number of participants in the study; the outcome data ignoring the cluster design for the total number of individuals; and an estimation of intracluster (or intraclass) correlation coefficient (ICC). This would have enabled an approximately correct analysis to be performed. Had the ICC not been available, we would have used ICCs from analogous cluster‐randomised trials. If analogous studies had not been available, we would have used a series of plausible values in a sensitivity analysis. An approximately correct analysis would proceed to reduce the size of each trial to its 'effective sample size' by dividing by the design effect. For continuous data, only the sample size needs to be reduced. For dichotomous data, the number of participants and those experiencing the event should be divided by the same design effect.

#### Dealing with missing data

We would have estimated the log rank statistics where these were not published in an article, and we would have used previously reported methods, where applicable (Parmar 1998; Tierney 2007). We would have addressed the potential impact of missing outcome data in the risk of bias assessment. If appropriate, we would have performed a sensitivity analysis to assess the impact of the missing information on our results (see Sensitivity analysis below), using the methods described in the *Cochrane Handbook for Systematic Reviews of Interventions* (Deeks 2021).

#### Data synthesis

Had it been inappropriate to combine the data in a meta‐analysis (on account of insufficient studies or data), we would have reported the effect sizes with 95% CI or standard errors of individual studies, and provided a narrative, rather than quantitative, summary of the findings that addressed the following aspects: 1) What was the direction of effect? 2) What was the size of effect? 3) Was the effect consistent across studies?

#### Subgroup analysis and investigation of heterogeneity

As described in Chapter 10 of the *Cochrane Handbook for Systematic Reviews of Interventions* (Deeks 2021), metaregression is an extension to subgroup analyses that allows the effect of continuous as well as categorical characteristics to be investigated and, in principle, allows the effects of multiple factors to be investigated simultaneously. Generally, metaregression should not be considered when there are fewer than 10 studies in a meta‐analysis. Had there been more than 10 studies available, we would have used metaregression techniques recommended for Stata (Harbord 2004; Stata 2017).

Participant characteristics have been identified as integral to subgroup analyses, as there may be differences in the efficacy of treatments for different groups of individuals. However, we are aware that subgroup analyses of subsets of participants are challenged because sufficient details to extract data about separate participant types are seldom published in reports. Recruitment setting has also been identified as important, as there may be differences between survivors recruited via mental health clinics as opposed to in the community or acute sexual assault services. Intensity of interventions is also of interest; for example, we might have compared outcomes from intense psychological therapies versus interventions oriented towards provision of psychosocial support. Finally, it would have been important to stratify analyses by type of intervention given their distinct mechanisms and theoretical underpinnings. Had we found sufficient studies, we would have conducted the following subgroup analyses:

- participant characteristics (e.g. gender, ethnicity, time to treatment, symptom load and types of trauma exposure);
- intensity of intervention (e.g. up to four sessions, five or more sessions);
- mode of intervention delivery (e.g. individual versus group); and
- setting of recruitment or intervention delivery (healthcare, community, acute settings).

#### Sensitivity analyses

We would have performed the following sensitivity analysis, provided there were sufficient numbers of studies, to examine any effects on the overall outcome:

- re‐analysis of the data excluding studies with missing outcome data.

Additional sensitivity analyses would have been required if particular issues related to the studies under review had arisen; for example, if ICCs were not available in included cluster trials or analogous cluster‐randomised trials, we would have used a series of plausible values in a sensitivity analysis.

## Data and analyses

**Comparison 1:** Psychosocial interventions versus inactive control

Outcome or subgroup title	No. of studies	No. of participants	Statistical method	Effect size
1.1 Psychosocial interventions versus inactive control; outcome 1: PTSD symptoms, post‐treatment	16	1130	Std. Mean Difference (IV, Random, 95% CI)	‐0.83 [‐1.22, ‐0.44]
1.1.1 Cognitive Behavioural Therapy	6	575	Std. Mean Difference (IV, Random, 95% CI)	‐0.77 [‐1.12, ‐0.42]
1.1.2 Behavioural Therapy	7	233	Std. Mean Difference (IV, Random, 95% CI)	‐1.85 [‐3.00, ‐0.70]
1.1.3 Psychosocial (low‐intensity) interventions	4	322	Std. Mean Difference (IV, Random, 95% CI)	0.26 [‐0.04, 0.55]
1.2 Psychosocial interventions versus inactive control; outcome 1: PTSD symptoms, 3 months	7	770	Std. Mean Difference (IV, Random, 95% CI)	‐0.13 [‐0.42, 0.17]
1.3 Psychosocial interventions versus inactive control; outcome 2: Depressive symptoms, post‐treatment	12	901	Std. Mean Difference (IV, Random, 95% CI)	‐0.82 [‐1.17, ‐0.48]
1.3.1 Cognitive Behavioural Therapy	7	595	Std. Mean Difference (IV, Random, 95% CI)	‐0.73 [‐1.13, ‐0.33]
1.3.2 Behavioural Therapy	4	123	Std. Mean Difference (IV, Random, 95% CI)	‐1.51 [‐2.58, ‐0.44]
1.3.3 Psychosocial (low‐intensity) interventions	2	183	Std. Mean Difference (IV, Random, 95% CI)	‐0.34 [‐1.12, 0.44]
1.4 Psychosocial interventions versus inactive control; outcome 2: Depressive symptoms, 3 months	3	376	Std. Mean Difference (IV, Random, 95% CI)	‐0.05 [‐0.39, 0.29]
1.5 Psychosocial interventions versus inactive control; outcome 3: Dropout from treatment	5	242	Risk Ratio (M‐H, Random, 95% CI)	0.85 [0.51, 1.44]
1.6 Psychosocial interventions versus inactive control; outcome 4: Adverse events	6	622	Risk Ratio (M‐H, Random, 95% CI)	1.92 [0.30, 12.41]
1.7 Psychosocial interventions versus inactive control; outcome 5: Anxiety symptoms, post‐treatment	10	436	Std. Mean Difference (IV, Random, 95% CI)	‐0.84 [‐1.26, ‐0.42]
1.8 Psychosocial interventions versus inactive control; outcome 5: Anxiety symptoms, 3 months	4	449	Std. Mean Difference (IV, Random, 95% CI)	‐0.26 [‐0.44, ‐0.07]
1.9 Psychosocial interventions versus inactive control; outcome 6: Global mental health/distress, post‐treatment	3	80	Std. Mean Difference (IV, Random, 95% CI)	‐0.92 [‐1.70, ‐0.13]

**Comparison 2:** Trauma‐focused versus non trauma‐focused interventions

Outcome or subgroup title	No. of studies	No. of participants	Statistical method	Effect size
2.1 Trauma‐focused versus non trauma‐focused interventions; outcome 1: PTSD symptoms, post‐treatment	10	727	Std. Mean Difference (IV, Random, 95% CI)	‐0.18 [‐0.48, 0.13]
2.2 Trauma‐focused versus non trauma‐focused interventions; outcome 1: PTSD symptoms, 3 months	8	584	Std. Mean Difference (IV, Random, 95% CI)	‐0.33 [‐0.49, ‐0.16]
2.3 Trauma‐focused versus non trauma‐focused interventions; outcome 1: PTSD symptoms, 6 months	5	533	Std. Mean Difference (IV, Random, 95% CI)	‐0.21 [‐0.43, 0.01]
2.4 Trauma‐focused versus non trauma‐focused interventions; outcome 2: Depressive symptoms, post‐treatment	9	673	Std. Mean Difference (IV, Random, 95% CI)	‐0.21 [‐0.54, 0.12]
2.5 Trauma‐focused versus non trauma‐focused interventions; outcome 2: Depressive symptoms, 3 months	7	535	Std. Mean Difference (IV, Random, 95% CI)	‐0.56 [‐0.97, ‐0.15]
2.6 Trauma‐focused versus non trauma‐focused interventions; outcome 2: Depressive symptoms, 6 months	5	532	Std. Mean Difference (IV, Random, 95% CI)	‐0.68 [‐1.49, 0.13]
2.7 Trauma‐focused versus non trauma‐focused interventions; outcome 3: Dropout from treatment	10	859	Risk Ratio (M‐H, Random, 95% CI)	1.43 [1.08, 1.87]
2.8 Trauma‐focused versus non trauma‐focused interventions; outcome 4: Adverse events	5	591	Risk Ratio (M‐H, Random, 95% CI)	0.63 [0.29, 1.37]
2.9 Trauma‐focused versus non trauma‐focused interventions; outcome 5: Anxiety symptoms, post‐treatment	7	540	Std. Mean Difference (IV, Random, 95% CI)	‐0.15 [‐0.42, 0.13]
2.10 Trauma‐focused versus non trauma‐focused interventions; outcome 5: Anxiety symptoms, 3 months	5	403	Std. Mean Difference (IV, Random, 95% CI)	‐0.23 [‐0.49, 0.02]
2.11 Trauma‐focused versus non trauma‐focused interventions; outcome 5: Anxiety symptoms, 6 months	3	399	Std. Mean Difference (IV, Random, 95% CI)	‐0.05 [‐0.37, 0.28]
2.12 Trauma‐focused versus non‐trauma focused interventions; outcome 7: Trauma‐related beliefs, post‐treatment	3	152	Std. Mean Difference (IV, Random, 95% CI)	0.16 [‐0.17, 0.50]
2.13 Trauma‐focused versus non trauma‐focused interventions; outcome 7: Global mental health/distress, post‐treatment	3	342	Std. Mean Difference (IV, Random, 95% CI)	0.07 [‐0.36, 0.51]
2.14 Trauma‐focused versus non trauma‐focused interventions; outcome 7: Global mental health/distress, 3 months	3	341	Std. Mean Difference (IV, Random, 95% CI)	‐0.35 [‐1.00, 0.31]

## Characteristics of studies

### Characteristics of included studies [ordered by study ID]

**Table:** Abrahams 2010

***Study characteristics***	
Methods	**Study design:** randomised controlled trial **Study grouping:** parallel group **Duration of study:** August 2007 to May 2008 **Location of study:** Eastern Cape Province and Western Cape, South Africa **Randomisation method:** a computer‐generated randomisation list was generated by the study statistician, and random block sizes of 4, 6 and 8 were used to ensure balance in the 2 arms. Participants were allocated to an arm by the study co‐ordinator after the initial data had been collected and leaflet explained. The contact information of intervention group participants was forwarded to the counsellors, who commenced the intervention within 12 h. **Ethics approvals:** ethical approval granted by the Medical Research Council
Participants	**Baseline characteristics** Other psychosocial interventions (telephonic psychosocial support) *Participants (at randomisation)*: 136 *Gender*: 100% female participants *Age*: 10.4% < 10 years, 18.4% 10 years to 14 years, 19.2% 15 years to 17 years, 30.4% 18 years to 22 years, 21.6% > 22 years *Ethnicity (% white, Black, other)*: 100% Black *Disability*: not given *Time since trauma in months, M (SD)*: < 7 d *Income*: not given *Education (% grade)*: 25.6% < 8, 25.6% 8 or 9, 39.2 10 or 11, 9.6% school completion or beyond *Employment*: 29.5% employed, 33.3% not employed, 37.2% student *Sexual violence in adulthood*: 52% *Comorbid conditions*: not given *Baseline PTSD*: not given *Partnered*: not given *Randomised (N)*: 136 *Completed post‐treatment assessment (N)*: 125 *Dropped out or removed prior to analysis (N)*: 11 *Numbers analysed at final applicable time point (N)*: 125 *Number of sessions, M (SD)*: 14.9 (9.8) *Treatment completion*: 119 (88.8%) Usual care *Participants (at randomisation)*: 138 *Gender*: 100% female participants *Age*: 4.7% < 10 years, 20.3% 10 years to 14 years, 22.7% 15 years to 17 years, 25.8% 18 years to 22 years, 26.6% > 22 years *Ethnicity*: 100% Black *Disability*: not given *Time since trauma*: not given *Income*: not given *Education (% grade)*: 26.6% < 8, 18.7% 8 to 9, 43.7% 10 to 11, 10.9% school completion or beyond *Employment*: 34.9% employed, 34.9% not employed, 30.1% student *Sexual violence in adulthood*: 52.4% *Comorbid conditions*: not given *Baseline PTSD*: not given *Partnered*: not given *Randomised (N)*: 138 *Completed post‐treatment assessment (N)*: 128 *Dropped out or removed prior to analysis (N)*: 10 *Numbers analysed at final applicable time point (N)*: 128 *Number of sessions*: not given *Treatment completion*: N/A Overall *Participants (at randomisation)*: 274 *Gender*: 100% female participants *Age*: not given *Ethnicity*: 100% Black *Disability*: not given *Time since trauma in months, M (SD)*: last 7 d *Income*: not given *Education*: not given *Employment*: not given *Sexual violence in adulthood*: 52% *Comorbid conditions*: not given *Baseline PTSD*: not given *Partnered*: not given *Approached*: 2011 *Ineligible (N)*: 1701 *Declined (N)*: 31 *Other (N)*: 5 *Randomised (N)*: 274 *Completed post‐treatment assessment (N)*: 253 *Dropped out or removed prior to analysis (N)*: 21 *Numbers analysed at final applicable time point (N)*: 253 *Number of sessions*: N/A *Treatment completion*: N/A **Inclusion criteria** Female victims of rape For children younger than 16 years, the carer became the focus of the intervention, was shown the leaflet and was provided with the telephonic adherence support. **Exclusion criteria** Rape victims who did not get PEP because they were HIV‐positive or did not take an HIV test Those who were unable to give consent for the research for reasons of severe injury or severe mental distress (including severely distressed guardians) Those who were not contactable telephonically **Pretreatment:** none reported
Interventions	**Intervention characteristics** Other psychosocial interventions (telephonic psychosocial support) *Intensity of intervention (sessions)*: 12 *Mode of delivery (face‐to‐face, online, video, telephone, blend, with explanation)*: telephone *Format (group, individual, blend)*: individual *Therapist qualifications and training appropriate (yes, no, unclear, with explanation)*: yes, counsellors had HIV and rape trauma counselling skills *Research allegiance or conflict of interest (yes, no, unclear, with explanation)*: unclear *Treatment fidelity (yes, no, unclear, with explanation)*: unclear; checklists were used during each call but no rating given to quality of fidelity to intervention and not used in analysis *Intervention aim and theoretical basis*: main purpose of the intervention was to test the hypothesis that the provision of telephonic psychosocial support to rape survivors and their families would lead to greater adherence to PEP for HIV over 28 d than would provision of a leaflet, adherence diary and standard post‐rape care; not a particularly strong theoretical basis beyond encouraging adherence to ART *Duration of intervention*: not specified, although it was for the period of 28 d of taking PEP Usual care *Intensity of intervention*: N/A *Mode of delivery (face‐to‐face, online, video, telephone, blend, with explanation)*: face‐to‐face *Format (group, individual, blend)*: individual *Therapist qualifications and training appropriate*: N/A *Research allegiance or conflict of interest*: N/A *Treatment fidelity*: N/A *Intervention aim and theoretical basis*: a leaflet, adherence diary and standard post‐rape care, which consisted of psychological 'containment', medical examination and collection of forensic evidence and HIV testing with precounselling and postcounselling
Outcomes	**Depression (data requested)** Outcome type: continuous outcome Scale used: CES‐D Direction: lower is better Score range: 0 to 60 Data value: endpoint **Treatment dropout (intervention only)** Outcome type: dichotomous outcome
Identification	**Sponsorship source:** Irish Aid and South African Medical Research Council **Country:** South Africa **Setting:** sexual assault services **Authors name:** Naeemah Abrahams **Institution:** Gender & Health Research Unit, Medical Research Council, Cape Town **Email:** nabraham@mrc.ac.za **Year:** 2010
Notes	No baseline data available for outcome Communicated with author(s) for purpose of review Data included in meta‐analysis

**Table:** Acierno 2021

***Study characteristics***	
Methods	**Study design:** randomised controlled trial **Study grouping:** parallel group **Duration of study:** 30 October 2014 to 10 September 2019 **Location of study:** Southeastern USA **Randomisation method:** eligible veterans were randomised by the project co‐ordinator using REDCap 1:1 to PE using HBT or in‐person PE, and REDCap generated assignments that were saved as they were made and reviewed by the study statistician; thereby, no modification or skipping of assignments was possible. **Ethics approvals:** not given
Participants	**Baseline characteristics** CBT (PE via HBT) *Participants*: 69 *Gender*: 100% female participants *Age, M (SD)*: 41.55 years (12.10 years) *Ethnicity*: 30.43% white, 62.32% African American, 7.25% other *Disability*: not given *Time since trauma*: not given *Income*: not given *Education*: not given *Employment*: not given *Sexual violence in adulthood*: 100% *Comorbid conditions*: not given *Baseline PTSD, M (SD)*: 52.90 (12.91) *Partnered*: not given *Randomised (N)*: 69 *Completed post‐treatment assessment (N)*: 46 *Dropped out or removed prior to analysis (N)*: 27 *Numbers analysed at final applicable time point (N)*: 42 *Number of sessions, M (SD)*: 6.80 (4.14) *Treatment completion*: 34 (49.27%) CBT (PE via in‐person) *Participants*: 67 *Gender (% female, male, other)*: 100% female participants *Age in years (M, SD)*: 45.31 (10.63) *Ethnicity*: 28.36% white, 65.67% African American, 5.97% other *Disability*: not given *Time since trauma*: not given *Income*: not given *Education*: not given *Employment*: not given *Sexual violence in adulthood*: 100% *Comorbid conditions*: not given *Baseline PTSD, M (SD)*: 52.30 (12.42) *Partnered*: not given *Randomised (N)*: 67 *Completed post‐treatment assessment (N)*: 49 *Dropped out or removed prior to analysis (N)*: 22 *Numbers analysed at final applicable time point (N)*: 45 *Number of sessions, M (SD)*: 6.28 (4.33) *Treatment completion*: 33 (49.25%) Overall *Participants*: 136 *Gender*: 100% female participants *Age, M (SD)*: 43.40 years (11.51 years) *Ethnicity*: 29.41% white, 63.97% African American, 6.62% other *Disability*: not given *Time since trauma*: not given *Income*: not given *Education*: not given *Employment*: not given *Sexual violence in adulthood*: 100% *Comorbid conditions*: not given *Baseline PTSD, M (SD)*: 52.60 (12.63) *Partnered*: not given *Approached (N)*: 172 Ineligible *(N)*: 19 *Declined (N)*: 16 *Other (N)*: 1 *Randomised (N)*: 136 *Completed post‐treatment assessment (N)*: 95 *Dropped out or removed prior to analysis (N)*: 49 *Numbers analysed at final applicable time point (N)*: 87 *Number of sessions, M (SD)*: 6.54 (4.23) *Treatment completion*: 67 (49.26%) **Inclusion criteria** An MST‐related index event as identified on the Stressful Events for Veterans Questionnaire Met criteria for PTSD related to MST based on the Clinician‐Administered PTSD Scale **Exclusion criteria** Active psychosis or dementia at screening Current suicidal ideation with clear intent Current severe substance use disorder Concurrently enrolled in a clinical trial for PTSD or depression or if they had a household member in the study Further, medication stabilisation was ensured with a 4‐week waiting period after recent medication change prior to enrolling in the study for more information about study design. **Pretreatment:** those assigned to the in‐person delivery condition were more likely to be Hispanic, but no other group differences were identified.
Interventions	**Intervention characteristics** CBT (PE via HBT) *Planned number of intervention sessions*: 12 to 15 *Mode of delivery (face‐to‐face, online, video, telephone, blend, with explanation)*: teleconference (video/audio) *Format (group, individual, blend)*: individual *Therapist qualifications and training appropriate (yes, no, unclear, with explanation)*: unclear, no information given *Research allegiance or conflict of interest (yes, no, unclear, with explanation)*: no, team is committed to establishing if HBT delivery brings benefits and has used the technology in several other trials, but there is no clear evidence of a conflict of interest. *Treatment fidelity (yes, no, unclear, with explanation)*: no, information not given *Intervention aim and theoretical basis*: PE is a manualised treatment (Foa 2007) that includes the following components: 1) psychoeducation and treatment rationale (Sessions 1 and 2); 2) repeated in vivo exposure to traumatic stimuli (in vivo exercises are assigned as homework during Sessions 3 through 11); 3) repeated, prolonged, imaginal exposure to traumatic memories (imaginal exposure is implemented during Sessions 3 through 11, and participants listen to session audiotapes for homework between sessions), and 4) relapse prevention strategies and further treatment planning (Session 12). *Duration of intervention*: 12 weeks to 15 weeks CBT (PE via in‐person) *Planned number of intervention sessions*: 12 to 15 *Mode of delivery (face‐to‐face, online, video, telephone, blend, with explanation)*: face‐to‐face *Format (group, individual, blend)*: individual *Therapist qualifications and training appropriate (yes, no, unclear, with explanation)*: unclear, no information given *Research allegiance or conflict of interest (yes, no, unclear, with explanation)*: no *Treatment fidelity (yes, no, unclear, with explanation)*: unclear, no information given *Intervention aim and theoretical basis*: in the case of women with a history of MST, accessibility will be enhanced by removing a barrier to care associated with the treatment environment that can potentially elicit an anxiety and avoidance response in the target population. HBT care is delivered using via Movi/Jabber software packages installed on standard tablet devices or home computers with standard internet connections to teleconference (video and audio) in real time. *Duration of intervention*: 12 weeks to 15 weeks
Outcomes	**PTSD** Outcome type: continuous outcome Scale used: PCL‐5 Direction: lower is better Score range: 0 to 80 Data value: endpoint **Depression** Outcome type: continuous outcome Scale used: BDI‐II Direction: lower is better Score range: 0 to 63 Data value: endpoint **Treatment dropout** Outcome type: dichotomous outcome
Identification	**Sponsorship source:** Department of Defense (W81XWH‐14‐1‐0264; PI: Acierno) and from the NIMH (T32 MH18869, PIs: Kilpatrick and Danielson) **Country:** USA **Setting:** VA Medical Centre; individuals from range of VA support services and affiliated community clinics screening positive for MST‐related PTSD **Authors name:** Ronald Acierno **Institution:** McGovern Medical School, University of Texas **Email:** ronald.acierno@uth.tmc.edu **Address:** N/A **Year:** unpublished
Notes	Unpublished data. This study is under review. This paper should not be used for any other purpose or shared with anyone else. Data not included in meta‐analysis Communicated with the author(s) for purpose of review

**Table:** Anderson 2010

***Study characteristics***	
Methods	**Study design:** randomised controlled trial **Study grouping:** parallel group **Duration of study:** not identified **Location of study:** Midwestern USA **Randomisation method:** not provided **Ethics approvals:** not provided
Participants	**Baseline characteristics** CBT (clinician‐assisted emotional disclosure) *Participants (select at randomisation if available)*: 15 *Gender*: 100% female participants *Age*: not given *Ethnicity*: not given *Disability*: not given *Time since trauma*: not given *Income*: not given *Education (% high school, college or apprentice, university or years of education)*: 100% university *Employment*: not given *Sexual violence in adulthood*: 100% *Comorbid conditions*: not given *Baseline PTSD, M (SD)*: 2.91 (0.80) *Partnered*: not given *Randomised (N)*: 15 *Completed post‐treatment assessment (N)*: 13 *Dropped out or removed prior to analysis (N)*: 2 *Numbers analysed at final applicable time point (N)*: 13 *Number of sessions, M (SD)*: 4 (0) *Treatment completion*: 13 (87%) No‐treatment control *Participants (select at randomisation if available)*: 13 *Gender*: 100% female participants *Age*: not given *Ethnicity*: not given *Disability*: not given *Time since trauma*: not given *Income*: not given *Education (% high school, college or apprentice, university or years of education)*: 100% university *Employment*: not given *Sexual violence in adulthood*: 100% *Comorbid conditions*: not given *Baseline PTSD, M (SD)*: 2.88 (0.63) *Partnered*: not given *Randomised (N)*: 13 *Completed post‐treatment assessment (N)*: 13 *Dropped out or removed prior to analysis (N)*: 0 *Numbers analysed at final applicable time point (N)*: 13 *Number of sessions*: N/A *Treatment completion*: N/A Overall *Participants (select at randomisation if available)*: 28 *Gender*: 100% female participants *Age, M (SD)*: 19.3 years (1.09 years) *Ethnicity*: 85.7% white *Disability*: not given *Time since trauma*: not given *Income*: not given *Education (% high school, college or apprentice, university or years of education)*: 100% university *Employment*: not given *Sexual violence in adulthood (%)*: 100% *Comorbid conditions*: not given *Baseline PTSD*: not given *Partnered*: not given *Approached (N)*: 670 *Ineligible (N)*: 627 *Declined (N)*: 6 *Other (N)*: 9 *Randomised (N)*: 28 *Completed post‐treatment assessment (N)*: 26 *Dropped out or removed prior to analysis (N)*: 2 *Numbers analysed at final applicable time point (N)*: 26 *Number of sessions*: N/A *Treatment completion*: N/A **Inclusion criteria** Experience of sexual assault that involved coerced or forced vaginal, oral or anal penetration Score of 59 or above on the Outcome Questionnaire‐45 **Exclusion criteria:** none reported **Pretreatment:** none reported
Interventions	**Intervention characteristics** CBT (clinician‐assisted emotional disclosure) *Planned number of intervention sessions*: 4 *Mode of delivery (face‐to‐face, online, video, telephone, blend, with explanation)*: face‐to‐face *Format (group, individual, blend)*: individual *Therapist qualifications and training appropriate (yes, no, unclear, with explanation)*: yes, presumably qualified therapists completed a 2‐day workshop and had at least 1 supervised training case *Research allegiance or conflict of interest (yes, no, unclear, with explanation)*: no *Treatment fidelity (yes, no, unclear, with explanation)*: unclear, used a manualised and replicable protocol but no recording of sessions, for example *Intervention aim and theoretical basis*: an adaptation of emotion‐focused therapy (Elliot 2004; Greenberg 1993) including 1) systematic evocative unfolding of emotional narratives and 2) emotional focusing, for experientially tracking emotions at a moment‐to‐moment level; 4 sessions over 10 d, ideally 4 consecutive days *Duration of intervention*: 2 weeks No‐treatment control *Planned number of intervention sessions*: N/A *Mode of delivery*: N/A *Format*: N/A *Therapist qualifications and training appropriate*: N/A *Research allegiance or conflict of interest*: N/A *Treatment fidelity*: N/A *Intervention aim and theoretical basis*: N/A *Duration of intervention*: N/A
Outcomes	**PTSD** Outcome type: continuous outcome Scale used: IES‐R Avoidance Direction: lower is better Score range: 0 to 4 Data value: endpoint **PTSD** Outcome type: continuous outcome Scale used: IES‐R Intrusion Direction: lower is better Score range: 0 to 4 Data value: endpoint **PTSD** Outcome type: continuous outcome Scale used: IES‐R Hyperarousal Direction: lower is better Score range: 0 to 4 Data value: endpoint **Global mental health** Outcome type: continuous outcome Scale used: Inventory of Interpersonal Problems Direction: lower is better Score range: 0 to 4 Data value: endpoint
Identification	**Sponsorship source: not given** **Country:** USA **Setting:** university undergraduates **Authors name:** Timothy Anderson **Institution:** Ohio University **Email:** andersot@ohio.edu **Year:** 2010
Notes	Included in meta‐analysis

**Table:** Bass 2013

***Study characteristics***	
Methods	**Study design:** cluster‐randomised controlled trial **Study grouping:** parallel group **Duration of study:** December 2010 to January 2012 **Ethics approvals:** IRBs at the Johns Hopkins Bloomberg School of Public Health and Kinshasa School of Public Health approved the protocol **Location of study:** 14 villages in South Kivu province and 2 villages on the border in North Kivu province, Democratic Republic of the Congo **Randomisation method:** the 16 study villages, each with 1 psychosocial assistant, were grouped into blocks of 2 to 4 villages on the basis of proximity and shared language and were randomly assigned to provide CPT or individual support. After therapy training, 1 psychosocial assistant was excluded because training‐based quizzes and skill observation raised competency concerns; therefore, the village in which the assistant worked was excluded. The trial included 15 study villages (7 that provided therapy and 8 that provided individual support).
Participants	**Baseline characteristics** CPT (no trauma narrative) *Participants (at randomisation)*: 7 villages, 157 *Gender*: 100% female participants *Age, M (SD)*: 36.9 years (13.4 years) *Ethnicity*: 100% Black *Disability*: not given *Time since trauma*: not given *Income*: not given *Education, M (SD) years*: 1.8 (2.8) *Employment*: not given *Sexual violence in adulthood*: 100% *Comorbid conditions*: not given *Baseline PTSD, M (SD)*: 1.9 (0.6) *Partnered*: 59% *Approached*: 222 *Ineligible (N)*: 32 *Declined (N)*: 0 *Other (N)*: 33 *Randomised (N)*: 157 *Completed post‐treatment assessment (N)*: 114 *Dropped out or removed prior to analysis (N)*: 19 *Numbers analysed at final applicable time point (N)*: 138 *Number of sessions, M (SD)*: 8.5 *Treatment completion*: 141 (90%) Other psychosocial interventions (individual support) *Participants (at randomisation)*: 8 villages, 248 *Gender*: 100% female participants *Age, M (SD)*: 33.8 years (12.4 years) *Ethnicity*: 100% Black *Disability*: not given *Time since trauma*: not given *Income*: not given *Education, M (SD) years*: 2.3 (3.1) *Employment*: not given *Sexual violence in adulthood*: 100% *Comorbid conditions*: not given *Baseline PTSD, M (SD)*: 2.2 (0.5) *Partnered*: 43% *Approached (N)*: 273 *Ineligible (N)*: 25 *Declined (N)*: 0 *Other (N)*: 0 *Randomised (N)*: 248 *Completed post‐treatment assessment (N)*: 156 *Dropped out or removed prior to analysis (N)*: 73 *Numbers analysed at final applicable time point (N)*: 175 *Number of sessions*: not given *Treatment completion*: 182 (73%) Overall *Participants (at randomisation)*: 15 villages, 405 *Gender*: 100% female participants *Age*: not given *Ethnicity*: 100% Black *Disability*: not given *Time since trauma*: not given *Income*: not given *Education*: not given *Employment*: not given *Sexual violence in adulthood*: 100% *Comorbid conditions*: not given *Baseline PTSD*: not given *Partnered*: not given *Approached (N)*: 495 *Ineligible (N)*: 57 *Declined (N)*: 0 *Other (N)*: 33 *Randomised (N)*: 405 *Completed post‐treatment assessment (N)*: 270 *Dropped out or removed prior to analysis (N)*: 92 *Numbers analysed at final applicable time point (N)*: 313 **Inclusion criteria** Clusters 14 villages in South Kivu province and 2 villages on the border in North Kivu province were selected from among 23 villages served by 3 Congolese nongovernmental organisations. Selection was based on accessibility, security and the availability of psychosocial assistants. Individuals Women who had experienced or witnessed sexual violence A total symptom score of at least 55 (i.e. an average score of 1 for each of 55 symptoms, comprising the Hopkins Symptom Checklist, the PTSD Checklist items and additional locally relevant symptoms) A functional‐impairment score of at least 10 (i.e. dysfunction on at least half the activities) **Exclusion criteria:** suicidality that was judged by clinical staff to require immediate treatment **Pretreatment:** women in the comparison group were younger, less likely to be married, lived with fewer people, and had higher levels of distress and stigma than the intervention group.
Interventions	**Intervention characteristics** CPT (no trauma narrative) *Planned number of intervention sessions*: 12 (23 h) *Mode of delivery (face‐to‐face, online, video, telephone, blend, with explanation)*: face‐to‐face *Format (group, individual, blend)*: group *Therapist qualifications and training appropriate (yes, no, unclear, with explanation)*: yes, all psychosocial assistants had 1 year to 9 years of experience providing case management and individual SC to survivors of sexual violence and at least 4 years of post‐primary school education. All underwent a 5‐day to 6‐day training session conducted by the IRC in case management and specific topics, including counselling, family mediation, stress management, clinical care of survivors and prevention of HIV infection and other sexually transmitted infections. Psychosocial assistants who provided therapy underwent 2 weeks of in‐person training with trainers based in the USA (fourth and fifth authors), with the use of a manual that was adapted and translated locally. Ongoing supervision was provided through a multitiered supervision system. Congolese psychosocial supervisors who were employees of the IRC provided direct supervision to psychosocial assistants through weekly telephone or in‐person meetings; a bilingual clinical social worker trained in the USA provided in‐country supervision and communicated with the US trainers through weekly calls for supervision and quality assurance. *Research allegiance or conflict of interest (yes, no, unclear, with explanation)*: no *Treatment fidelity (yes, no, unclear, with explanation)*: yes, fidelity to the therapy protocol was assessed with the use of checklists of key treatment elements and global ratings of treatment knowledge and skills, as observed by supervisors during group sessions. *Intervention aim and theoretical basis*: CPT is a protocol‐based therapy for treating depression, anxiety, and PTSD in sexual violence survivors. The group format was chosen to reach large numbers of women. They used the cognitive‐only model (i.e. without a trauma narrative) because its efficacy is similar to that of the full version of the therapy, while providing greater ease of administration in groups and greater retention by participants. One individual session (1 h) was held, followed by 11 group sessions (2 h). *Duration of intervention*: 17 weeks Other psychosocial interventions (individual support) *Planned number of intervention sessions*: as desired *Mode of delivery (face‐to‐face, online, video, telephone, blend, with explanation)*: face‐to‐face *Format (group, individual, blend)*: individual *Therapist qualifications and training appropriate (yes, no, unclear, with explanation)*: as previous *Research allegiance or conflict of interest (yes, no, unclear, with explanation)*: no *Treatment fidelity (yes, no, unclear, with explanation)*: yes, monthly visits by IRC supervisors and reviews of interim monitoring forms *Intervention aim and theoretical basis*: psychosocial assistants invited women to receive individual support services as desired, including psychosocial support and economic, medical and legal referrals. Psychosocial assistants were available throughout the treatment period for women who sought their services. *Duration of intervention*: 17 weeks
Outcomes	**PTSD** Outcome type: continuous outcome Scale used: PCL‐C Direction: lower is better Score range: 17 to 85 Data value: endpoint, 6 months **PTSD** Outcome type: dichotomous outcome Scale used: PCL‐C Direction: lower is better Score range: 17 to 85 Data value: endpoint, 6 months **Global mental health** Outcome type: continuous outcome Scale used: Hopkins Symptom Checklist Direction: lower is better Score range: 1 to 4 Data value: endpoint, 6 months **Global mental health** Outcome type: dichotomous outcome Scale used: Hopkins Symptom Checklist Direction: lower is better Score range: 1 to 4 Data value: endpoint, 6 months **Treatment dropout** Outcome type: dichotomous outcome **Stigma** Outcome type: continuous outcome Scale used: Perceived and Internalised Stigma Direction: lower is better Score range: 1 to 4 Data value: endpoint, 6 months
Identification	**Sponsorship source:** funded by the US Agency for International Development Victims of Torture Fund and the World Bank **Country:** Democratic Republic of the Congo **Setting:** community **Authors name:** Judith K Bass **Institution:** Johns Hopkins Bloomberg School of Public Health **Email:** jbass@jhsph.edu **Year:** 2013
Notes	The CPT had no trauma component. Note there is a correction paper as well as the original. Data also taken from Murray 2018 Not included in meta‐analysis

**Table:** Bass 2016

***Study characteristics***	
Methods	**Study design:** randomised controlled trial **Study grouping:** parallel group **Duration of study:** October 2010 to August 2012 (intervention ran from April 2011 to April 2012) **Location of study:** 9 communities in South Kivu, Democratic Republic of the Congo **Randomisation method:** 66 VSLA groups with 301 study women were available for randomisation in March 2011. The 66 groups were randomised into immediate start (intervention; 33 groups) and delayed start (control; 33 groups). The control groups did not receive VSLA training until year 2, when follow‐up data collection was completed. **Ethics approvals:** study protocols were reviewed and approved by IRBs at the Johns Hopkins School of Public Health and Kinshasa School of Public Health.
Participants	**Baseline characteristics** Other psychosocial interventions (VSLAs) *Participants (select at randomisation if available)*: 159 *Gender*: 100% female participants *Age, M (SD)*: 40.1 years (11.7 years) *Ethnicity*: 100% Black *Disability*: not given *Time since trauma*: not given *Income*: not given *Education, M (SD) years*: 2 (3.1) *Employment*: 18.9 h (16.5 h) of paid work in past 7 d *Sexual violence in adulthood*: 54.7% personally experienced sexual violence; 81.1% personally witnessed sexual violence *Comorbid conditions*: not given *Baseline PTSD*: not given *Partnered*: 54.1% *Randomised (N)*: 159 *Completed post‐treatment assessment (N)*: 135 *Dropped out or removed prior to analysis (N)*: 0 *Numbers analysed at final applicable time point (N)*: 159 *Number of sessions*: not given *Treatment completion*: not given Wait‐list *Participants (select at randomisation if available)*: 142 *Gender*: 100% female participants *Age, M (SD)*: 41.5 years (12.8 years) *Ethnicity*: 100% Black *Disability*: not given *Time since trauma*: not given *Income*: not given *Education, M (SD) years*: 1.9 (3.0) *Employment*: 22.5 h (19.9 h) paid work in past 7 d *Sexual violence in adulthood*: 68.3% personally experienced sexual violence; 85.9% personally witnessed sexual violence *Comorbid conditions*: not given *Baseline PTSD*: not given *Partnered*: 53.5% *Randomised (N)*: 142 *Completed post‐treatment assessment (N)*: 115 *Dropped out or removed prior to analysis (N)*: 0 *Numbers analysed at final applicable time point (N)*: 142 *Number of sessions*: not given *Treatment completion*: not given Overall *Participants (select at randomisation if available)*: 301 *Gender*: 100% female participants *Age*: not given *Ethnicity*: 100% Black *Disability*: not given *Time since trauma*: not given *Income*: not given *Education*: not given *Employment*: not given *Sexual violence in adulthood*: 61% *Comorbid conditions*: not given *Baseline PTSD*: not given *Partnered*: 54% *Approached (N)*: 695 *Ineligible (N)*: 236 *Declined (N)*: 2 *Other (N)*: 156 *Randomised (N)*: 301 *Completed post‐treatment assessment (N)*: 250 *Dropped out or removed prior to analysis (N)*: 0 *Numbers analysed at final applicable time point (N)*: 301 *Number of sessions*: N/A *Treatment completion*: N/A **Inclusion criteria** Women aged 18 years of age or older Living in 1 of the 9 study sites Personally experienced or witnessed sexual violence (defined as rape locally) A score of at least 10 on the function assessment (i.e. some dysfunction on at least half of the tasks questions) A score of at least 55 on the mental health assessment (i.e. an average score of 1 for each symptom) **Exclusion criteria:** severe suicidality **Pretreatment:** women in the control group reported more types of traumatic events, were more ethnically diverse, worked more hours, had more people they could rely on, expressed greater group membership and spent more money on food for the household.
Interventions	**Intervention characteristics** Other psychosocial interventions (VSLAs) *Intensity of intervention*: 10 months *Mode of delivery (face‐to‐face, online, video, telephone, blend, with explanation)*: face‐to‐face *Format (group, individual, blend)*: group *Therapist qualifications and training appropriate (yes, no, unclear, with explanation)*: yes, the IRC is experienced at delivering the intervention. *Research allegiance or conflict of interest (yes, no, unclear, with explanation)*: no, none declared *Treatment fidelity (yes, no, unclear, with explanation)*: no, although the programme is described in detail in supplementary materials, there is no information on fidelity to the protocol. *Intervention aim and theoretical basis*: VSLA model was developed by CARE International, based on indigenous savings and loans groups in Africa. VSLAs provide a community‐managed mechanism for savings, loans and insurance for people who cannot access banks or microfinance institutions. It was hypothesised that participation in VSLAs would provide women who lacked access to financial services access to savings and loans in the safety of a trusted group. It was also hypothesised that for women who had experienced sexual violence, participation in the group‐based economic programme would improve their ability to care for themselves and contribute to their family's well‐being, resulting in improved self‐efficacy and a reduction in mental health symptoms; it was hypothesised that women who had experienced sexual violence would improve their social connectedness through being part of the economic group and that this, together with increased economic benefits, would reduce their experience of stigma. Wait‐list *Intensity of intervention*: N/A *Mode of delivery*: N/A *Format*: N/A *Therapist qualifications and training appropriate*: N/A *Research allegiance or conflict of interest*: N/A *Treatment fidelity*: N/A *Intervention aim and theoretical basis*: N/A
Outcomes	**PTSD** Outcome type: continuous outcome Scale used: Harvard Trauma Questionnaire Direction: lower is better Score range: 1 to 4 Data value: endpoint **Depression** Outcome type: continuous outcome Scale used: Hopkins Symptom Checklist Direction: lower is better Score range: 1 to 4 Data value: endpoint **Anxiety** Outcome type: continuous outcome Scale used: Hopkins Symptom Checklist Direction: lower is better Score range: 1 to 4 Data value: endpoint **Stigma** Outcome type: continuous outcome Scale used: Perceived and Internalised Stigma Direction: lower is better Score range: 1 to 4 Data value: endpoint **Global mental health** Outcome type: continuous outcome Scale used: Hopkins Symptom Checklist‐adapted Direction: lower is better Score range: 1 to 4 Data value: endpoint
Identification	**Sponsorship source:** United States Agency for International Development Victims of Torture Fund and the World Bank. Part of the analysis was supported by UK Aid from the UK Department for International Development for the benefit of developing countries. **Country:** Democratic Republic of the Congo **Setting:** women in the community who had sought support or disclosed to community‐based organisations about mental health problems due to potential trauma **Authors name:** Judith K Bass **Institution:** Johns Hopkins School of Public Health **Email:** jbass@jhu.edu **Year:** 2016 **Ethics Approvals:** study protocols were reviewed and approved by IRBs at the Johns Hopkins School of Public Health and Kinshasa School of Public Health.
Notes	This study was included in meta‐analysis Review team selected imputed data, but numbers per group included in analysis were not entirely clear.

**Table:** Bell 2019

***Study characteristics***	
Methods	**Study design:** randomised controlled trial **Study grouping:** parallel group **Duration of study:** not given **Ethics approvals:** Saybrook IRB approved the study **Location of study:** greater Denver‐Boulder, Colorado, USA, area **Randomisation method:** 24 eligible adults were enrolled on a first‐come, first‐served basis and alternately assigned between the LZNF group and HRVB group according to the order in which they returned their prescreening materials.
Participants	**Baseline characteristics** Behavioural (LZNF) *Participants (select at randomisation if available)*: 12 *Gender*: not given *Age, M (SD)*: 43.73 years (8.79 years) *Ethnicity*: not given *Disability*: not given *Time since trauma*: not given *Income*: not given *Education*: not given *Employment*: not given *Sexual violence in adulthood*: not given *Comorbid conditions*: not given *Baseline PTSD, M (SD)*: 46.17 (14.23) *Partnered*: not given *Randomised (N)*: 12 *Completed post‐treatment assessment (N)*: 12 *Dropped out or removed prior to analysis (N)*: 0 *Numbers analysed at final applicable time point (N)*: 12 *Number of sessions*: not given *Treatment completion*: 12 (100%) Minimal intervention (HRVB) *Participants (select at randomisation if available)*: 11 *Gender*: not given *Age, M (SD)*: 44.58 years (13.06 years) *Ethnicity*: not given *Disability*: not given *Time since trauma*: not given *Income*: not given *Education*: not given *Employment*: not given *Sexual violence in adulthood*: not given *Comorbid conditions*: not given *Baseline PTSD, M (SD)*: 49.82 (10.16) *Partnered*: not given *Randomised (N)*: 12 *Completed post‐treatment assessment (N)*: 11 *Dropped out or removed prior to analysis (N)*: 1 *Numbers analysed at final applicable time point (N)*: 11 *Number of sessions*: not given *Treatment completion*: 11 (92%) Overall *Participants (at randomisation)*: 23 *Gender*: not given *Age in years, M (SD)*: 44 *Ethnicity*: not given *Disability*: not given *Time since trauma*: not given *Income*: not given *Education*: not given *Employment*: not given *Sexual violence in adulthood*: 65% *Comorbid conditions*: 91% reported at least 1 comorbid psychiatric disorder; physical assault (61%), childhood abuse or neglect (52%), life‐threatening illness or injury (48%), natural disaster (35%), work‐related trauma (e.g. first responders; 26%) and military combat (13%) *Baseline PTSD*: not given *Partnered*: not given *Approached*: not given *Ineligible*: not given *Declined*: not given *Other*: not given *Randomised (N)*: 24 *Completed post‐treatment assessment (N)*: 23 *Dropped out or removed prior to analysis (N)*: 1 *Numbers analysed at final applicable time point (N)*: 23 **Inclusion criteria** Self‐reported having experienced a traumatic event 6 months or more had passed since the traumatic event Total score on the PCL‐5 was > 20 Proficient in English Between the ages of 18 years and 80 years **Exclusion criteria** Moderate to severe brain injury Current diagnosis of a seizure disorder Current diagnosis of a personality disorder Active psychosis Active suicidal ideation Pregnancy **Pretreatment:** the largest baseline differences were in the HRV measures, for which the LZNF group had higher initial levels. The LZNF group reported a larger number of comorbid diagnoses at baseline (LZNF = 21, HRVB = 15).
Interventions	**Intervention characteristics** Behavioural (LZNF) *Planned number of intervention sessions*: 15 (20 min of direct training, divided into 4 rounds of 5 min) *Mode of delivery (face‐to‐face, online, video, telephone, blend, with explanation)*: brain‐computer interface *Format (group, individual, blend)*: individual *Therapist qualifications and training appropriate*: N/A *Research allegiance or conflict of interest (yes, no, unclear, with explanation)*: no *Treatment fidelity (yes, no, unclear, with explanation)*: yes, as a way of standardising an individualised training modality, NeuroGuide’s Symptom Checklist‐Neural Network Match method was utilised to generate each participant’s training protocol (Thatcher 2013). *Intervention aim and theoretical basis*: the purpose of this study was to assess the effectiveness and specificity of LZNF training, as compared to HRVB training, for reducing mental health symptoms, improving autonomic regulation, and regulating abnormal brainwave activity in adults with chronic PTSD (i.e. symptoms for a minimum of 6 months following a traumatic event). *Duration of intervention*: 8 weeks Minimal intervention (HRVB) *Planned number of intervention sessions*: 15 (20 min of direct training, divided into 4 rounds of 5 min) *Mode of delivery (face‐to‐face, online, video, telephone, blend, with explanation)*: brain‐computer interface *Format (group, individual, blend)*: individual *Therapist qualifications and training appropriate*: N/A *Research allegiance or conflict of interest (yes, no, unclear, with explanation)*: no *Treatment fidelity (yes, no, unclear, with explanation)*: yes, the HRVB protocol for this study was largely based on the resonant frequency training protocol outlined by Lehrer 2000. This is one of the most common protocols in HRVB training and has previously demonstrated effectiveness for reducing PTSD symptoms (Tan 2011). *Intervention aim and theoretical basis*: HRVB training utilises electrocardiography and a respiratory belt, paired with a breath pacer and audiovisual feedback, to train the heart towards healthier levels of HRV. This modality of biofeedback has previously demonstrated effectiveness for reducing PTSD symptoms, even when compared to various control conditions. Here, it was viewed as a more ethical option than sham neurofeedback for this sensitive population. *Duration of intervention*: 8 weeks
Outcomes	**PTSD (PCL‐5)** Outcome type: continuous outcome Scale used: PCL‐5 Direction: lower is better Score range: 0 to 80 Data value: endpoint **Anxiety** Outcome type: continuous outcome Scale used: BAI Direction: lower is better Score range: 0 to 63 Data value: endpoint **Treatment dropout** Outcome type: dichotomous outcome
Identification	**Sponsorship source:** not reported **Country:** USA **Setting:** academic/experimental; individuals with chronic PTSD recruited through advertisements on social media, in health and mental health centres, and in various community locations **Authors name:** Ashlie Bell **Institution:** Saybrook University, Oakland, California **Email:** abell@saybrook.edu **Year:** 2019
Notes	No indication of gender breakdown Included in meta‐analysis Communicated with author for purpose of the review

**Table:** Belleville 2018

***Study characteristics***	
Methods	**Study design:** randomised controlled trial **Study grouping:** parallel group **Duration of study:** 2 years and 3 months; recruitment from March 2013 to January 2015; assessments conducted March to June 2015 **Location of study:** city of Quebec, Canada **Randomisation method:** an independent evaluator who was not involved with assessment or treatment completed randomisation using a random number generator.
Participants	**Baseline characteristics** CBT (IRT + PE) *Participants*: 7 *Gender*: 100% female participants *Age*: not given *Ethnicity*: not given *Disability*: not given *Time since trauma*: not given *Income*: not given *Education*: not given *Employment*: not given *Sexual violence in adulthood*: 100% *Comorbid conditions*: not given *Baseline PTSD, M (SD)*: 65.57 (12.54) *Partnered*: not given *Randomised (N)*: 7 *Completed post‐treatment assessment (N)*: 4 *Dropped out or removed prior to analysis (N)*: 3 *Numbers analysed at final applicable time point (N)*: 4 *Number of sessions*: not given *Treatment completion*: 57% CBT (PE only) *Participants*: 9 *Gender*: 100% female participants *Age*: not given *Ethnicity*: not given *Disability*: not given *Time since trauma*: not given *Income*: not given *Education*: not available *Employment*: not available *Sexual violence in adulthood*: 100% *Comorbid conditions*: not given *Baseline PTSD, M (SD)*: 69.17 (22.14) *Partnered*: not given *Randomised (N)*: 9 *Completed post‐treatment assessment (N)*: 8 *Dropped out or removed prior to analysis (N)*: 1 *Numbers analysed at final applicable time point (N)*: 8 *Number of sessions*: not given *Treatment completion*: 89% Overall *Participants*: 16 *Gender*: 100% female participants *Age*: not given *Ethnicity*: not given *Disability*: not given *Time since trauma*: not given *Income*: not available *Education*: not given *Employment*: not given *Sexual violence in adulthood*: 100% *Comorbid conditions*: not given *Baseline PTSD, M (SD)*: 67.59 (18.10) *Partnered*: not given *Approached*: not given *Ineligible*: not given *Declined:* not given *Other:* not given *Randomised (N)*: 16 *Completed post‐treatment assessment (N)*: 12 *Dropped out or removed prior to analysis (N)*: 4 *Numbers analysed at final applicable time point (N)*: 12 **Inclusion criteria** 18 years and older Able to understand and speak French History of unwanted sexual experience PTSD diagnosis Sleep complaints (score of ≥ 5 and mean of ≥ 1 nightmare per week for at least 1 month on Pittsburgh Sleep Quality Index) Psychotropic medication dosage unchanged for 3 months Available for in‐person assessments and therapy sessions **Exclusion criteria** Past or present psychotic episode, bipolar disorder or organic mental disorder Current substance use disorder Sleep apnoea diagnosis Use of prazosin to treat nightmares Currently in treatment for psychological difficulties Suicidal thoughts requiring immediate intervention Participants had to agree to keep their medication unchanged and not receive any other interventions. **Pretreatment:** none reported
Interventions	**Intervention characteristics** CBT (IRT + PE) *Planned number of intervention sessions*: 20 (5 sessions of IRT followed immediately by 15 sessions of CBT) *Mode of delivery (face‐to‐face, online, video, telephone, blend, with explanation)*: face‐to‐face *Format (group, individual, blend)*: individual *Therapist qualifications and training appropriate (yes, no, unclear, with explanation)*: no (supervised graduate psychology students following manual) *Research allegiance or conflict of interest (yes, no, unclear, with explanation)*: no *Treatment fidelity (yes, no, unclear, with explanation)*: yes, all therapy sessions were filmed, and independent judges reviewed a randomly selected 25% of the videos of completed treatments to evaluate treatment integrity. The evaluation revealed that no elements of IRT were presented in the control condition and that all of the appropriate strategies were presented in each condition. *Intervention aim and theoretical basis*: IRT (designed to alter the conditioned association between nightmares and sleep via rehearsal of a modified dream script during daytime); used French‐language translation of IRT protocol by Nappi 2010; PE protocol (Germain 2009, adapted from Foa 2007) *Duration of intervention*: 20 weeks (5 × 60 min + 15 × 60 min to 90 min) CBT (PE only) *Planned number of intervention sessions*: 15 (15 sessions CBT, which followed 5‐week 'waiting period' that included short (5‐minute) weekly telephone calls to provide support and monitor suicidal thoughts) *Mode of delivery (face‐to‐face, online, video, telephone, blend, with explanation)*: face‐to‐face *Format (group, individual, blend)*: individual *Therapist qualifications and training appropriate (yes, no, unclear, with explanation)*: no (supervised graduate psychology students following manual) *Research allegiance or conflict of interest (yes, no, unclear, with explanation)*: no *Treatment fidelity (yes, no, unclear, with explanation)*: as previous *Intervention aim and theoretical basis*: PE protocol (Germain 2009, adapted from Foa 2007) including psychoeducation, breathing techniques, exposure to traumatic memories, feared objects, activities and situations *Duration of intervention*: 15 weeks (× 60 min to 90 min)
Outcomes	**PTSD** Outcome type: continuous outcome Scale used: MPSS‐SR Direction: lower is better Score range: 0 to 119 Data value: endpoint **Global mental health** Outcome type: continuous outcome Scale used: SF‐36 Mental Direction: higher is better Score range: 0 to 100 Data value: endpoint
Identification	**Sponsorship source:** none **Country:** Canada **Setting:** sexual assault victims seeking treatment, recruited from university, community, rape crisis and mental health organisations, intervention delivered in academic setting **Authors name:** Geneviève Belleville **Institution:** Laval University, Quebec **Email:** genevieve.belleville@psy.ulaval.ca **Year:** 2018
Notes	Review team have reported on the 16 participants who experienced sexual assault at 18+ years supplied by the authors. Data on population characteristics for this subsample were not available to the Review. Communicated with the author(s) for purpose of review This study has not been included in meta‐analysis.

**Table:** Bomyea 2015

***Study characteristics***	
Methods	**Study design:** randomised controlled trial **Study grouping:** parallel group **Duration of study:** not given **Ethics approvals:** approved by the IRBs at the University of California, San Diego and San Diego State University **Location of study:** California, USA **Randomisation method:** individuals who completed all baseline assessments were randomly assigned to the high‐interference control condition or the low‐interference control condition based on a computer‐generated random number system prior to attending the first training session. Conditions were assigned by an independent third party using computer software so that participants and research personnel remained blind to participants’ conditions.
Participants	**Baseline characteristics** Behavioural (high‐interference control) *Participants (select at randomisation if available)*: 22 *Gender*: 100% female participants *Age, M (SD)*: 29.82 years (1.71 years) *Ethnicity*: 73% white, 9% Black, 18% other *Disability*: not given *Time since trauma, M (SD)*: 146.91‐month (159.33‐month) duration of PTSD *Income*: 36% < USD 15,000, 36% USD 20,000 to USD 50,000, 23% > USD 50,000 *Education (% high school, college or apprentice, university or years of education)*: 14% 12 years; 50% 13 years to 15 years; 27% 16 years; 9% > 16 years *Employment*: not given *Sexual violence in adulthood*: 100% *Comorbid conditions*: not given *Baseline PTSD, M (SD)*: 61.41 (12.54) *Partnered*: 19% *Randomised (N)*: 22 *Completed post‐treatment assessment (N)*: 13 *Dropped out or removed prior to analysis (N)*: 0 *Numbers analysed at final applicable time point (N)*: 22 *Number of sessions*: not given *Treatment completion*: 15 (68%) Minimal intervention (low‐interference control) *Participants (select at randomisation if available)*: 20 *Gender*: 100% female participants *Age, M (SD)*: 26.00 years (10.6 years) *Ethnicity*: 65% white, 5% Black, 30% other *Disability*: not given *Time since trauma, M (SD)*: 97.25‐month (113.46‐month) duration of PTSD *Income*: 60% < USD 15,000, 30% USD 20,000 to USD 50,000, 10% > USD 50,000 *Education (% high school, college or apprentice, university or years of education)*: 5% < 12 years, 20% 12 years; 40% 13 years to 15 years; 25% 16 years, 10% > 16 years *Employment*: not given *Sexual violence in adulthood*: 100% *Comorbid conditions*: not given *Baseline PTSD, M (SD)*: 67.35 (15.0) *Partnered*: 47% *Randomised (N)*: 20 *Completed post‐treatment assessment (N)*: 8 *Dropped out or removed prior to analysis (N)*: 0 *Numbers analysed at final applicable time point (N)*: 20 *Number of sessions*: not given *Treatment completion*: 10 (50%) Overall *Participants (select at randomisation if available)*: 42 *Gender*: 100% female participants *Age*: not given *Ethnicity*: 69% white, 7% Black, 24% other *Disability*: not given *Time since trauma*: not given *Income*: 48% < USD 15,000, 33% USD 20,000 to USD 50,000, 17% > USD 50,000 *Education (% high school, college or apprentice, university or years of education)*: 2% < 12 years, 17% 12 years, 45% 13 years to 15 years, 26% 16 years; 10% > 16 years *Employment*: not given *Sexual violence in adulthood*: 100% *Comorbid conditions*: 52.4% major depressive disorder or other mood disorders, 47.6% anxiety disorders, 9.5% substance abuse, 4.8% eating disorders *Baseline PTSD*: not given *Partnered*: 32% *Approached (N)*: 139 *Ineligible (N)*: 15 *Declined (N)*: 2 *Other (N)*: 80 *Randomised (N)*: 42 *Completed post‐treatment assessment (N)*: 21 *Dropped out or removed prior to analysis (N)*: 0 *Numbers analysed at final applicable time point (N)*: 42 *Number of sessions*: not given *Treatment completion*: 25 (60%) **Inclusion criteria** Women between the ages of 18 years and 65 years diagnosed with PTSD secondary to sexual trauma In the case where a participant had experienced multiple traumatic events, she was considered eligible for the study if sexual assault was subjectively considered the most distressing event. Participants taking medications (N = 6) were required to meet a 6‐week stability criterion. English‐language proficiency **Exclusion criteria** Current trauma‐ or PTSD‐focused psychosocial treatment Recent change in non‐trauma‐focused psychosocial treatment Active suicidality (i.e. expression of intent or plan to commit suicidal gestures, or suicide attempt within the past 6 months) Evidence of substance dependence in the past 6 months Evidence of current or past schizophrenia, bipolar disorder or organic mental disorder (individuals with additional diagnoses were not excluded as long as PTSD was the primary diagnosis) **Pretreatment:** participants in the 2 groups did not differ in age or measures of clinical features, including CAPS total severity, duration of PTSD symptoms, number of trauma types experienced, STAI‐Trait, BDI‐II, Self‐Directed Search Questionnaire, or OSpan performance. No differences were identified in participant ethnic background, education, income or marital status.
Interventions	**Intervention characteristics** Behavioural (high‐interference control) *Planned number of intervention sessions*: 8 *Mode of delivery (face‐to‐face, online, video, telephone, blend, with explanation)*: computer trial *Format (group, individual, blend)*: individual *Therapist qualifications and training appropriate*: N/A *Research allegiance or conflict of interest (yes, no, unclear, with explanation)*: yes *Treatment fidelity*: N/A *Intervention aim and theoretical basis*: ability to regulate cognitions is related to PTSD and linked to persistence of distressing and intrusive thoughts. Interference control is a key regulatory process in modulating intrusive cognitions. Performance during working memory capacity tasks is associated with intrusive thoughts during deliberate thought suppression attempts. Interference control training aims to alter the functioning of basic cognitive systems hypothesised to regulate re‐experiencing symptoms. *Duration of intervention*: 4 weeks Minimal intervention (low‐interference control) *Planned number of intervention sessions*: 8 *Mode of delivery (face‐to‐face, online, video, telephone, blend, with explanation)*: computer trial *Format (group, individual, blend)*: individual *Therapist qualifications and training appropriate*: N/A *Research allegiance or conflict of interest (yes, no, unclear, with explanation)*: yes *Treatment fidelity*: N/A *Intervention aim and theoretical basis*: control programme *Duration of intervention*: 4 weeks
Outcomes	**PTSD** Outcome type: continuous outcome Scale used: CAPS Direction: lower is better Score range: 0 to 80 Data value: endpoint Depression Outcome type: continuous outcome Scale used: BDI Direction: lower is better Score range: 0 to 63 Data value: endpoint **Anxiety** Outcome type: continuous outcome Scale used: STAI‐Trait Direction: lower is better Score range: 20 to 80 Data value: endpoint **PTSD** Outcome type: continuous outcome Scale used: CAPS‐Avoidance Direction: lower is better Score range: 0 to 28 Data value: endpoint **PTSD** Outcome type: continuous outcome Scale used: CAPS‐Arousal Direction: lower is better Score range: 0 to 28 Data value: endpoint **Treatment dropout** Outcome type: dichotomous outcome
Identification	**Sponsorship source:** NIMH F31 MH0881704 **Country:** USA **Setting:** intervention delivered in academic setting; women diagnosed with PTSD secondary to sexual trauma recruited through university participant pool and community mental health providers **Authors name:** Jessica A Bomyea **Institution:** University of California **Email:** jbomyea@ucsd.edu **Year:** 2015
Notes	Uses imputed data in ITT analyses Communicated with the author(s) for purpose of review Included in meta‐analysis

**Table:** Bowland 2012

***Study characteristics***	
Methods	**Study design:** randomised controlled trial **Study grouping:** parallel group **Duration of study:** 2 years: recruitment February 2006 to July 2007; intervention July 2006 to January 2008 **Ethics approvals:** Human Research Protection Office, Washington University in St. Louis, Missouri **Location of study:** St. Louis, Missouri, USA **Randomisation method:** women were paired on scores from a spiritual distress scale and then randomised into treatment or control groups using a random number table.
Participants	**Baseline characteristics** Other psychologically oriented interventions (spiritually focused group therapy) *Participants (completers)*: 21 *Gender*: 100% female participants *Age, M (SD)*: 60.33 years (4.6 years) *Ethnicity*: not given *Disability*: not given *Time since trauma*: not given *Income*: 44% < USD 50,000 *Education (% high school, college or apprentice, university or years of education)*: 48% college *Employment*: not given *Sexual violence in adulthood*: 48% *Comorbid conditions*: not given *Baseline PTSD, M (SD)*: 19.43 (9.65) *Partnered*: 50% *Randomised (N)*: 22 *Completed post‐treatment assessment (N)*: 21 *Dropped out or removed prior to analysis (N)*: 1 *Numbers analysed at final applicable time point (N)*: 21 *Number of sessions*: not given *Treatment completion*: not given No‐treatment control *Participants (completers)*: 22 *Gender*: 100% female participants *Age, M (SD)*: 62.32 years (7.7 years) *Ethnicity*: not given *Disability*: not given *Time since trauma*: not given *Income*: 56% < USD 50,000 *Education (% high school, college or apprentice, university or years of education)*: 52% college *Employment*: not given *Sexual violence in adulthood*: 50% *Comorbid conditions*: not given *Baseline PTSD, M (SD)*: 16.10 (9.32) *Partnered*: 50% *Randomised (N)*: 22 *Completed post‐treatment assessment (N)*: 22 *Dropped out or removed prior to analysis (N)*: 0 *Numbers analysed at final applicable time point (N)*: 22 *Number of sessions*: not given *Treatment completion*: not given Overall *Participants (completers)*: 43 *Gender*: 100% female participants *Age, M (range)*: 61.3 years (55 years to 83 years) *Ethnicity*: 84% white, 14% Black, 2% other *Disability*: not given *Time since trauma*: not given *Income*: 65% < USD 50,000 *Education (% high school, college or apprentice, university or years of education)*: 61% associate or bachelor degree *Employment*: not given *Sexual violence in adulthood*: 49% sexual violence in adulthood; all participants reported > 1 traumatic event (child sexual or physical abuse or both, domestic violence, sexual assault) *Comorbid conditions*: not given *Baseline PTSD*: not given *Partnered*: 52% *Approached (N)*: 129 *Ineligible (N)*: 60 *Declined (N)*: 0 *Other (N)*: 25 *Randomised (N)*: 44 *Completed post‐treatment assessment (N)*: 43 *Dropped out or removed prior to analysis (N)*: 1 *Numbers analysed at final applicable time point (N)*: 43 *Number of sessions*: N/A *Treatment completion*: N/A **Inclusion criteria** Current trauma‐related distress History in the Christian tradition **Exclusion criteria** In current psychotherapy Significant cognitive impairments Actively suicidal or psychotic **Pretreatment:** none reported
Interventions	**Intervention characteristics** Other psychologically oriented interventions (spiritually focused group therapy) *Planned number of intervention sessions*: 11 *Mode of delivery (face‐to‐face, online, video, telephone, blend, with explanation)*: face‐to‐face *Format (group, individual, blend)*: group *Therapist qualifications and training appropriate (yes, no, unclear, with explanation)*: yes *Research allegiance or conflict of interest (yes, no, unclear, with explanation)*: unclear, as Fallot designed intervention as an offshoot of the Trauma Recovery and Empowerment Model *Treatment fidelity (yes, no, unclear, with explanation)*: yes, mean adherence score for 18 sessions was 83.3% (SD = 19.40%; range 40% to 100%) *Intervention aim and theoretical basis*: manualised psychoeducational, CR and skill‐building approach to addressing spiritual struggles in recovery (Fallot and The Spirituality Workgroup 2001‐2004). Offshoot of the Trauma Recovery and Empowerment Model (Harris 1998). Feminist perspective used to raise questions, for instance, about endurance and sacrifice of self for others, based on a critique of patriarchal Christianity and potentially harmful interpretations and practices (see Brock 2001; Jonker 1992) *Duration of intervention*: 11 weeks No‐treatment control *Planned number of intervention sessions*: N/A *Mode of delivery*: N/A *Format*: N/A *Therapist qualifications and training appropriate*: N/A *Research allegiance or conflict of interest*: N/A *Treatment fidelity*: N/A *Intervention aim and theoretical basis*: N/A *Duration of intervention*: N/A
Outcomes	**Treatment dropout (intervention group only)** Outcome type: dichotomous outcome **Depression** Outcome type: continuous outcome Scale used: Geriatric Depression Scale Direction: lower is better Score range: 0 to 30 Data value: endpoint **Anxiety** Outcome type: continuous outcome Scale used: BAI Direction: lower is better Score range: 0 to 63 Data value: endpoint
Identification	**Sponsorship source:** not given **Country:** USA **Setting:** older survivors recruited through community and university hospital; academic setting for delivery **Authors name:** Sharon Bowland **Institution:** Washington University in St. Louis **Email:** sbowland@utk.edu **Year:** 2012
Notes	Population characteristics are reported for intervention completers. The Posttraumatic Diagnostic Scale data were not given for the control group. Communicated with the author(s) for purpose of review Included in meta‐analysis

**Table:** Brady 2021

***Study characteristics***	
Methods	**Study design:** randomised controlled trial **Study grouping:** parallel group **Duration of study:** recruitment May 2017 and September 2018 **Location of study:** UK **Randomisation method:** after diagnostic assessment and completion of baseline outcome measures, a research assistant randomised participants to either trial condition using a virtual coin toss programme. **Ethics approvals:** the study was approved by the ethics committee of University College London (813/002).
Participants	**Baseline characteristics** Behavioural (NET) *Participants*: 15 *Gender*: 73% female participants, 4 male participants *Age, M (SD)*: 26.73 years (9.35 years) *Ethnicity*: 47% African, 53% other *Disability*: not given *Time since trauma*: not given *Income*: not given *Education*: not given *Employment*: not given *Sexual violence in adulthood*: 53% *Comorbid conditions*: not given *Baseline PTSD, M (SD)*: 43 (34) *Partnered*: not given *Randomised (N)*: 15 *Completed post‐treatment assessment (N)*: 13 *Dropped out or removed prior to analysis (N)*: 2 *Numbers analysed at final applicable time point (N)*: 13 *Number of sessions, M (SD)*: 17 (4.07) *Treatment completion*: 13 (87%) Minimal intervention (psychoeducation + wait‐list) *Participants*: 10 *Gender*: 80% female participants, 2 male participants *Age, M (SD)*: 32.8 years (10.96 years) *Ethnicity*: 20% African, 80% other *Disability*: not given *Time since trauma*: not given *Income*: not given *Education*: not given *Employment*: not given *Sexual violence in adulthood*: 70% *Comorbid conditions*: not given *Baseline PTSD, M (SD)*: 46 (25) *Partnered*: not given *Randomised (N)*: 10 *Completed post‐treatment assessment (N)*: 7 *Dropped out or removed prior to analysis (N)*: 3 *Numbers analysed at final applicable time point (N)*: 7 *Number of sessions*: N/A *Treatment completion*: N/A Overall *Participants*: 25 *Gender*: 76% female participants, 24% male participants *Age*: not given *Ethnicity*: 36% African, 66% other *Disability*: not given *Time since trauma*: not given *Income*: not given *Education*: not given *Employment*: not given *Sexual violence in adulthood*: 60% *Comorbid conditions*: not given *Baseline PTSD, M (SD)*: 43 (17) *Partnered*: 45.1% *Approached (N)*: 55 Ineligible *(N)*: 20 *Declined (N)*: 9 *Other (N)*: 1 *Randomised (N)*: 25 *Completed post‐treatment assessment (N)*: 20 *Dropped out or removed prior to analysis (N)*: 5 *Numbers analysed at final applicable time point (N)*: 20 *Number of sessions*: N/A *Treatment completion*: N/A **Inclusion criteria** Adult survivors of trafficking meeting DSM‐5 diagnostic criteria for PTSD Not having received any type of trauma‐focused therapy previously Willing to engage in NET **Exclusion criteria** Recent suicide attempt or persistent severe self‐harm Significant and frequent substance misuse Severe preoccupation with social or legal issues that would interfere with engagement in regular therapy sessions Facing imminent removal from the UK Currently in a situation of abuse or exploitation **Pretreatment:** randomisation did not yield equal allocation across the 2 groups. There were also some notable group differences in the distribution of other participant characteristics, including whether the participant was an English speaker, and in terms of country of origin. Those in the wait‐list group reported more dissociative symptoms.
Interventions	**Intervention characteristics** Behavioural (NET) *Intensity of intervention (sessions)*: 90 min to 120 min × 20 *Mode of delivery (face‐to‐face, online, video, telephone, blend, with explanation)*: face‐to‐face *Format (group, individual, blend)*: individual *Therapist qualifications and training appropriate (yes, no, unclear, with explanation)*: yes, NET sessions were delivered by 7 female psychological therapists (6 clinical psychologists and 1 psychotherapist); all had training and prior experience in working with survivors of trafficking and in delivering NET (trained by FB, EW and KR and supervised by FB and EW). *Research allegiance or conflict of interest (yes, no, unclear, with explanation)*: no, declaration of interest considered/made and does not appear to affect allegiance to the therapy *Treatment fidelity (yes, no, unclear, with explanation)*: yes, treatment fidelity was monitored through the recording of therapy sessions and regular case supervision by clinical psychologists with extensive experience of NET. *Intervention aim and theoretical basis*: as directed by the NET manual, the majority of time in sessions was spent on facilitation of the participant’s detailed narration of traumatic experiences to ‘process’ the trauma memory, facilitate attachment repair and make meaning of traumatic and adverse experiences. Where time permitted, positive life events were also explored. A written narrative was created following the therapy sessions; this was read back and given to participants in the final therapy session. Through detailed narration, NET processes and contextualises traumatic memories and helps individuals to establish a coherent autobiographical narrative of their experiences. *Duration of intervention*: 20 weeks Minimal intervention (psychoeducation + wait‐list) *Intensity of intervention (sessions)*: 3 *Mode of delivery (face‐to‐face, online, video, telephone, blend, with explanation)*: face‐to‐face *Format (group, individual, blend)*: individual *Therapist qualifications and training appropriate (yes, no, unclear, with explanation)*: psychoeducation sessions were delivered by NET therapists and trained assistant psychologists. *Research allegiance or conflict of interest*: N/A *Treatment fidelity (yes, no, unclear, with explanation)*: interpreters were used where required. The study used protocolised psychoeducation about PTSD symptoms and symptom management strategies. *Intervention aim and theoretical basis*: irrespective of group, each participant was initially offered 3 sessions of protocolised psychoeducation about PTSD symptoms and symptom management strategies. *Duration of intervention*: N/A
Outcomes	**PTSD** Outcome type: continuous outcome Scale used: Clinician‐Administered PTSD Scale Direction: lower is better Score range: 0 to 80 Data value: endpoint, post‐treatment, 6 months and 12 months (NET only) **PTSD** Outcome type: continuous outcome Scale used: PCL‐5 Direction: lower is better Score range: 0 to 80 Data value: endpoint, post‐treatment **Depression** Outcome type: continuous outcome Scale used: Patient Health Questionnaire Direction: lower is better Score range: 0 to 27 Data value: endpoint, post‐treatment **Anxiety** Outcome type: continuous outcome Scale used: Generalized Anxiety Disorder Scale Direction: lower is better Score range: 0 to 21 Data value: endpoint, post‐treatment **Dissociation** Outcome type: continuous outcome Scale used: Shutdown Dissociation Scale Direction: lower is better Score range: 0 to 39 Data value: endpoint, post‐treatment **Treatment dropout** Outcome type: dichotomous outcome
Identification	**Sponsorship source:** this project was supported by a grant from the Oak Foundation (ref: OCAY‐15‐286). **Country:** UK **Setting:** people seeking care post‐trafficking at either a charity or other support services **Authors name:** Francesca Brady **Institution:** The Helen Bamber Foundation, London, UK; Woodfield Trauma Service, Central and North West London NHS Foundation Trust, London, UK; and Department of Clinical, Educational and Health Psychology, University College London, UK **Email:** francesca@helenbamber.org **Year:** 2021
Notes	Data obtained from author

**Table:** Covers 2021

***Study characteristics***	
Methods	**Study design:** randomised controlled trial **Study grouping:** parallel group **Duration of study:** recruitment between March 2018 and January 2020 **Location of study:** the Netherlands **Randomisation method:** per centre (4), a randomisation sequence was computer‐generated. Service users were randomly assigned to 1 of the 2 study conditions on a 1:1 ratio: EMDR therapy or TAU. **Ethics approvals:** the study was approved by the Medical Ethical Committee of the University Medical Centre Utrecht.
Participants	**Baseline characteristics** Behavioural (EMDR) *Participants (at randomisation)*: 29 *Gender*: 100% female participants *Age, M (SD)*: 25.52 years (7.93 years) *Ethnicity*: not given *Disability*: not given *Time since trauma, M (SD)*: 2 weeks to 4 weeks post‐rape *Income*: not given *Education*: not given *Employment*: not given *Sexual violence in adulthood*: 100% *Comorbid conditions*: not given *Baseline PTSD, M (SD)*: 45.69 (13.45) *Partnered*: not given *Randomised (N)*: 29 *Completed post‐treatment assessment (N)*: 23 *Dropped out or removed prior to analysis (N)*: 6 *Numbers analysed at final applicable time point (N)*: 20 *Number of sessions*: not given *Treatment completion*: 26 (90%) TAU *Participants (at randomisation)*: 28 *Gender*: 95% female participants (1 male participant) *Age, M (SD)*: 25.88 years (8.23 years) *Ethnicity*: not given *Disability*: not given *Time since trauma, M (SD)*: 2 weeks to 4 weeks post‐rape *Income*: not given *Education*: not given *Employment*: not given *Sexual violence in adulthood*: 100% *Comorbid conditions*: not given *Baseline PTSD, M (SD)*: 47.84 (13.65) *Partnered*: not given *Randomised (N)*: 28 *Completed post‐treatment assessment (N)*: 20 *Dropped out or removed prior to analysis (N)*: 8 *Numbers analysed at final applicable time point (N)*: 17 *Number of sessions*: not given *Treatment completion*: 22 (79%) Overall *Participants (at randomisation)*: 57 *Gender*: 98% female participants (1 male participant) *Age, M (SD)*: 25.81 years (8.18 years) *Ethnicity*: not given *Disability*: not given *Time since trauma*: 2 weeks to 4 weeks post‐rape *Income*: not given *Education*: not given *Employment*: not given *Sexual violence in adulthood*: 100% *Comorbid conditions*: not given *Baseline PTSD, M (SD)*: 46 (13.5) *Partnered*: not given *Approached (N)*: 796 *Ineligible (N)*: 475 *Declined (N)*: 58 *Other (N)*: 206 *Randomised (N)*: 57 *Completed post‐treatment assessment (N)*: 43 *Dropped out or removed prior to analysis (N)*: 14 *Numbers analysed at final applicable time point (N)*: 37 *Number of sessions*: not given *Treatment completion*: 48 (84%) **Inclusion criteria** Victims of rape who contacted 1 of the participating sexual assault centres within 1 week after they experienced rape **Exclusion criteria** Those younger than 16 years Those with cognitive disabilities Non‐Dutch‐speaking Those who required immediate psychological care for psychoses, suicidal ideation or addiction Those who received concurrent trauma‐focused treatment for prior experiences **Pretreatment:** some imbalance at baseline in prior trauma and prior trauma‐based treatment but not on study measures, gender or age
Interventions	**Intervention characteristics** Behavioural (EMDR) *Intensity of intervention (sessions)*: 3.5 h of therapy across 2 sessions *Mode of delivery (face‐to‐face, online, video, telephone, blend, with explanation)*: face‐to‐face *Format (group, individual, blend)*: individual *Therapist qualifications and training appropriate (yes, no, unclear, with explanation)*: yes. Therapists were 8 licenced psychologists who completed an accredited course of EMDR therapy and were trained in the application of the study protocol. *Research allegiance or conflict of interest (yes, no, unclear, with explanation)*: no indication of risk of allegiance or conflict of interest *Treatment fidelity (yes, no, unclear, with explanation)*: yes. Video recordings of the sessions were made for bimonthly supervision by 2 EMDR Europe‐accredited trainers in EMDR therapy. An EMDR‐specific treatment integrity checklist was used. Twenty‐five per cent of the 48 video recordings were assessed by 2 independent psychology graduates. There were no inconsistencies in these 2 assessments, and treatment fidelity proved high (97%). *Intervention aim and theoretical basis*: the trauma memory was conceptualised as a memory lasting from a period of time from the rape until the day of treatment. *Duration of intervention*: 2 weeks TAU (other psychosocial interventions) *Intensity of intervention (sessions)*: 2 × 30 min *Mode of delivery (face‐to‐face, online, video, telephone, blend, with explanation)*: telephone *Format (group, individual, blend)*: individual *Therapist qualifications and training appropriate (yes, no, unclear, with explanation)*: yes, a case manager based at the sexual assault centre *Research allegiance or conflict of interest (yes, no, unclear, with explanation)*: no *Treatment fidelity (yes, no, unclear, with explanation)*: unclear but anticipated to meet standard practice at the centres *Intervention aim and theoretical basis*: psychoeducation and emotional support in accordance with a watchful waiting protocol, which stipulates screening for post‐traumatic stress symptoms at least 2 times during the first month post‐rape and, if indicated, subsequent referral for evidence‐based treatment *Duration of intervention*: 2 weeks
Outcomes	**PTSD** Outcome type: continuous outcome Scale used: PCL‐5 Direction: lower is better Score range: 0 to 80 Data value: endpoint, post‐treatment, and 8 weeks and 12 weeks post‐assault **Depression** Outcome type: continuous outcome Scale used: HADS‐Depression Scale Direction: lower is better Score range: 0 to 21 Data value: endpoint, post‐treatment, and 8 and 12 weeks post‐assault **Anxiety** Outcome type: continuous outcome Scale used: HADS‐Anxiety Scale Direction: lower is better Score range: 0 to 21 Data value: endpoint, post‐treatment, and 8 and 12 weeks post‐assault **Global mental health functioning/distress** Outcome type: continuous outcome Scale used: BSI Direction: lower is better Score range: 0 to 112 Data value: endpoint, post‐treatment, and 8 and 12 weeks post‐assault **Dissociation** Outcome type: continuous outcome Scale used: Dissociation Tension Scale Direction: lower is better Score range: 0 to 100 Data value: endpoint, post‐treatment, and 8 and 12 weeks post‐assault **Pelvic floor** Outcome type: continuous outcome Scale used: Amsterdam Overactive Pelvic Floor Scale for Women Direction: lower is better Score range: 4 to 20 Data value: endpoint, 12 weeks post‐assault **Sexual function** Outcome type: continuous outcome Scale used: Female Sexual Functioning Index total score and Desire and Satisfaction Direction: higher is better Score range: 2 to 36 Data value: endpoint, 12 weeks post‐assault **Guilt and shame** Outcome type: continuous outcome Scale used: items based on Foa 1991 Direction: lower is better Data value: endpoint, 12 weeks post‐assault **Treatment dropout** Outcome type: dichotomous outcome
Identification	**Sponsorship source:** the work was supported by the Achmea Association Victims & Society, Innovatiefonds Zorgverzekeraars, EMDR Research Foundation, Vereniging EMDR Nederland and PAOS fonds. **Country:** the Netherlands **Setting:** sexual assault centres **Authors name:** Milou Covers **Institution:** National Psychotrauma Center for Children and Youth, University Medical Center Utrecht, Utrecht, the Netherlands **Email:** m.l.v.covers@umcutrecht.nl **Year:** 2021
Notes	Time of assessment was defined as pretreatment (Pre), post‐treatment (Post), follow‐up at 8 weeks post‐rape and follow‐up at 12 weeks post‐assault.

**Table:** Creech 2021

***Study characteristics***	
Methods	**Study design:** randomised controlled trial **Study grouping:** parallel group **Duration of study:** May 2017 to April 2019 **Location of study:** Texas, USA **Randomisation method:** after completion of the baseline assessment, the (computer) narrator “flipped a coin” and women (N = 153) were randomised into the control or SHE intervention. The randomisation sequence was known only to the computer program and optimised for balanced assignment over time between the 2 conditions. **Ethics approvals:** the study was approved by the IRB.
Participants	**Baseline characteristics** Other psychosocial interventions (SHE) *Participants*: 76 *Gender*: 100% female participants *Age, M (SD)*: 43.46 years (9.97 years) *Ethnicity*: 36.8% white, 47.4% African American, 15.8% other *Disability*: not given *Time since trauma*: not given *Income*: not given *Education (% high school, college or apprentice, university or years of education)*: 2.6% high school, 13.2% college or apprentice, 40.8% some college, 43.4% university *Employment*: not given *Sexual violence in adulthood*: 100% *Comorbid conditions*: 28.9% hazardous drinking, 55.2% intimate partner violence *Baseline PTSD, M (SD)*: 51.91 (16.95) *Partnered*: 39.5% *Randomised (N)*: 76 *Completed post‐treatment assessment (N)*: 64 *Dropped out or removed prior to analysis (N)*: 12 *Numbers analysed at final applicable time point (N)*: 66 *Number of sessions*: N/A *Treatment completion*: 61 (80%) Assessment control *Participants*: 77 *Gender*: 100% female participants *Age, M (SD)*: 43.63 years (10.28 years) *Ethnicity*: 33.8% white, 48.1% African American, 18.1% other *Disability*: not given *Time since trauma*: not given *Income*: not given *Education (% high school, college or apprentice, university or years of education)*: 13% high school, 2.6% college or apprentice, 40.3% some college, 44.2% university *Employment*: not given *Sexual violence in adulthood*: 97.4% *Comorbid conditions*: 28.9% hazardous drinking, 54.5% intimate partner violence *Baseline PTSD, M (SD)*: 49.71 (17.44) *Partnered*: 48.1% *Randomised (N)*: 77 *Completed post‐treatment assessment (N)*: 67 *Dropped out or removed prior to analysis (N)*: 10 *Numbers analysed at final applicable time point (N)*: 69 *Number of sessions*: N/A *Treatment completion*: N/A Overall *Participants*: 153 *Gender*: 100% female participants *Age, M (SD)*: 43.55 years (10.10 years) *Ethnicity*: 35.3% white, 47.7% African American, 17% other *Disability*: not given *Time since trauma*: not given *Income*: not given *Education (% high school, college or apprentice, university or years of education)*: 7.8% high school, 7.8% college or apprentice, 40.5% some college, 43.8% university *Employment*: not given *Sexual violence in adulthood*: 100% *Comorbid conditions*: 28.9% hazardous drinking, 55% intimate partner violence *Baseline PTSD, M (SD)*: 50 (17) *Partnered*: 45.1% *Approached (N)*: 2309 Ineligible *(N)*: 114 *Declined (N)*: 60 *Other (N)*: 1982 *Randomised (N)*: 153 *Completed post‐treatment assessment (N)*: 131 *Dropped out or removed prior to analysis (N)*: 22 *Numbers analysed at final applicable time point (N)*: 135 *Number of sessions*: N/A *Treatment completion*: N/A **Inclusion criteria** Self‐identified female gender Aged between 18 years and 65 years History of sexual assault (defined as at least 1 incident of unwanted lifetime sexual contact) At least 1 current psychosocial health risk (PTSD, hazardous drinking or intimate partner violence) **Exclusion criteria** Inability to understand study procedures in English Active suicidal or homicidal crisis warranting imminent clinical intervention **Pretreatment:** at baseline, the group difference in number of risks was statistically significant, with those in the SHE group reporting a higher number of risks.
Interventions	**Intervention characteristics** Other psychosocial interventions (SHE) *Intensity of intervention (sessions)*: 1 × 20 min *Mode of delivery (face‐to‐face, online, video, telephone, blend, with explanation)*: computer *Format (group, individual, blend)*: individual *Therapist qualifications and training appropriate*: N/A *Research allegiance or conflict of interest*: N/A *Treatment fidelity*: N/A *Intervention aim and theoretical basis*: the SHE for women to address barriers to screening for and addressing lifetime sexual assault and related psychosocial health risks of PTSD, hazardous drinking and intimate partner violence. SHE is a modular computer‐based screen and brief intervention relying on psychoeducation and the principles of motivational interviewing to reduce health risks in women with lifetime sexual trauma histories. *Duration of intervention*: 1 week Assessment control *Intensity of intervention (sessions)*: N/A *Mode of delivery*: N/A *Format*: N/A *Therapist qualifications and training appropriate*: N/A *Research allegiance or conflict of interest*: N/A *Treatment fidelity*: N/A *Intervention aim and theoretical basis*: those randomised to the control condition completed assessments only. After baseline, they were offered a list of mental health and intimate partner violence referrals and resources. They were assisted with referrals directly any time throughout the study if requested. *Duration of intervention*: N/A
Outcomes	**PTSD** Outcome type: continuous outcome Scale used: PCL‐5 Direction: lower is better Score range: 0 to 80 Data value: endpoint, 2 months and 4 months **Intimate partner violence** Outcome type: continuous outcome Scale used: Composite Abuse Scale Direction: lower is better Score range: 0 to 150 Data value: endpoint, 2 months and 4 months **Hazardous drinking** Outcome type: continuous outcome Scale used: AUDIT Direction: lower is better Score range: 0 to 40 Data value: endpoint, 2 months and 4 months **Treatment use** Outcome type: continuous outcome Scale used: Treatment Services Review Direction: lower scores mean less treatment use Score range: N/A (measure provides information on the type, amount and efficacy of services provided) Data value: endpoint, 2 months and 4 months **Treatment dropout** Outcome type: dichotomous outcome (intervention group only)
Identification	**Sponsorship source:** this work was supported by a grant from the Department of Defense, W81XWH‐14‐1‐0368. ClinicalTrials.gov identifier: NCT02957747. This work was also supported by additional funds and resources from the VHA VISN 17 Center of Excellence and the Central Texas VA Health Care System. **Country:** USA **Setting:** veterans seeking primary care at a VHA medical centre. The study was advertised via fliers, in‐person recruitment in women’s primary care clinics and letters to all women who had primary care appointments scheduled in the next month. **Authors name:** Suzannah Creech **Institution:** Center of Excellence for Research on Returning War Veterans and the Central Texas Veterans Health Care System **Email:** Suzannah.Creech@va.gov **Year:** 2021
Notes	—

**Table:** Echeburua 1996

***Study characteristics***	
Methods	**Study design:** randomised controlled trial **Study grouping:** parallel group **Duration of study:** 5 years (recruitment April 1989 to March 1993) **Ethics approvals:** not given **Location of study:** San Sebastian, Bilbao and Vitoria, Spain **Randomisation method:** assignment of participants to 1 of the 2 experimental conditions was carried out randomly in order of arrival at the Psychological Counselling Centers for Women
Participants	**Baseline characteristics** CBT (CR + coping skills) *Participants (at randomisation)*: 10 *Gender*: 100% female participants *Age, M*: 24 years *Ethnicity*: not given *Disability*: not given *Time since trauma, M*: 1 month *Income*: not given *Education (% high school, college or apprentice, university or years of education)*: 30% high school, 20% college *Employment*: not given *Sexual violence in adulthood*: 100% *Comorbid conditions*: not given *Baseline PTSD, M (SD)*: 36.7 (8.59) *Partnered*: 20% *Randomised (N)*: 10 *Completed post‐treatment assessment (N)*: 10 *Dropped out or removed prior to analysis (N)*: 0 *Numbers analysed at final applicable time point (N)*: 10 *Number of sessions, M (SD)*: 5 (0) *Treatment completion, N (SD)*: 10 (100%) Behavioural (progressive relaxation) *Participants (at randomisation)*: 10 *Gender*: 100% female participants *Age, M*: 20 years *Ethnicity*: not given *Disability*: not given *Time since trauma, M*: 1 month *Income*: not given *Education (% high school, college or apprentice, university or years of education)*: 30% high school, 10% college *Employment*: not given *Sexual violence in adulthood*: 100% *Comorbid conditions*: not given *Baseline PTSD, M (SD)*: 34.3 (7.54) *Partnered*: 10% *Randomised (N)*: 10 *Completed post‐treatment assessment (N)*: 10 *Dropped out or removed prior to analysis (N)*: 0 *Numbers analysed at final applicable time point (N)*: 10 *Number of sessions, M (SD)*: 5 (0) *Treatment completion*: 10 (100%) Overall *Participants (at randomisation)*: 20 *Gender*: 100% female participants *Age, M (SD)*: 22 years (6.9 years) *Ethnicity*: not given *Disability*: not given *Time since trauma, M*: 1 month *Income*: not given *Education (% high school, college or apprentice, university or years of education)*: 30% high school, 15% college *Employment*: not given *Sexual violence in adulthood*: 100% *Comorbid conditions*: not given *Baseline PTSD, M (SD)*: 35.5 (7.96) *Partnered*: 15% *Approached*: 31 *Ineligible (N)*: 11 *Declined (N)*: 0 *Other (N)*: 0 *Randomised (N)*: 20 *Completed post‐treatment assessment (N)*: 20 *Dropped out or removed prior to analysis (N)*: 0 *Numbers analysed at final applicable time point (N)*: 20 *Number of sessions*: N/A *Treatment completion*: 20 (100%) **Inclusion criteria** Experienced sexual aggression Met DSM‐III‐R criteria for PTSD Within 3 months of sexual aggression **Exclusion criteria:** suffering severe physical or mental disorder **Pretreatment:** none reported
Interventions	**Intervention characteristics** CBT (CR + coping skills) *Planned number of intervention sessions*: 5 (× 1 h) *Mode of delivery (face‐to‐face, online, video, telephone, blend, with explanation)*: face‐to‐face *Format (group, individual, blend)*: individual *Therapist qualifications and training appropriate (yes, no, unclear, with explanation)*: yes, the assessment and therapy were tried with 5 people before the study. The intervention was delivered by a clinical psychologist with 5 years of experience in cognitive and behavioural treatment of victims of sexual violence. *Research allegiance or conflict of interest (yes, no, unclear, with explanation)*: no *Treatment fidelity (yes, no, unclear, with explanation)*: unclear, not given *Intervention aim and theoretical basis*: first, explaining the normal reaction to sexual aggression and process of developing and maintaining fear; secondly, modifying negative thoughts with more adaptive ones. Traumatic event was resituated in its appropriate dimensions. Positive aspects of the new situation are pointed out. Coping skills training included progressive relaxation (Bernstein 1973), thought‐stopping, cognitive distractions and instruction in gradual exposure in order to resume habitual activities. *Duration of intervention*: 5 weeks Behavioural (progressive relaxation) *Planned number of intervention sessions*: 5 (× 1 h) *Mode of delivery (face‐to‐face, online, video, telephone, blend, with explanation)*: face‐to‐face *Format (group, individual, blend)*: individual *Therapist qualifications and training appropriate (yes, no, unclear, with explanation)*: as previous *Research allegiance or conflict of interest (yes, no, unclear, with explanation)*: no *Treatment fidelity (yes, no, unclear, with explanation)*: unclear, not given *Intervention aim and theoretical basis*: first, general instruction about the psychological impact of sexual aggression; second, training in progressive muscular relaxation (Bernstein 1973) *Duration of intervention*: 5 weeks
Outcomes	**Depression** Outcome type: continuous outcome Scale used: BDI Direction: lower is better Score range: 0 to 63 Data value: endpoint, 6 months and 12 months **Treatment dropout** Outcome type: dichotomous outcome **PTSD** Outcome type: continuous outcome Scale used: Scale of Severity of Posttraumatic Stress Disorder Symptoms Direction: lower is better Score range: 0 to 51 Data value: endpoint, 6 months and 12 months **Anxiety** Outcome type: continuous outcome Scale used: STAI Direction: lower is better Score range: 20 to 80 Data value: endpoint, 6 months and 12 months **PTSD** Outcome type: continuous outcome Scale used: SSS Reexperience Direction: lower is better Score range: 0 to 5 Data value: endpoint, 6 months and 12 months **PTSD** Outcome type: continuous outcome Scale used: SSS Avoidance Direction: lower is better Score range: 0 to 2 Data value: endpoint, 6 months and 12 months **PTSD** Outcome type: continuous outcome Scale used: SSS Arousal Direction: lower is better Score range: 0 to 3 Data value: endpoint, 6 months and 12 months **Trauma‐related beliefs** Outcome type: continuous outcome Scale used: Mental Fatigue Scale‐III Direction: lower is better Score range: 120 to 600 (rape section: 42 to 210) Data value: endpoint, 6 months and 12 months
Identification	**Sponsorship source:** University of the Basque Country UPV 006.230‐0106/88 **Country:** Spain **Setting:** women seeking support following sexual victimisation at community counselling centres for women **Authors name:** Enrique Echeburúa **Institution:** Universidad del País Vasco **Year:** 1996
Notes	Not included in meta‐analysis

**Table:** Falsetti 2008

***Study characteristics***	
Methods	**Study design:** randomised controlled trial **Study grouping:** parallel group **Duration of study:** 12 weeks of treatment and follow‐up to 6 months **Ethics approvals:** not given **Location of study:** South Carolina, USA **Randomisation method:** not given
Participants	**Baseline characteristics** CBT (M‐CET) *Participants (prerandomisation assessment)*: N/A *Gender*: not given *Age*: not given *Ethnicity*: not given *Disability*: not given *Time since trauma*: not given *Income*: not given *Education*: not given *Employment*: not given *Sexual violence in adulthood*: not given *Comorbid conditions*: not given *Baseline PTSD*: not given *Partnered*: not given *Randomised (N)*: 29 *Completed post‐treatment assessment (N)*: 22 *Dropped out or removed prior to analysis (N)*: 7 *Numbers analysed at final applicable time point (N)*: 22 *Number of sessions*: not given *Treatment completion*: 16 (55%) Wait‐list *Participants (prerandomisation assessment)*: N/A *Gender*: not given *Age*: not given *Ethnicity*: not given *Disability*: not given *Time since trauma*: not given *Income*: not given *Education*: not given *Employment*: not given *Sexual violence in adulthood*: not given *Comorbid conditions*: not given *Baseline PTSD*: not given *Partnered*: not given *Randomised (N)*: 31 *Completed post‐treatment assessment (N)*: 23 *Dropped out or removed prior to analysis (N)*: 0 *Numbers analysed at final applicable time point (N)*: 31 *Number of sessions*: N/A *Treatment completion*: N/A Overall *Participants (prerandomisation assessment)*: 62 *Gender*: 100% female participants *Age, M (SD)*: 35 years (9.82 years) *Ethnicity*: 69% white, 24% Black, 7% other *Disability*: not given *Time since trauma*: not given *Income*: 32% < USD 5000, 18% USD 15,000 to USD 25,000 *Education (% high school, college or apprentice, university or years of education)*: 87% high school *Employment (% employed; unemployed, other)*: 36% employed *Sexual violence in adulthood*: 76% sexual violence in adulthood; 69% unwanted sexual contact before 18 years *Comorbid conditions*: 89% anxiety disorders (ADIS‐R); moderate levels of depression (BDI) *Baseline PTSD*: not given *Partnered*: 34% *Approached*: 62 *Ineligible (N)*: 2 *Declined (N)*: 0 *Other (N)*: 0 *Randomised (N)*: 60 *Completed post‐treatment assessment (N)*: 43 *Dropped out or removed prior to analysis (N)*: 7 *Numbers analysed at final applicable time point (N)*: 53 *Number of sessions*: N/A *Treatment completion*: N/A **Inclusion criteria** Experienced a traumatic event at least 3 months before assessment PTSD Experiencing panic attacks **Exclusion criteria** Active psychosis Intellectual disability Current suicidal or parasuicidal behaviour Current drug or alcohol dependency Illiteracy **Pretreatment:** none reported
Interventions	**Intervention characteristics** CBT (M‐CET) *Planned number of intervention sessions*: 12 *Mode of delivery (face‐to‐face, online, video, telephone, blend, with explanation)*: face‐to‐face *Format (group, individual, blend)*: group *Therapist qualifications and training appropriate (yes, no, unclear, with explanation)*: yes, participants were treated in groups of 5 to 7 by therapists trained in M‐CET. Three therapists conducted the groups, and every group had 2 therapists assigned. The third therapist was Joanne L Davis, a postdoctoral fellow at the time the study was conducted. *Research allegiance or conflict of interest (yes, no, unclear, with explanation)*: yes, Falsetti and Resnick developed the treatment. *Treatment fidelity (yes, no, unclear, with explanation)*: yes, all therapy sessions were audiotaped. A random sampling of 20 tapes of 72 were evaluated for treatment adherence and competence by an independent evaluator trained in the treatment. There were no violations of adherence to the treatment protocol, and all tapes were rated to be satisfactory or better on therapist skills. *Intervention aim and theoretical basis*: with M‐CET, exposure to the physiological channel is conducted through interoceptive exposure to physiological reactions. Exposure to panic symptoms decreases fear of physiological symptoms and also provides exposure to the physiological component of fear associated with trauma, thereby weakening the association of physiological arousal and the traumatic memory. The interoceptive exposure is hypothesised to decrease fear of physiological arousal symptoms experienced in panic and PTSD; thus, when exposed to traumatic memories and cues, clients may be less fearful of physiological reactions. Exposure to the cognitive channel is conducted through writing assignments about the meaning of the trauma, writings about the traumatic event itself, and challenging cognitive distortions about how the trauma has affected safety, trust, esteem, intimacy, and power and control beliefs. These components have been adapted from CPT. Finally, exposure to the behavioural channel is conducted through in vivo exposure to conditioned cues to the traumatic event. *Duration of intervention*: 12 weeks Wait‐list *Planned number of intervention sessions*: N/A *Mode of delivery*: N/A *Format*: N/A *Therapist qualifications and training appropriate (yes, no, unclear, with explanation)*: N/A *Research allegiance or conflict of interest (yes, no, unclear, with explanation)*: N/A *Treatment fidelity (yes, no, unclear, with explanation)*: N/A *Intervention aim and theoretical basis*: participants in the control condition were called every 2 weeks and given supportive phone counselling by 1 of the therapists. These women were told that if their condition worsened, they would receive immediate treatment. *Duration of intervention*: N/A
Outcomes	**Depression** Outcome type: continuous outcome Scale used: BDI Direction: lower is better Score range: 0 to 63 Data value: endpoint **PTSD** Outcome type: continuous outcome Scale used: MPSS‐SR Direction: lower is better Score range: 0 to 119 Data value: endpoint **Treatment dropout (intervention group only)** Outcome type: dichotomous outcome
Identification	**Sponsorship source:** NIMH grant MH‐53381‐01A1 **Country:** USA **Setting:** women seeking treatment for PTSD and panic attacks at the National Crime Victims Research and Treatment Center and a community sample of women seeking help for PTSD and panic attacks **Authors name:** Sherry A Falsetti **Institution:** University of Illinois College of Medicine, Rockford **Email:** falsetti@uic.edu **Year:** 2008
Notes	62 is the number approached and assessed for inclusion but reported on in population characteristics; 60 women were randomised. Included in meta‐analysis

**Table:** Feske 2008

***Study characteristics***	
Methods	**Study design:** randomised controlled trial **Study grouping:** parallel group **Duration of study:** recruitment timeframe not stated; up to 12 weeks of treatment and 3 months of follow‐up post‐treatment **Location of study:** Pittsburgh, Pennsylvania, USA **Randomisation method:** participants were randomly assigned to PE or TAU. Therapists were crossed with treatment condition, and participants were randomly assigned to therapists within scheduling constraints. **Ethics approval:** author reports that participants provided signed informed consent in compliance with the University of Pittsburgh’s IRB procedures.
Participants	**Baseline characteristics** CBT (PE) *Participants (treatment completers only)*: 9 *Gender*: 100% female participants *Age*: not given *Ethnicity*: not given *Disability*: not given *Time since trauma*: not given *Income*: not given *Education*: not given *Employment*: not given *Sexual violence in adulthood*: not given *Comorbid conditions*: not given *Baseline PTSD, M (SD)*: 34.89 (7.64) *Partnered*: not given *Randomised (N)*: 13 *Completed post‐treatment assessment (N)*: 9 *Dropped out or removed prior to analysis (N)*: 4 *Numbers analysed at final applicable time point (N)*: 9 *Number of sessions, M (SD)*: 9.3 (1.0) *Treatment completion*: 9 (69.2%) Usual care *Participants (treatment completers only)*: 12 *Gender*: 100% female participants *Age*: not given *Ethnicity*: not given *Disability*: not given *Time since trauma*: not given *Income*: not given *Education*: not given *Employment*: not given *Sexual violence in adulthood*: not given *Comorbid conditions*: not given *Baseline PTSD, M (SD)*: 35.17 (8.31) *Partnered*: not given *Randomised (N)*: 14 *Completed post‐treatment assessment (N)*: 12 *Dropped out or removed prior to analysis (N)*: 2 *Numbers analysed at final applicable time point (N)*: 12 *Number of sessions, M (SD)*: 9.5 (1.2) *Treatment completion*: 12 (85.7%) Overall *Participants (treatment completers only)*: 21 *Gender*: 100% female participants *Age, M (range)*: 43.1 years (29 years to 55 years) *Ethnicity*: 4.8% white, 95.2% Black *Disability*: 33% received disability payments *Time since trauma*: not given *Income*: 42.9% < USD 4999, 23.8% USD 5000 to USD 9999, 3.3% > USD 10,000 *Education (% high school, college or apprentice, university or years of education)*: 38.1% high school, 33.2% some high school, 28.7% some college or vocational training *Employment (% employed, unemployed, other)*: 57.1% unemployed, 33.3% other, 9.6% employed *Sexual violence in adulthood*: 47.6% adulthood; 85.7% multiple traumas including child sexual abuse *Comorbid conditions*: 95.2% had comorbid conditions – major depression (66.7%), panic disorder (38.1%), social phobia (28.6%) and generalised anxiety disorder (23.8%); 52.4% met DSM‐IV criteria for borderline personality disorder *Baseline PTSD*: not given *Partnered*: 4.8% married *Approached (N)*: 168 *Ineligible (N)*: 131 *Declined (N)*: 10 *Other (N)*: 0 *Randomised (N)*: 27 *Completed post‐treatment assessment (N)*: 21 *Dropped out or removed prior to analysis (N)*: 6 *Numbers analysed at final applicable time point (N)*: 21 *Number of sessions*: N/A *Treatment completion*: N/A **Inclusion criteria** Female Psychiatric outpatients attending the participating outreach clinic **Exclusion criteria** Current depression with psychotic symptoms or suicidal ideation Current alcohol or substance dependence A lifetime diagnosis of a psychotic or organic mental disorder Current, clinically significant self‐injurious behaviours Unstable life circumstances that would interfere with treatment (e.g. immediate threat of loss of housing, impending prison sentence) Women currently at risk of abuse by partner/assailant **Pretreatment:** none reported
Interventions	**Intervention characteristics** CBT (PE) *Planned number of intervention sessions*: 9 to 12 weekly 90‐minute individual sessions *Mode of delivery (face‐to‐face, online, video, telephone, blend, with explanation)*: face‐to‐face *Format (group, individual, blend)*: individual *Therapist qualifications and training appropriate (yes, no, unclear, with explanation)*: yes, community therapists were 5 masters‐level social workers and 1 registered nurse. They did not specialise in the treatment of PTSD and had no prior training in behaviour therapy for anxiety disorders; however, they did receive 52 h of training over 6 months. The therapists were trained by the author who was trained in PE by EB Foa. *Research allegiance or conflict of interest (yes, no, unclear, with explanation)*: no *Treatment fidelity (yes, no, unclear, with explanation)*: yes, therapists were given an adherence checklist for each protocol session. PE checklists summarised the core interventions and order in which they should be applied for each session. Therapists participated in weekly supervisory sessions with the author to review PE treatments. All sessions were videotaped. The author reviewed every second PE session using an integrity checklist and discussed any deviations from the protocol. None of the PE sessions she reviewed warranted exclusion due to treatment integrity violations. Formal treatment integrity checks were not conducted, on grounds that this was preliminary research. *Intervention aim and theoretical basis*: PE was based on the manual created by Foa 1998 and aims to reduce PTSD by clients reliving the traumatic event by imagining it as vividly as possible and describing it aloud in the present tense several times for a total of 30 min to 60 min per session (imaginal exposure). After the exposure, therapists discussed clients’ experiences during the reliving and assigned imaginal and in vivo homework. The first 2 sessions comprised information gathering, education about PTSD, the practice of diaphragmatic breathing, explanation of the treatment rationale, and construction of the in vivo exposure hierarchy. During the remaining sessions, clients relived the traumatic event by imagining it as vividly as possible and describing it aloud in the present tense several times for a total of 30 min to 60 min per session (imaginal exposure). After the exposure, therapists discussed clients’ experiences during the reliving and assigned imaginal and in vivo homework. Clients were encouraged to listen to their audiotaped imaginal exposure narratives at least once daily. In vivo exposure was conducted along a hierarchy of feared situations judged by the therapist and client to be safe. In Session 8, clients who reported an improvement in PTSD symptoms of < 70% in relation to their pretreatment PDS‐I score were encouraged to participate in 3 additional PE sessions *Duration of intervention*: 9 weeks to 12 weeks Usual care *Planned number of intervention sessions*: 9 to 12 weekly 60‐minute individual sessions *Mode of delivery (face‐to‐face, online, video, telephone, blend, with explanation)*: face‐to‐face *Format (group, individual, blend)*: blend *Therapist qualifications and training appropriate (yes, no, unclear, with explanation)*: yes, existing TAU therapists received additional training *Research allegiance or conflict of interest (yes, no, unclear, with explanation)*: no *Treatment fidelity (yes, no, unclear, with explanation)*: yes, TAU checklists summarised permitted and proscribed interventions. Therapists participated in weekly supervisory sessions with the author to review PE and TAU treatments. All sessions were videotaped. The author reviewed every second usual treatment session using an integrity checklist and discussed any deviations from the protocol. None reviewed included PE interventions. *Intervention aim and theoretical basis*: TAU consisted of the standard treatment provided at the clinic and included 1 weekly 60‐minute individual counselling session (i.e. 1 weekly TAU protocol session). In addition, TAU clients received 1 weekly 90‐minute group treatment session (e.g. anger management). TAU therapists were trained in a treatment rationale that emphasised the following: 1) It is not known whether PE is more effective for PTSD than TAU is for low‐income women; 2) addressing current life problems other than PTSD is likely to reduce clients’ general stress level, thereby reducing PTSD symptoms; and 3) targeting symptoms related to depression and interpersonal difficulties may have a similarly positive effect on PTSD symptoms. TAU clients who reported an improvement in PTSD symptoms of < 70% in Session 8 were encouraged to participate in 3 additional TAU sessions. TAU therapists were allowed to discuss clients’ traumatic experiences and PTSD symptoms but were to refrain from using formal PE procedures (i.e. imaginal or in vivo exposure as specified in the PE manual). *Duration of intervention*: 9 weeks to 12 weeks
Outcomes	**Depression** Outcome type: continuous outcome Scale used: BDI‐III Direction: lower is better Score range: 0 to 63 Data value: endpoint, 3 months **PTSD** Outcome type: continuous outcome Scale used: PDS‐I Direction: lower is better Score range: 0 to 51 Data value: endpoint, 3 months **Treatment dropout (both groups)** Outcome type: dichotomous outcome **Anxiety** Outcome type: continuous outcome Scale used: BAI Direction: lower is better Score range: 0 to 63 Data value: endpoint, 3 months **Global mental health** Outcome type: continuous outcome Scale used: BSI Direction: lower is better Score range: 0 to 112 Data value: endpoint, 3 months **Adverse events** Outcome type: adverse event Notes: 1 person in TAU dropped out due to worsening depression after the third TAU session.
Identification	**Sponsorship source:** NIMH grant MH01675 **Country:** USA **Setting:** African American women who were psychiatric outpatients at a university‐affiliated outreach clinic, recruited through fliers and referrals by mental health professionals; intervention was delivered by nonspecialist community therapists. **Authors name:** Ulrike Feske **Institution:** University of Pittsburgh **Email:** ulf1@pitt.edu **Year:** 2008
Notes	Population characteristics described for full sample of 21 PE and usual care completers only. Included in meta‐analysis

**Table:** Foa 1991

***Study characteristics***	
Methods	**Study design:** randomised controlled trial **Study grouping:** parallel group **Duration of study:** recruitment timeframe not specified; 4.5 week treatment, 3‐month follow‐up post‐treatment **Ethics approvals:** not given **Location of study:** Pennsylvania, USA **Randomisation method:** eligible individuals were randomly assigned to 1 of the 4 conditions. After 10 participants were entered into the wait‐list condition, subsequent admissions were randomly assigned to 1 of the 3 treatment groups.
Participants	**Baseline characteristics** CBT (SIT) *Participants (completers)*: 14 *Gender*: 100% female participants *Age, M (SD)*: 29.3 years (6.3 years) *Ethnicity*: Black 15.4%, white 84.6% *Disability*: not given *Time since trauma, M (SD)*: 120.5 months (131 months) *Income*: 7.7% > USD 30,000, 15.4% USD 20,000 to USD 30,000, 38.5% USD 10,000 to USD 20,000, 38.5% < USD 10,000 *Education:* not given *Employment (% employed; unemployed, other)*: 74.9% employed, 25% other *Sexual violence in adulthood*: not given *Comorbid conditions*: not given *Baseline PTSD, M (SD)*: 24.48 (6.62) *Partnered*: 30.8% *Randomised (N)*: 17 *Included cases at post‐treatment assessment (N)*: 14 *Dropped out or removed prior to analysis (N)*: 8 *Numbers analysed at final applicable time point (N)*: 9 *Number of sessions*: not given *Treatment completion*: 14 (82.3%) CBT (PE) *Participants (completers)*: 10 *Gender*: 100% female participants *Age, M (SD)*: 32.7 years (7.3 years) *Ethnicity*: Black 30%, white 70% *Disability*: not given *Time since trauma, M (SD)*: 165 months (248 months) *Income*: 40% > USD 30,000, 10% USD 20,000 to USD 30,000, 10% USD 10,000 to USD 20,000, 40% < USD 10,000 *Education*: not given *Employment (% employed; unemployed, other)*: 55.5% employed; 44% other *Sexual violence in adulthood*: not given *Comorbid conditions*: not given *Baseline PTSD, M (SD)*: 25.78 (5.01) *Partnered*: 30% *Randomised (N)*: 14 *Included cases at post‐treatment assessment (N)*: 10 *Dropped out or removed prior to analysis (N)*: 5 *Numbers analysed at final applicable time point (N)*: 9 *Number of sessions*: not given *Treatment completion*: 10 (71.4%) Humanistic (SC) *Participants (completers)*: 11 *Gender*: 100% female participants *Age, M (SD)*: 34.2 years (9.8 years) *Ethnicity*: Black 27.3%, white 63.6%, other 9.1% *Disability*: not given *Time since trauma, M (SD)*: 282 months (263 months) *Income*: 36.4% > USD 30,000, 9.1% USD 20,000 to USD 30,000, 36.4% USD 10,000 to USD 20,000, 18.2% < USD 10,000 *Education*: not given *Employment (% employed; unemployed, other)*: 80% employed; 20% other *Sexual violence in adulthood*: not given *Comorbid conditions*: not given *Baseline PTSD, M (SD)*: 24.39 (6.62) *Partnered*: 18.2% *Randomised (N)*: 14 *Included cases at post‐treatment assessment (N)*: 11 *Dropped out or removed prior to analysis (N)*: 5 *Numbers analysed at final applicable time point (N)*: 9 *Number of sessions*: not given *Treatment completion*: 11 (79%) Wait‐list *Participants (completers)*: 10 *Gender*: 100% female participants *Age, M (SD)*: 32.0 years (9.6 years) *Ethnicity*: Black 30%, white 70% *Disability*: not given *Time since trauma, M (SD)*: 205 months (150 months) *Income*: 20% > USD 30,000, 10% USD 20,000 to USD 30,000, 50% USD 10,000 to USD 20,000, 20% < USD 10,000 *Education*: not given *Employment (% employed; unemployed, other)*: 50% employed, 50% other *Sexual violence in adulthood*: not given *Comorbid conditions*: not given *Baseline PTSD, M (SD)*: 24.43 (4.64) *Partnered*: 20% *Randomised (N)*: 10 *Included cases at post‐treatment assessment (N)*: 10 *Dropped out or removed prior to analysis (N)*: 10 *Numbers analysed at final applicable time point (N)*: 0 *Number of sessions*: N/A *Treatment completion*: N/A Overall *Participants (completers)*: 45 *Gender*: 100% female participants *Age, M (SD)*: 31.8 years (8.2 years) *Ethnicity*: Black 25.0%, white 72.7%, other 2.3% *Disability*: not given *Time since trauma, M (SD)*: 6.2 years (6.7 years); range from 3 months to 12 years *Income*: 25% > USD 30,000, 11.4% USD 20,000 to USD 30,000, 34.1% USD 10,000 to USD 20,000, 29.5% < USD 10,000 *Education*: not given *Employment (% employed, unemployed, other)*: 65.9% employed; 12.2% other *Sexual violence in adulthood*: not given *Comorbid conditions*: not given *Baseline PTSD*: not given *Partnered*: 25% *Approached (N)*: not given *Ineligible (N)*: not given *Declined (N)*: 11 *Other (N)*: 0 *Randomised (N)*: 55 *Included cases at post‐treatment assessment (N)*: 45 *Dropped out or removed prior to analysis (N)*: 28 *Numbers analysed at final applicable time point (N)*: 27 *Number of sessions*: N/A *Treatment completion*: N/A **Inclusion criteria** Victims of rape or attempted rape who met DSM‐III‐R diagnostic criteria for PTSD Raped minimum 3 months before participation in the study **Exclusion criteria** Current or previous diagnosis of organic mental disorder, schizophrenia or paranoid disorders as defined in the DSM‐III‐R Depression severe enough to require immediate psychiatric treatment, bipolar depression or depression accompanied by delusions, hallucinations or bizarre behaviour Current alcohol or drug abuse Assault by spouse or other family member Illiteracy in English **Pretreatment:** significantly fewer participants in SIT group reported injury during the assault.
Interventions	**Intervention characteristics** CBT (SIT) *Planned number of intervention sessions*: 9 (90‐minute sessions twice a week) *Mode of delivery (face‐to‐face, online, video, telephone, blend, with explanation)*: face‐to‐face *Format (group, individual, blend)*: individual *Therapist qualifications and training appropriate (yes, no, unclear, with explanation)*: yes, therapists had master's or doctoral degrees in psychology or clinical social work. Therapists were trained in treatments by EB Foa and BO Rothbaum. *Research allegiance or conflict of interest (yes, no, unclear, with explanation)*: no *Treatment fidelity (yes, no, unclear, with explanation)*: yes, therapists were supervised biweekly by Foa. Each therapy session was monitored during supervision to examine possible deviations from protocol. No gross deviations were detected; subtle deviations were noted, and suggestions for correction were provided by the supervisor. Nonparametric (Kruskal‐Wallis) tests were conducted to examine possible therapist effects. The therapists did not differ in the percentage of improvement demonstrated by their patients on any of the 7 outcome measures used in the current study. *Intervention aim and theoretical basis*: the procedures included in this treatment programme were adapted from (Veronen 1983). The first session was devoted to information gathering through the initial interview described above. The session terminated with breathing exercises to diminish anxiety that may have been elicited by the interview. During the second session, the treatment method was described to the participant, a rationale for treatment was given, and an explanation for the origin of fear and anxiety was presented. The next 7 sessions were devoted to instruction in coping skills. During the third and fourth sessions, the participants were taught deep muscle relaxation and controlled breathing. In the fifth session, they were taught thought‐stopping to counter ruminative or obsessive thinking (Wolpe 1958). The sixth session was devoted to CR (Beck 1979; Ellis 1977), the seventh to guided self‐dialogue (Meichenbaum 1977), the eighth to covert modelling, and the ninth to role‐playing. No instructions for exposure were included. *Duration of intervention*: 4.5 weeks CBT (PE) *Planned number of intervention sessions*: 9 (90‐minute sessions twice a week) *Mode of delivery (face‐to‐face, online, video, telephone, blend, with explanation)*: face‐to‐face *Format (group, individual, blend)*: individual *Therapist qualifications and training appropriate (yes, no, unclear, with explanation)*: as previous *Research allegiance or conflict of interest (yes, no, unclear, with explanation)*: no *Treatment fidelity (yes, no, unclear, with explanation)*: as previous *Intervention aim and theoretical basis*: the first 2 sessions were devoted to information gathering through the initial interview described above, explanation of treatment rationale, and treatment planning. The next 7 sessions were devoted to reliving the rape scene in imagination (imaginal exposure). Participants were instructed to relive the assault by imagining it as vividly as possible and describing it aloud using the present tense. The participant repeated the rape scenario several times for a total of 60 min per session. The participants' narratives were tape‐recorded, and participants were instructed to listen to the tape at least once daily as homework. Additional homework involved in vivo exposure to feared and avoided situations judged by the participant and the therapist to be safe. *Duration of intervention*: 4.5 weeks Humanistic (SC) *Planned number of intervention sessions*: 9 (90‐minute sessions twice a week) *Mode of delivery (face‐to‐face, online, video, telephone, blend, with explanation)*: face‐to‐face *Format (group, individual, blend)*: individual *Therapist qualifications and training appropriate (yes, no, unclear, with explanation)*: as previous *Research allegiance or conflict of interest (yes, no, unclear, with explanation)*: no *Treatment fidelity (yes, no, unclear, with explanation)*: as previous *Intervention aim and theoretical basis*: involved gathering information through the initial interview in the first session and presenting the rationale for treatment in the second session. During the remaining sessions, participants were taught a general problem‐solving technique. Therapists played an indirect and unconditionally supportive role. Homework consisted of the participants keeping a diary of daily problems and attempts at problem‐solving. Participants were immediately redirected to focus on current daily problems if discussions of the assault occurred. No instructions for exposure or anxiety management were included. *Duration of intervention*: 4.5 weeks Wait‐list *Planned number of intervention sessions*: N/A *Mode of delivery*: N/A *Format*: N/A *Therapist qualifications and training appropriate*: N/A *Research allegiance or conflict of interest*: N/A *Treatment fidelity*: N/A *Intervention aim and theoretical basis*: N/A *Duration of intervention*: N/A
Outcomes	**PTSD** Outcome type: continuous outcome Scale used: PTSD interview Direction: lower is better Score range: 17 to 119 Data value: endpoint, 3 months, 6 months and 12 months **Depression** Outcome type: continuous outcome Scale used: BDI Direction: lower is better Score range: 0 to 63 Data value: endpoint, 3 months, 6 months and 12 months **Trauma‐related beliefs** Outcome type: continuous outcome Scale used: RAST Direction: lower is better Score range: 0 to 280 Data value: endpoint, 3 months, 6 months and 12 months **Anxiety** Outcome type: continuous outcome Scale used: STAI‐State Direction: lower is better Score range: 0 to 80 Data value: endpoint, 3 months, 6 months and 12 months **Treatment dropout** Outcome type: dichotomous outcome **PTSD** Outcome type: continuous outcome Scale used: PTSD interview Intrusion Direction: lower is better Score range: 4 to 28 Data value: endpoint, 3 months, 6 months and 12 months **PTSD** Outcome type: continuous outcome Scale used: PTSD interview Arousal Direction: lower is better Score range: 7 to 49 Data value: endpoint, 3 months, 6 months and 12 months **PTSD** Outcome type: continuous outcome Scale used: PTSD interview Avoidance Direction: lower is better Score range: 6 to 42 Data value: endpoint, 3 months, 6 months and 12 months
Identification	**Sponsorship source:** NIMH grant MH42178 **Country:** USA **Setting:** survivors of rape recruited through local providers and victim assistance agencies, media and other research; intervention delivered in academic setting **Authors name:** Edna Foa **Institution:** Department of Psychiatry, Medical College of Pennsylvania **Email:** foa@auhs.edu **Year:** 1991
Notes	Numbers reported for population characteristics refer to those who completed the therapies. Included in meta‐analysis Concerns about process of assigning people to wait‐list led to exclusion of this arm in meta‐analyses.

**Table:** Foa 1999

***Study characteristics***	
Methods	**Study design:** randomised controlled trial **Study grouping:** parallel group **Duration of study:** recruitment timescale not specified; 9‐week intervention, 12‐month follow‐up: 1 year and 2 months **Location of study:** Philadelphia, Pennsylvania, USA **Randomisation method:** participants were randomly assigned to 1 of the following 4 conditions: PE, SIT, combined treatment (PE‐SIT), or wait‐list. Having enrolled 10 participants into wait‐list control, more participants were assigned to the 3 active groups than to wait‐list. **Ethics approvals:** not given
Participants	**Baseline characteristics** CBT (PE) *Participants (select at randomisation if available)*: 25 *Gender*: 100% female participants *Age*: not given *Ethnicity*: not given *Disability*: not given *Time since trauma*: not given *Income*: not given *Education*: not given *Employment*: not given *Sexual violence in adulthood*: not given *Comorbid conditions*: not given *Baseline PTSD, M (SD)*: 29.28 (9.94) *Partnered*: not given *Randomised (N)*: 25 *Completed post‐treatment assessment (N)*: 23 *Dropped out or removed prior to analysis (N)*: 9 *Numbers analysed at final applicable time point (N)*: 16 *Number of sessions*: not given *Treatment completion*: 23 (92%) CBT (PE + SIT) *Participants (select at randomisation if available)*: 30 *Gender*: 100% female participants *Age*: not given *Ethnicity*: not given *Disability*: not given *Time since trauma*: not given *Income*: not given *Education*: not given *Employment*: not given *Sexual violence in adulthood*: not given *Comorbid conditions*: not given *Baseline PTSD, M (SD)*: 29.95 (6.97) *Partnered*: not given *Randomised (N)*: 30 *Completed post‐treatment assessment (N)*: 22 *Dropped out or removed prior to analysis (N)*: 14 *Numbers analysed at final applicable time point (N)*: 16 *Number of sessions*: not given *Treatment completion*: 22 (73%) CBT (SIT) *Participants (select at randomisation if available)*: 26 *Gender*: 100% female participants *Age*: not given *Ethnicity*: not given *Disability*: not given *Time since trauma*: not given *Income*: not given *Education*: not given *Employment*: not given *Sexual violence in adulthood*: not given *Comorbid conditions*: not given *Baseline PTSD, M (SD)*: 29.42 (9.69) *Partnered*: not given *Randomised (N)*: 26 *Completed post‐treatment assessment (N)*: 19 *Dropped out or removed prior to analysis (N)*: 12 *Numbers analysed at final applicable time point (N)*: 14 *Number of sessions*: not given *Treatment completion*: 19 (73%) Wait‐list *Participants (select at randomisation if available)*: 15 *Gender*: 100% female participants *Age*: not given *Ethnicity*: not given *Disability*: not given *Time since trauma*: not given *Income*: not given *Education*: not given *Employment*: not given *Sexual violence in adulthood*: not given *Comorbid conditions*: not given *Baseline PTSD, M (SD)*: 32.93 (5.89) *Partnered*: not given *Randomised (N)*: 15 *Completed post‐treatment assessment (N)*: 15 *Dropped out or removed prior to analysis (N)*: 0 *Numbers analysed at final applicable time point (N)*: 0 *Number of sessions*: N/A *Treatment completion*: N/A Overall *Participants (select at randomisation if available)*: 96 *Gender*: 100% female participants *Age, M (SD)*: 34.9 years (10.6 years) *Ethnicity*: 63% white, 36% Black *Disability*: not given *Time since trauma*: not given *Income*: household income < USD 10,000 for a third of the participants; 38% > USD 30,000 *Education (% high school, college or apprentice, university or years of education)*: 10% some high school, 18% high school diplomas, 41% some college education, 31% bachelor's degrees or higher *Employment (% employed; unemployed, other)*: most employed full‐time (46%) or part‐time (16%) *Sexual violence in adulthood*: 72% (69) victims of sexual violence as index trauma; 48% reported at least 1 physical or sexual assault in adulthood prior to the index trauma, and 48% reported at least 1 incident of childhood physical or sexual abuse *Comorbid conditions*: not given *Baseline PTSD*: not given *Partnered*: not given *Approached (N)*: 117 *Ineligible (N)*: 13 *Declined (N)*: 0 *Other (N)*: 8 *Randomised (N)*: 96 *Completed post‐treatment assessment (N)*: 79 *Dropped out or removed prior to analysis (N)*: 35 *Numbers analysed at final applicable time point (N)*: 46 *Number of sessions*: N/A *Treatment completion*: N/A **Inclusion criteria** Participants met the criteria for PTSD based on DSM‐III‐R as their primary diagnosis **Exclusion criteria** Current schizophrenia, bipolar disorder, organic mental disorder, alcohol or drug dependence, severe suicidal ideation Being in intimate relationship with assailant **Pretreatment:** No significant differences in demographics or pretreatment measures of psychopathology. There was an observed trend towards group differences on employment status – 19% of PE participants were unemployed compared with 30% of SIT, 43% of PE‐SIT, and 8% of wait‐list participants. No pre‐ or post‐treatment differences were detected between victims of sexual (n = 69) and nonsexual assault (n = 27).
Interventions	**Intervention characteristics** CBT (PE) *Planned number of intervention sessions*: 9 sessions totalling 14.5 h (2 sessions of 120 min + 7 sessions of 90 min) *Mode of delivery (face‐to‐face, online, video, telephone, blend, with explanation)*: face‐to‐face *Format (group, individual, blend)*: individual *Therapist qualifications and training appropriate (yes, no, unclear, with explanation)*: yes, PhD‐level clinical psychologists were trained to use manuals that specified precise treatment guidelines for each session and received ongoing supervision by EB Foa and CV Dancu. *Research allegiance or conflict of interest (yes, no, unclear, with explanation)*: no *Treatment fidelity (yes, no, unclear, with explanation)*: yes, in addition to precise treatment guidelines and regular supervision, 9% of sessions were videotaped and assessed for presence of 52 intervention components across the treatments. On average, therapists completed 93% (SD = 12%) of the components prescribed for a given session in the corresponding protocol *Intervention aim and theoretical basis*: to encourage clients to relive memories of the traumatic event (i.e. imaginal exposure) and confront situations that are avoided because they trigger distressing memories and thoughts. Based on description in Foa 1998, PE focused on reliving the traumatic event in imagination (i.e. imaginal exposure). Homework assignments consisted of in vivo exposure to objectively safe situations that caused anxiety or were avoided. *Duration of intervention*: 5 weeks CBT (PE + SIT) *Planned number of intervention sessions*: 9 sessions totalling 14.5 h (2 sessions of 120 min + 7 sessions of 90 min) *Mode of delivery (face‐to‐face, online, video, telephone, blend, with explanation)*: face‐to‐face *Format (group, individual, blend)*: individual *Therapist qualifications and training appropriate (yes, no, unclear, with explanation)*: as previous *Research allegiance or conflict of interest (yes, no, unclear, with explanation)*: no *Treatment fidelity (yes, no, unclear, with explanation)*: yes, as previous *Intervention aim and theoretical basis*: combination of PE and SIT elements as described, with the aim of demonstrating a superior outcome to either treatments alone *Duration of intervention*: 5 weeks CBT (SIT) *Planned number of intervention sessions*: 9 sessions totalling 14.5 h (2 sessions of 120 min + 7 sessions of 90 min) *Mode of delivery (face‐to‐face, online, video, telephone, blend, with explanation)*: face‐to‐face *Format (group, individual, blend)*: individual *Therapist qualifications and training appropriate (yes, no, unclear, with explanation)*: as previous *Research allegiance or conflict of interest (yes, no, unclear, with explanation)*: no *Treatment fidelity (yes, no, unclear, with explanation)*: yes, as previous; however, 1 insignificant deviation was observed *Intervention aim and theoretical basis*: to teach clients various coping strategies to manage trauma‐related anxiety (i.e. relaxation training, thought‐stopping, CR and positive self‐statements), adapted from Veronen 1983. SIT focused on anxiety management skills (i.e. breathing retraining, thought‐stopping, CR, positive affirmations and problem‐solving). SIT homework assignments consisted of assigned practice of the instructed coping skill. *Duration of intervention*: 5 weeks Wait‐list *Planned number of intervention sessions*: N/A *Mode of delivery*: N/A *Format*: N/A *Therapist qualifications and training appropriate*: N/A *Research allegiance or conflict of interest*: N/A *Treatment fidelity*: N/A *Intervention aim and theoretical basis*: N/A *Duration of intervention*: wait‐list ceased after 5 weeks
Outcomes	**PTSD** Outcome type: continuous outcome Scale used: PSS‐I Direction: lower is better Score range: 0 to 51 Data value: endpoint, 3 months, 6 months and 12 months **Depression** Outcome type: continuous outcome Scale used: BDI Direction: lower is better Score range: 0to 63 Data value: endpoint, 3 months, 6 months and 12 months **Anxiety** Outcome type: continuous outcome Scale used: STAI Direction: lower is better Score range: 20 to 80 Data value: endpoint, 3 months, 6 months and 12 months **Treatment dropout** Outcome type: dichotomous outcome
Identification	**Sponsorship source:** NIMH grant MH4217 **Country:** USA **Setting:** academic/clinical **Comments:** not clear how potential participants were approached and recruited into the study or setting of interventions. Extracted data included those for whom assessments were available rather than ITT. **Authors name:** Edna Foa **Institution:** Pennsylvania‐Hahnemann University **Email:** foa@mail.med.upenn.edu **Year:** 1999
Notes	Included in meta‐analysis

**Table:** Foa 2005

***Study characteristics***	
Methods	**Study design:** randomised controlled trial **Study grouping:** parallel group **Duration of study:** recruitment took place January 1995 to September 2000 **Location of study:** Philadelphia, Pennsylvania, USA **Randomisation method:** the study statistician assigned participants who provided informed consent to 1 of the 3 conditions using a weighted randomisation procedure such that participants were assigned to 1 of the active treatment conditions at a greater rate than to wait‐list. Therapists made contact with the participants and arranged initial therapy appointments with those assigned to active treatment, and they also informed them of the specific treatment condition at the first session. Wait‐list participants were informed by phone that they had been assigned to the wait‐list condition. **Ethics approvals:** not given
Participants	**Baseline characteristics** CBT (PE) *Participants (select at randomisation if available)*: 79 *Gender*: 100% female participants *Age*: not given *Ethnicity*: not given *Disability*: not given *Time since trauma*: not given *Setting*: not given *Education*: not given *Employment*: not given *Sexual violence in adulthood*: not given *Comorbid conditions*: not given *Baseline PTSD, M (SD)*: 34.0 (5.9) *Partnered*: not given *Randomised (N)*: 79 *Completed post‐treatment assessment (N)*: 52 *Dropped out or removed prior to analysis (N)*: 27 *Numbers analysed at final applicable time point (N)*: 52 *Number of sessions*: not given *Treatment completion*: 52 (66%) CBT (PE + CR) *Participants (select at randomisation if available)*: 74 *Gender*: 100% female participants *Age*: not given *Ethnicity*: not given *Disability*: not given *Time since trauma*: not given *Setting*: not given *Education*: not given *Employment*: not given *Sexual violence in adulthood*: not given *Comorbid conditions*: not given *Baseline PTSD, M (SD)*: 31.1 (8.1) *Partnered*: not given *Randomised (N)*: 74 *Completed post‐treatment assessment (N)*: 44 *Dropped out or removed prior to analysis (N)*: 30 *Numbers analysed at final applicable time point (N)*: 44 *Number of sessions*: not given *Treatment completion*: 44 (59%) Wait‐list *Participants (select at randomisation if available)*: 26 *Gender*: 100% female participants *Age*: not given *Ethnicity*: not given *Disability*: not given *Time since trauma*: not given *Setting*: not given *Education*: not given *Employment*: not given *Sexual violence in adulthood*: not given *Comorbid conditions*: not given *Baseline PTSD, M (SD)*: 33.3 (6.2) *Partnered*: not given *Randomised (N)*: 26 *Completed post‐treatment assessment (N)*: 25 *Dropped out or removed prior to analysis (N)*: 1 *Numbers analysed at final applicable time point (N)*: 25 *Number of sessions*: N/A *Treatment completion*: N/A Overall *Participants (select at randomisation if available)*: 179 *Gender*: 100% female participants *Age, M (SD)*: 31.3 years (9.8 years) *Ethnicity*: 49.2% white, 43.6% Black, 7.3% other *Disability*: 7.4% *Time since trauma, M (SD)*: 108 months (135.6 months) *Setting*: 47.4% < USD 15,000, 24.3% USD 15,001 to USD 30,000, 16.2% USD 30,001 to USD 50,000, 12.1% > USD 50,001 *Education (% high school, college or apprentice, university or years of education)*: 29.9% some/high school, 43.5% some college, 26.5% BA/BS or greater *Employment (% employed; unemployed, other)*: 50.6% employed, 22.7% unemployed, 26.7% other *Sexual violence in adulthood*: 68.7% *Comorbid conditions (%)*: Axis I condition 67.3%; the most common comorbid conditions were as follows: major depression (41.2%), social anxiety disorder (20.4%), specific phobias (20.4%), generalised anxiety disorder (13.9%) and panic disorder (11.9%). All other disorders were present at rates of 6% or less. *Baseline PTSD*: not given *Partnered*: 21.5% *Approached (N)*: 285 *Ineligible (N)*: 56 *Declined (N)*: 19 *Other (N)*: 31 *Randomised (N)*: 179 *Completed post‐treatment assessment (N)*: 121 *Dropped out or removed prior to analysis (N)*: 58 *Numbers analysed at final applicable time point (N)*: 121 *Number of sessions*: N/A *Treatment completion*: N/A **Inclusion criteria** Adult women A primary diagnosis of PTSD related to a sexual or nonsexual assault that occurred at least 3 months prior to the evaluation **Exclusion criteria** Being in an abusive relationship Current diagnosis of organic mental disorder, schizophrenia or psychotic disorder Unmedicated, symptomatic bipolar disorder; substance dependence Illiteracy in English Women deemed at high risk for suicidal behaviour or with recent history of serious self‐injurious behaviour Women taking psychiatric medication were required to have been taking a stable dose for at least 3 months prior to entry, and they were asked to maintain this regimen during treatment. **Pretreatment:** significant site differences were found on 5 demographic variables – age, index trauma, relationship status, employment and overall comorbidity – although sites did not differ on any specific disorder. There was also a trend for a difference in ethnicity.
Interventions	**Intervention characteristics** CBT (PE) *Planned number of intervention sessions*: 9 to 12 *Mode of delivery (face‐to‐face, online, video, telephone, blend, with explanation)*: face‐to‐face *Format (group, individual, blend)*: individual *Therapist qualifications and training appropriate (yes, no, unclear, with explanation)*: yes, 5 clinicians with doctoral degrees in clinical psychology administered the treatments at the CTSA; 6 clinicians with master’s degrees in counselling or social work administered the treatments at WOAR. In the startup phase of the study, all CTSA and WOAR therapists were trained together in a 5‐day workshop led by Foa and Dancu (PE) and a second 5‐day workshop led by Clark (CR). Therapists were trained to use manuals that described the procedures for each session in great detail. All therapists received ongoing supervision at the therapists’ site throughout the study from Foa, Dancu and Hembree. *Research allegiance or conflict of interest (yes, no, unclear, with explanation)*: unclear, as PE treatment has been developed by the co‐authors, but several areas where attempts were made to minimise bias *Treatment fidelity (yes, no, unclear, with explanation)*: yes, adherence to treatment protocol was monitored during weekly supervision meetings. Using adherence manuals, they randomly selected and rated videotapes of 141 therapy sessions (11.5% of 1227 sessions) for fidelity to the treatment manual. Ten raters trained to conduct the adherence ratings reviewed session videotapes, rated each essential component as present or absent, and monitored for protocol violations. Of these sessions, 29 (21%) were rated independently by 2 raters. Interrater reliability was 0.88. Therapists completed 97% of the components prescribed in the protocol. Seventeen protocol violations were observed in the 141 sessions; 24% of these were observed in the CTSA sessions and 76% in the WOAR sessions. *Intervention aim and theoretical basis*: the first aim of this study was to compare the efficacy of PE alone with a programme that combined PE with CR (PE/CR), a potential technique for ameliorating anxiety disorders. Participants in active treatment who, at the end of 8 sessions, reached at least 70% improvement in self‐reported PTSD symptoms completed treatment after Session 9. The rest were offered up to 12 sessions. They hypothesised the following: 1) there would be greater reduction in PTSD and depression in PE and PE/CR than in wait‐list; 2) PE/CR would be superior to PE alone. *Duration of intervention*: 9 weeks to 12 weeks CBT (PE + CR) *Planned number of intervention sessions*: 9 to 12 *Mode of delivery (face‐to‐face, online, video, telephone, blend, with explanation)*: face‐to‐face *Format (group, individual, blend)*: individual *Therapist qualifications and training appropriate (yes, no, unclear, with explanation)*: as previous *Research allegiance or conflict of interest (yes, no, unclear, with explanation)*: as previous *Treatment fidelity (yes, no, unclear, with explanation)*: as previous *Intervention aim and theoretical basis*: the procedure in the PE/CR treatment was identical to PE alone with 2 exceptions. First, Session 3 was devoted to presenting the idea that post‐trauma symptoms are maintained in part by trauma‐related thoughts and beliefs and to practising CR. Specifically, participants were taught to identify and challenge erroneous and unhelpful beliefs and instructed to record and challenge them for homework using a daily diary. Imaginal exposure was introduced in Session 4, following a review of the preceding week’s diaries. Second, all subsequent sessions included 30 min to 45 min of imaginal exposure followed by 15 min to 25 min of CR. Participants in PE/CR were given the same amount of exposure homework as those in PE, and they also practised CR using their diaries. *Duration of intervention*: 9 weeks to 12 weeks Wait‐list *Planned number of intervention sessions*: N/A *Mode of delivery*: N/A *Format*: N/A *Therapist qualifications and training appropriate*: N/A *Research allegiance or conflict of interest*: N/A *Treatment fidelity*: N/A *Intervention aim and theoretical basis*: participants were assigned a therapist who informed them that they would receive treatment in 9 weeks. Therapists encouraged the wait‐list participants to call at anytime if they were having problems. In addition, the therapists called the wait‐list participants halfway through the waiting period to check in with them and determine their state. *Duration of intervention*: N/A
Outcomes	**PTSD** Outcome type: continuous outcome Scale used: PSS‐I Direction: lower is better Score range: 0 to 51 Data value: endpoint, 3 months, 6 months and 12 months **Depression** Outcome type: continuous outcome Scale used: BDI Direction: lower is better Score range: 0 to 63 Data value: endpoint, 3 months, 6 months and 12 months **Treatment dropout** Outcome type: dichotomous outcome *Adverse events* Outcome type: adverse event Notes: there was no breakdown by group, so we could only include the total for the study. Twelve serious adverse events led to termination in the study, 6 of which are included in the postrandomisation removal category in Figure 1 (4 participants reassaulted, 1 developing a life‐threatening illness and 1 death). The remaining 6 serious adverse events were classified as dropouts (4 had severe depression and suicidal ideation that required immediate intervention, 2 of which were hospitalised, and 2 exhibited extreme dissociative symptoms). Adverse events: 12 serious adverse events led to termination in the study, 6 of which are included in the postrandomisation removal category (4 participants reassaulted, 1 developing a life‐threatening illness and 1 death). The remaining 6 serious adverse events were classified as dropouts (4 had severe depression and suicidal ideation that required immediate intervention, 2 of which were hospitalised, and 2 exhibited extreme dissociative symptoms).
Identification	**Sponsorship source:** this study was supported by NIMH grant MH42178. **Country:** USA **Setting:** CTSA, an academic centre, or WOAR, a community clinic for rape survivors with no experience of CBT delivery **Authors name:** Edna Foa **Institution:** University of Pennsylvania **Email:** foa@mail.med.upenn.edu **Year:** 2005
Notes	Included in meta‐analysis

**Table:** Foa 2006

***Study characteristics***	
Methods	**Study design:** randomised controlled trial **Study grouping:** parallel group **Duration of study:** no recruitment timeframe stated: 4‐week intervention, with the last follow‐up at 12 months post‐intervention **Ethics approvals:** not given **Location of study:** Philadelphia, Pennsylvania, USA **Randomisation method:** participants were randomly assigned to brief CBT or an assessment condition (no‐treatment control); 1 year into the study, the SC condition was added, and randomisation was adjusted accordingly to increase the probability of participants being assigned to the SC condition.
Participants	**Baseline characteristics** CBT (brief CBT) *Participants (at randomisation)*: 31 *Gender*: 100% female participants *Age*: not given *Ethnicity*: not given *Disability*: not given *Time since trauma*: not given *Income*: not given *Education*: not given *Employment*: not given *Sexual violence in adulthood*: 77% *Comorbid conditions*: not given *Baseline PTSD, M (SD)*: 34.03 (7.86) *Partnered*: not given *Randomised (N)*: 31 *Completed post‐treatment assessment (N)*: 22 *Dropped out or removed prior to analysis (N)*: 9 *Numbers analysed at final applicable time point (N)*: 22 *Number of sessions*: not given *Treatment completion*: 22 (71%) Humanistic (SC) *Participants (select at randomisation if available)*: 29 *Gender*: 100% female participants *Age*: not given *Ethnicity*: not given *Disability*: not given *Time since trauma in months*: not given *Income*: not given *Education*: not given *Employment*: not given *Sexual violence in adulthood*: 38% *Comorbid conditions*: not given *Baseline PTSD, M (SD)*: 35.07 (7.63) *Partnered*: not given *Randomised (N)*: 29 *Completed post‐treatment assessment (N)*: 24 *Dropped out or removed prior to analysis (N)*: 5 *Numbers analysed at final applicable time point (N)*: 24 *Number of sessions*: not given *Treatment completion*: 24 (82.8%) Assessment control (AC) *Participants (at randomisation)*: 30 *Gender*: 100% female participants *Age*: not given *Ethnicity*: not given *Disability*: not given *Time since trauma*: not given *Income*: not given *Education*: not given *Employment*: not given *Sexual violence in adulthood*: 73% *Comorbid conditions*: not given *Baseline PTSD, M (SD)*: 33.17 (6.48) *Partnered*: not given *Randomised (N)*: 30 *Completed post‐treatment assessment (N)*: 20 *Dropped out or removed prior to analysis (N)*: 10 *Numbers analysed at final applicable time point (N)*: 20 *Number of sessions*: not given *Treatment completion*: 20 (66.7%) Overall *Participants (randomisation)*: 90 *Gender*: 100% female participants *Age, M (SD)*: 33.7 years (11.13 years) *Ethnicity*: 31.3% white, 62.7% Black, 6% other *Disability*: not given *Time since trauma, M (SD)*: 20.5 d (range of 2 d to 46 d) *Income*: 27.2% > USD 30,000, 42.9% USD 10,000 to USD 30,000, 29.9% < USD 10,000 *Education (% high school, college or apprentice, university or years of education)*: 36.5% high school education or less, 43.9% some technical school or college education, 4.9% college education *Employment*: not given *Sexual violence in adulthood*: 63% *Comorbid conditions*: not given *Baseline PTSD*: not given *Partnered*: 15% *Approached*: not given *Ineligible*: not given *Declined*: not given *Other*: not given *Randomised (N)*: 90 *Completed post‐treatment assessment (N)*: 66 *Dropped out or removed prior to analysis (N)*: 24 *Numbers analysed at final applicable time point (N)*: 66 **Inclusion criteria** Recent survivors of sexual assault or nonsexual assault Met DSM‐IV symptom (not duration) criteria for PTSD **Exclusion criteria** Women who were assaulted by an intimate partner with whom they had an ongoing relationship Primary diagnoses of organic mental disorder, schizophrenia, bipolar disorder Current alcohol or drug dependence **Pretreatment:** 77% of the brief CBT and 73% of the assessment condition reported an index trauma of sexual assault, but only 38% of the SC condition reported a sexual assault as the index trauma.
Interventions	**Intervention characteristics** CBT (brief CBT) *Planned number of intervention sessions*: 4 × 2‐hour sessions *Mode of delivery (face‐to‐face, online, video, telephone, blend, with explanation)*: face‐to‐face *Format (group, individual, blend)*: individual *Therapist qualifications and training appropriate (yes, no, unclear, with explanation)*: yes, masters‐ and doctoral‐level therapists who received ongoing supervision and followed manuals that specified guidelines for each session conducted the interventions. *Research allegiance or conflict of interest (yes, no, unclear, with explanation)*: unclear, the study builds on Foa 1995 and there is clear expectation that brief CBT should be superior; however, there are several measures to reduce any risk of research allegiance. *Treatment fidelity (yes, no, unclear, with explanation)*: yes, tapes of intervention sessions were reviewed in weekly supervision to ensure adherence. *Intervention aim and theoretical basis*: used cognitive behavioural procedures that have been found effective for alleviating chronic PTSD in assault survivors (Foa 1999; Foa 1991). These included 1) education about the normal reactions to assault; 2) breathing/relaxation training; 3) recounting the assault (imaginal exposure); 4) approaching feared, but safe, situations (in vivo exposure); and 5) CR. *Duration of intervention*: 4 weeks Humanistic (SC) *Planned number of intervention sessions*: 4 × 2‐hour sessions *Mode of delivery (face‐to‐face, online, video, telephone, blend, with explanation)*: face‐to‐face *Format (group, individual, blend)*: individual *Therapist qualifications and training appropriate (yes, no, unclear, with explanation)*: as previous *Research allegiance or conflict of interest (yes, no, unclear, with explanation)*: no *Treatment fidelity (yes, no, unclear, with explanation)*: yes, tapes of intervention sessions were reviewed in weekly supervision to ensure adherence. *Intervention aim and theoretical basis*: intervention consisted of active listening only. Neither discussion of assault‐related symptoms nor procedures aimed at promoting processing of the traumatic event were implemented. Also differed from assessment condition as there as no focus on symptoms *Duration of intervention*: 4 weeks Assessment control *Planned number of intervention sessions*: 4 × 2‐hour sessions *Mode of delivery (face‐to‐face, online, video, telephone, blend, with explanation)*: face‐to‐face *Format (group, individual, blend)*: individual *Therapist qualifications and training appropriate*: N/A *Research allegiance or conflict of interest*: N/A *Treatment fidelity*: N/A *Intervention aim and theoretical basis*: the AC condition consisted of 4 meetings weekly for 2 h each that focused on a thorough assessment of each of the PTSD symptoms and post‐assault functioning by a PTSD clinician. *Duration of intervention*: 4 weeks
Outcomes	**PTSD** Outcome type: continuous outcome Scale used: PSS‐I Direction: lower is better Score range: 0 to 51 Data value: endpoint, 3 months and 12 months **PTSD** Outcome type: continuous outcome Scale used: PSS‐SR Direction: lower is better Score range: 0 to 119 Data value: endpoint, 3 months and 12 months **Depression** Outcome type: continuous outcome Scale used: BDI Direction: lower is better Score range: 0 to 63 Data value: endpoint, 3 months and 12 months **Anxiety** Outcome type: continuous outcome Scale used: BAI Direction: lower is better Score range: 0 to 63 Data value: endpoint, 3 months and 12 months **Treatment dropout** Outcome type: dichotomous outcome **Trauma‐related beliefs** Outcome type: continuous outcome Scale used: PBRS‐self Direction: higher is better Score range: 0 to 6 Data value: endpoint, 12 months
Identification	**Sponsorship source:** NIMH grant MH42173 **Country:** USA **Setting:** urban clinic for the treatment and study of anxiety; recruitment through EDs and other medical settings, police, victim agencies and media **Authors name:** Edna Foa **Institution:** University of Pennsylvania; University of Washington; Case Western Reserve University **Email:** foa@mail.med.upenn.edu **Year:** 2006
Notes	The SC arm was added later, and systematic differences in the prevalence of sexual trauma were identified. Therefore, this group has been omitted. Not an ITT analysis, as data were only collected at follow‐up from intervention completers Included in meta‐analysis

**Table:** Galovski 2016

***Study characteristics***	
Methods	**Study design:** randomised controlled trial **Study grouping:** parallel group **Duration of study:** not given **Ethics approvals:** all study procedures were approved by the IRB at the University of Missouri‐St. Louis. **Location of study:** St. Louis, Missouri, USA **Randomisation method:** using a 1:1 allocation ratio, a computer‐generated randomisation sequence randomised eligible participants to the ssmCPT condition or sleep‐directed hypnosis condition.
Participants	**Baseline characteristics** CBT (CPT) *Participants (at randomisation)*: 56 *Gender*: 100% female participants *Age*: not given *Ethnicity*: not given *Disability*: not given *Time since trauma*: not given *Income*: not given *Education, M (SD) years*: 13.5 (3.19) *Employment*: not given *Sexual violence in adulthood*: not given *Comorbid conditions*: not given *Baseline PTSD, M (SD)*: 69.23 (15.74) *Partnered*: not given *Randomised (N)*: 56 *Completed post‐treatment assessment (N)*: 25 *Dropped out or removed prior to analysis (N)*: 21 *Numbers analysed at final applicable time point (N)*: 35 *Number of sessions*: not given *Treatment completion*: 25 (52%) CBT (CPT + hypnosis) *Participants (at randomisation)*: 52 *Gender*: 100% female participants *Age*: not given *Ethnicity*: not given *Disability*: not given *Time since trauma*: not given *Income*: not given *Education, M (SD) years*: 14.7 (2.46) *Employment*: not given *Sexual violence in adulthood*: not given *Comorbid conditions*: not given *Baseline PTSD, M (SD)*: 72.48 (15.01) *Partnered*: not given *Randomised (N)*: 52 *Completed post‐treatment assessment (N)*: 29 *Dropped out or removed prior to analysis (N)*: 22 *Numbers analysed at final applicable time point (N)*: 30 *Number of sessions*: not given *Treatment completion*: 26 (59%) Overall *Participants (at randomisation)*: 108 *Gender*: 100% female participants *Age, M (SD)*: 36.87 years (11.8 years) *Ethnicity*: 50% predominantly Black; 50% predominantly white; 20% mixed race; 3% Hispanic *Disability*: not given *Time since trauma, M (SD)*: 196 months (170.4 months) *Income*: 74% < USD 20,000 *Education (% high school, college or apprentice, university or years of education; M (SD) years)*: 50% at least some post‐high school education; 14.16 years (2.89 years) *Employment*: not given *Sexual violence in adulthood*: 63% sexual violence in adulthood; complex trauma histories, with lifetime endorsements of child sexual abuse 71%, child physical abuse 58%, adult criminal victimisation 32% and domestic violence 56% *Comorbid conditions*: not given *Baseline PTSD*: not given *Partnered*: 46% *Approached (N)*: 181 *Ineligible (N)*: 62 *Declined (N)*: 11 *Other (N)*: 0 *Randomised (N)*: 108 *Completed post‐treatment assessment (N)*: 54 *Dropped out or removed prior to analysis (N)*: 43 *Numbers analysed at final applicable time point (N)*: 65 *Number of sessions*: not given *Treatment completion*: 51 (47%) **Inclusion criteria** Female gender Diagnosis of PTSD secondary to sexual or physical assault Clinically significant sleep impairment as indicated by a severity score of 3 or more on the CAPS sleep impairment symptom At least 3 months post‐trauma at initial assessment Stable on any psychotropic medication for at least 1 month **Exclusion criteria** Psychosis Learning disability Active suicidality, parasuicidality Current drug or alcohol dependence Individuals currently in an abusive relationship or being stalked No prior therapy with CPT, or current trauma‐ or sleep‐focused therapy **Pretreatment:** years of education was the only difference detected, being slightly higher in the hypnosis group.
Interventions	**Intervention characteristics** CBT (cognitive processing theory) *Planned number of intervention sessions*: 12 *Mode of delivery (face‐to‐face, online, video, telephone, blend, with explanation)*: face‐to‐face *Format (group, individual, blend)*: individual *Therapist qualifications and training appropriate (yes, no, unclear, with explanation)*: unclear, questionable whether the therapists were sufficiently experienced to deliver the CPT and hypnosis, given the high level of dropout from treatment and average scores on independent ratings, although study was strong on supervision and fidelity checks. *Research allegiance or conflict of interest (yes, no, unclear, with explanation)*: no *Treatment fidelity (yes, no, unclear, with explanation)*: yes, the CPT and hypnosis manuals and relevant readings were provided in the training. Expert CPT clinicians not otherwise affiliated with the study reviewed and rated treatment sessions for adherence and competence. Scores were acceptable, although some concerns around the drop out rate for 1 therapist and unclear if detected in the fidelity assessment *Intervention aim and theoretical basis*: following the 3 weeks of daily symptom monitoring, ssmCPT participants received CPT (see Resick 2010 for a complete protocol). CPT is predominantly a cognitive therapy in which patients are taught to identify, question, challenge and replace faulty assumptions and thoughts about the traumatic event and its implications in their current lives. While the patients engage with the traumatic memory, they are able to allow natural affect to run its course. CPT first targets specific, inaccurate interpretations of the trauma itself and then targets current and future maladaptive and inaccurate beliefs about world, self and others across a number of belief systems typically disrupted after experiencing a traumatic event. *Duration of intervention*: 15 weeks; 3 weeks of symptom monitoring plus 12 weeks of CPT CBT (CPT + hypnosis) *Planned number of intervention sessions*: 15 *Mode of delivery (face‐to‐face, online, video, telephone, blend, with explanation)*: face‐to‐face *Format (group, individual, blend)*: individual *Therapist qualifications and training appropriate (yes, no, unclear, with explanation)*: as before *Research allegiance or conflict of interest (yes, no, unclear, with explanation)*: no *Treatment fidelity (yes, no, unclear, with explanation)*: as before *Intervention aim and theoretical basis*: researchers hypothesised that improvements in sleep realised during hypnosis would augment CPT, such that PTSD symptom reduction (during CPT) would be greater in the CPT + hypnosis condition relative to the CPT condition. Hypnosis was conducted in accordance with a manual devised for the trial following Wright 1987. *Duration of intervention*: 15 weeks
Outcomes	**PTSD** Outcome type: continuous outcome Scale used: CAPS Direction: lower is better Score range: 0 to 80 Data value: endpoint, 3 months **Depression** Outcome type: continuous outcome Scale used: BDI‐II Direction: lower is better Score range: 0 to 63 Data value: endpoint, 3 months **Treatment dropout** Outcome type: dichotomous outcome
Identification	**Sponsorship source:** National Institutes of Health **Country:** USA **Setting:** community sample recruited through flyers, advertisements, referrals and word‐of‐mouth; academic setting for intervention **Authors name:** Tara E Galovski **Institution:** University of Missouri **Email:** galovskit@msx.umsl.edu **Year:** 2016
Notes	Not included in meta‐analysis

**Table:** Gray 2020

***Study characteristics***	
Methods	**Study design:** randomised controlled trial **Study grouping:** parallel group **Duration of study:** recruiting began during November 2015 and was completed by mid‐May 2016; all treatments were completed by June 2016. Follow‐ups to 1 year were completed by August 2017. **Ethics approvals:** the study protocol and informed consent were approved by the NEIRB. Following NEIRB guidelines, the protocol and all aspects of participation were reviewed with participants, and signed informed consent was obtained from each participant. **Location of study:** suburban municipality in northern San Diego County, California, USA **Randomisation method:** participants were admitted to the study in cohorts of 10 and randomly assigned to treatment or control groups by the site manager. This assignment was based on a list of random numbers, previously generated at an independent location using Microsoft Excel 2016’s random number function.
Participants	**Baseline characteristics** Behavioural (RTM) *Participants (at randomisation)*: 15 *Gender*: not given *Age*: not given *Ethnicity*: not given *Disability*: not given *Time since trauma*: not given *Income*: not given *Education*: not given *Employment*: not given *Sexual violence in adulthood*: not given *Comorbid conditions*: not given *Baseline PTSD, M (SD)*: 43.6 (2.5) *Partnered*: not given *Randomised (N)*: 15 *Completed post‐treatment assessment (N)*: 14 *Dropped out or removed prior to analysis (N)*: 1 *Numbers analysed at final applicable time point (N)*: 14 *Number of sessions, M (SD)*: 3 (0) *Treatment completion*: 15 (100%) Wait‐list *Participants (at randomisation)*: 15 *Gender*: not given *Age in years*: not given *Ethnicity*: not given *Disability*: not given *Time since trauma*: not given *Income*: not given *Education*: not given *Employment*: not given *Sexual violence in adulthood*: not given *Comorbid conditions*: not given *Baseline PTSD, M (SD)*: 43.6 (3.5) *Partnered*: not given *Randomised (N)*: 15 *Completed post‐treatment assessment (N)*: 15 *Dropped out or removed prior to analysis (N)*: 0 *Numbers analysed at final applicable time point (N)*: 15 *Number of sessions*: N/A *Treatment completion*: N/A Overall *Participants (at randomisation)*: 30 *Gender*: 100% female participants *Age, M (SD)*: 33.7 years (14 years) *Ethnicity*: 76% Caucasian (understood to be white), 7% Native American, 10% Hispanic, 3% African American, 3% Asian *Disability*: not given *Time since trauma*: not given *Income*: not given *Education*: not given *Employment (% employed; unemployed, other)*: 47% employed *Sexual violence in adulthood*: 73% *Comorbid conditions*: not given *Baseline PTSD*: not given *Partnered*: not given *Approached (N)*: 46 *Ineligible (N)*: 10 *Declined (N)*: 6 *Other (N)*: 0 *Randomised (N)*: 30 *Completed post‐treatment assessment (N)*: 29 *Dropped out or removed prior to analysis (N)*: 1 *Numbers analysed at final applicable time point (N)*: 29 *Number of sessions*: N/A *Treatment completion*: N/A **Inclusion criteria** Symptom assessments for PTSD above commonly used diagnostic thresholds (PSS‐I > 20) Autonomic arousal observable to the interviewer (e.g. tearing, tensing, flushing, tremors, hesitation, changes in voice tonality) while the participant recounts the index trauma Reports of at least 1 flashback or nightmare during the preceding month **Exclusion criteria** Comorbid DSM‐IV Axis I or II disorders sufficiently severe to interfere with the participant’s ability to complete treatment PTSD symptoms judged by the interviewer or clinician to be part of the participant’s identity structure Individuals judged by the interviewer or clinician to be incapable of sustained attention, whether through florid psychosis, inebriation or other observable alterations of consciousness; persons unable to identify a discreet traumatic event for treatment targeting **Pretreatment:** none reported
Interventions	**Intervention characteristics** Behavioural (RTM) *Planned number of intervention sessions*: 3 (120 min each) *Mode of delivery (face‐to‐face, online, video, telephone, blend, with explanation)*: face‐to‐face *Format (group, individual, blend)*: individual *Therapist qualifications and training appropriate (yes, no, unclear, with explanation)*: yes, 1 credentialed PhD‐level clinical psychologist, experienced in delivering the RTM protocol, delivered the treatments. *Research allegiance or conflict of interest (yes, no, unclear, with explanation)*: yes, the authors were involved in the original development. *Treatment fidelity (yes, no, unclear, with explanation)*: yes, all screening and treatment sessions were video‐recorded on secure digital media and stored on HIPAA‐compliant cloud servers for assessment of treatment fidelity. Three experts in the administration of the RTM protocol (2 PhD‐level psychologists and 1 with a Master of Social Work degree) periodically reviewed treatment videos, evaluating them for 1) adherence to the RTM procedure and 2) skills used by the clinician to track the client’s arousal levels. Raters recorded compliance information using a standardised, in‐house, 20‐element checklist. The level of compliance was found to be high. *Intervention aim and theoretical basis*: RTM relies upon the hypothesis that trauma memories may be reactivated and updated using the reconsolidation mechanism (Gray 2019; Lee 2017; Nader 2000; Tylee 2017). After an activation that is too brief to support extinction, the target memory is believed to become malleable, and new information, relevant to the perceived threat, can be incorporated into its structure (Fernández 2016; Suzuki 2004; Tylee 2017). We hypothesise that reconsolidation may be used to change structural elements of the memory related to its perceptual salience and, by reducing the impact of the memory, render it nontraumatising. *Duration of intervention*: 3 weeks Wait‐list *Planned number of intervention sessions*: N/A *Mode of delivery*: N/A *Format*: N/A *Therapist qualifications and training appropriate*: N/A *Research allegiance or conflict of interest*: N/A *Treatment fidelity*: N/A *Intervention aim and theoretical basis*: N/A *Duration of intervention*: N/A
Outcomes	**PTSD** Outcome type: continuous outcome Scale used: PSS‐I Direction: lower is better Score range: 0 to 51 Data value: endpoint **Adverse events** Outcome type: dichotomous outcome **Treatment dropout (intervention only)** Outcome type: dichotomous outcome
Identification	**Sponsorship source:** this research was supported by a grant from the Blue Angels Foundation. **Country:** USA **Setting:** female US veterans and active‐duty service members recruited from veterans’ groups and mental health services; all treatments and evaluations were performed in a private office suite dedicated to the study in a professional office complex. **Comments:** N/A **Authors name:** Richard Gray **Institution:** Research and Recognition Project, Corning, New York **Email:** richard.gray@randrproject.com **Year:** 2020
Notes	Communicated with the authors(s) as part of the review Included in meta‐analysis

**Table:** Katz 2014

***Study characteristics***	
Methods	**Study design:** randomised controlled trial **Study grouping:** parallel group **Duration of study:** not given **Ethics approvals:** study approved by local internal review board **Location of study:** Seattle, Washington, USA **Randomisation method:** not given
Participants	**Baseline characteristics** CBT (PE) *Participants (at randomisation)*: 17 *Gender*: 100% female participants *Age, M*: 36 years *Ethnicity*: 12% African American, 12% Hispanic, 41% white non‐Hispanic, 35% missing information *Disability*: 71% (This was reported with those who were unemployed and unable to work or find work.) *Time since trauma*: not given *Income*: not given *Education (% high school, college or apprentice, university or years of education)*: 59% college or higher *Employment (% employed, unemployed, other)*: 12% employed *Sexual violence in adulthood*: 47% *Comorbid conditions*: not given *Baseline PTSD, M (SD)*: 66.2 (12.9) *Partnered*: 6% *Randomised (N)*: 17 *Completed post‐treatment assessment (N)*: 10 *Dropped out or removed prior to analysis (N)*: 7 *Numbers analysed at final applicable time point (N)*: 10 *Number of sessions, M*: 5.2 *Treatment completion*: 10 (59%) Integrative therapy (holographic reprocessing) *Participants (at randomisation)*: 17 *Gender*: 100% female participants *Age, M*: 45 years *Ethnicity*: 24% African American, 6% Hispanic, 41% white non‐Hispanic, 29% missing information *Disability*: 47% (This was reported with those who were unemployed and unable to work or find work.) *Time since trauma*: not given *Income*: not given *Education (% high school, college or apprentice, university or years of education)*: 42% college or higher *Employment (% employed; unemployed, other)*: 24% employed *Sexual violence in adulthood*: 71% *Comorbid conditions*: not given *Baseline PTSD, M (SD)*: 54.31 (13.6) *Partnered*: 24% *Randomised (N)*: 17 *Completed post‐treatment assessment (N)*: 16 *Dropped out or removed prior to analysis (N)*: 1 *Numbers analysed at final applicable time point (N)*: 16 *Number of sessions, M*: 10 *Treatment completion*: 16 (94%) Humanist (person‐centred therapy) *Participants (select at randomisation if available)*: 17 *Gender*: 100% female participants *Age, M*: 42 years *Ethnicity*: 24% African American, 17% Hispanic, 47% white non‐Hispanic, 12% missing information *Disability*: 41% (This was reported with those who were unemployed and unable to work or find work.) *Time since trauma*: not given *Income*: not given *Education (% high school, college or apprentice, university or years of education)*: 54% college or higher *Employment (% employed, unemployed, other)*: 24% employed *Sexual violence in adulthood*: 41% *Comorbid conditions*: not given *Baseline PTSD, M (SD)*: 51.6 (17.8) *Partnered*: 29% *Randomised (N)*: 17 *Completed post‐treatment assessment (N)*: 11 *Dropped out or removed prior to analysis (N)*: 6 *Numbers analysed at final applicable time point (N)*: 11 *Number of sessions*: not given *Treatment completion*: 11 (65%) Overall *Participants (at randomisation)*: 51 *Gender*: 100% female participants *Age, M (SD)*: 42 years (12.34 years) *Ethnicity*: 20% African American, 12% Hispanic; 43% white non‐Hispanic, 25% missing information *Disability*: 53% (This was reported with those who were unemployed and unable to work or find work.) *Time since trauma*: not given *Income*: not given *Education (% high school, college or apprentice, university or years of education)*: 48% college or higher *Employment (% employed, unemployed, other)*: 20% employed *Sexual violence in adulthood*: 53% sexual violence in adulthood, 86% MST, 71% domestic abuse, 80% child sexual abuse *Comorbid conditions*: not given *Baseline PTSD*: not given *Partnered*: 20% *Approached (N)*: 53 *Ineligible (N)*: 2 *Declined (N)*: 0 *Other (N)*: 0 *Randomised (N)*: 51 *Completed post‐treatment assessment (N)*: 37 *Dropped out or removed prior to analysis (N)*: 14 *Numbers analysed at final applicable time point (N)*: 37 *Number of sessions*: not given *Treatment completion*: 37 (73%) **Inclusion criteria:** history of sexual trauma (e.g. childhood, adult or military sexual assault; molestation; or domestic violence) **Exclusion criteria** Suicidal attempts or hospitalisations in the last 6 months prior to treatment Psychotic symptoms or suffering from a psychotic‐related disorder Actively using alcohol or drugs for at least 3 months prior to the study Strong tendency to dissociate to the point that it could interfere with ability to participate in this study **Pretreatment:** none reported
Interventions	**Intervention characteristics** CBT (PE) *Planned number of intervention sessions*: 10 *Mode of delivery (face‐to‐face, online, video, telephone, blend, with explanation)*: face‐to‐face *Format (group, individual, blend)*: individual *Therapist qualifications and training appropriate (yes, no, unclear, with explanation)*: yes, trainees were given weekly individual supervision by the licenced psychologist. *Research allegiance or conflict of interest (yes, no, unclear, with explanation)*: no *Treatment fidelity (yes, no, unclear, with explanation)*: yes, as a post hoc fidelity test, 2 chart notes were randomly printed for each participant in the study. Two raters (certified research assistants) sorted the notes into the 3 groups following the guidelines of a brief written description of each of the 3 therapies. Both sorted the notes with 100% accuracy. Based on supervision and documentation of treatment, it appears that the therapists in this study (minus 1) followed their respective protocols. *Intervention aim and theoretical basis*: using imaginal exposure and in vivo exposure, clients reduce their fear, desensitise to memories, and learn that they can venture to places previously avoided. *Duration of intervention*: 10 weeks Integrative therapy (holographic reprocessing) *Planned number of intervention sessions*: 10 *Mode of delivery (face‐to‐face, online, video, telephone, blend, with explanation)*: face‐to‐face *Format (group, individual, blend)*: individual *Therapist qualifications and training appropriate (yes, no, unclear, with explanation)*: yes, trainees were given weekly individual supervision by the licenced psychologist. *Research allegiance or conflict of interest (yes, no, unclear, with explanation)*: yes, the lead researcher also designed the therapy, and it is unclear if that researcher also delivered the therapies and if other therapists were listed as co‐authors. *Treatment fidelity (yes, no, unclear, with explanation)*: yes *Intervention aim and theoretical basis*: holographic reprocessing uses a low‐arousing method of revisiting a formative event (e.g. representative of the person’s hologram) to facilitate holistic reappraisal and insight. In reprocessing, participants do not revisit an act of trauma itself, as this is not an exposure treatment. Clients are instructed to recall the event as a distanced observer, describing it from the third person and noticing a larger context for the event, which facilitates broad cognitive reappraisals. *Duration of intervention*: 10 weeks Humanist (person‐centred therapy) *Planned number of intervention sessions*: 10 *Mode of delivery (face‐to‐face, online, video, telephone, blend, with explanation)*: face‐to‐face *Format (group, individual, blend)*: individual *Therapist qualifications and training appropriate (yes, no, unclear, with explanation)*: yes, trainees were given weekly individual supervision by the licenced psychologist. *Research allegiance or conflict of interest (yes, no, unclear, with explanation)*: no *Treatment fidelity (yes, no, unclear, with explanation)*: yes *Intervention aim and theoretical basis*: PCT focuses on providing support and discussing and understanding here‐and‐now issues that are generated by clients. *Duration of intervention*: 10 weeks
Outcomes	**PTSD** Outcome type: continuous outcome Scale used: PCL‐C Direction: lower is better Score range: 17 to 85 Data value: endpoint **Global mental health** Outcome type: continuous outcome Scale used: BSI Direction: lower is better Score range: 0 to 112 Data value: endpoint **Depression** Outcome type: continuous outcome Scale used: BSI Direction: lower is better Score range: 0 to 112 Data value: endpoint **Anxiety** Outcome type: continuous outcome Scale used: BSI Direction: lower is better Score range: 0 to 112 Data value: endpoint **Self‐blame** Outcome type: continuous outcome Scale used: PTCI Direction: lower is better Score range: 33 to 231 Data value: endpoint **Adverse events** Outcome type: adverse event **Treatment dropout** Outcome type: dichotomous outcome **Trauma‐related beliefs** Outcome type: continuous outcome Scale used: PCTI‐total Direction: lower is better Score range: 33 to 231 Data value: endpoint
Identification	**Sponsorship source**: not given **Country:** USA **Setting:** VA medical centre (women's mental health clinic); female veterans with sexual trauma histories seeking psychotherapy **Authors name:** Lori S Katz **Institution:** VA Puget Sound Healthcare System **Email:** lori.katz@va.gov **Year:** 2014
Notes	Included in meta‐analysis

**Table:** Kelly 2021

***Study characteristics***	
Methods	**Study design:** randomised controlled trial **Study grouping:** parallel **Duration of study:** enrolment from 2016 to 2020 **Ethics approvals:** the study was approved by the Emory University IRB and the appropriate VA committees **Location of study:** Atlanta, Georgia, USA **Randomisation method:** participants within each of 9 cohorts were randomised to the 2 groups by the block randomisation algorithm "random sorting using maximum allowable percent deviation" using the PASS v.15 power analysis software package. Randomisation sequences were generated for each cohort, initially in blocks of 24, then in 20 based on enrolment numbers. Using the randomisation sequences, the study co‐ordinator set up sequentially numbered, sealed envelopes containing randomisation assignment for use at each baseline data collection. Once participants completed baseline data collection, the data collector opened the envelope to determine randomisation allocation and informed the participant and study co‐ordinator.
Participants	**Baseline characteristics** Other psychologically oriented interventions (trauma‐sensitive yoga) *Participants (at randomisation)*: 58 *Gender*: not given *Age*: not given *Ethnicity*: not given *Disability*: not given *Time since trauma*: not given *Income*: not given *Education*: not given *Employment*: not given *Sexual violence in adulthood*: not given *Comorbid conditions*: not given *Baseline PTSD, M (SD)*: 50.80 (11.74) *Partnered*: not given *Randomised (N)*: 58 *Completed post‐treatment assessment (N)*: 38 *Dropped out or removed prior to analysis (N)*: 20 *Numbers analysed at final applicable time point (N)*: 28 *Number of sessions*: not given *Treatment completion*: 35 (60.3%) CBT (CPT) *Participants (at randomisation)*: 46 *Gender*: not given *Age*: not given *Ethnicity*: not given *Disability*: not given *Time since trauma*: not given *Income*: not given *Education*: not given *Employment*: not given *Sexual violence in adulthood*: not given *Comorbid conditions*: not given *Baseline PTSD, M (SD)*: 50.37 (12.11) *Partnered*: not given *Randomised (N)*: 46 *Completed post‐treatment assessment (N)*: 20 *Dropped out or removed prior to analysis (N)*: 26 *Numbers analysed at final applicable time point (N)*: 19 *Number of sessions*: not given *Treatment completion*: 16 (34.8%) Overall *Participants (at randomisation)*: 104 *Gender*: 100% female participants *Age, M (SD)*: 48.38 years (11.1 years) *Ethnicity*: 91.3% African American *Disability*: not given *Time since trauma*: not given *Income*: 54% USD 2000 or more per month *Education (% high school, college or apprentice, university or years of education)*: 16 years (median) *Employment*: not given *Sexual violence in adulthood*: 100% *Comorbid conditions*: not given *Baseline PTSD*: not given *Partnered*: 28.2% *Approached (N)*: 466 *Ineligible (N)*: 225 *Declined (N)*: 89 *Other (N)*: 48 *Randomised (N)*: 104 *Completed post‐treatment assessment (N)*: 58 *Dropped out or removed prior to analysis (N)*: 46 *Numbers analysed at final applicable time point (N)*: 47 *Number of sessions*: N/A *Treatment completion*: N/A **Inclusion criteria:** PTSD related to MST diagnosed in the PTSD clinic or in the first study assessment. MST is defined as the experience of sexual assault or repeated threatening sexual harassment during military service. **Exclusion criteria** Severe psychosis; current, active suicidal intent or plan Current moderate or severe alcohol or substance use disorder Moderate or severe cognitive impairment Current engagement in trauma‐focused treatment or yoga practice or other activity at odds with the study interventions **Pretreatment:** none reported
Interventions	**Intervention characteristics** Other psychologically oriented interventions (trauma‐sensitive yoga) *Planned number of intervention sessions*: 10 × 1 h *Mode of delivery (face‐to‐face, online, video, telephone, blend, with explanation)*: face‐to‐face *Format (group, individual, blend)*: group *Therapist qualifications and training appropriate (yes, no, unclear, with explanation)*: yes, David Emerson, the developer of TCTSY, trained the yoga facilitators and provided weekly consultation to them during the 9 intervention cohorts *Research allegiance or conflict of interest (yes, no, unclear, with explanation)*: no *Treatment fidelity (yes, no, unclear, with explanation)*: unclear, no explanation *Intervention aim and theoretical basis*: the theoretical framework of the study is psychoneuroimmulogic. Yoga is theorised to have the opposite effects on the autonomic nervous system and stress response than the dysregulating effects associated with PTSD. TCTSY, the experimental intervention in this randomised controlled trial, was developed for civilian women survivors of complex trauma, specifically childhood sexual trauma, with chronic PTSD. TCTSY focuses on interoception – that is, the sense of the physiological condition of the body – in Hatha style yoga and addresses themes related to establishing safety, individual choice, being in the present moment and taking effective action *Duration of intervention*: 10 weeks CBT (CPT) *Planned number of intervention sessions*: 12 × 90 min *Mode of delivery (face‐to‐face, online, video, telephone, blend, with explanation)*: face‐to‐face *Format (group, individual, blend)*: group *Therapist qualifications and training appropriate (yes, no, unclear, with explanation)*: yes, the CPT intervention was conducted by VA clinicians (psychologists and licenced clinical social workers) certified in CPT. *Research allegiance or conflict of interest (yes, no, unclear, with explanation)*: no *Treatment fidelity (yes, no, unclear, with explanation)*: unclear, no explanation *Intervention aim and theoretical basis*: the sessions focused on identifying how thoughts change as a result of trauma exposure and ways in which to evaluate maladaptive thoughts and create alternative thoughts. *Duration of intervention*: 12 weeks
Outcomes	**PTSD** Outcome type: continuous outcome Scale used: CAPS‐5 Direction: lower is better Score range: 0 to 80 Data value: endpoint, 3 months **PTSD** Outcome type: continuous outcome Scale used: PCL‐5 Direction: lower is better Score range: 0 to 80 Data value: endpoint, 3 months **Adverse events** Outcome type: adverse event **Treatment dropout** Outcome type: dichotomous outcome Direction: lower is better Notes: 2 participants in the CPT group withdrew due to increased psychological distress and were referred for individual therapy.
Identification	**Sponsorship source:** this material is based upon work supported by the Department of Veterans Affairs, VHA, Office of Research and Development, Health Services Research and Development, grant no. 5I01HX001087‐02 **Country:** USA **Setting:** women veterans with PTSD related to MST who were service users of a VA Health Care System (outpatients); Women’s Wellness Clinics; and Primary Care Clinics **Authors name:** Ursula Kelly **Institution:** Atlanta VA Health Care System **Email:** Ursula.Kelly@va.gov **Year:** 2021
Notes	Included in meta‐analysis ClinicalTrials.gov: NCT02640690

**Table:** Krakow 2001

***Study characteristics***	
Methods	**Study design:** randomised controlled trial **Study grouping:** parallel group **Duration of study:** 4 years (1995 to 1999) **Ethics approvals:** University of New Mexico Health Sciences Center IRB **Location of study:** New Mexico, USA **Randomisation method:** participants mailed a postcard, and the postcard's time and date were logged into a computer and entered into a previously generated list of numbers that randomly assigned participants.
Participants	**Baseline characteristics** CBT (imagery rehearsal therapy) *Participants (at randomisation)*: 88 *Gender*: 100% female participants *Age, M (SD)*: completer 40 years (11.2 years), non‐completer 37 years (12.7 years) *Ethnicity*: 69% non‐Hispanic white, 31% other (mostly Hispanic) *Disability*: not given *Time since trauma*: not given *Income*: 49% < USD 10,000, 51% > USD 10,000 *Education (% high school, college or apprentice, university or years of education)*: 38% college degree *Employment*: not given *Sexual violence in adulthood*: 100% *Comorbid conditions*: not given *Baseline PTSD, M (SD)*: 81.88 (16.96) *Partnered*: 38% *Randomised (N)*: 88 *Completed post‐treatment assessment (N)*: 44 *Dropped out or removed prior to analysis (N)*: 34 *Numbers analysed at final applicable time point (N)*: 54 *Number of sessions*: not given *Treatment completion*: 66 (75%) Wait‐list *Participants (select at randomisation if available)*: 80 *Gender*: 100% female participants *Age, M (SD)*: completer 36 years (9.3 years), non‐completer 31 years (10.5 years) *Ethnicity*: 55% non‐Hispanic white, 45% other (mostly Hispanic) *Disability*: not given *Time since trauma*: not given *Income*: 40% < USD 10,000, 60% > USD 10,000 *Education (% high school, college or apprentice, university or years of education)*: 39% college degree *Employment*: not given *Sexual violence in adulthood*: 100% *Comorbid conditions*: not given *Baseline PTSD, M (SD)*: 79.62 (24.37) *Partnered*: 43% *Randomised (N)*: 80 *Completed post‐treatment assessment (N)*: 52 *Dropped out or removed prior to analysis (N)*: 20 *Numbers analysed at final applicable time point (N)*: 60 *Number of sessions*: N/A *Treatment completion*: N/A Overall *Participants (at randomisation)*: 168 *Gender*: 100% female participants *Age*: not given *Ethnicity*: 62.5% non‐Hispanic white, 37.5% other (mostly Hispanic) *Disability*: not given *Time since trauma*: not given *Income*: 44% < USD 10,000, 56% > USD 10,000 *Education (% high school, college or apprentice, university or years of education)*: 38% college degree *Employment*: not given *Sexual violence in adulthood*: 100% sexual violence in adulthood, 58% multiple exposures, 90% abused as children *Comorbid conditions*: not given *Baseline PTSD*: 85% reported CAPS score > 65 *Partnered*: 40% *Approached (N)*: 203 *Ineligible (N)*: 0 *Declined (N)*: 35 *Other (N)*: 0 *Randomised (N)*: 168 *Completed post‐treatment assessment (N)*: 96 *Dropped out or removed prior to analysis (N)*: 54 *Numbers analysed at final applicable time point (N)*: 114 *Number of sessions*: N/A *Treatment completion*: N/A **Inclusion criteria** Female sexual assault survivors 18 years or older Self‐reported nightmares (at least once a week for more than 6 months) Insomnia PTSD coupled with criterion A trauma link (DSM‐IV) **Exclusion criteria** Acute intoxication Withdrawal Psychosis **Pretreatment:** no statistically significant baseline differences with exception of age, whereby control non‐completers were younger than treatment completers
Interventions	**Intervention characteristics** CBT (imagery rehearsal therapy) *Planned number of intervention sessions*: 3 (2 × 3‐hour sessions spaced 1 week apart with a 1‐hour follow‐up 3 weeks later) *Mode of delivery (face‐to‐face, online, video, telephone, blend, with explanation)*: face‐to‐face *Format (group, individual, blend)*: group *Therapist qualifications and training appropriate (yes, no, unclear, with explanation)*: yes, although not described in any great detail, the lead author is an expert in the field of IRT for PTSD and sleep disorders. There was minimal reference to supervision and training. Treatment followed a manual and focused on nightmares within the framework of an imagery and CR paradigm. *Research allegiance or conflict of interest (yes, no, unclear, with explanation)*: yes, cognitive‐imagery treatment was previously tested in studies of the authors. Author links to both not‐for‐profit and for‐profit sleep centres. *Treatment fidelity (yes, no, unclear, with explanation)*: no, information for assessing fidelity not provided *Intervention aim and theoretical basis*: to determine if treating chronic nightmares with imagery rehearsal therapy reduces the frequency of disturbing dreams, improves sleep quality and decreases PTSD symptom severity. Involves story line alteration of the nightmare while awake, followed by rehearsal of a new set of images *Duration of intervention*: 4 weeks Wait‐list *Planned number of intervention sessions*: N/A *Mode of delivery*: N/A *Format*: N/A *Therapist qualifications and training appropriate*: N/A *Research allegiance or conflict of interest*: N/A *Treatment fidelity*: N/A *Intervention aim and theoretical basis*: contact with control participants was limited to brief telephone calls and letters to remind them of future appointments. All controls continued any treatment they were already receiving and were offered treatment at no charge on completion of their 6‐month wait‐list period. *Duration of intervention*: N/A
Outcomes	**Adverse events** Outcome type: adverse event Notes: IRT can produce imagery adverse effects, and 4 participants reported increased negative imagery and eventually withdrew; 12 of 66 who completed treatment did not complete follow‐up for unknown reasons. **Treatment dropout (intervention only)** Outcome type: dichotomous outcome **PTSD** Outcome type: continuous outcome Scale used: CAPS Direction: lower is better Score range: 0 to 80 Data value: endpoint
Identification	**Sponsorship source:** NIMH (MH53239) and University of New Mexico Health Sciences Centre Research Allocation Committee **Country:** USA **Setting:** academic setting for intervention delivery; sample was community, rape crisis and mental health services **Authors name:** Barry Krakow **Institution:** University of New Mexico Health Sciences Centre **Email:** bkrakow@salud.unm.edu **Year:** 2001
Notes	Included in meta‐analysis

**Table:** Littleton 2016

***Study characteristics***	
Methods	**Study design:** randomised controlled trial **Study grouping:** parallel group **Duration of study:** May 2012 to August 2014 **Ethics approvals:** approval from 4 IRBs at the universities and colleges where recruitment took place **Location of study:** 4 US universities and community colleges, including East Carolina **Randomisation method:** participants were randomised to the interactive programme or psychoeducational website based on a computerised coin flip.
Participants	**Baseline characteristics** CBT (CBT‐online) *Participants (at randomisation)*: 46 *Gender*: 100% female participants *Age*: not given *Ethnicity*: 41.3% white, 21.7% Black, 38.3% other *Disability*: not given *Time since trauma*: not given *Income*: not given *Education (% high school, college or apprentice, university or years of education)*: all college students *Employment*: not given *Sexual violence in adulthood*: 100% *Comorbid conditions*: not given *Baseline PTSD, M (SD)*: 23.7 (6.5) *Partnered*: not given *Randomised (N)*: 46 *Completed post‐treatment assessment (N)*: 26 *Dropped out or removed prior to analysis (N)*: 24 *Numbers analysed at final applicable time point (N)*: 22 *Number of sessions*: not given *Treatment completion*: 26 (57%) Minimal intervention (psychoeducation website) *Participants (select at randomisation if available)*: 41 *Gender*: 100% female participants *Age*: not given *Ethnicity*: 51.2% white, 29.3% Black, 14.6% other *Disability*: not given *Time since trauma*: not given *Income*: not given *Education (% high school, college or apprentice, university or years of education)*: all college students *Employment*: not given *Sexual violence in adulthood*: 100% *Comorbid conditions*: not given *Baseline PTSD, M (SD)*: 23 (7.3) *Partnered*: not given *Randomised (N)*: 41 *Completed post‐treatment assessment (N)*: 29 *Dropped out or removed prior to analysis (N)*: 20 *Numbers analysed at final applicable time point (N)*: 21 *Number of sessions*: not given *Treatment completion*: 29 (71%) Overall *Participants (at randomisation)*: 87 *Gender*: 100% female participants *Age, M (range)*: 22 years (18 years to 42 years) *Ethnicity*: 46% white, 25.3% Black, 20.7% other *Disability*: not given *Time since trauma, M*: < 4 years *Income*: not given *Education (% high school, college or apprentice, university or years of education)*: all college students *Employment*: not given *Sexual violence in adulthood*: 100% *Comorbid conditions*: 56.2% *Baseline PTSD*: not given *Partnered*: not given *Approached (N)*: 150 *Ineligible (N)*: 60 *Declined (N)*: 4 *Other (N)*: 1 *Randomised (N)*: 87 *Completed post‐treatment assessment (N)*: 55 *Dropped out or removed prior to analysis (N)*: 44 *Numbers analysed at final applicable time point (N)*: 43 **Inclusion criteria** Participants had to have experienced a completed rape since the age of 14 years. Participants had to have a PTSD diagnosis determined using the PSS‐I (Foa 1993). Regular access to a computer and a telephone number at which they could be reliably reached Currently a student enrolled at one of the universities involved in the study **Exclusion criteria** Currently receiving psychotherapy Lack of stability on psychotropic medication (not been on current medication and dosage for at least 3 months) Active suicidality as determined by interview utilising the Scale for Suicidal Ideation (Beck 1979) Meeting DSM‐IV criteria for current substance dependence assessed with substance use disorder module of Structured Clinical Interview for the DSM‐IV Too acutely distressed to participate **Pretreatment:** none identified, with the exception that women assigned to the interactive programme were more likely to report that the perpetrator used severe force
Interventions	**Intervention characteristics** CBT (online) *Planned number of intervention sessions*: 9 *Mode of delivery (face‐to‐face, online, video, telephone, blend, with explanation)*: online with feedback from therapist on exercises *Format (group, individual, blend)*: individual *Therapist qualifications and training appropriate (yes, no, unclear, with explanation)*: unclear, study staff are referred to as doctoral clinical psychology students or postdoctoral fellows, but training not specified *Research allegiance or conflict of interest (yes, no, unclear, with explanation)*: unclear, but presume they have developed the online programme *Treatment fidelity (yes, no, unclear, with explanation)*: yes, external raters reviewed therapeutic feedback provided to participants, which was "uniformly high", although unclear who they are or against what criteria *Intervention aim and theoretical basis*: Survivor to Thriver Program consists of sequential modules, focusing on psychoeducation in modules 1 to 3; modules 4 and 5 introduce the cognitive model, teaching participants to identify distorted and unhelpful thoughts; the third phase focused on using a number of CBT techniques to address specific concerns among women following sexual assault. *Duration of intervention*: 14 weeks Minimal intervention (psychoeducation website) *Planned number of intervention sessions*: 3; identical to Sessions 1 to 3 in other arm *Mode of delivery (face‐to‐face, online, video, telephone, blend, with explanation)*: online *Format (group, individual, blend)*: individual *Therapist qualifications and training appropriate*: N/A *Research allegiance or conflict of interest*: N/A *Treatment fidelity*: N/A *Intervention aim and theoretical basis*: the psychoeducational website contained the written content of the first 3 modules of the interactive programme, including the symptoms of PTSD, information about relaxing and grounding and information about healthy coping strategies. *Duration of intervention*: 14 weeks
Outcomes	**PTSD** Outcome type: continuous outcome Scale used: PSS‐I Direction: lower is better Score range: 0 to 51 Data value: endpoint, 3 months **Depression** Outcome type: continuous outcome Scale used: CES‐D Direction: lower is better Score range: 0 to 60 Data value: endpoint, 3 months **Anxiety** Outcome type: continuous outcome Scale used: Four‐Dimensional Anxiety Scale Direction: lower is better Score range: 35 to 175 Data value: endpoint, 3 months **Adverse events** Outcome type: continuous outcome Notes: adverse events – 2 in interactive programme and 1 in psychoeducational arm had increased depressive symptoms; 1 participant in interactive programme had increased anxiety at follow‐up. **Treatment dropout** Outcome type: dichotomous outcome
Identification	**Sponsorship source:** the research was supported by funding from the NIMH grant number 1R34MH085118. **Country:** USA **Setting:** university students with rape‐related PTSD; online therapy **Authors name:** Heather Littleton **Institution:** East Carolina University **Email:** hlittlet@uccs.edu **Year:** 2017
Notes	Numbers in population characteristics are at randomisation. Number approached less ineligible is out by 1, so we added 1 to the 'Other' category. Included in meta‐analysis

**Table:** Miller 2015

***Study characteristics***	
Methods	**Study design:** randomised controlled trial **Study grouping:** parallel group **Duration of study:** 3 years **Ethics approvals:** procedures were approved by a university IRB **Location of study:** Tulsa, Oklahoma, USA **Randomisation method:** not given
Participants	**Baseline characteristics** Other psychologically oriented interventions (psychoeducation and coping video) *Participants (receiving intervention)*: 79 *Gender*: 100% female participants *Age*: not given *Ethnicity*: not given *Disability*: not given *Time since trauma, M*: < 72 h *Income*: not given *Education*: not given *Employment*: not given *Sexual violence in adulthood*: 100% *Comorbid conditions*: not given *Baseline PTSD, M (SD)*: 25.82 (15.06) *Partnered*: not given *Randomised (N)*: 94 *Completed post‐treatment assessment (N)*: 31 *Dropped out or removed prior to analysis (N)*: 61 *Numbers analysed at final applicable time point (N)*: 33 *Number of sessions, M (SD)*: 1 (0) *Treatment completion*: 79 (84%) Usual care *Participants (at randomisation)*: 85 *Gender*: 100% female participants *Age*: not given *Ethnicity*: not given *Disability*: not given *Time since trauma, M*: < 72 h *Income*: not given *Education*: not given *Employment*: not given *Sexual violence in adulthood*: 100% *Comorbid conditions*: not given *Baseline PTSD, M (SD)*: 27.76 (14.56) *Partnered*: not given *Randomised (N)*: 85 *Completed post‐treatment assessment (N)*: 38 *Dropped out or removed prior to analysis (N)*: 44 *Numbers analysed at final applicable time point (N)*: 41 Overall *Participants (those receiving the intervention)*: 164 *Gender*: 100% female participants *Age, M (SD)*: 28.79 years (10.47 years) *Ethnicity*: 61.5% white, 15.4% Black, 14.7% Native American, 5.1% Hispanic, 3.2% other *Disability*: not given *Time since trauma, M*: < 72 h *Income*: not given *Education*: not given *Employment*: not given *Sexual violence in adulthood*: 100% *Comorbid conditions*: not given *Baseline PTSD*: not given *Partnered*: not given *Approached (N)*: not given *Ineligible (N)*: not given *Declined (N)*: not given *Other (N)*: not given *Randomised (N)*: 179 *Completed post‐treatment assessment (N)*: 69 *Dropped out or removed prior to analysis (N)*: 105 *Numbers analysed at final applicable time point (N)*: 74 **Inclusion criteria** Women participating in a sexual assault examination at the hospital within 72 h of sexual victimisation English‐speaking 18 years and over **Exclusion criteria** Unable to provide consent because of intoxication, loss of consciousness, apparent psychosis or other reasons preventing them from providing consent (e.g. ventilator dependent, developmental delays) **Pretreatment:** more individuals in standard care (usual care) reported rape (67.1%) compared to the video intervention condition (46.8%).
Interventions	**Intervention characteristics** Other psychologically oriented interventions (psychoeducation and coping video) *Planned number of intervention sessions*: 1 *Mode of delivery (face‐to‐face, online, video, telephone, blend, with explanation)*: video, 9‐minute psychoeducational video for coping with sexual assault *Format (group, individual, blend)*: individual *Therapist qualifications and training appropriate*: N/A *Research allegiance or conflict of interest (yes, no, unclear, with explanation)*: unclear, some authors appear to have developed the video intervention. *Treatment fidelity (yes, no, unclear, with explanation)*: yes, because it was a standard 9‐minute video *Intervention aim and theoretical basis*: video focused on instruction of empirically effective coping strategies as well as modelling of such strategies. Provided a rationale and instructions for self‐directed in vivo exposure to sexual assault‐related cues. Showed methods of using repeated exposure to reduce anxiety in typical assault‐related cue situations *Duration of intervention*: one‐off video Usual care *Planned number of intervention sessions*: 1 *Mode of delivery (face‐to‐face, online, video, telephone, blend, with explanation)*: face‐to‐face *Format (group, individual, blend)*: individual *Therapist qualifications and training appropriate (yes, no, unclear, with explanation)*: yes, rape crisis advocate *Research allegiance or conflict of interest (yes, no, unclear, with explanation)*: no, this was standard care offered at the hospital by the forensic nurse. *Treatment fidelity*: N/A *Intervention aim and theoretical basis*: the advocate provided information about what would happen during the examination and about services available in the community. The SANE nurse on duty provided verbal information about the examination to the survivor and performed a forensic medicolegal examination. *Duration of intervention*: 1 visit
Outcomes	**PTSD** Outcome type: continuous outcome Scale used: PSS‐SR Direction: lower is better Score range: 0 to 119 Data value: endpoint, 3 months **Treatment dropout (intervention only)** Outcome type: dichotomous outcome **Anxiety** Outcome type: continuous outcome Scale used: STAI Direction: lower is better Score range: 20 to 80 Data value: endpoint, 3 months
Identification	**Sponsorship source:** Oklahoma Center for Advancement of Science and Technology HR08‐017 **Country:** USA **Setting:** SANE programme at 1 hospital **Authors name:** Katherine Miller **Institution:** University of Tulsa **Email:** katherine‐miller@utulsa.edu **Year:** 2015
Notes	Total N for population characteristics excludes 15 people in the video intervention arm who did not view the video. Included in meta‐analysis Communicated with author(s) for purpose of the review including additional data

**Table:** Nixon 2016

***Study characteristics***	
Methods	**Study design:** randomised controlled trial **Study grouping:** parallel group **Duration of study:** June 2008 to April 2011 **Ethics approvals:** relevant hospital ethics committee **Location of study:** Adelaide, Australia **Randomisation method:** not given
Participants	**Baseline characteristics** CBT (CPT) *Participants (at randomisation)*: 25 *Gender*: 96% female participants, 4% (1) male participants *Age, M (SD)*: 32.46 years (11.43 years) *Ethnicity*: 83% white, 17% other *Disability*: not given *Time since trauma, M*: < 1 month *Income*: 26% < AUD 10,000 *Education (% high school, college or apprentice, university or years of education)*: 13.48 years (2.87 years) *Employment*: not given *Sexual violence in adulthood*: 100% *Comorbid conditions*: 52% mood, 44% anxiety, 26% substance *Baseline PTSD, M (SD)*: 70.52 (25.51) *Partnered*: not given *Randomised (N)*: 25 *Completed post‐treatment assessment (N)*: 15 *Dropped out or removed prior to analysis (N)*: 1 *Numbers analysed at final applicable time point (N)*: 24 *Number of sessions*: not given *Treatment completion*: 9 (37.5%) Integrative *Participants (at randomisation)*: 22 *Gender*: 100% female participants *Age, M (SD)*: 29.95 years (8.48 years) *Ethnicity*: 91% white, 9% other *Disability*: not given *Time since trauma, M*: < 1 month *Income:* 18% < AUD 10,000 *Education (% high school, college or apprentice, university or years of education)*: 13.91 years (2.74 years) *Employment*: not given *Sexual violence in adulthood*: 100% *Comorbid conditions*: 73% mood, 64% anxiety, 32% substance *Baseline PTSD, M (SD)*: 79.77 (21.21) *Partnered*: not given *Randomised (N)*: 22 *Completed post‐treatment assessment (N)*: 17 *Dropped out or removed prior to analysis (N)*: 0 *Numbers analysed at final applicable time point (N)*: 22 *Number of sessions*: not given *Treatment completion*: N/A Overall *Participants (at randomisation)*: 47 *Gender*: 98% female participants, 2% (1) male participants *Age*: not given *Ethnicity*: 87%% white, 13% other *Disability*: not given *Time since trauma, M*: < 1 month *Income*: 22% < AUD 10,000 *Education*: not given *Employment*: not given *Sexual violence in adulthood*: 100% *Comorbid conditions*: 60% mood, 52% anxiety, 28% substance *Baseline PTSD*: not given *Partnered*: not given *Approached (N)*: 158 *Ineligible (N)*: 79 *Declined (N)*: 10 *Other (N)*: 22 *Randomised (N)*: 47 *Completed post‐treatment assessment (N)*: 32 *Dropped out or removed prior to analysis (N)*: 1 *Numbers analysed at final applicable time point (N)*: 46 *Number of sessions*: N/A *Treatment completion*: N/A **Inclusion criteria** 18 years or older Experienced rape or sexual assault in the past month Able to attend face‐to‐face counselling Met criteria for acute stress disorder Stable for at least prior 4‐week period if taking psychotropic medication **Exclusion criteria** Uncontrolled psychosis Current substance dependence requiring immediate attention Insufficient English Significant cognitive impairment or disability Significant suicide risk Ongoing traumatisation (e.g. being stalked) **Pretreatment:** clinically negligible
Interventions	**Intervention characteristics** CBT (CPT) *Planned number of intervention sessions*: 6 *Mode of delivery (face‐to‐face, online, video, telephone, blend, with explanation)*: face‐to‐face *Format (group, individual, blend)*: individual *Therapist qualifications and training appropriate (yes, no, unclear, with explanation)*: yes, all had at least a bachelor's degree in social work and significant experience of working with assault survivors, and all were trained in delivering the 6‐session CPT by the lead author. *Research allegiance or conflict of interest (yes, no, unclear, with explanation)*: yes, the lead author developed the adapted version of Resick's CPT and ran the study. *Treatment fidelity (yes, no, unclear, with explanation)*: yes, therapy was audiotaped and assessed for adherence to CPT components and competency (rated as 'good'). *Intervention aim and theoretical basis*: this is a modified and abbreviated version of Resick's 12‐session CPT. Initial sessions introduced CR techniques; later sessions included advanced worksheets and alternative ways of thinking. Writing an account of the traumatic event happened in Session 2. Processing the traumatic event happened throughout therapy. *Duration of intervention*: 6 weeks Integrative *Planned number of intervention sessions*: 6 *Mode of delivery (face‐to‐face, online, video, telephone, blend, with explanation)*: face‐to‐face *Format (group, individual, blend)*: individual *Therapist qualifications and training appropriate*: N/A *Research allegiance or conflict of interest*: N/A *Treatment fidelity*: N/A *Intervention aim and theoretical basis*: TAU included many different types of support and may have included some CBT techniques at times. *Duration of intervention*: 6 weeks
Outcomes	**PTSD** Outcome type: continuous outcome Scale used: CAPS Direction: lower is better Score range: 0 to 80 Data value: endpoint, 3 months, 6 months and 12 months **Adverse events** Outcome type: adverse event Notes: worsening symptoms and no other adverse events **Treatment dropout** Outcome type: dichotomous outcome **PTSD** Outcome type: continuous outcome Scale used: PCL‐S Direction: lower is better Score range: 0 to 80 Data value: endpoint, 3 months, 6 months and 12 months **PTSD** Outcome type: continuous outcome Scale used: PTCI Direction: lower is better Score range: 33 to 231 Data value: endpoint, 3 months, 6 months and 12 months **Depression** Outcome type: continuous outcome Scale used: BDI‐II Direction: lower is better Score range: 0 to 63 Data value: endpoint, 3 months, 6 months and 12 months
Identification	**Sponsorship source:** Australian Rotary Health Research Fund grant awarded to RDV Nixon **Country:** Australia **Setting:** sexual assault services **Authors name:** Reg Nixon **Institution:** Flinders University **Email:** reg.nixon@flinders.edu.au **Year:** 2017
Notes	Review team extracted imputed data for our analyses. Included in meta‐analysis

**Table:** Rajan 2020

***Study characteristics***	
Methods	**Study design:** randomised controlled trial **Study grouping:** parallel group **Duration of study:** enrolment was between 26 April 2016 and 17 June 2019 **Ethics approvals:** the authors assert that all procedures contributing to this work comply with the ethical standards of the relevant national and institutional committees on human experimentation and with the Helsinki Declaration of 1975, as revised in 2008. All procedures involving human participants were approved by the regional ethical review board in Stockholm (2015/1868‐31/2). **Location of study:** Stockholm, Sweden **Randomisation method:** after inclusion, participants were randomised to intervention or wait‐list. Preparation of the trial material and randomisation was conducted by the independent data and safety monitoring board KTA via sequenced computer‐generated simple randomisation with 1:1 allocation. Participants received sequentially numbered trial materials with concealed allocation. Allocation envelopes were kept and handled by the trial staff. Due to initial misunderstandings, the prepared sealed envelopes were initially not always picked in strict numeric order. However, the study was monitored and periodically reviewed by the KTA, and the misunderstanding was corrected.
Participants	**Baseline characteristics** Behavioural (MLI) *Participants (at randomisation)*: 21 *Gender*: not given *Age, M (SD)*: 24.2 years (6.3 years) *Ethnicity*: not given *Disability*: not given *Time since trauma, M (SD)*: 31.7 months (16.4 months) *Income:* not given *Education*: not given *Employment*: not given *Sexual violence in adulthood*: not given *Comorbid conditions*: not given *Baseline PTSD, M (SD)*: 58.0 (13.7) *Partnered*: not given *Randomised (N)*: 21 *Completed post‐treatment assessment (N)*: 19 *Dropped out or removed prior to analysis (N)*: 2 *Numbers analysed at final applicable time point (N)*: 19 *Number of sessions*: N/A *Treatment completion*: 20 (95%) Wait‐list *Participants (select at randomisation if available)*: 17 *Gender*: not given *Age, M (SD)*: 24.2 years (6.3 years) *Ethnicity*: not given *Disability*: not given *Time since trauma, M (SD)*: 23.6 months (17.6 months) *Income*: not given *Education*: not given *Employment*: not given *Sexual violence in adulthood*: not given *Comorbid conditions*: not given *Baseline PTSD, M (SD)*: 55.2 (14.1) *Partnered*: not given *Randomised (N)*: 17 *Completed post‐treatment assessment (N)*: 17 *Dropped out or removed prior to analysis (N)*: 0 *Numbers analysed at final applicable time point (N)*: 17 *Number of sessions*: N/A *Treatment completion*: N/A Overall *Participants (select at randomisation if available)*: 38 *Gender*: 100% female participants *Age, M (SD)*: 24 years (6 years) *Ethnicity*: not given *Disability*: not given *Time since trauma*: not given *Income:* not given *Education*: not given *Employment*: not given *Sexual violence in adulthood*: not given *Comorbid conditions*: not given *Baseline PTSD*: not given *Partnered*: not given *Approached (N)*: 135 *Ineligible (N)*: 87 *Declined (N)*: 10 *Other (N)*: 0 *Randomised (N)*: 38 *Completed post‐treatment assessment (N)*: 36 *Dropped out or removed prior to analysis (N)*: 2 *Numbers analysed at final applicable time point (N)*: 36 *Number of sessions*: N/A *Treatment completion*: N/A **Inclusion criteria** Any gender Aged 15 years and above 1 single sexual assault 0 years to 5 years prior to inclusion **Exclusion criteria** Poor understanding of Swedish Multiple traumas Active substance abuse Active psychosis ADHD or autism spectrum disorder as self‐reported or assessed during the doctor’s visit **Pretreatment:** none reported
Interventions	**Intervention characteristics** Behavioural (MLI) *Planned number of intervention sessions*: 1 × 90‐minute to 140‐minute plus preparation *Mode of delivery (face‐to‐face, online, video, telephone, blend, with explanation)*: face‐to‐face *Format (group, individual, blend)*: individual *Therapist qualifications and training appropriate (yes, no, unclear, with explanation)*: yes, likely although we are only told that "the clinic is run by the nongovernmental organization WONSA. All staff at the clinic are specially trained in trauma sensitive care and treating sexually abused patients." *Research allegiance or conflict of interest (yes, no, unclear, with explanation)*: yes, the modified version of LI was developed at the WONSA clinic. *Treatment fidelity (yes, no, unclear, with explanation)*: unclear *Intervention aim and theoretical basis*: the aim of this study was to test the hypothesis that 1 session of MLI could reduce symptoms of PTSD. In LI, the core of the PTSD symptoms is seen to be due to a failure of the index trauma to anchor as an episodic memory in the traumatised individual’s chronologic autobiographic memory. Subsequently, as the index trauma is transformed into an episodic memory anchored in a chronological timeline, the limbic system stops perceiving the index trauma as a potential threat in present time, intrusion and hypervigilance stop, avoidance is no longer needed, and the cardinal symptoms of PTSD decline. *Duration of intervention*: 1 week Wait‐list *Planned number of intervention sessions*: N/A *Mode of delivery*: N/A *Format (group, individual, blend)*: individual *Therapist qualifications and training appropriate*: N/A *Research allegiance or conflict of interest*: N/A *Treatment fidelity*: N/A *Intervention aim and theoretical basis*: participants randomised to the comparison arm received an attention control in the form of the same doctor visit as the intervention arm at which they were clinically assessed, given information about the treatment and asked to prepare an 'MC list' – memory cues through a chronological timeline for each year since the index trauma up to the present time – 1 d or 2 d before the offered intervention, after the second measurement at time point 2. *Duration of intervention*: N/A
Outcomes	**Global mental health** Outcome type: continuous outcome Scale used: General Health Questionnaire 12 Direction: lower is better Score range: 0 to 36 Data value: endpoint **PTSD** Outcome type: continuous outcome Scale used: IES‐R total Direction: lower is better Score range: 0 to 4 Data value: endpoint **Adverse events** Outcome type: adverse event **Treatment dropout (intervention only)** Outcome type: dichotomous outcome
Identification	**Sponsorship source:** the present study was partly funded by the Stockholm Region (ALF, Stockholm County Council) and Magnus Bergvalls Stiftelse **Country:** Sweden **Setting:** specialist clinic for sexually traumatised individuals **Authors name:** Gita Rajan **Institution:** Karolinska Institutet, Huddinge, Sweden **Email:** Gita.rajan@wonsa.org **Year:** 2020
Notes	Study protocol is available at www.WONSA.org. Registration number NCT03141047 at ClinicalTrials.gov (https://register.clinicaltrials.gov/) Communicated with author(s) for purposes of the review Included in meta‐analysis

**Table:** Resick 2002

***Study characteristics***	
Methods	**Study design:** randomised controlled trial **Study grouping:** parallel group **Duration of study:** dates not reported; 11 months including 6‐week intervention with follow‐up at 9 months; longer follow‐up reported in linked papers of 5 years to 10 years **Ethics approvals:** not given **Location of study:** Missouri, USA **Randomisation method:** not given
Participants	**Baseline characteristics** CBT (CPT) *Participants*: 62 (at randomisation) *Gender*: 100% female participants *Age*: not given *Ethnicity*: not given *Disability*: not given *Time since trauma*: not given *Income*: not given *Education*: not given *Employment*: not given *Sexual violence in adulthood*: not given *Comorbid conditions*: not given *Baseline PTSD, M (SD)*: 74.76 (18.77) *Partnered*: not given *Randomised (N)*: 62 *Completed post‐treatment assessment (N)*: 41 *Dropped out or removed prior to analysis (N)*: 36 *Numbers analysed at final applicable time point (N)*: 26 *Number of sessions*: not given *Treatment completion*: 41 (66.12%) CBT (PE) *Participants*: 62 (at randomisation) *Gender*: 100% female participants *Age*: not given *Ethnicity*: not given *Disability*: not given *Time since trauma*: not given *Income*: not given *Education*: not given *Employment*: not given *Sexual violence in adulthood*: not given *Comorbid conditions*: not given *Baseline PTSD, M (SD)*: 76.60 (19.72) *Partnered*: not given *Randomised (N)*: 62 *Completed post‐treatment assessment (N)*: 40 *Dropped out or removed prior to analysis (N)*: 36 *Numbers analysed at final applicable time point (N)*: 26 *Number of sessions*: not given *Treatment completion*: 40 (64.5%) Wait‐list *Participants*: 47 (at randomisation) *Gender*: 100% female participants *Age*: not given *Ethnicity*: not given *Disability*: not given *Time since trauma*: not given *Income*: not given *Education*: not given *Employment*: not given *Sexual violence in adulthood*: not given *Comorbid conditions*: not given *Baseline PTSD, M (SD)*: 69.85 (19.57) *Partnered*: N/A *Randomised (N)*: 47 *Completed post‐treatment assessment (N)*: 40 *Dropped out or removed prior to analysis (N)*: 47 *Numbers analysed at final applicable time point (N)*: 0 Overall *Participants*: 171 (at randomisation) *Gender*: 100% female participants *Age, M (SD)*: 32 years (9.9 years) *Ethnicity*: 71% white, 25% Black, 4% other *Disability*: not given *Time since trauma, M (SD)*: 102 months (102 months), range 3 months to 33 years *Income*: not given *Education (% high school, college or apprentice, university or years of education)*: mean 14.3 years (SD=2.6) *Employment*: not given *Sexual violence in adulthood*: 48% had at least 1 additional rape; 41% reported child sexual abuse; 13.6% reported serious physical assaults; 35.6% reported attempted rape *Comorbid conditions*: 30.7% taking psychotropic medication *Baseline PTSD*: not given *Partnered*: 24.3% *Approached (N)*: 267 *Ineligible (N)*: 84 *Declined (N)*: 0 *Other (N)*: 12 *Randomised (N)*: 171 *Completed post‐treatment assessment (N)*: 121 *Dropped out or removed prior to analysis (N)*: 119 *Numbers analysed at final applicable time point (N)*: 52 **Inclusion criteria** Individuals who have experienced an incident of rape in childhood or adulthood At least 3 months post‐trauma (no upper limit) Stabilised, if taking medication Women with a history of substance dependence off the substance(s) for 6 months Those with substance abuse histories were permitted to participate if they agreed and were deemed able to desist from usage during the period of treatment. Those with a history of incest were not excluded as long as there was another index rape that met the primary criterion for PTSD. **Exclusion criteria** Current psychosis Developmental disabilities Suicidal intent Current parasuicidal behaviour Current dependence on drugs or alcohol Illiteracy Being in a current abusive relationship or being stalked In cases of marital rape, the individuals were in the relationship not less than 6 months ago. **Pretreatment:** none reported
Interventions	**Intervention characteristics** CBT (CPT) *Planned number of intervention sessions*: 9 (1 × 60 min, 8 × 90 min) *Mode of delivery (face‐to‐face, online, video, telephone, blend, with explanation)*: face‐to‐face *Format (group, individual, blend)*: individual *Therapist qualifications and training appropriate (yes, no, unclear, with explanation)*: yes, therapists were 8 women with doctorates in clinical or counselling psychology and a background in CBT. Assignments were balanced so that each therapist handled an approximately equal number of therapy cases in each condition. After the therapists had read the manuals, there was a 2‐day workshop for each therapy. They were trained in CPT by PA Resick and in PE by E Foa. The therapists watched training tapes of the therapy being conducted and then conducted therapy on 2 clients in each of the conditions as pilot participants. Throughout the study, all of the sessions were videotaped, and therapy was closely supervised by the PI, with weekly peer‐supervision sessions to ensure competence and adherence to the protocols. *Research allegiance or conflict of interest (yes, no, unclear, with explanation)*: yes, CPT followed the manual written by Resick 1993; Resick is the lead author of the present study in which CPT is being trialled. However, independent raters who were not otherwise involved in the project conducted assessments of treatment adherence and therapist competence. *Treatment fidelity (yes, no, unclear, with explanation)*: yes, independent raters conducted assessments of treatment adherence and therapist competence. All therapy sessions were videotaped and were available at random for rating. Ratings were made with rating forms developed for this project. In the CPT protocol, there were 5 to 8 unique and essential items for each session (69 items in total). For adherence, the element was checked if it occurred; for competence, a rating was made on a 7‐point scale (poor to excellent, with satisfactory at the midpoint). The 2 protocols had only 1 item in common, an overall rating of the therapist’s skill across the sessions for a given client; ratings ranged from 1 (poor) to 7 (excellent). Adherence and competence ratings were made for 23 CPT clients, and for each, 3 randomly chosen sessions were rated. One of every 3 rated sets of tapes was sent to a second rater for a reliability check. Regarding adherence, all unique and essential elements were included in all sessions, and there were no violations of the protocols in which proscribed elements were introduced in therapy. *Intervention aim and theoretical basis*: CPT followed the format described by Resick 1993 with only a few minor modifications. Session 1 begins with education about PTSD, an overview of treatment, and an assignment to write an impact statement about the personal meaning of the event. After reading and discussing this in Session 2, clients are introduced to the identification of and relationship among events, thoughts, and emotions. At the end of Session 3, clients are given the assignment of writing a detailed account of the trauma, including sensory details, thoughts, and emotions. They are encouraged to experience their emotions as they write their account and read it back to themselves. This account is read to the therapist in Session 4, and cognitive therapy begins with Socratic questions regarding self‐blame and other distortions. The account is written and processed a second time in Session 5. The focus of the therapy shifts to teaching clients to challenge and change their beliefs about the meaning of the event and the implications of the trauma for their lives. From that point, beginning with Session 7, clients use worksheets that incorporate the earlier ones and are asked to develop and practice alternative, more balanced self‐statements. From Sessions 7 to 12, clients are asked to focus on 1 theme each week (safety, trust, power‐control, esteem or intimacy) and correct any overgeneralised beliefs related to that theme. At the 11th session, clients are also asked to rewrite their impact statements to reflect their current beliefs. CPT group averaged 22.6 h of homework. *Duration of intervention*: 6 weeks CBT (PE) *Planned number of intervention sessions*: 9 (1 × 60 min, 8 × 90 min) *Mode of delivery (face‐to‐face, online, video, telephone, blend, with explanation)*: face‐to‐face *Format (group, individual, blend)*: individual *Therapist qualifications and training appropriate (yes, no, unclear, with explanation)*: as previous *Research allegiance or conflict of interest (yes, no, unclear, with explanation)*: no *Treatment fidelity (yes, no, unclear, with explanation)*: yes, independent raters conducted assessments of treatment adherence and therapist competence. In the PE protocol, there were 8 to 15 unique and essential elements for each session (85 items in total). Adherence and competence ratings were made for 21 PE clients, and for each, 3 randomly chosen sessions were rated. One of every 3 rated sets of tapes was sent to a second rater for a reliability check. Regarding adherence, all unique and essential elements were included in all sessions, and there were no violations of the protocols in which proscribed elements were introduced in therapy. *Intervention aim and theoretical basis*: the PE manual was similar to Foa 1998. PE included 4 components: education‐rationale, breathing retraining, behavioural exposures, and imaginal exposures. During the first session, clients are educated about the symptoms of PTSD, and the therapist provides a rationale for in vivo and imaginal exposure in the context of avoidance reduction and habituation of conditioned negative emotional responses. Clients are also introduced to breathing retraining. In Session 2, rationale and education continue, subjective units of distress ratings are introduced, the therapist and client generate an in vivo exposure hierarchy, and the first in vivo exposure assignment is given. Sessions 3 to 9 begin by reviewing homework assignments, conducting imaginal exposure for 45 min to 60 min of the 90‐minute session, and processing the exposure experience with nondirective statements (e.g. education about trauma reactions, paraphrasing). Clients are instructed to listen to the tape of the imaginal exposure sessions each day and to engage in behavioural exposures with increasing difficulty for at least 45 min per day. PE group averaged 44.8 h of homework. *Duration of intervention*: 6 weeks Wait‐list *Planned number of intervention sessions*: N/A *Mode of delivery*: N/A *Format*: N/A *Therapist qualifications and training appropriate*: N/A *Research allegiance or conflict of interest*: N/A *Treatment fidelity*: N/A *Intervention aim and theoretical basis*: women who were assigned to this condition were told that therapy would be provided in 6 weeks and that an interviewer would call them every 2 weeks to ensure that they did not need emergency services. They were also encouraged to call if they wished to talk to a therapist who could provide client‐centred telephone counselling. *Duration of intervention*: N/A
Outcomes	**PTSD** Outcome type: continuous outcome Scale used: CAPS Direction: lower is better Score range: 0 to 80 Data value: endpoint and 12 months **Depression** Outcome type: continuous outcome Scale used: BDI Direction: lower is better Score range: 0 to 63 Data value: endpoint and 12 months **PTSD** Outcome type: continuous outcome Scale used: PSS‐SR Direction: lower is better Score range: 0 to 119 Data value: endpoint and 12 months **Trauma‐related beliefs** Outcome type: continuous outcome Scale used: TRGI global guilt Direction: lower is better Score range: 0 to 4 Data value: endpoint and 12 months **Treatment dropout** Outcome type: dichotomous outcome
Identification	**Sponsorship source:** this work was supported by grant NIH‐1 R01‐MH51509 from the NIMH. **Country:** USA **Setting:** academic/clinical intervention setting; recruitment setting not reported **Authors name:** Patricia Resick **Institution:** Center for Trauma Recovery, Department of Psychology, University of Missouri‐St. Louis **Email:** resick@umsl.edu **Year:** 2002
Notes	Data included are imputed rather than completer; 9‐month data are assigned to our 1‐year time point; 5‐year to 10‐year data are reported by Wachen 2014. Included in meta‐analysis

**Table:** Resick 2008a

***Study characteristics***	
Methods	**Study design:** randomised controlled trial **Study grouping:** parallel group **Duration of study:** October 2000 to August 2005 (data collection) **Ethics approvals:** the study was conducted in compliance with the University of Missouri IRB **Location of study:** St. Louis, Missouri, USA, metropolitan area **Randomisation method:** not given
Participants	**Baseline characteristics** CBT (CPT) *Participants (at randomisation)*: 56 *Gender*: 100% female participants *Age*: not given *Ethnicity*: not given *Disability*: not given *Time since trauma*: not given *Income*: 79% < USD 20,000 *Education*: not given *Employment*: not given *Sexual violence in adulthood*: not given *Comorbid conditions*: not given *Baseline PTSD, M (SD)*: 70.19 (15.5) *Partnered*: not given *Randomised (N)*: 56 *Completed post‐treatment assessment (N)*: 43 *Dropped out or removed prior to analysis (N)*: 11 *Numbers analysed at final applicable time point (N)*: 45 *Number of sessions*: not given *Treatment completion*: 27 (48%) CBT (cognitive therapy) *Participants (select at randomisation if available)*: 51 *Gender*: 100% female participants *Age*: not given *Ethnicity*: not given *Disability*: not given *Time since trauma*: not given *Income*: 46% < USD 20,000 *Education*: not given *Employment*: not given *Sexual violence in adulthood*: not given *Comorbid conditions*: not given *Baseline PTSD, M (SD)*: 73.87 (21.04) *Partnered*: not given *Randomised (N)*: 51 *Completed post‐treatment assessment (N)*: 38 *Dropped out or removed prior to analysis (N)*: 15 *Numbers analysed at final applicable time point (N)*: 36 *Number of sessions*: not given *Treatment completion*: 29 (57%) CBT (written exposure) *Participants (at randomisation)*: 55 *Gender*: 100% female participants *Age*: not given *Ethnicity*: not given *Disability*: not given *Time since trauma*: not given *Income*: 42% < USD 20,000 *Education*: not given *Employment*: not given *Sexual violence in adulthood*: not given *Comorbid conditions*: not given *Baseline PTSD, M (SD)*: 70.38 (18.65) *Partnered*: not given *Randomised (N)*: 55 *Completed post‐treatment assessment (N)*: 38 *Dropped out or removed prior to analysis (N)*: 15 *Numbers analysed at final applicable time point (N)*: 40 *Number of sessions*: not given *Treatment completion*: 30 (54%) Overall *Participants (at randomisation)*: 162 *Gender*: 100% female participants *Age, M (SD)*: 35.4 years (12.4 years) *Ethnicity*: 62% white, 34% African American, 4% other *Disability*: not given *Time since trauma, M (SD)*: 175 months (174 months) *Income*: not given *Education (% high school, college or apprentice, university or years of education)*: 13.8 years (2.8 years), 52% partial college or technical training *Employment*: not given *Sexual violence in adulthood*: 80.7% sexual violence in adulthood; 78% child sexual abuse; 60.7% domestic abuse; 84% adult physical assault *Comorbid conditions*: 50% current major depression; 20% panic disorder *Baseline PTSD*: not given *Partnered*: 20 *Approached (N)*: 256 *Ineligible (N)*: 75 *Declined (N)*: 0 *Other (N)*: 19 *Randomised (N)*: 162 *Completed post‐treatment assessment (N)*: 119 *Dropped out or removed prior to analysis (N)*: 41 *Numbers analysed at final applicable time point (N)*: 121 **Inclusion criteria** Experienced sexual or physical assault in childhood or adulthood and met criteria for PTSD at the time of initial assessment At least 3 months post‐trauma (no upper limit) If taking medication, is stabilised Had been abstinent from substance use for 6 months, if had substance dependence Current users had to agree to desist from substance use during treatment. **Exclusion criteria** Illiteracy Current psychosis, suicidal intent, dependence on drugs or alcohol **Pretreatment:** CPT group had a significantly lower income than other 2 groups (cognitive therapy and written exposure/accounts). Lowest‐income participants were least likely to complete the full course of therapy.
Interventions	**Intervention characteristics** CBT (CPT) *Planned number of intervention sessions*: 12 (12 × 60‐minute sessions) *Mode of delivery (face‐to‐face, online, video, telephone, blend, with explanation)*: face‐to‐face *Format (group, individual, blend)*: individual *Therapist qualifications and training appropriate (yes, no, unclear, with explanation)*: yes, therapists across arms were 8 women with either a doctorate or master's degree in clinical psychology and training in CBT. They were trained by Resick or another senior team member in the therapy required here; they had a manual, watched training videos and had practice sessions. They were supervised during the trial at weekly sessions. *Research allegiance or conflict of interest (yes, no, unclear, with explanation)*: yes, first author developed the therapy. *Treatment fidelity (yes, no, unclear, with explanation)*: yes, trained independent raters conducted assessment of treatment adherence and therapist competence. All sessions were recorded and available for random selection for assessment of fidelity. Ninety per cent of unique and essential elements were included in all sessions, and there were no violations of the protocols regarding proscribed elements. *Intervention aim and theoretical basis*: CPT followed the manual as written by Resick 1993. CPT is a highly structured protocol in which the client learns the skill of recognising and challenging dysfunctional cognitions, first about the worst traumatic event and then later with regard to the meaning of the events for current beliefs about self and others. *Duration of intervention*: 6 weeks CBT (cognitive therapy) *Planned number of intervention sessions*: 12 (12 × 60‐minute sessions) *Mode of delivery (face‐to‐face, online, video, telephone, blend, with explanation)*: face‐to‐face *Format (group, individual, blend)*: individual *Therapist qualifications and training appropriate (yes, no, unclear, with explanation)*: as previous *Research allegiance or conflict of interest (yes, no, unclear, with explanation)*: as previous *Treatment fidelity (yes, no, unclear, with explanation)*: as previous, and adherence for unique and essential elements was 90% adherence to the protocol across all sessions, and there were no protocol violations on proscribed elements. *Intervention aim and theoretical basis*: identical to CPT but without inclusion of the written account component *Duration of intervention*: 6 weeks CBT (written exposure) *Planned number of intervention sessions*: 7 (2 × 60‐minute sessions; 5 × 120 min) *Mode of delivery (face‐to‐face, online, video, telephone, blend, with explanation)*: face‐to‐face *Format (group, individual, blend)*: individual *Therapist qualifications and training appropriate (yes, no, unclear, with explanation)*: as previous *Research allegiance or conflict of interest (yes, no, unclear, with explanation)*: as previous *Treatment fidelity (yes, no, unclear, with explanation)*: as previous, and for unique and essential elements on written accounts adherence, there was 80% adherence to the protocol across all sessions, and there were 2 violations on proscribed elements. *Intervention aim and theoretical basis*: included only the writing account component, although this has been expanded considerably to fit into a similar amount of therapy time *Duration of intervention*: 6 weeks
Outcomes	**PTSD** Outcome type: continuous outcome Scale used: CAPS Direction: lower is better Score range: 0 to 80 Data value: endpoint and 6 months **Adverse events** Outcome type: adverse event Notes: the adverse event in the written accounts only arm was increased suicide ideation; 1 other adverse event was noted as being unrelated, and they did not report what it was or which group it had occurred in. **Treatment dropout** Outcome type: dichotomous outcome **PTSD** Outcome type: continuous outcome Scale used: PDS – Self‐report Direction: lower is better Score range: 0 to 51 Data value: endpoint and 6 months **Depression** Outcome type: continuous outcome Scale used: BDI‐II Direction: lower is better Score range: 0 to 63 Data value: endpoint and 6 months **Anxiety** Outcome type: continuous outcome Scale used: STAI‐State Direction: lower is better Score range: 0 to 80 Data value: endpoint and 6 months **Trauma‐related beliefs** Outcome type: continuous outcome Scale used: PBRS Direction: higher is better Score range: 0 to 6 Data value: endpoint and 6 months **Guilt** Outcome type: continuous outcome Scale used: TRGI Direction: lower is better Score range: 0 to 4 Data value: endpoint and 6 months **Shame** Outcome type: continuous outcome Scale used: Epworth Sleepiness Scale Direction: lower is better Score range: 25 to 100 Data value: endpoint and 6 months
Identification	**Sponsorship source:** NIMH grant 2‐R01‐MH51509 **Country:** USA **Setting:** community sample identified through victim assistance agencies, community therapists and self‐referral; interventions delivered in academic centre **Authors name:** Patricia Resick **Institution:** Boston University **Email:** Patricia.Resick@va.gov **Year:** 2008
Notes	—

**Table:** Rothbaum 1997

***Study characteristics***	
Methods	**Study design:** randomised controlled trial **Study grouping:** parallel group **Duration of study:** 4 months **Ethics approvals:** not given **Location of study:** Atlanta, Georgia, USA **Randomisation method:** not given
Participants	**Baseline characteristics** Behavioural (EMDR) *Participants (select at randomisation if available)*: 11 *Gender*: 100% female participants *Age, M (SD)*: 31.6 years (9.8 years) *Ethnicity*: not given *Disability*: not given *Time since trauma, M (SD)*: 62.2 months (53.3 months) *Income*: 27% < USD 10,000 *Education (% high school, college or apprentice, university or years of education)*: 50% high school, 50% college *Employment (% employed; unemployed, other)*: 70% employed; 10% unemployed; 20% student *Sexual violence in adulthood*: 100% *Comorbid conditions*: not given *Baseline PTSD, M (SD)*: 33.3 (8.7) *Partnered*: 30% *Randomised (N)*: 11 *Completed post‐treatment assessment (N)*: 10 *Dropped out or removed prior to analysis (N)*: 1 *Numbers analysed at final applicable time point (N)*: 10 *Number of sessions, M*: 4 *Treatment completion*: 10 (91%) Wait‐list *Participants (at randomisation)*: 10 *Gender*: 100% female participants *Age, M (SD)*: 37.5 years (11.1 years) *Ethnicity*: not given *Disability*: not given *Time since trauma, M (SD)*: 155.8 months (106.6 months) *Income:* 10% < USD 10,000 *Education (% high school, college or apprentice, university or years of education)*: 50% high school, 50% college *Employment (% employed, unemployed, other)*: 62.5% employed, 37.5% unemployed *Sexual violence in adulthood*: 100% *Comorbid conditions*: not given *Baseline PTSD, M (SD)*: 39 (8.2) *Partnered*: 25% *Randomised (N)*: 10 *Completed post‐treatment assessment (N)*: 8 *Dropped out or removed prior to analysis (N)*: 2 *Numbers analysed at final applicable time point (N)*: 8 Overall *Participants (at randomisation)*: 21 *Gender*: 100% female participants *Age*: not given *Ethnicity*: not given *Disability*: not given *Time since trauma*: not given *Income*: 22% < USD 10,000 *Education (% high school, college or apprentice, university or years of education)*: 50% high school, 50% college *Employment (% employed; unemployed, other)*: 55.5% employed, 22.2% unemployed; 11.1% student *Sexual violence in adulthood*: 100% *Comorbid conditions*: not given *Baseline PTSD*: not given *Partnered*: 28% *Approached (N)*: not given *Ineligible (N)*: not given *Declined (N)*: not given *Other (N)*: not given *Randomised (N)*: 21 *Completed post‐treatment assessment (N)*: 18 *Dropped out or removed prior to analysis (N)*: 3 *Numbers analysed at final applicable time point (N)*: 18 **Inclusion criteria** Adult females Victim of rape Rape occurred at least 3 months prior to allow for the natural decline in PTSD symptoms Participants met the criteria for DSM‐III‐R PTSD **Exclusion criteria** Alcohol or drug dependence Had used cocaine in last 60 d **Pretreatment:** none reported
Interventions	**Intervention characteristics** Behavioural (EMDR) *Planned number of intervention sessions*: 4 *Mode of delivery (face‐to‐face, online, video, telephone, blend, with explanation)*: face‐to‐face *Format (group, individual, blend)*: individual *Therapist qualifications and training appropriate (yes, no, unclear, with explanation)*: yes, the author was the therapist for all participants in the EMDR condition. She was trained by Francine Shapiro, PhD, at levels I and II. The therapists for the participants receiving EMDR following completion of the wait‐list condition, in addition to the author, included another female psychologist and a female psychiatrist, both also having received training at levels I and II. *Research allegiance or conflict of interest (yes, no, unclear, with explanation)*: yes, the author was the therapist, and the treatment integrity was assessed by those aligned with the therapy developer. *Treatment fidelity (yes, no, unclear, with explanation)*: unclear, videotapes of 1 session for each EMDR participant were randomly selected and reviewed by someone from the therapy developers team, but outcomes of that were not reported. *Intervention aim and theoretical basis*: EMDR is based around the idea that PTSD is caused by unprocessed emotions and memory from the traumatic event. The therapy allows the person to process the trauma and retrains the person's emotional response to stimuli that have become associated with the trauma but that are in fact generally safe, leading to reduced symptoms of PTSD. *Duration of intervention*: 4 weeks Wait‐list *Planned number of intervention sessions*: N/A *Mode of delivery*: N/A *Format*: N/A *Therapist qualifications and training appropriate*: N/A *Research allegiance or conflict of interest*: N/A *Treatment fidelity*: N/A *Intervention aim and theoretical basis*: N/A *Duration of intervention*: N/A
Outcomes	**PTSD** Outcome type: continuous outcome Scale used: PSS‐I Direction: lower is better Score range: 0 to 51 Data value: endpoint and 3 months **Depression** Outcome type: continuous outcome Scale used: BDI Direction: lower is better Score range: 0 to 63 Data value: endpoint and 3 months **PTSD** Outcome type: continuous outcome Scale used: IES‐R Intrusion Direction: lower is better Score range: 0 to 4 Data value: endpoint and 3 months **PTSD** Outcome type: continuous outcome Scale used: IES‐R Avoidance Direction: lower is better Score range: 0 to 4 Data value: endpoint and 3 months **PTSD** Outcome type: continuous outcome Scale used: IES‐R total Direction: lower is better Score range: 0 to 12 Data value: endpoint and 3 months **Treatment dropout** Outcome type: dichotomous outcome **Global mental health (RAST)** Outcome type: continuous outcome Scale used: RAST Direction: lower is better Score range: 0 to 280 Data value: endpoint and 3 months **Anxiety (STAI‐State)** Outcome type: continuous outcome Scale used: STAI‐State Direction: lower is better Score range: 0 to 80 Data value: endpoint and 3 months **Anxiety (STAI‐Trait)** Outcome type: continuous outcome Scale used: STAI‐Trait Direction: lower is better Score range: 20 to 80 Data value: endpoint and 3 months
Identification	**Sponsorship source:** University Research Committee of Emory University **Country:** USA **Setting:** academic setting with community sample **Authors name:** Barbara O Rothbaum **Institution:** Emory University **Email:** brothba@emory.edu **Year:** 1997
Notes	Wait‐list group received intervention after post‐treatment measures, so no 3‐month follow‐up data included for them. Follow‐up data for the wait‐list group are not included at all for PTSD (PSS‐I) as a total score, although the subscale data are provided.

**Table:** Rothbaum 2005

***Study characteristics***	
Methods	**Study design:** randomised controlled trial **Study grouping:** parallel group **Duration of study:** not given **Ethics approvals:** the informed consent form and this study were approved by an IRB (Emory). **Location of study:** Atlanta, Georgia, USA **Randomisation method:** not given
Participants	**Baseline characteristics** Behavioural (EMDR) *Participants (completers)*: 20 *Gender*: 100% female participants *Age*: not given *Ethnicity*: not given *Disability*: not given *Time since trauma, M (SD)*: 145.9 months (146.8 months) *Income*: not given *Education*: not given *Employment*: not given *Sexual violence in adulthood*: not given *Comorbid conditions*: not given *Baseline PTSD, M*: 80 *Partnered*: not given *Randomised (N)*: 25 *Completed post‐treatment assessment (N)*: 20 *Dropped out or removed prior to analysis (N)*: 5 *Numbers analysed at final applicable time point (N)*: 19 *Number of sessions*: not given *Treatment completion*: 20 (80%) CBT (PE) *Participants (completers)*: 20 *Gender*: 100% female participants *Age*: not given *Ethnicity*: not given *Disability*: not given *Time since trauma, M (SD)*: 120.9 months (94.1 months) *Income*: not given *Education*: not given *Employment*: not given *Sexual violence in adulthood*: not given *Comorbid conditions*: not given *Baseline PTSD, M*: 61 *Partnered*: not given *Randomised (N)*: 23 *Completed post‐treatment assessment (N)*: 20 *Dropped out or removed prior to analysis (N)*: 5 *Numbers analysed at final applicable time point (N)*: 18 *Number of sessions*: not given *Treatment completion*: 20 (87%) Wait‐list *Participants (completers)*: 20 *Gender*: 100% female participants *Age*: not given *Ethnicity*: not given *Disability*: not given *Time since trauma, M (SD)*: 162.9 months (136.9 months) *Income*: not given *Education:* not given *Employment*: not given *Sexual violence in adulthood*: not given *Comorbid conditions*: not given *Baseline PTSD, M*: 78 *Partnered*: not given *Randomised (N)*: 24 *Completed post‐treatment assessment (N)*: 20 *Dropped out or removed prior to analysis (N)*: 20 *Numbers analysed at final applicable time point (N)*: 0 Overall *Participants (select at randomisation if available)*: 60 *Gender*: 100% female participants *Age, M (SD)*: 33.8 years (11.0 years) *Ethnicity*: 68% white *Disability*: not given *Time since trauma*: not given *Income*: 30% household income USD 40,000 or higher *Education (% high school, college or apprentice, university or years of education)*: 48% college degree or higher *Employment (% employed; unemployed, other)*: 77% employed or full‐time students *Sexual violence in adulthood*: 100% sexual violence > 12 years; including the index assault, participants experienced a mean of 6.0 traumas (SD = 4.1) prior to study entry *Comorbid conditions*: 65% had 1 or more comorbid mental health diagnosis. *Baseline PTSD*: not given *Partnered*: 26.7% *Approached (N)*: not given *Ineligible (N)*: 1 *Declined (N)*: not given *Other (N)*: 1 *Randomised (N)*: 72 *Completed post‐treatment assessment (N)*: 60 *Dropped out or removed prior to analysis (N)*: 35 *Numbers analysed at final applicable time point (N)*: 37 **Inclusion criteria** A rape or attempted rape in adulthood (age 12 years or older) or a single incident of rape in childhood (age 0 years to 11 years) by either a family or a non‐family member If taking psychotropic medication, participants were required to be stable on the medication at the same dosage for 30 d prior to study entry and to agree not to change medication or dosage for duration of the study. **Exclusion criteria** History of schizophrenia or other psychoses Current suicidal risk or practised self‐mutilation Illiterate and thus unable to complete self‐reports Current alcohol or drug dependence as determined by the Structured Clinical Interview for the DSM‐IV Blind or had a history of serious eye disease (e.g. detached retina) that would cause risk with rapid eye movement Use of cocaine in any form within 60 d of treatment administration In an ongoing threatening situation (e.g. domestic violence) **Pretreatment:** participants in the EMDR condition exhibited significantly higher overall PTSD symptoms; higher levels of intrusive symptoms on the PSS; and higher levels of depression, dissociation and trait anxiety than the PE group.
Interventions	**Intervention characteristics** Behavioural (EMDR) *Planned number of intervention sessions*: 9 (× 90 min) *Mode of delivery (face‐to‐face, online, video, telephone, blend, with explanation)*: face‐to‐face *Format (group, individual, blend)*: individual *Therapist qualifications and training appropriate (yes, no, unclear, with explanation)*: unclear, there was not much information other than that participants were assigned to 1 of 3 doctoral‐level psychologists who were trained in both therapies. *Research allegiance or conflict of interest (yes, no, unclear, with explanation)*: no *Treatment fidelity (yes, no, unclear, with explanation)*: yes, 92.09% of sessions adherent for essential and unique items; therapist skill was rated 6.04 (SD 0.58) *Intervention aim and theoretical basis*: EMDR involves having the patient imagine a scene that represents the worst part of the trauma, focusing on the sensations of distress in her body, and rehearsing negative thoughts that match the picture. The patient simultaneously follows the therapist’s fingers moving back and forth approximately 18 inches in front of her, a minimum of 20 times each repetition. Distress ratings are gathered using a SUDs from 0 to 10. Once the distress about this scene from the memory drops to 0 or 1, the patient is asked to track the therapist’s finger while rehearsing a new, preferred belief, repeating this sequence until the new statement feels true to the patient. Cognitive work is accomplished through the use of cognitive interweaves. *Duration of intervention*: 5 weeks CBT (PE) *Planned number of intervention sessions*: 9 (× 90 min) *Mode of delivery (face‐to‐face, online, video, telephone, blend, with explanation)*: face‐to‐face *Format (group, individual, blend)*: individual *Therapist qualifications and training appropriate (yes, no, unclear, with explanation)*: as previous *Research allegiance or conflict of interest (yes, no, unclear, with explanation)*: unclear *Treatment fidelity (yes, no, unclear, with explanation)*: yes, 90.46% of sessions adherent for essential and unique items; therapist skill was rated 5.80 (SD = 0.66) *Intervention aim and theoretical basis*: PE treatment (Foa 1998; Foa 1991) – a hierarchy of avoided situations is constructed for in vivo exposure homework. The next 7 sessions are devoted to reliving the rape scene in imagination. Participants are instructed to try to imagine the assault scene as vividly as possible and describe it aloud in the present tense. Anxiety levels (SUDs 0 to 100) are monitored every 5 min during exposure. Participants are encouraged to describe the rape in its entirety, repeating it several times for 45 min to 60 min per session. Following exposure, the participant’s reaction to the exposure situation is discussed, and a homework assignment is assigned. Participants' narratives are tape‐recorded, and they are instructed to listen to the tapes at home at least once daily. *Duration of intervention*: 5 weeks Wait‐list *Planned number of intervention sessions*: N/A *Mode of delivery*: N/A *Format*: N/A *Therapist qualifications and training appropriate*: N/A *Research allegiance or conflict of interest*: N/A *Treatment fidelity*: N/A *Intervention aim and theoretical basis*: N/A *Duration of intervention*: N/A
Outcomes	**Depression** Outcome type: continuous outcome Scale used: BDI Direction: lower is better Score range: 0 to 63 Data value: endpoint and 6 months **Treatment dropout** Outcome type: dichotomous outcome **Anxiety** Outcome type: continuous outcome Scale used: STAI‐State Direction: lower is better Score range: 0 to 80 Data value: endpoint and 6 months **Dissociation** Outcome type: continuous outcome Scale used: Dissociative Experiences Scale‐II Direction: lower is better Score range: 0 to 100 Data value: endpoint and 6 months
Identification	**Sponsorship source:** NIMH grant 1 R01 MH56351‐01A1 **Country:** USA **Setting:** women with rape‐related PTSD; interventions delivered in clinical academic setting **Authors name:** Barbara O Rothbaum **Institution:** Emory University **Email:** brothba@emory.edu **Year:** 2005
Notes	No explanation on where sample was derived from. The 60 in the population characteristics is the intervention completer sample. Final time point is 6 months with 18 PE and 19 EMDR participants and no control.

**Table:** Schnurr 2007

***Study characteristics***	
Methods	**Study design:** randomised controlled trial **Study grouping:** parallel group **Duration of study:** August 2002 to October 2005 **Ethics approvals:** IRB at each recruitment site approved the protocol. **Location of study:** Vermont, USA **Randomisation method:** study staff called a computerised voice information system at the study co‐ordinating centre to obtain the treatment assignment for participants. The voice information system first verified entry criteria to ensure accuracy and reduce errors. Verified eligible participants were randomised within each site to PE or PCT using permuted blocks with random block sizes of 4 or 6.
Participants	**Baseline characteristics** CBT (PE) *Participants (at randomisation)*: 141 *Gender*: 100% female participants *Age, M (95% CI)*: 44.6 years (43.1 years to 46.2 years) *Ethnicity*: 56% white, 33.3% Black, Hispanic 5.7%, other 5% *Disability (%)*: 19.9% (based on PTSD disability status approved) *Time since trauma*: not given *Income*: not given *Education (% high school, college or apprentice, university or years of education)*: 91.5% post high‐school education *Employment (% employed; unemployed, other)*: 37.6% unemployed *Sexual violence in adulthood*: 69.5% *Comorbid conditions*: 75.2% *Baseline PTSD, M (95% CI)*: 77.6 (74.8 to 80.4) *Partnered*: 31.9% *Randomised (N)*: 141 *Completed post‐treatment assessment (N)*: 141 *Dropped out or removed prior to analysis (N)*: 21 *Numbers analysed at final applicable time point (N)*: 141 *Number of sessions, M*: 8 *Treatment completion*: 88 (62%) Other psychologically oriented interventions (PCT) *Participants (at randomisation)*: 143 *Gender*: 100% female participants *Age, M (95% CI)*: 44.9 years (43.4 years to 46.5 years) *Ethnicity*: 53.1% white, 32.2% Black, Hispanic 6.3%, other 8.4% *Disability*: 23.8% (based on PTSD disability status approved) *Time since trauma*: not given *Income*: not given *Education (% high school, college or apprentice, university or years of education)*: 86.7% post‐high school education *Employment (% employed, unemployed, other)*: 39.2% unemployed *Sexual violence in adulthood*: 76.2% *Comorbid conditions*: 80.4% *Baseline PTSD, M (95% CI)*: 77.9 (75.1 to 80.6) *Partnered*: 31.5% *Randomised (N)*: 143 *Completed post‐treatment assessment (N)*: 143 *Dropped out or removed prior to analysis (N)*: 17 *Numbers analysed at final applicable time point (N)*: 143 *Number of sessions, M*: 9.3 *Treatment completion*: 113 (79%) Overall *Participants (select at randomisation if available)*: 284 *Gender*: 100% female participants *Age*: not given *Ethnicity*: 55% white, 33% Black, 6% Hispanic, 6% other *Disability*: 21.8% (based on PTSD disability status approved) *Time since trauma*: not given *Income*: not given *Education (% high school, college or apprentice, university or years of education)*: 89% post‐high school *Employment (% employed; unemployed, other)*: 38% unemployed *Sexual violence in adulthood*: 73% *Comorbid conditions*: 78% *Baseline PTSD*: not given *Partnered*: 31% *Approached (N)*: 396 *Ineligible (N)*: 48 *Declined (N)*: 5 *Other (N)*: 59 *Randomised (N)*: 284 *Completed post‐treatment assessment (N)*: 284 *Dropped out or removed prior to analysis (N)*: 38 *Numbers analysed at final applicable time point (N)*: 284 **Inclusion criteria** Adult females Current PTSD – assessed using CAPS – symptom severity of 45 or higher 3 or more months since experiencing trauma Clear memory of the trauma that caused the PTSD Agreement to not receive other psychotherapy for PTSD during study If taking psychoactive medication, stable regimen for last 2 months **Exclusion criteria** Substance dependence not in remission for at least 3 months Current psychotic symptoms, mania or bipolar disorder Prominent current suicidal or homicidal ideation Cognitive impairment Current involvement in a violent relationship Self‐mutilation within the past 6 months **Pretreatment:** none reported
Interventions	**Intervention characteristics** CBT (PE) *Planned number of intervention sessions*: 10 *Mode of delivery (face‐to‐face, online, video, telephone, blend, with explanation)*: face‐to‐face *Format (group, individual, blend)*: individual *Therapist qualifications and training appropriate (yes, no, unclear, with explanation)*: yes, master's‐ or doctorate‐level qualifications among 52 female therapists all receiving bespoke training and supervision of practice *Research allegiance or conflict of interest (yes, no, unclear, with explanation)*: yes, developers of therapy are co‐authors of the study. *Treatment fidelity (yes, no, unclear, with explanation)*: yes, 11.7% of sessions were reviewed by someone independent of therapy delivery and rated as very good to excellent on average on competence and completeness. *Intervention aim and theoretical basis*: PE included education about common reactions to trauma; breathing retraining; prolonged (repeated) recounting (imaginal exposure) of trauma memories during sessions; homework (listening to a recording of the recounting made during the therapy session and repeated in vivo exposure to safe situations the participant avoids because of trauma‐related fear); and discussion of thoughts and feelings related to exposure exercises. Sessions 1 and 2 were introductory and included provision of the treatment rationale and education about PTSD. Imaginal exposure occurred in Sessions 3 through 10. *Duration of intervention*: 10 weeks Other psychologically oriented interventions (PCT) *Planned number of intervention sessions*: 10 *Mode of delivery (face‐to‐face, online, video, telephone, blend, with explanation)*: face‐to‐face *Format (group, individual, blend)*: individual *Therapist qualifications and training appropriate (yes, no, unclear, with explanation)*: yes, as before *Research allegiance or conflict of interest (yes, no, unclear, with explanation)*: yes, developers of therapy are co‐authors of the study. *Treatment fidelity (yes, no, unclear, with explanation)*: yes, as before *Intervention aim and theoretical basis*: instead of focusing on trauma, PCT focuses on current life problems as manifestations of PTSD. The aim of using PCT in this study was to provide a credible therapeutic alternative to control for nonspecific therapeutic factors so that observed effects of PE could be attributed to its specific effects beyond the benefits of good therapy. Treatment followed the same format as PE, although the content differed. Sessions 1 and 2 were introductory and included provision of the treatment rationale and education about PTSD. Sessions 3 through 9 focused on discussing and reviewing general daily difficulties. Session 10 focused on reviewing accomplishments made during therapy and plans for the future. *Duration of intervention*: 10 weeks
Outcomes	**PTSD** Outcome type: continuous outcome Scale used: CAPS Direction: lower is better Score range: 0 to 80 Data value: endpoint, 3 months and 6 months **Depression** Outcome type: continuous outcome Scale used: BDI Direction: lower is better Score range: 0 to 63 Data value: endpoint, 3 months and 6 months **Adverse events** Outcome type: adverse event **Treatment dropout** Outcome type: dichotomous outcome **PTSD** Outcome type: continuous outcome Scale used: PCL‐C Direction: lower is better Score range: 17 to 85 Data value: endpoint, 3 months and 6 months **Anxiety** Outcome type: continuous outcome Scale used: STAI‐State Direction: lower is better Score range: 0 to 80 Data value: endpoint, 3 months and 6 months **Quality of life** Outcome type: continuous outcome Scale used: Quality of Life Inventory Direction: higher is better Score range: −6 to 6 Data value: endpoint, 3 months and 6 months **Global mental health** Outcome type: continuous outcome Scale used: SF‐36 Mental Direction: higher is better Score range: 0 to 100 Data value: endpoint, 3 months and 6 months **Quality of life** Outcome type: continuous outcome Scale used: SF‐36 Physical Direction: higher is better Score range: 0 to 100 Data value: endpoint, 3 months and 6 months **Substance use** Outcome type: continuous outcome Scale used: ASI Alcohol Direction: lower is better Score range: 0 to 9 Data value: endpoint, 3 months and 6 months **Substance use** Outcome type: continuous outcome Scale used: ASI Drug Direction: lower is better Score range: 0 to 9 Data value: endpoint, 3 months and 6 months
Identification	**Sponsorship source:** VA Cooperative Studies Program and the Department of Defense **Country:** USA **Setting:** VA medical centres, counselling centres and a military hospital **Authors name:** Paula Schnurr **Institution:** Dartmouth Medical School **Email:** Paula.Schnurr@Dartmouth.edu **Year:** 2007
Notes	Included in meta‐analysis

**Table:** Sikkema 2018

***Study characteristics***	
Methods	**Study design:** randomised controlled trial **Study grouping:** parallel group **Duration of study:** March 2016 to July 2017 **Ethics approvals:** all procedures performed in studies involving human participants were in accordance with the ethical standards of the institutional or national research committee and with the 1964 Helsinki declaration and its later amendments or comparable ethical standards (Duke C0147, UCT 071/2014). **Location of study:** Cape Town, South Africa **Randomisation method:** Participants were randomly assigned (1:1) to the SoC control condition (SoC: 3 adherence 'readiness' sessions) or the experimental intervention condition (SoC, plus ImpACT) using a small block (size 8 or 10) randomisation procedure. Condition assignments were placed in sealed envelopes, blinded to study staff until assignment. Due to the nature of the intervention conditions, neither participants nor staff could be blinded to condition assignment.
Participants	**Baseline characteristics** Other psychosocial interventions (ImpACT) *Participants (at randomisation)*: 32 *Gender*: 100% female participants *Age, M (SD)*: 29.2 years (6.6 years) *Ethnicity*: not given *Disability*: not given *Time since trauma*: 43.8% in last 3 months *Income*: not given *Education (% high school, college or apprentice, university or years of education)*: 81.3% some high school or less, 15.6% completed high school, 3.1% some university or more *Employment (% employed; unemployed, other)*: 31.2% employed, 6.3% casual or sometimes working, 62.5% unemployed *Sexual violence in adulthood*: 75.0% *Comorbid conditions*: 100% HIV‐infected; 93.8% lifetime physical partner abuse; 46.9% hazardous drinking *Baseline PTSD, M (SD)*: 43.28 (3.88) *Partnered*: 93.7% *Randomised (N)*: 32 *Completed post‐treatment assessment (N)*: 19 *Dropped out or removed prior to analysis (N)*: 9 *Numbers analysed at final applicable time point (N)*: 23 *Number of sessions, M (SD)*: individual sessions, 3.1 (1.3); group sessions, no mean given but attendance was poor *Treatment completion*: 21 (66%) Usual care *Participants (at randomisation)*: 32 *Gender*: 100% female participants *Age, M (SD)*: 29.0 years (8.4 years) *Ethnicity*: not given *Disability*: not given *Time since trauma*: 37.5% in last 3 months *Income*: not given *Education (% high school, college or apprentice, university or years of education)*: 75% some high school or less, 15.6% completed high school, 9.4% some university or more *Employment (% employed, unemployed, other)*: 18.7% employed, 6.3% casual or sometimes working, 75% unemployed *Sexual violence in adulthood*: 78.1% *Comorbid conditions*: 100% HIV infected; 75% lifetime physical partner abuse; 40.6% hazardous drinking *Baseline PTSD, M (SD)*: 30.72 (3.58) *Partnered*: 81.2% *Randomised (N)*: 32 *Completed post‐treatment assessment (N)*: 26 *Dropped out or removed prior to analysis (N)*: 4 *Numbers analysed at final applicable time point (N)*: 28 Overall *Participants (at randomisation)*: 64 *Gender*: 100% female participants *Age, M (SD)*: 29.1 years (7.5 years) *Ethnicity*: not given *Disability*: not given *Time since trauma*: 40.6% in last 3 months *Income*: not given *Education (% high school, college or apprentice, university or years of education)*: 78.1% some high school or less, 15.6% completed high school, 6.3% some university or more *Employment (% employed, unemployed, other)*: 25% employed, 6.3% casual or sometimes working, 68.7% unemployed *Sexual violence in adulthood*: 76.6% *Comorbid conditions*: 100% HIV infected; 84.4% lifetime physical partner abuse; 43.8% hazardous drinking *Baseline PTSD*: not given *Partnered*: 87.5% *Approached (N)*: 141 *Ineligible (N)*: 59 *Declined (N)*: 10 *Other (N)*: 8 *Randomised (N)*: 64 *Completed post‐treatment assessment (N)*: 45 *Dropped out or removed prior to analysis (N)*: 13 *Numbers analysed at final applicable time point (N)*: 51 **Inclusion criteria** Women who were HIV‐infected and met ART initiation criteria History of sexual abuse, defined as sexual abuse or assault that occurred during childhood, adolescence or adulthood, based on the WHO Composite International Diagnostic Interview and the Childhood Trauma Questionnaire 18 years or older Xhosa‐speaking Accessing HIV care services at the study clinic **Exclusion criteria** Women with current suicidal intent were excluded and referred for psychiatric treatment **Pretreatment:** no significant differences were identified between groups on sample characteristics recorded, although there was a difference on the baseline scores for PTSD (PCL‐5).
Interventions	**Intervention characteristics** Other psychosocial interventions (ImpACT) *Planned number of intervention sessions*: 10 *Mode of delivery (face‐to‐face, online, video, telephone, blend, with explanation)*: face‐to‐face *Format (group, individual, blend)*: blend *Therapist qualifications and training appropriate (yes, no, unclear, with explanation)*: unclear, the provider was trained by the study PI and co‐ordinator to facilitate ImpACT using an intervention manual, participant workbook and culturally tailored visual aids. There was individual supervision from a clinical psychologist weekly. *Research allegiance or conflict of interest (yes, no, unclear, with explanation)*: yes, PI developed the intervention. *Treatment fidelity (yes, no, unclear, with explanation)*: yes, quality assurance data collected after each session and checked and reviewed by study co‐ordinator with supervisory meetings. Assessment of fidelity for individual sessions high at 97.8%. Fidelity for group sessions also reported as high *Intervention aim and theoretical basis*: designed to reduce traumatic stress and improve HIV care engagement by developing effective strategies for coping with HIV and trauma and by enhancing adherence to ART (not singularly intended to treat PTSD). It addressed commonalities of stressors in trauma and HIV also included elements of CPT and adaptive coping strategies. *Duration of intervention*: 10 weeks Usual care *Planned number of intervention sessions*: 3 *Mode of delivery (face‐to‐face, online, video, telephone, blend, with explanation)*: face‐to‐face *Format (group, individual, blend)*: individual *Therapist qualifications and training appropriate*: N/A *Research allegiance or conflict of interest*: N/A *Treatment fidelity*: N/A *Intervention aim and theoretical basis*: SoC counselling designed to support adherence to ART for HIV but not to tackle trauma from abuse *Duration of intervention*: 3 weeks
Outcomes	**PTSD** Outcome type: continuous outcome Scale used: PCL‐5 Direction: lower is better Score range: 0 to 80 Data value: endpoint and 3 months **Treatment dropout (intervention only)** Outcome type: dichotomous outcome
Identification	**Sponsorship source:** Duke University, NIMH and University of Cape Town **Country:** South Africa **Setting:** HIV‐infected women initiating ART recruited (and received intervention) at a primary healthcare clinic **Authors name:** Kathleen Sikkema **Institution:** Duke University and University of Cape Town **Email:** kathleen.sikkema@duke.edu **Year:** 2018
Notes	ClinicalTrials.gov NCT02223390 Review team counted the data in the paper that describe 3‐month follow‐up as post‐treatment because the intervention itself lasted for 10 weeks and these data are the first post‐treatment assessment of outcome. Likewise, the 6‐month follow‐up was treated as 3‐month post‐treatment follow‐up for our purposes. Included in meta‐analysis

**Table:** Surís 2013

***Study characteristics***	
Methods	**Study design:** randomised controlled trial **Study grouping:** parallel group **Duration of study:** not given **Location of study:** Dallas, Texas, USA **Randomisation method:** for the purpose of randomisation, participants were assigned sequential PIN numbers as they entered the study. Blocks of random numbers were generated for each therapist and were allocated to either CPT or PCT using a conditional statement. The random number sequence was maintained on an Excel spreadsheet, and as participants’ PINs were entered into the spreadsheet, the preassigned condition was revealed. Assignment to therapist was based on therapist availability. **Ethics approvals:** all study‐related procedures were conducted in compliance with the IRB of the VA North Texas Health Care System, Dallas VA Medical Center.
Participants	**Baseline characteristics** CBT (CPT) *Participants (completers)*: 52 *Gender*: 83% female participants; 17% (9) male participants *Age, M (SD)*: 44.6 years (10.5 years) *Ethnicity*: 44% white, non‐Hispanic, 39% Black, 17% other *Disability*: 61% (based on disability status approved), 20% (based on PTSD disability status approved) *Time since trauma*: not given *Income*: not given *Education (% high school, college or apprentice, university or years of education)*: 17% high school, 67% college, 16% post‐college *Employment (% employed, unemployed, other)*: 44% employed, 35% unemployed, 21% other *Sexual violence in adulthood*: 100% *Comorbid conditions*: 61% VA disability status *Baseline PTSD, M (SD)*: 85.07 (2.69) *Partnered*: 29% *Randomised (N)*: 72 *Completed post‐treatment assessment (N)*: 45 *Dropped out or removed prior to analysis (N)*: 20 *Numbers analysed at final applicable time point (N)*: 52 *Number of sessions, M (SD)*: 9.7 (3.5) *Treatment completion*: 34 (47%) Other psychologically orientated interventions (PCT) *Participants (completers)*: 34 *Gender:* 88% female participants, 12% (4) male participants *Age, M (SD)*: 48.4 years (8.2 years) *Ethnicity*: 44% white, non‐Hispanic, 44% Black, 12% other *Disability*: 65% (based on disability status approved), 24% (based on PTSD disability status approved) *Time since trauma*: not given *Income*: not given *Education (% high school, college or apprentice, university or years of education)*: 26% high school, 62% college, 12% post‐college *Employment (% employed, unemployed, other)*: 32% employed, 41% unemployed, 27% other *Sexual violence in adulthood*: 100% *Comorbid conditions*: 65% VA disability status *Baseline PTSD, M (SD)*: 83.81 (3.29) *Partnered*: 32% *Randomised (N)*: 57 *Completed post‐treatment assessment (N)*: 44 *Dropped out or removed prior to analysis (N)*: 23 *Numbers analysed at final applicable time point (N)*: 34 *Number of sessions, M (SD)*: 10.7 (3.1) *Treatment completion*: 28 (49%) Overall *Participants (select at randomisation if available)*: 86 *Gender*: 85% female participants, 15% male participants *Age, M (SD)*: 46.1 years (9.8 years) *Ethnicity*: 44% white, non‐Hispanic; 41% Black; 15% other *Disability*: 62% (based on disability status approved), 21% (based on PTSD disability status approved) *Time since trauma*: not given *Income*: not given *Education (% high school, college or apprentice, university or years of education)*: 21% high school, 65% college, 14% post‐college *Employment (% employed, unemployed, other)*: 39% employed, 37% unemployed, 24% other *Sexual violence in adulthood*: 100% *Comorbid conditions*: 62% VA disability status *Baseline PTSD*: not given *Partnered*: 30% *Approached (N)*: 481 *Ineligible (N)*: 147 *Declined (N)*: 119 *Other (N)*: 86 *Randomised (N)*: 129 *Completed post‐treatment assessment (N)*: 89 *Dropped out or removed prior to analysis (N)*: 43 *Numbers analysed at final applicable time point (N)*: 86 **Inclusion criteria** Veteran status with a current diagnosis of PTSD related to MST MST event occurred ≥ 3 months prior to study entry MST was the veteran’s lifetime trauma associated with the most severe current distress Veteran had > 1 clear memory of the trauma Any psychiatric medication regimen was stable for ≥ 6 weeks **Exclusion criteria** Active substance dependence within the last 3 months Current psychotic symptoms Current unstable bipolar disorder Current prominent suicidal or homicidal intent Severe cognitive impairment Currently receiving other psychotherapy specifically for PTSD Current involvement in a violent relationship **Pretreatment:** although the 2 treatment groups did not significantly differ in any demographic characteristics, the baseline CAPS re‐experiencing subscale (CAPS B) was significantly higher for the CPT than for the PCT group. This difference was addressed in relevant statistical analyses.
Interventions	**Intervention characteristics** CBT (CPT) *Planned number of intervention sessions*: 12 *Mode of delivery (face‐to‐face, online, video, telephone, blend, with explanation)*: face‐to‐face *Format (group, individual, blend)*: individual *Therapist qualifications and training appropriate (yes, no, unclear, with explanation)*: yes, 4 master's‐ or doctoral‐level mental health providers were trained in both the CPT and PCT protocols. *Research allegiance or conflict of interest (yes, no, unclear, with explanation)*: unclear, as Chard, a co‐author of the current paper, is one of the authors of the adapted CPT tested in this randomised controlled trial. Thus, there may have been an incentive to see the therapy work. On the other hand, Alina Suris led the current paper and is based at different institution, and there were good efforts to monitor fidelity. *Treatment fidelity (yes, no, unclear, with explanation)*: yes, all therapy sessions were videotaped after obtaining informed consent. A minimum of 2 practice cases were required for each treatment modality. Therapists received weekly supervision, including videotape review. Twelve per cent of therapy tapes from each condition were randomly selected for rating by an independent expert. Ratings were made on a scale of 1 = poor to 7 = excellent, with 4 = satisfactory. Ratings were made for unique and essential elements specific to each session of each therapy approach, as well as essential but not unique elements (i.e. rapport‐related) that were the same for every session across both approaches. Ratings were averaged across sessions for each therapist, for each condition. Treatment fidelity analysis indicated a below‐satisfactory competency rating in the CPT condition for 1 therapist. This therapist’s data were excluded from study analyses (129 to 86). *Intervention aim and theoretical basis*: CPT is a manualised CBT developed by Resick 1993 for the treatment of rape‐related PTSD and further adapted for the treatment of PTSD in veterans and military personnel (Resick 2008b). CPT provides a framework for conceptualising PTSD as a disorder of nonrecovery from trauma. Interventions include education, CR and writing trauma narratives. The first 7 sessions address education, examination of thoughts through Socratic dialogue and skill building; the remaining 5 sessions challenge beliefs surrounding themes of safety, trust, power, self‐esteem and intimacy. *Duration of intervention*: 6 weeks to 12 weeks Other psychologically orientated interventions (PCT) *Planned number of intervention sessions*: 12 *Mode of delivery (face‐to‐face, online, video, telephone, blend, with explanation)*: face‐to‐face *Format (group, individual, blend)*: individual *Therapist qualifications and training appropriate (yes, no, unclear, with explanation)*: yes, as previous *Research allegiance or conflict of interest (yes, no, unclear, with explanation)*: no *Treatment fidelity (yes, no, unclear, with explanation)*: yes, as previous *Intervention aim and theoretical basis*: PCT is a manualised therapy for the treatment of PTSD, but without the cognitive‐behavioural or trauma‐focused components of CPT. In PCT, therapeutic focus is deliberately redirected away from discussion of traumatic events. PCT provides general support and education focused on current issues in the participant’s life. Emphasis is on problem‐solving and improving relationships, with connections made between current problems and PTSD symptoms. Like CPT, PCT utilises a written component, but in the form of daily journaling as opposed to written trauma accounts. In the current study, the number of sessions of PCT was extended from 10 to 12 to match the time and attention components of CPT. The PCT manual has not been published. *Duration of intervention*: 6 weeks to 12 weeks
Outcomes	**PTSD (PCL‐C)** Outcome type: continuous outcome Scale: PCL‐C Direction: lower is better Score range: 17 to 85 Data value: endpoint, 3 months and 6 months **PTSD** Outcome type: continuous outcome Scale: CAPS Direction: lower is better Score range: 0 to 80 Data value: endpoint, 3 months and 6 months **Depression** Outcome type: continuous outcome Scale: Quick Inventory of Depressive Symptomology Direction: lower is better Score range: 0 to 27 Data value: endpoint, 3 months and 6 months **PTSD** Outcome type: continuous outcome Scale: PCL‐5 Direction: lower is better Score range: 0 to 80 Data value: endpoint, 3 months and 6 months **PTSD** Outcome type: continuous outcome Scale: PSS‐I Direction: lower is better Score range: 0 to 51 Data value: endpoint, 3 months and 6 months **PTSD** Outcome type: continuous outcome Scale: PSS‐SR Direction: lower is better Score range: 0 to 119 Data value: endpoint, 3 months and 6 months **Depression** Outcome type: continuous outcome Scale: BDI Direction: lower is better Score range: 0 to 63 Data value: endpoint, 3 months and 6 months **PTSD** Outcome type: continuous outcome Scale: IES‐R Intrusion Direction: lower is better Score range: 0 to 4 Data value: endpoint, 3 months and 6 months **PTSD** Outcome type: continuous outcome Scale: IES‐R Avoidance Direction: lower is better Score range: 0 to 4 Data value: endpoint, 3 months and 6 months **PTSD** Outcome type: continuous outcome Scale: IES‐R Hyperarousal Direction: lower is better Score range: 0 to 4 Data value: endpoint, 3 months and 6 months **PTSD** Outcome type: continuous outcome Scale: MPSS‐SR Direction: lower is better Score range: 0 to 119 Data value: endpoint, 3 months and 6 months **Adverse events** Outcome type: adverse event Notes: during the course of the study, there were 3 adverse events in the CPT condition (1 suicide attempt by overdose and 2 psychiatric hospitalisations) and 2 adverse events in the PCT condition (1 suicide attempt by overdose and 1 psychiatric hospitalisation). No events were deemed definitely study‐related; however, 1 psychiatric hospitalisation in the CPT condition was deemed possibly related. "Although we did not collect data on causes of dropout, we hypothesize that this is related to the fact that participants write their trauma narratives as homework for sessions 3 and 4. The writing of trauma narratives can be an especially challenging component of this therapy approach in terms of the emotional demands involved. Dropping out of therapy is one method of avoidance coping when faced with distressing emotions, or even the possibility of distressing emotions. In addition to the emotional demands of directly confronting one’s trauma, CPT is also more demanding in terms of the mental focus and the homework required (as opposed to the trauma‐free journaling of PCT), which may have also contributed to the differential dropout between the treatment groups." (Discussion, p. 35) **Treatment dropout** Outcome type: dichotomous outcome
Identification	**Sponsorship source:** Veterans Administration Rehabilitation Research & Development Service (US government) **Country:** USA **Setting:** outpatients clinic at a VA Medical Centre **Comments:** Clinical trial NCT00371644 at http://clinicaltrials.gov/. There were 13 male participants in this study. We included the 4‐month outcome data as our 3‐month time point. There were no significant differences between the randomised sample of 129 and the final sample of 86 on demographic or baseline measures, and the study only reported the latter group. **Authors name:** Alina Suris **Institution:** VA North Texas Health Care System **Email:** alina.suris@va.gov **Year:** 2013
Notes	Included in meta‐analysis

**Table:** Walsh 2017

***Study characteristics***	
Methods	**Study design:** randomised controlled trial **Study grouping:** parallel group **Duration of study:** recruitment May 2009 to December 2013 **Ethics approvals:** all procedures were approved by 2 university and 2 affiliated hospital IRBs **Location of study:** Midwestern USA **Randomisation method:** the study was designed as a parallel trial with an allocation ratio of 1:1:1. A computerised random numbers generator was used to randomly assign participants to 1 of 3 conditions via a stratified blocked randomisation procedure with variable block sizes of 9 or 12. Nurses who enrolled participants immediately after the study commenced (phase 1) accessed videos for participants via a secure internet link and administered videos prior to the medical examination. Following an approved change of scope, those enrolled in phase 2 (n = 126) were administered videos on CDs following the medical examination that were stored in envelopes prepared by a study coordinator and labelled only with a participant number until opened by the nurse, who was blind to study condition to that point.
Participants	**Baseline characteristics** Other psychosocial interventions (prevention of post‐rape stress video) *Participants (completers/assessed)*: 54 *Gender*: 100% female participants *Age, M (SD)*: 26.79 years (8.22 years) *Ethnicity*: 66.7% minority status *Disability*: not given *Time since trauma, M*: < 7 d *Income*: 84.8% < USD 25,000; 10.9% USD 25,000 to USD 50,000; 4.3% > USD 50,000 *Education (% high school, college or apprentice, university or years of education)*: 27.8% less than high school; 59.3% high school diploma, some college or both; 13% college degree *Employment (% employed, unemployed, other)*: 33.3% employed; 11.1% student *Sexual violence in adulthood*: 96.1% *Comorbid conditions*: 50% past‐year binge‐drinking; 42.6% past‐year marijuana use *Baseline PTSD*: not given *Partnered*: 16.7% *Randomised (N)*: 82 *Completed post‐treatment assessment (N)*: 54 *Dropped out or removed prior to analysis (N)*: 44 *Numbers analysed at final applicable time point (N)*: 38 *Number of sessions, M*: 1 *Treatment completion*: 77 (94%) Behavioural (relaxation video) *Participants (completers/assessed)*: 48 *Gender*: 100% female participants *Age, M (SD)*: 27.99 years (10.77 years) *Ethnicity*: 53.8% minority status *Disability*: not given *Time since trauma, M*: < 7 d *Income*: 78.3% < USD 25,000; 19.6% USD 25,000 to USD 50,000; 2.2% > USD 50,000 *Education (% high school, college or apprentice, university or years of education)*: 22.9% less than high school; 66.7% high school diploma, some college or both; 10.4% college degree *Employment (% employed; unemployed, other)*: 34.4% employed; 10.4% student *Sexual violence in adulthood*: 93.5% *Comorbid conditions*: 48.1% past‐year binge‐drinking; 51.9% past‐year marijuana use *Baseline PTSD*: not given *Partnered*: 10.4% *Randomised (N)*: 82 *Completed post‐treatment assessment (N)*: 48 *Dropped out or removed prior to analysis (N)*: 41 *Numbers analysed at final applicable time point (N)*: 41 *Number of sessions, M*: 1 *Treatment completion*: 77 (94%) Usual care *Participants (completers/assessed)*: 52 *Gender*: 100% female participants *Age, M (SD)*: 27.52 years (10.01 years) *Ethnicity*: 50.0% minority status *Disability*: not given *Time since trauma, M*: < 7 d *Income*: 88.9% < USD 25,000; 6.7% USD 25,000 to USD 50,000; 4.4% > USD 50,000 *Education (% high school, college or apprentice, university or years of education)*: 17.3% less than high school; 78.8% high school diploma, some college or both; 3.8% college degree *Employment (% employed, unemployed, other)*: 34.6% employed; 19.2% student *Sexual violence in adulthood*: 97.5% *Comorbid conditions*: 33.3% past‐year binge‐drinking; 45.8% past‐year marijuana *Baseline PTSD*: not given *Partnered*: 13.5% *Randomised (N)*: 81 *Completed post‐treatment assessment (N)*: 52 *Dropped out or removed prior to analysis (N)*: 39 *Numbers analysed at final applicable time point (N)*: 42 Overall *Participants (completers/assessed)*: 154 *Gender*: 100% female participants *Age, M (SD)*: 27.53 years (9.7 years) *Ethnicity*: 42.9% white; 29.2% Black/African American; 11% Hispanic/Latina, 9.7% multiracial; 7.1% Native American *Disability*: not given *Time since trauma, M*: < 7 d *Income*: 83.9% < USD 25,000; 12.4% USD 25,000 to USD 50,000; 3.6% > USD 50,000 *Education (% high school, college or apprentice, university or years of education)*: 22.7% less than high school; 68.2% high school diploma, some college or both; 9.1% college degree *Employment (% employed, unemployed, other)*: 34.4% employed; 13.6% student *Sexual violence in adulthood*: 95.7% sexual violence in adulthood and 62% had a prior sexual assault *Comorbid conditions*: 44.2% past‐year binge‐drinking; 46.8% past‐year marijuana use *Baseline PTSD*: not given *Partnered*: 13.6% *Approached (N)*: 711 *Ineligible (N)*: 231 *Declined (N)*: 209 *Other (N)*: 26 *Randomised (N)*: 245 *Completed post‐treatment assessment (N)*: 154 *Dropout/removed prior to analysis (N)*: 124 *Numbers analysed at final applicable time point (N)*: 121 **Inclusion criteria** Girls and women 15 years or older who were recent victims of sexual assault (rape, suspected rape or attempted rape) Had a sexual assault medical and forensic examination within 7 d of assault **Exclusion criteria** Non‐English‐speaking Presented with serious injuries or medical issues, psychological distress, acute intoxication or other factors that would preclude participating in informed consent procedures **Pretreatment:** none reported
Interventions	**Intervention characteristics** Other psychosocial interventions (prevention of post‐rape stress video) *Planned number of intervention sessions*: 1 *Mode of delivery (face‐to‐face, online, video, telephone, blend, with explanation)*: video *Format (group, individual, blend)*: individual *Therapist qualifications and training appropriate*: N/A *Research allegiance or conflict of interest (yes, no, unclear, with explanation)*: unclear, authors seem to have developed the video. *Treatment fidelity (yes, no, unclear, with explanation)*: yes, video consistently delivers same content. *Intervention aim and theoretical basis*: this 9‐minute video entitled Steps to Recovery included a female narrator providing information that could be used by victims to prevent future emotional problems and substance abuse, such as instructions for proper implementation of self‐directed exposure exercises, methods to recognise and terminate inappropriate avoidance, and strategies to engage in activities that specifically did not involve alcohol or drug use and avoid situations or cues that have been triggers for use. Video included information, psychoeducation and modelling of adaptive behavioural coping strategies for use post‐assault. *Duration of intervention*: 1 session in the ED Behavioural (relaxation video) *Planned number of intervention sessions*: 1 *Mode of delivery (face‐to‐face, online, video, telephone, blend, with explanation)*: video *Format (group, individual, blend)*: individual *Therapist qualifications and training appropriate*: N/A *Research allegiance or conflict of interest (yes, no, unclear, with explanation)*: no *Treatment fidelity (yes, no, unclear, with explanation)*: yes, video consistently delivers same content *Intervention aim and theoretical basis*: this active control is a 9‐minute video that included a female narrator providing instructions in diaphragmatic breathing, use of words such as 'relax' paired with exhalation, instructions regarding muscle relaxation, and pleasant nature‐related imagery and sounds. The original DVD was a commercial product entitled Relax© (David Garrigus Productions) that was edited for content and length. *Duration of intervention*: 1 session in the ED Usual care *Planned number of intervention sessions*: N/A *Mode of delivery*: N/A *Format*: N/A *Therapist qualifications and training appropriate*: N/A *Research allegiance or conflict of interest*: N/A *Treatment fidelity*: N/A *Intervention aim and theoretical basis*: N/A *Duration of intervention*: N/A
Outcomes	**PTSD** Outcome type: continuous outcome Scale used: PSS‐SR Direction: lower is better Score range: 0 to 119 Data value: endpoint **Substance use** Outcome type: continuous outcome Scale used: DAST Direction: lower is better Score range: 0 to 28 Data value: endpoint **Substance use** Outcome type: continuous outcome Scale used: AUDIT Direction: lower is better Score range: 0 to 40 Data value: endpoint **Adverse events** Outcome type: adverse event **Treatment dropout** Outcome type: dichotomous outcome
Identification	**Sponsorship source:** the study was conducted with support from National Institute on Drug Abuse grant DA023099. Manuscript preparation was supported by National Institutes of Health grants MH107641, MH107641‐02S1, DA036213 and T32‐MH018869. Trial registered at clinicaltrials.gov under the registration number NCT01430624 **Country:** USA **Setting:** 2 medical centres (acute) providing post‐sexual assault medical forensic examinations **Authors name:** Kate Walsh **Institution:** Yeshiva University **Email:** kate.walsh@einstein.yu.edu **Year:** 2017
Notes	No baseline data, and we treated the 6‐week data as post‐treatment; population characteristics by the groups N included in final analysis (n = 154). PTSD data taken from trial registry https://clinicaltrials.gov/ct2/show/results/NCT01430624 Included in meta‐analysis Communicated with the author(s) for purposes of the research

AC: Assessment Control; ADIS‐R: Anxiety Disorders Interview Schedule‐Revised; ART: antiretroviral therapy; ASI: Addiction Severity Index; AUD: Australian dollar; AUDIT: Alcohol Use Disorders Identification Test; BA: Bachelor of Arts degree; BAI: Beck Anxiety Inventory; BDI: Beck Depression Inventory; BS: Bachelor of Science degree; BSI: Brief Symptom Inventory; CAPS: Clinician‐Administered PTSD Scale; CAPS‐5: Clinician‐Administered PTSD Scale for DSM‐5; CBT: cognitive behavioural therapy; CES‐D: Center for Epidemiologic Studies Depression Scale; CI: confidence interval; CPT: cognitive processing therapy; CR: cognitive restructuring; CTSA: Center for the Treatment and Study of Anxiety; DAST: Drug Abuse Screening Test; DSM‐III‐R: *Diagnostic and Statistical Manual of Mental Disorders, Third Edition, Revised*; DSM‐IV: *Diagnostic and Statistical Manual of Mental Disorders, Fourth Edition*; DSM‐5: *Diagnostic and Statistical Manual of Mental Disorders, Fifth Edition*; ED: emergency department; EMDR: eye movement desensitisation and reprocessing; HADS: Hospital Anxiety and Depression Scale; HBT: home‐based telemedicine; HIPAA: Health Insurance Portability and Accountability Act; HRV: heart rate variability; HRVB: heart rate variability biofeedback; IES‐R: Impact of Event Scale – Revised; ImpACT: improving AIDS care after trauma IRB: institutional review board; IRC: International Rescue Committee; IRT: image rehearsal therapy; ITT: intention‐to‐treat; KTA: Karolinska Trial Alliance; LI: Lifespan Integration; LZNF: low‐resolution electromagnetic tomography Z score neurofeedback; M: mean; M‐CET: multiple‐channel exposure therapy; MLI: Modified Lifespan Integration; MPSS‐SR: Modified PTSD Symptom Scale – Self‐Report; MST: military sexual trauma; N/A: not applicable; NEIRB: New England Independent Review Board; NET: narrative exposure therapy; NIMH: National Institute of Mental Health; PAOS: Postacademisch Onderwijs in de Sociale Wetenschappen; PBRS: Pediatric Behavior Rating Scale; PCL‐5: Post‐traumatic Stress Disorder Checklist for DSM‐5 criteria; PCL‐C: Post‐traumatic Stress Disorder Checklist for DSM‐5 criteria – Civilian version; PCT: present‐centred therapy; PDS‐I: Posttraumatic Diagnostic Scale; PDS‐I: Posttraumatic Diagnostic Scale – Interview; PE: prolonged exposure; PEP: postexposure prophylaxis*;* PI: principal investigator; PhD: Doctor of Philosophy; PSS‐I: PTSD Symptom Scale – Interview; PSS‐SR: PTSD Symptom Scale – Self‐Report; PTCI: Post‐traumatic Cognitions Inventory; PTSD: post‐traumatic stress disorder; QOLI: Quality of Life Inventory; RAST: Rape Aftermath Symptom Test; REDCap: Research Electronic Data Capture; RTM: reconsolidation of traumatic memories; SANE: sexual assault nurse examiner; SC: supportive counselling; SD: standard deviation; SF‐36: 36‐item Short‐Form Health Survey; SHE: Safe and Healthy Experiences programme; SIT: stress inoculation therapy; SoC: standard of care; ssmCPT: sleep and symptom monitoring; SSS: Symptom Severity Score; STAI: State‐Trait Anxiety Inventory; SUDs: subjective units of discomfort scale; TAU: treatment as usual; TCTSY: trauma centre trauma‐sensitive yoga; TRGI: Trauma‐Related Guilt Inventory; USD: US dollar; VA: Veterans Affairs; VSLA: Village Savings and Loans Associations; WHO: World Health Organization; WOAR: Women Organized Against Rape; WONSA: World of No Sexual Abuse

### Characteristics of excluded studies [ordered by study ID]

**Table:**

Study	Reason for exclusion
Annan 2017	Ineligible population: focus on IPV survivors^b^
Arntz 2007	Ineligible population: trauma population but did not meet our inclusion criterion that 50% of those randomised had been subjected to adulthood sexual violence and abuse^a^
Boals 2016	Ineligible population: trauma population but did not meet our inclusion criterion that 50% of those randomised had been subjected to adulthood sexual violence and abuse^a^
Bragesjö 2021	Ineligible population: trauma population but did not meet our inclusion criterion that 50% of those randomised had been subjected to adulthood sexual violence and abuse^a^
Bryant 2017	Ineligible population: trauma population but did not meet our inclusion criterion that 50% of those randomised had been subjected to adulthood sexual violence and abuse^a^
Cheung 2019	Ineligible population: focus was on IPV survivors^b^
Coffey 2016	Ineligible population: trauma population but did not meet our inclusion criterion that 50% of those randomised had been subjected to adulthood sexual violence and abuse^a^
Crespo 2010	Ineligible population: focus was on IPV survivors^b^
Devilly 1999	Ineligible population: trauma population but did not meet our inclusion criterion that 50% of those randomised had been subjected to adulthood sexual violence and abuse^a^
Echeburúa 1997	Ineligible population: trauma population but did not meet our inclusion criterion that 50% of those randomised had been subjected to adulthood sexual violence and abuse^a^
Graham‐Bermann 2015	Ineligible population: focus was on IPV survivors^b^
Ha 2019	Ineligible population: childhood trauma population but did not meet our inclusion criterion that 50% of those randomised had been subjected to adulthood sexual violence and abuse^a^
Jalal 2020	Ineligible population: trauma population but did not meet our inclusion criterion that 50% of those randomised had been subjected to adulthood sexual violence and abuse^a^
Johnson 2020	Ineligible population: focus was on IPV survivors^b^
Kanady 2018	Ineligible population: trauma population but did not meet our inclusion criterion that 50% of those randomised had been subjected to adulthood sexual violence and abuse^a^
Kip 2013	Ineligible population: combat trauma population and did not meet our inclusion criterion that 50% of those randomised had been subjected to adulthood sexual violence and abuse^a^
Krupnick 2008	Ineligible population: trauma population but did not meet our inclusion criterion that 50% of those randomised had been subjected to adulthood sexual violence and abuse^a^
Kubany 2004	Ineligible population: focus was on IPV survivors^b^
Latif 2017	Ineligible population: focus was on IPV survivors^b^
Lee 2021	Ineligible population: childhood trauma population and did not meet our inclusion criterion that 50% of those randomised had been subjected to adulthood sexual violence and abuse^a^
Meffert 2021	Ineligible population: trauma population but did not meet our inclusion criterion that 50% of those randomised had been subjected to adulthood sexual violence and abuse^a^
Patel 2019	Ineligible population: focus was on IPV survivors^b^
Rothbaum 2012	Ineligible population: trauma population but did not meet our inclusion criterion that 50% of those randomised had been subjected to adulthood sexual violence and abuse^a^
Sack 2016	Ineligible population: trauma population but did not meet our inclusion criterion that 50% of those randomised had been subjected to adulthood sexual violence and abuse^a^
Saftlas 2014	Ineligible population: focus was on IPV survivors^b^
Scheck 1998	Ineligible population: trauma population but did not meet our inclusion criterion that 50% of those randomised had been subjected to adulthood sexual violence and abuse^a^
Sharma 2018	Ineligible population: focus was on IPV survivors^b^
Tiwari 2012	Ineligible population: focus was on IPV survivors^b^
Tiwari 2017	Ineligible population: focus was on IPV survivors^b^
Wagman 2015	Ineligible population: focus was on IPV survivors and HIV population^b^
Wells 2019	Ineligible population: trauma population but did not meet our inclusion criterion that 50% of those randomised had been subjected to adulthood sexual violence and abuse^a^

^a^For trials delivered in PTSD and trauma populations, we searched for proportions affected by rape, sexual assault or abuse in adulthood. If these were missing, we contacted authors and requested disaggregated data. Where the proportion of randomised participants affected by sexual violence and abuse in adulthood was > 50%, we excluded trials. ^b^Whilst we recognise that a substantial proportion of victims of IPV are subjected to sexual abuse, we excluded IPV trials as it is the topic of another Cochrane Review. *Abbreviations* IPV: intimate partner violence; PTSD: post‐traumatic stress disorder

### Characteristics of studies awaiting classification [ordered by study ID]

**Table:** Dutton 2021

Methods	**Scientific title:** The holistic healing arts retreat: an intensive, experiential intervention for survivors of interpersonal trauma
Participants	**Sample size:** 109 (49 retreat now; 60 retreat later) **Location/setting:** USA **Inclusion criteria** Identification as female Self‐reported history of interpersonal trauma (child abuse, sexual assault or domestic violence) Being at least 18 years of age Having previously received clinical or support services related to trauma **Exclusion criteria** Currently living in a violent or abusive relationship Current pregnancy Current alcohol or drug abuse Hospitalisation for emotional distress or suicidal attempts within the past 6 months Inability to speak and write in English
Interventions	Allocation using 1:1 ratio to re‐treat now and re‐treat later conditions **Experimental 1:** re‐treat now (5‐day residential retreat to promote healing for women) **Comparator:** re‐treat later
Outcomes	PTSD symptoms (using PCL‐5) Depressive symptoms (using PHQ‐9) Perceived stress (using PSS)
Notes	**Trial registry:** not declared **Trial registration number:** not declared **Funding source:** the Joyful Heart Foundation, the Blue Shield of California Foundation, Sukey Novogratz, the Nathan Cummings Foundation, Jenny Lorant‐Grouf, Bloomingdale’s, the Verizon Foundation, the Annenberg Foundation, and the Dreiseszun Family Foundation

PCL‐5: Post‐Traumatic Stress Disorder Checklist for DSM‐5; PHQ‐9: Physician Health Questionnaire‐9; PSS: Perceived Stress Scale; PTSD: post‐traumatic stress disorder

### Characteristics of ongoing studies [ordered by study ID]

**Table:** IRCT20120619010063N8

Study name	**Public title:** The effectiveness of mindfulness‐based group art therapy (MBAT) on improving psychological symptoms in sexual assault victims **Scientific title:** The effectiveness of mindfulness‐based group art therapy (MBAT) on depression, anxiety, intrusive thoughts and shame in sexual assault victims
Methods	**Design:** randomised trial **Location or setting:** Hamedan, Iran
Participants	**Sample size:** 24 (target) **Inclusion criteria** Being female Having been sexually assaulted Experiencing the trauma with a slight intensity (having a score between 24 and 32 on Weiss and Marmar's IES‐R) Having the doctor's permission for taking drugs (if taken any) in a fixed dosage during the treatment sessions Age range from 18 years to 65 years Signing the informed consent Having been educated for at least 8 years **Exclusion criteria** Diagnosis of schizophrenia, bipolar type I, being delusional, having active suicidal thoughts, substance abuse Possibility of getting uncontrollably anxious or aggressive or having uncontrollable impulses The existence of potential harm for participants Being diagnosed with full criteria of personality disorders Being diagnosed with panic disorder **Recruitment status:** completed
Interventions	**Experimental group:** received 8 sessions of mindfulness‐based art therapy **Comparator:** wait‐list
Outcomes	Depression score on short form of Depression Anxiety Stress Scales (timing of assessment: end of the study and 2 months after the end of study) Anxiety score on short form of Depression Anxiety Stress Scales (timing of assessment: end of the study and 2 months after the end of study) Intrusive thoughts score on the IES‐R (timing of assessment: end of the study and 2 months after the end of study) Shame score on Personal Feelings Questionnaire (timing of assessment: end of the study and 2 months after the end of study)
Starting date	**Study start date:** 23 July 2019 **Study end date:** 23 August 2019
Contact information	**Name(s):** Kheirollah Sadeghi **E‐mail:** khsadeghi@kums.ac.ir
Notes	**Trial registry:**Iranian Registry of Clinical Trials **Trial registration number:**IRCT20120619010063N8 **Funding source:** Kermanshah University of Medical Sciences

**Table:** ISRCTN16806208

Study name	**Public title:** A randomised controlled trial of therapist‐assisted online psychological therapies for post‐traumatic stress disorder **Scientific title:** A randomised controlled trial of therapist‐assisted online psychological therapies for post‐traumatic stress disorder
Methods	**Design:** single‐blind, randomised controlled trial, with an embedded process study **Location or setting:** UK
Participants	**Sample size:** 217 participants (target) **Inclusion criteria** Aged 18 years and above Diagnosis of PTSD (as assessed with the Structured Clinical Interview for DSM‐5) Their current re‐experienced symptoms are linked to 1 or 2 discrete traumatic events that they experienced in adulthood or adolescence, or several traumatic episodes during a longer period of high threat (e.g. domestic abuse, war zone experiences). PTSD is the main psychological problem needing treatment. Willing and able to provide informed consent Able to read and write in English Access to internet Willing to be randomly allocated to one of the psychological treatments or wait If taking psychotropic medication, the dose must be stable for at least 1 month before randomisation. If currently receiving psychological therapy for PTSD, this treatment must have ended before randomisation. **Exclusion criteria** History of psychosis Current substance dependence Current borderline personality disorder Acute serious suicide risk **Recruitment:** completed
Interventions	Allocation using a 3:3:1 ratio **Experimental 1:** internet‐delivered cognitive therapy (iCT‐PTSD) **Experimental 2:** internet‐delivered stress‐management (iStress‐PTSD) **Comparator:** wait‐list
Outcomes	PTSD (PCL‐5, CAPS‐5, IES‐R) (timing of assessment: 6 weeks, 13 weeks, 26 weeks, 39 weeks, 65 weeks) Depression (PHQ‐9) (timing of assessment: 6 weeks, 13 weeks, 26 weeks, 39 weeks, 65 weeks) Anxiety (GAD‐7) (timing of assessment: 6 weeks, 13 weeks, 26 weeks, 39 weeks, 65 weeks) Disability (Work and Social Adjustment Scale) (timing of assessment: 6 weeks, 13 weeks, 26 weeks, 39 weeks, 65 weeks) Sleep problems (Insomnia Sleep Index) (timing of assessment: 6 weeks, 13 weeks, 26 weeks, 39 weeks, 65 weeks) Wellbeing (WHO Five Well‐being Index) (timing of assessment: 6 weeks, 13 weeks, 26 weeks, 39 weeks, 65 weeks) QoL (Endicott QoL Scale) (timing of assessment: 6 weeks, 13 weeks, 26 weeks, 39 weeks, 65 weeks) Health economics (timing of assessment: 6 weeks, 13 weeks, 26 weeks, 39 weeks, 65 weeks) Process measures (timing of assessment: 6 weeks, 13 weeks, 26 weeks, 39 weeks, 65 weeks) Therapeutic alliance (timing of assessment: 2 and 6 weeks) Satisfaction (timing of assessment: 13 weeks) Primary endpoint at 13 weeks
Starting date	**Study start date:** 15 December 2017 **Study end date:** 31 December 2021 (estimated)
Contact information	**Name(s):** Prof Anke Ehlers, University of Oxford **E‐mail:** anke.ehlers@psy.ox.ac.uk
Notes	**Trial registry:**ISRCTN Registry **Trial registration number:** ISRCTN16806208 **Funding source:** Wellcome Trust

**Table:** NCT02808468

Study name	**Public title:** Brief restructuring intervention following trauma exposure (BRITE) **Scientific title:** Developing a brief early cognitive intervention for PTSD and alcohol misuse
Methods	**Study design:** randomised trial **Location/setting:** USA
Participants	**Sample size:** 63 participants who self‐identify as female (actual) **Inclusion criteria** Identifies as female Age > 18 years 2 weeks to 10 weeks post‐sexual assault PTSD symptoms related to recent sexual assault, specifically defined as a minimum of 1 re‐experiencing, 1 avoidance, 2 negative alterations in mood/cognition, or 2 hyperarousal symptoms Drinking > 3 drinks on 1 occasion in the past month and at least 2 reported negative consequences of alcohol use Capacity to provide informed consent English fluency No planned absences that they would be unable to complete 5 weeks of assessments and coaching calls Access to a telephone **Exclusion criteria** Acutely suicidal with intent, plan or both Exhibit current psychosis Previous nonresponse to an adequate trial (8 or more sessions) of CPT **Recruitment status:** completed
Interventions	**Active comparator:** brief cognitive therapy **Control:** assessment only
Outcomes	PTSD (timing of assessment: 12 weeks) Drinking frequency and quantity (timing of assessment: 12 weeks) Alcohol use consequences (timing of assessment: 12 weeks)
Starting date	**Study start date:** 1 March 2015 **Study end date:** February 2020 (actual)
Contact information	**Name(s):** Michele Bedard‐Gilligan, PhD **E‐mail:** not reported
Notes	**Trial registry:**ClinicalTrials.Gov **Trial registration number:**NCT02808468 **Funding source:** National Institute on Alcohol Abuse and Alcoholism (NIAAA)

**Table:** NCT03019497

Study name	**Public title:** Cognitive‐behavioral therapy for treatment of post‐traumatic stress disorder and related problems (CBT‐PTSD‐RP) **Scientific title:** Towards optimization of traumatic cognitive‐behavioral therapy for treatment of post‐traumatic stress disorder and related problems
Methods	**Study design:** randomised trial **Location/setting:** Canada
Participants	**Sample size:** 134 participants (actual) **Inclusion criteria** Exposed to a traumatic event at an adult age Primary PTSD diagnosis according to the DSM‐5 criteria At least 1 PTSD‐related problem (depressive, anxiety or sleep disorders, pain, psychosocial stressors, inadequate social support, substance use disorder) Fluent in French **Exclusion criteria** Schizophrenia diagnostic, current or past psychotic episodes, bipolar disorder, organic cerebral disorder or intellectual deficiency Presence of a trouble linked to a substance developed prior to the traumatic event Physical condition preventing participation in the study (e.g. cerebral trauma) Presence of active suicide ideas **Recruitment status:** completed
Interventions	**Experimental:** CBT with specific modules about a specific related problem **Active comparator:** CBT without specific modules
Outcomes	PTSD (timing of assessment: 1 week, 3 months and 6 months) Depression (timing of assessment: 1 week, 3 months and 6 months) Anxiety (timing of assessment: 1 week, 3 months and 6 months) QoL (timing of assessment: 1 week, 3 months and 6 months) Social support in anxious situations (timing of assessment: 1 week, 3 months and 6 months) Sleep quality (timing of assessment: 1 week, 3 months and 6 months) Life events (timing of assessment: 1 week, 3 months and 6 months) Pain (timing of assessment: 1 week, 3 months and 6 months) Health cost (timing of assessment: 3 months) Social provisions (timing of assessment: 1 week, 3 months and 6 months)
Starting date	**Study start date:** January 2017 **Study end date:** June 2021 (actual)
Contact information	**Name(s):** Stéphane Guay, PhD **E‐mail:** not reported
Notes	**Trial registry:**ClinicalTrials.Gov **Trial registration number:**NCT03019497 **Funding source:** Université de Montréal

**Table:** NCT03429166

Study name	**Public title:** Connecting women to care: home‐based psychotherapy for women with MST living in rural areas (CWC) **Scientific title:** Connecting women to care: home‐based psychotherapy for women with MST living in rural areas
Methods	**Study design:** randomised trial **Location/setting:** USA
Participants	**Sample size:** 170 female veterans (target) **Inclusion criteria** Veteran A positive screen for MST A positive PTSD screen defined as PC‐PTSD cutoff of > 3 **Exclusion criteria** Substance abuse not in remission for at least 3 months Current psychotic symptoms Unmedicated mania or bipolar disorder Prominent current suicidal or homicidal ideation Cognitive impairment indicated by chart diagnoses or observable cognitive difficulties Current involvement in a violent relationship defined as more than casual contact (e.g. dating or living with an abusive partner) **Recruitment status:** recruiting
Interventions	**Experimental:** Non‐trauma‐focused treatment (skills training in affective and interpersonal regulation) **Active comparator:** Non‐trauma‐focused treatment (present centred‐therapy)
Outcomes	PTSD (timing of assessment: 26 weeks) Interpersonal support (timing of assessment: 26 weeks) Interpersonal problems (timing of assessment: 26 weeks) Difficulties in emotion regulation (timing of assessment: 26 weeks) Depression (timing of assessment: 26 weeks) Anxiety (timing of assessment: 26 weeks)
Starting date	**Study start date:** 3 September 2018 **Study end date:** 31 March 2022 (estimated)
Contact information	**Name(s):** Marylene Cloitre, PhD **E‐mail:** not reported
Notes	**Trial registry:**ClinicalTrials.Gov **Trial registration number:**NCT03429166 **Funding source:** VA Office of Research and Development

**Table:** NCT03703258

Study name	**Public title:** Tools for health and resilience implemented after violence exposure (Project THRIVE) **Scientific title:** Preventing risky drinking and PTSD after sexual assault: a web‐based intervention
Methods	**Study design:** randomised trial **Location/setting:** USA
Participants	**Sample size:** 40 participants who self‐identify as female (target) **Inclusion criteria** Informed consent Self‐identification as female Sexual assault, defined as endorsement of unwanted, attempted or completed sexual contact in the past 10 weeks Age > 18 years English fluency Smartphone and internet access at least daily for 3 weeks and at least weekly for 3 months Consumption of > 1 alcoholic drink in the past month > 1 episode of high‐risk drinking in past 6 months, defined as either > 3 drinks on a given day or > 7 drinks in a given week At least 3 symptom clusters endorsed on the PTSD Checklist **Exclusion criteria** Active suicidality Psychosis **Recruitment status:** recruiting
Interventions	**Experimental:** app‐based cognitive‐behavioural intervention **Control:** assessment‐only (app use but restricted to daily surveys and symptom tracker)
Outcomes	Problem drinking (timing of assessment: 3 weeks and 3 months) PTSD (timing of assessment: 3 weeks and 3 months) Anxiety (timing of assessment: 3 weeks and 3 months) Coping self‐efficacy (timing of assessment: 3 weeks and 3 months) Depression (timing of assessment: 3 weeks and 3 months) Alcohol consumption (timing of assessment: 3 weeks and 3 months)
Starting date	**Study start date:** 13 January 2021 **Study end date:** 1 September 2023 (estimated)
Contact information	**Name(s):** Emily Dworkin, PhD; Christine Lee **E‐mail:** edworkin@uw.edu; leecm@uw.edu
Notes	**Trial registry:**ClinicalTrials.Gov **Trial registration number:**NCT03703258 **Funding source:** University of Washington

**Table:** NCT03794986

Study name	**Public title:** Peer online motivational interviewing for sexual and gender minority male survivors **Scientific title:** Peer online motivational interviewing for sexual and gender minority male survivors
Methods	**Study design:** randomised trial **Location/setting:** USA
Participants	**Sample size:** 344 SGM survivors (actual) **Inclusion criteria** 18 years or older English‐speaking men who have sex with men or individuals identifying as SGM males Individuals who report a history of sexual abuse and individuals who self‐report a minimum cutoff score of 3.0 or higher on emotional distress, using a 4‐question symptom inventory **Exclusion criteria** Individuals who endorse active psychosis Individuals who have a cognitive dysfunction SGM men who report that they are currently in formal mental health counselling **Recruitment status:** active, not recruiting
Interventions	6‐week duration **Experimental:** motivational interviewing **Active comparator:** motivational interviewing with trauma‐informed SGM affirmative care
Outcomes	Number of participants who engage in treatment (timing of assessment: within 120 d of last online group) Depression (timing of assessment: 14 d, 60 d and 120 d) PTSD (timing of assessment: 14 d, 60 d and 120 d) Substance abuse (timing of assessment: 14 d, 60 d and 120 d) Psychosocial functioning (timing of assessment: 14 d, 60 d and 120 d) Service utilisation (timing of assessment: 14 d, 60 d and 120 d)
Starting date	**Study start date:** 1 April 2019 **Study end date:** 31 March 2023 (estimated)
Contact information	**Name(s):** Joan Cook, PhD **E‐mail:** not reported
Notes	**Trial registry:**ClinicalTrials.Gov **Trial registration number:**NCT03794986 **Funding source:** Yale University

**Table:** NCT04124380

Study name	**Public title:** Understanding and testing recovery processes for PTSD and alcohol use following sexual assault **Scientific title:** Understanding and testing recovery processes for PTSD and alcohol use following sexual assault
Methods	**Study design:** randomised trial **Location/setting:** USA
Participants	**Sample size:** 180 participants (target) **Inclusion criteria** Identifies as female Between the ages of 18 years and 65 years Reports a sexual assault in the last 4 weeks to 1 year Current PTSD severity of ≥ 23 on the PSS‐I‐5 Current heavy alcohol use (≥ 2 heavy episodic drinking occasions (≥ 4 drinks on 1 occasion) in past month) Access to the internet and a device with a webcam **Exclusion criteria** Current diagnosis of schizophrenia, delusional disorder or organic mental disorder as defined by the DSM‐5 Current diagnosis of bipolar disorder, depression with psychotic features or depression severe enough to require immediate psychiatric treatment (i.e. serious suicide risk with intent and plan) Unwilling or unable to discontinue current trauma‐focused psychotherapy or current substance use psychotherapy Unstable dose of psychotropic medications in the prior 3 months Ongoing intimate relationship with the perpetrator of most recent assault Current diagnosis of a severe substance use disorder according to DSM‐5, other than alcohol in the last month No clear trauma memory Current higher dose use of benzodiazepines (greater than the equivalent of 4 mg of lorazepam, 2 mg of alprazolam, 1.5 mg of clonazepam or 20 mg of diazepam) **Recruitment status:** recruiting
Interventions	**Experimental 1:** imaginal exposure, then alcohol skills training **Experimental 2:** alcohol skills training, then imaginal exposure **Experimental 3:** alcohol skills training, no additional treatment **Experimental 4:** imaginal exposure, no additional treatment **Active comparator:** supportive counselling/telehealth
Outcomes	Alcohol use (timing of assessment: unknown) PTSD (timing of assessment: unknown) Psychosocial functioning (timing of assessment: unknown) QoL (timing of assessment: unknown) Alcohol cravings (timing of assessment: unknown) Alcohol consequences (timing of assessment: unknown) Depression (timing of assessment: unknown) Reward (timing of assessment: unknown) Fear (timing of assessment: unknown)
Starting date	**Study start date:** 22 March 2021 **Study end date:** 31 August 2024 (estimated)
Contact information	**Name(s):** Michele Bedard‐Gilligan, PhD **E‐mail:** mab29@uw.edu
Notes	**Trial registry:**ClinicalTrials.Gov **Trial registration number:**NCT04124380 **Funding source:** University of Washington

**Table:** NCT04582695

Study name	**Public title:** Early intervention following sexual assault **Scientific title:** Integrated early intervention for alcohol use disorder and posttraumatic stress disorder following sexual assault
Methods	**Study design:** randomised trial **Location/setting:** USA
Participants	**Sample size:** 64 participants (target) **Inclusion criteria** Female; any race or ethnicity; age 18 years to 65 years Sexual assault that occurred within the past 6 weeks Participants must be able to comprehend English A score of 3 or greater on the AUDIT‐C screen A score of 33 or greater on the PCL‐5. Participants may also meet criteria for an alcohol use disorder, previous history of PTSD, mood disorder (except bipolar affective disorder, see Exclusion Criteria) or other anxiety disorders (panic disorder, agoraphobia, social phobia, generalised anxiety disorder or obsessive‐compulsive disorder). The inclusion of participants with affective and other anxiety disorders is essential because of the marked frequency of the co‐existence of mood and other anxiety disorders among participants with alcohol use disorder and PTSD (Norman 2018; Zinzow 2012). Participants may meet the DSM‐5 criteria for another substance use disorder if alcohol is the primary substance of choice. Must consent to complete all treatment and follow‐up visits **Exclusion criteria** Lack of any memory of the sexual assault Participants meeting the DSM‐5 criteria for a history of or current psychotic, bipolar or dissociative identify disorder or a current eating disorder, as the study protocol may be therapeutically insufficient Participants experiencing significant withdrawal symptoms, as evidence by a score of 10 or more on the CIWA. These participants will be referred for clinical detoxification and may be reassessed for study eligibility after medically supervised detoxification has been completed. Individuals considered an immediate suicide risk, with current suicidal ideation and intent. These individuals will be referred directly for treatment Individuals on psychotropic medications must be stabilised on it for at least 2 weeks prior to beginning the study. Any other medical or psychiatric conditions that the investigators believe may compromise the individual's ability to safely participate in the study **Recruitment status:** recruiting
Interventions	**Experimental:** written exposure therapy integrated with CBT **Active comparator:** written exposure therapy
Outcomes	Alcohol use disorder symptoms (timing of assessment: 6 weeks) Per cent drinking days (timing of assessment: 6 weeks) PTSD (timing of assessment: 6 weeks) Depression (timing of assessment: 6 weeks) Anxiety (timing of assessment: 6 weeks)
Starting date	**Study start date:** 15 December 2020 **Study end date:** 31 July 2025 (estimated)
Contact information	**Name(s):** Christine K Hahn, PhD; Sudie Back, PhD **E‐mail:** hahnc@musc.edu; backs@musc.edu
Notes	**Trial registry:**ClinicalTrials.Gov **Trial registration number:** NCT04582695 **Funding source:** Medical University of South Carolina

AUDIT‐C: Alcohol Use Disorders Identification Test; CAPS‐5: Clinician‐Administered PTSD Checklist for DSM‐5; CBT: cognitive behavioural therapy; CIWA: Clinical Institute Withdrawal Assessment of Alcohol; CPT: cognitive processing therapy; DSM‐5: *Diagnostic and Statistical Manual of Mental Disorders, Fifth Edition*; GAD‐7: Generalised Anxiety Disorder‐7; IES‐R: Impact of Event Scale‐Revised; IPV: intimate partner violence; ISRCTN: International Standard Randomised Controlled Trial Number; MST: military sexual trauma; PCL‐5: PTSD Checklist for DSM‐5 criteria; PC‐PTSD: Primary Care Posttraumatic Stress Disorder; PHQ: Patient Health Questionnaire; PSS‐I: PTSD Symptom Scale‐Interview, for DSM‐5; PTSD: post‐traumatic stress disorder; QoL: quality of life; SGM: sexual‐ or gender‐minority male; WHO: World Health Organization; VA: Veterans Affairs

## Differences between protocol and review

In our protocol (Brown 2019), we listed Stress Inoculation Training (SIT), Prolonged Exposure Therapy (PET) and Cognitive Processing Therapy (CPT) as examples of integrative therapies, based on our reading of descriptions of these interventions in the literature we used to guide the development of our review. However, when we started to categorise the interventions identified in studies that met our review criteria, we found that not all easily fit with categories of the former Cochrane Depression, Anxiety and Neurosis Group (CCDAN) list of psychological therapies. We therefore consulted experts and clinical psychologists for guidance and concluded that we had previously miscategorised SIT, PET and CPT, which may be better categorised as Cognitive Behavioural Therapy. In addition, trauma‐focused interventions compared to non‐trauma‐focused was an aspect we raised as important in the Protocol ("It is recognised that the main comparison combines all intervention types in one and that it may be more useful to stakeholders to understand effects by type. Thus, depending on availability of data, we will stratify results using primary outcomes for CBT versus inactive controls and behavioural therapies versus inactive controls, presenting these in additional tables"). However, we had not clearly described trauma‐focused interventions in the Protocol. Hence, we edited the Description of the intervention section in light of our increased learning in the topic and our previous oversight, and used these updated categorisations throughout the review.

In the Protocol, we stated we would include all time points but that the primary time point for treatment efficacy would be three months post‐treatment. The selection of this time point had been based on a scoping review done at the outset of the research. However, in the process of selecting studies for inclusion, it became clear that the majority assessed treatment efficacy at post‐treatment (in the days and weeks following treatment), and three months was much less common. We still conducted meta‐analyses based on the outcomes at three months; however, ultimately post‐treatment outcomes were reported in Table 1.

In the Protocol, we intended to extract sexual identity but this was rarely mentioned in any study and was not ultimately listed among the characteristics of included studies. In the Review, we listed additional features for extraction than initially included in the Protocol. These changes included adding information about interventions such as format of delivery, theoretical basis for the intervention, fidelity, mean number of sessions taken up by participants and completion rates. We were more precise in the review about the difference between dropout and non‐completion. Dropout indicated the number of participants who did not complete outcome assessments/evaluation. Non‐completion referred to the number of participants allocated to an intervention group that did not receive the intervention (or access a minimum number of intervention sessions predefined by individual studies as the dose required for 'completion'). We differentiated between study setting and recruitment setting. These differences were implemented so that the data extracted accurately reflected what was reported in the included studies and could be synthesised across all studies.

The review, unlike the protocol, specifies a minimum number of included studies required to perform meta‐analyses. We performed a meta‐analysis if there were three or more studies. This was selected as a threshold given the potential for a very large number of analyses across our four time points and primary and secondary outcomes.

## References

[CD013456-bib-0001] Abrahams N, Jewkes R, Lombard C, Mathews S, Campbell J, Meel B. Impact of telephonic psycho-social support on adherence to post-exposure prophylaxis (PEP) after rape. AIDS Care 2010;22(10):1173-81. [DOI: 10.1080/09540121003692185] [PMID: 20640949]

[CD013456-bib-0002] Acierno R, Jaffe AE, Gilmore AK, Birks A, Denier C, Muzzy W, et al. A randomized clinical trial of in-person vs home-based telemedicine delivery of prolonged exposure for PTSD in military sexual trauma survivors. Journal of Anxiety Disorders 2021;83:102461. [DOI: 10.1016/j.janxdis.2021.102461] [PMID: 34391978]

[CD013456-bib-0003] Gilmore AK, Davis MT, Grubaugh A, Resnick H, Birks A, Denier C, et al. "Do you expect me to receive PTSD care in a setting where most of the other patients remind me of the perpetrator?": home-based telemedicine to address barriers to care unique to military sexual trauma and veterans affairs hospitals. Contemporary Clinical Trials 2016;48:59‐64. [DOI: 10.1016/j.cct.2016.03.004] [PMCID: PMC4926870] [PMID: 26992740] PMC4926870

[CD013456-bib-0004] Gilmore AK, Lopez C, Muzzy W, Brown WJ, Grubaugh A, Oesterle DW, et al. Emotion dysregulation predicts dropout from prolonged exposure treatment among women veterans with military sexual trauma-related posttraumatic stress disorder. Women's Health Issues 2020;30(6):462-9. [DOI: 10.1016/j.whi.2020.07.004] PMC8101418 32843240

[CD013456-bib-0005] NCT02417025. Innovative delivery of evidence based psychotherapy to women with military sexual trauma [Do you really expect me to get MST care in a VA where everyone is male? Innovative delivery of evidence based psychotherapy to women with military sexual trauma (MST)]. clinicaltrials.gov/ct2/show/NCT02417025 (first received 15 December 2014).

[CD013456-bib-0006] Anderson T, Fende Guajardo J, Luthra R, Edwards KM. Effects of clinician-assisted emotional disclosure for sexual assault survivors: a pilot study. Journal of Interpersonal Violence 2010;25(6):1113-31. [DOI: 10.1177/0886260509340542] [PMID: 20410374]

[CD013456-bib-0007] Bass JK, Annan J, McIvor Murray S, Kaysen D, Griffiths S, Cetinoglu T, et al. Controlled trial of psychotherapy for Congolese survivors of sexual violence. New England Journal of Medicine 2013;368(23):2182-91. [DOI: 10.1056/NEJMoa1211853] [PMID: 23738545]

[CD013456-bib-0008] Hall BJ, Bolton PA, Annan J, Kaysen D, Robinette K, Cetinoglu T, et al. The effect of cognitive therapy on structural social capital: results from a randomized controlled trial among sexual violence survivors in the Democratic Republic of the Congo. American Journal of Public Health 2014;104(9):1680‐6. [DOI: 10.2105/AJPH.2014.301981] [PMCID: PMC4151928] [PMID: 25033113] PMC4151928

[CD013456-bib-0009] Murray SM, Augustinavicius J, Kaysen D, Rao D, Murray LK, Wachter K, et al. The impact of cognitive processing therapy on stigma among survivors of sexual violence in eastern Democratic Republic of Congo: results from a cluster randomized controlled trial. Conflict and Health 2018;12:1. [DOI: 10.1186/s13031-018-0142-4] PMC5808396 29449879

[CD013456-bib-0010] NCT01385163. Intervention effectiveness in improving psychosocial and economic well-being of sexual violence survivors in DRC [Study of intervention effectiveness in improving psychosocial and economic well-being of sexual violence survivors in DRC]. clinicaltrials.gov/ct2/show/NCT01385163 (first received 22 June 2011).

[CD013456-bib-0011] Bass J, Murray S, Cole G, Bolton P, Poulton C, Robinette K, et al. Economic, social and mental health impacts of an economic intervention for female sexual violence survivors in Eastern Democratic Republic of Congo. Global Mental Health 2016;3:e19. [DOI: 10.1017/gmh.2016.13] [PMCID: PMC5314746] [PMID: 28596887] PMC5314746

[CD013456-bib-0012] Bell AN, Moss D, Kallmeyer RJ. Healing the neurophysiological roots of trauma: a controlled study examining loreta z-score neurofeedback and HRV biofeedback for chronic PTSD. Neuroregulation 2019;6(2):54‐70. [DOI: 10.15540/nr.6.2.54]

[CD013456-bib-0013] Belleville G, Dubé‐Frenette M, Rousseau A. Efficacy of imagery rehearsal therapy and cognitive behavioral therapy in sexual assault victims with posttraumatic stress disorder: a randomized controlled trial. Journal of Traumatic Stress 2018;31(4):591-601. [DOI: 10.1002/jts.22306] [PMID: 30070398]

[CD013456-bib-0014] NCT03169712. IRT and CBT in sexual assault victims with PTSD [Efficacy of sequential imagery rehearsal therapy and cognitive-behavioural therapy in sexual assault victims with posttraumatic stress disorder: a randomized control trial]. www.clinicaltrials.gov/ct2/show/NCT03169712 (first received 23 May 2017).

[CD013456-bib-0015] Bomyea JA, Stein MB, Lang AJ. Interference control training for PTSD: a randomized controlled trial of a novel computer-based intervention. Journal of Anxiety Disorders 2015;34:33-42. [DOI: 10.1016/j.janxdis.2015.05.010] [PMID: 26114901] [PMID: PMC4532583] PMC4532583

[CD013456-bib-0016] Bowland S, Edmond T, Fallot RD. Evaluation of a spiritually focused intervention with older trauma survivors. Social Work 2012;57(1):73-82. [DOI: 10.1093/sw/swr001] [PMID: 22768630]

[CD013456-bib-0017] Brady F, Chisholm A, Walsh E, Ottisova L, Bevilacqua L, Mason C, et al. Narrative exposure therapy for survivors of human trafficking: feasibility randomised controlled trial. BJPsych Open 2021;7(6):e196. [DOI: 10.1192/bjo.2021.1029] [PMCID: PMC8570103]

[CD013456-bib-0018] Covers ML, De Jongh A, Huntjens RJ, De Roos C, Van den Hout M, Bicanic IA. Early intervention with eye movement desensitization and reprocessing (EMDR) therapy to reduce the severity of post-traumatic stress symptoms in recent rape victims: a randomized controlled trial. European Journal of Psychotraumatology 2021;12(1):1943188. [DOI: 10.1080/20008198.2021.1943188] [PMCID: PMC8439210] [PMID: 34531963] PMC8439210

[CD013456-bib-0019] NL6586. Early intervention with eye movement desensitization and reprocessing to reduce PTSD symptom severity: a randomized controlled trial in recent rape victims. Netherlands Trial Registry. https://www.onderzoekmetmensen.nl/en/trial/19897 (first received 18 October 2017).

[CD013456-bib-0020] Creech SK, Pulverman CS, Kahler CW, Orchowski LM, Shea MT, Wernette GT, et al. Computerized intervention in primary care for women veterans with sexual assault histories and psychosocial health risks: a randomized clinical trial. Journal of General Internal Medicine 2021 May 19 [Epub ahead of print]. [DOI: 10.1007/s11606-021-06851-0] [PMID: 34013470] PMC8971224

[CD013456-bib-0021] NCT02957747. Addressing the health concerns of VA women with sexual trauma (SHE). clinicaltrials.gov/ct2/show/NCT02957747 (first received 8 November 2016).

[CD013456-bib-0022] Echeburua E, De Corral P, Sarasua B, Zubizarreta I. Treatment of acute posttraumatic stress disorder in rape victims: an experimental study. Journal of Anxiety Disorders 1996;10(3):185-99. [DOI: 10.1016/0887-6185(96)89842-2]

[CD013456-bib-0023] Falsetti SA, Resnick HS, Davis JL. Multiple channel exposure therapy for women with PTSD and comorbid panic attacks. Cognitive Behavior Therapy 2008;37(2):117-30. [DOI: 10.1080/16506070801969088] 18470742

[CD013456-bib-0024] Feske U. Treating low-income and minority women with posttraumatic stress disorder: a pilot study comparing prolonged exposure and treatment as usual conducted by community therapists. Journal of Interpersonal Violence 2008;23(8):1027-40. [DOI: 10.1177/0886260507313967] [PMID: 18292398]

[CD013456-bib-0025] Foa EB, Rothbaum BO, Riggs DS, Murdock TB. Treatment of posttraumatic stress disorder in rape victims: a comparison between cognitive behavioral procedures and counseling. Journal of Consulting and Clinical Psychology 1991;59(5):715-23. [DOI: 10.1037//0022-006X.59.5.715] [PMID: 1955605]

[CD013456-bib-0026] Feeny NC, Zoellner LA, Foa EB. Treatment outcome for chronic PTSD among female assault victims with borderline personality characteristics: a preliminary examination. Journal of Personality Disorders 2002;16(1):30‐40. [DOI: 10.1521/pedi.16.1.30.22555] [PMID: 11881159]

[CD013456-bib-0027] Foa EB, Dancu CV, Hembree EA, Jaycox LH, Meadow EA, Street GP. A comparison of exposure therapy, stress inoculation training, and their combination for reducing posttraumatic stress disorder in female assault victims. Journal of Consulting and Clinical Psychology 1999;67(2):194‐200. [DOI: 10.1037//0022-006x.67.2.194] [PMID: 10224729]

[CD013456-bib-0028] Zoellner LA, Feeny NC, Fitzgibbons LA, Foa EB. Response of African American and Caucasian women to cognitive behavioral therapy for PTSD. Behavior Therapy 1999;30(4):581-95. [DOI: 10.1016/S0005-7894(99)80026-4]

[CD013456-bib-0029] Aderka IM, Gillihan SJ, McLean CP, Foa EB. The relationship between posttraumatic and depressive symptoms during prolonged exposure with and without cognitive restructuring for the treatment of posttraumatic stress disorder. Journal of Consulting and Clinical Psychology 2013;81(3):375‐82. [DOI: 10.1037/a0031523] [PMID: 23339538]

[CD013456-bib-0030] Foa EB, Hembree EA, Cahill SP, Rauch SA, Riggs DS, Feeny NC, et al. Randomized trial of prolonged exposure for posttraumatic stress disorder with and without cognitive restructuring: outcome at academic and community clinics. Journal of Consulting and Clinical Psychology 2005;73(5):953-64. [DOI: 10.1037/0022-006X.73.5.953] [PMID: 16287395]

[CD013456-bib-0031] Rauch SA, Grunfeld TE, Yadin E, Cahill SP, Hembree EA, Foa EB. Changes in reported physical health symptoms and social function with prolonged exposure therapy for chronic posttraumatic stress disorder. Depression and Anxiety 2009;26(8):732-8. [DOI: 10.1002/da.20518] [PMID: 18781660]

[CD013456-bib-0032] Foa EB, Zoellner LA, Feeny NC. An evaluation of three brief programs for facilitating recovery after assault. Journal of Traumatic Stress 2006;19(1):29-43. [DOI: 10.1002/jts.20096] [PMID: 16568461]

[CD013456-bib-0033] Zoellner LA, Feeny NC, Eftekhari A, Foa EB. Changes in negative beliefs following three brief programs for facilitating recovery after assault. Depression and Anxiety 2011;28(7):532‐40. [DOI: 10.1002/da.20847] [PMCID: PMC3138647] [PMID: 21721072] PMC3138647

[CD013456-bib-0034] Galovski TE, Harik JM, Blain LM, Elwood LS, Gloth CA, Fletcher TD. Augmenting cognitive processing therapy to improve sleep impairment in PTSD: a randomized controlled trial. Journal of Consulting and Clinical Psychology 2016;84(2):167-77. [DOI: 10.1037/ccp0000059] [PMCID: PMC4738064] [PMID: 26689303] PMC4738064

[CD013456-bib-0035] NCT00725192. Sleep-directed hypnosis as a complement to cognitive processing therapy (CPT) in treating posttraumatic stress disorder (PTSD) [Sleep-directed hypnosis as a complement CPT in treating PTSD ]. clinicaltrials.gov/ct2/show/NCT00725192 (first received 30 July 2008).

[CD013456-bib-0036] Gray RM, Budden-Potts D, Schwall RJ, Bourke FF. An open-label, randomized controlled trial of the reconsolidation of traumatic memories protocol (RTM) in military women. Psychological Trauma: Theory, Pesearch, Practice and Policy 2020 Nov 19 [Epub ahead of print]. [DOI: 10.1037/tra0000986] [PMID: 33211519]

[CD013456-bib-0037] Katz LS, Douglas S, Zaleski K, Williams J, Huffman C, Cojucar G. Comparing holographic reprocessing and prolonged exposure for women veterans with sexual trauma: a pilot randomized trial. Journal of Contemporary Psychotherapy 2014;44(1):9-19. [DOI: 10.1007/s10879-013-9248-6]

[CD013456-bib-0038] Kelly U, Haywood T, Segell E, Higgins M. Trauma-sensitive yoga for post-traumatic stress disorder in women veterans who experienced military sexual trauma: interim results from a randomized controlled trial. Journal of Alternative and Complementary Medicine 2021;27(Suppl 1):S45-S59. [DOI: 10.1089/acm.2020.0417] [PMID: 33788599]

[CD013456-bib-0039] NCT02640690. Trauma-sensitive yoga for female veterans with PTSD who experienced military sexual trauma (PSL II). www.clinicaltrials.gov/ct2/show/NCT02640690 (first received 29 December 2015).

[CD013456-bib-0040] Zaccari B, Loftis J, Hubbard K, Haywood T and Kelly U. Trauma-sensitive yoga and cognitive processing group therapies for women veterans with PTSD: a multisite randomized controlled trial adapted for COVID-19. Global Advances in Health and Medicine 2021;10:31. 10.1089/tmj.2021.0612 PMC9519809 35357957

[CD013456-bib-0041] Krakow B, Hollifield M, Johnston L, Koss M, Schrader R, Warner TD, et al. Imagery rehearsal therapy for chronic nightmares in sexual assault survivors with posttraumatic stress disorder - a randomized controlled trial. JAMA 2001;286(5):537-45. [DOI: 10.1001/jama.286.5.537] [PMID: 11476655]

[CD013456-bib-0042] Krakow BJ, Hollifield M, Schrader R, Koss MP, Tandberg D, Lauriello J, et al. A controlled study of imagery rehearsal for chronic nightmares in sexual assault survivors with PTSD: a preliminary report. Journal of Traumatic Stress 2000;13(4):589-609. [DOI: 10.1023/A:1007854015481] [PMID: 11109233]

[CD013456-bib-0043] Littleton H, Grills A. Changes in coping and negative cognitions as mechanisms of change in online treatment for rape-related posttraumatic stress disorder. Journal of Traumatic Stress 2019;32(6):927-35. [DOI: 10.1002/jts.22447] [PMCID: PMC6938536] [PMID: 31742796] PMC6938536

[CD013456-bib-0044] Littleton HL, Grills AE, Kline KD, Schoemann AM, Dodd JC. The From Survivor to Thriver program: RCT of an online therapist-facilitated program for rape-related PTSD. Journal of Anxiety Disorders 2016;43:41-51. [DOI: 10.1016/j.janxdis.2016.07.010] [PMCID: PMC5056149] [PMID: 27513363] PMC5056149

[CD013456-bib-0045] NCT02777294. Evaluation of web-based CBT for rape victims [Evaluation of web-based CBT for rape victims]. clinicaltrials.gov/ct2/show/NCT02777294 (first received 16 May 2016).

[CD013456-bib-0046] Miller KE, Cranston CC, Davis JL, Newman E, Resnick H. Psychological outcomes after a sexual assault video intervention: a randomized trial. Journal of Forensic Nursing 2015;11(3):129‐36. [DOI: 10.1097/JFN.0000000000000080] [PMID: 26291847]

[CD013456-bib-0047] ACTRN12611001065987. Therapy for acute posttrauma stress after recent sexual assault [Treatment of acute stress disorder secondary to sexual assault in adults: effectiveness of cognitive processing therapy compared with treatment as usual in a community mental health setting]. www.anzctr.org.au/ACTRN12611001065987.aspx (first received 11 October 2011).

[CD013456-bib-0048] Nixon RD, Best T, Wilksch SR, Angelakis S, Beatty LJ, Weber N. Cognitive processing therapy for the treatment of acute stress disorder following sexual assault: a randomised effectiveness study. Behaviour Change 2016;33(4):232‐50. [DOI: 10.1017/bec.2017.2]

[CD013456-bib-0049] Rajan G, Wachtler C, Lee S, Wändell P, Philips B, Wahlström L, et al. A one-session treatment of PTSD after single sexual assault trauma. A pilot study of the WONSA MLI project: a randomized controlled trial. Journal of Interpersonal Violence 2020 Oct 21 [Epub ahead of print]. [DOI: 10.1177/0886260520965973] [PMID: 33084475] PMC9092905

[CD013456-bib-0050] Gallagher MW, Resick PA. Mechanisms of change in cognitive processing therapy and prolonged exposure therapy for PTSD: preliminary evidence for the differential effects of hopelessness and habituation. Cognitive Therapy and Research 2012;36(6):750‐5. [DOI: 10.1007/s10608-011-9423-6] [PMCID: PMC3866807] [PMID: 24363472] PMC3866807

[CD013456-bib-0051] Galovski TE, Monson CM, Bruce SE, Resick PA. Does cognitive-behavioral therapy for PTSD improve perceived health and sleep impairment? Journal of Traumatic Stress 2009;22(3):197-204. [DOI: 10.1002/jts.20418] [PMCID: PMC2765684] [PMID: 19466746] PMC2765684

[CD013456-bib-0052] Gutner CA, Casement MD, Stavitsky Gilbert K, Resick PA. Change in sleep symptoms across cognitive processing therapy and prolonged exposure: a longitudinal perspective. Behaviour Research and Therapy 2013;51(12):817‐22. [DOI: 10.1016/j.brat.2013.09.008] [PMCID: PMC3849697] [PMID: 24184428] PMC3849697

[CD013456-bib-0053] Larsen SE, Fleming CJ, Resick PA. Residual symptoms following empirically supported treatment for PTSD. Psychological Trauma: Theory, Research, Practice and Policy 2019;11(2):207-15. [DOI: 10.1037/tra0000384] [PMID: 29963892]

[CD013456-bib-0054] NCT00239772. Cognitive processing therapy versus prolonged exposure for treating women with post-traumatic stress disorder brought on by sexual assault [Cognitive processes in PTSD: treatment]. clinicaltrials.gov/show/nct00239772 (first received 13 October 2005).

[CD013456-bib-0055] Nishith P, Nixon RD, Resick PA. Resolution of trauma-related guilt following treatment of PTSD in female rape victims: a result of cognitive processing therapy targeting comorbid depression? Journal of Affective Disorders 2005;86(2-3):259-65. [DOI: 10.1016/j.jad.2005.02.013] [PMCID: PMC2970919] [PMID: 15935245] PMC2970919

[CD013456-bib-0056] Nishith P, Weaver TL, Resick PA, Uhlmansiek MH. General memory functioning at pre- and posttreatment in female rape victims with posttraumatic stress disorder. In: Williams LM, Banyard VL, editors(s). Trauma and Memory. Thousand Oaks (CA): Sage Publications, 1999:47-55.

[CD013456-bib-0057] Resick PA, Nishith P, Weaver TL, Astin MC, Feuer CA. A comparison of cognitive-processing therapy with prolonged exposure and a waiting condition for the treatment of chronic posttraumatic stress disorder in female rape victims. Journal of Consulting and Clinical Psychology 2002;70(4):867‐79. [DOI: 10.1037//0022-006x.70.4.867] [PMCID: PMC2977927] [PMID: 12182270] PMC2977927

[CD013456-bib-0058] Resick PA, Williams LF, Suvak MK, Monson CM, Gradus JL. Long-term outcomes of cognitive-behavioral treatments for posttraumatic stress disorder among female rape survivors. Journal of Consulting and Clinical Psychology 2012;80(2):201‐10. [DOI: 10.1037/a0026602] [PMCID: PMC3336190] [PMID: 22182261] PMC3336190

[CD013456-bib-0059] Rizvi SL, Vogt DS, Resick PA. Cognitive and affective predictors of treatment outcome in cognitive processing therapy and prolonged exposure for posttraumatic stress disorder. Behaviour Research and Therapy 2009;47(9):737‐43. [DOI: 10.1016/j.brat.2009.06.003] [PMCID: PMC3467002] [PMID: 19595295] PMC3467002

[CD013456-bib-0060] Scher CD, Suvak MK, Resick PA. Trauma cognitions are related to symptoms up to 10 years after cognitive behavioral treatment for posttraumatic stress disorder. Psychological Trauma: Theory, Research, Practice and Policy 2017;9(6):750-7. [DOI: 10.1037/tra0000258] [PMID: 28182457] [PMID: PMC5550371] PMC5550371

[CD013456-bib-0061] Wachen JS, Jimenez S, Smith K, Resick PA. Long-term functional outcomes of women receiving cognitive processing therapy and prolonged exposure. Psychological Trauma: Theory, Research, Practice, and Policy 2014;6(Suppl 1):S58-65. [DOI: 10.1037/a0035741]

[CD013456-bib-0062] NCT00245232. Cognitive processing therapy versus its individual components in the treatment of post-traumatic stress disorder and depression in women who have been sexually abused [Cognitive processes in PTSD: treatment]. clinicaltrials.gov/show/nct00245232 (first received 25 October 2005).

[CD013456-bib-0063] Resick PA, Galovski TE, Uhlmansiek MO, Scher CD, Clum GA, Young-Xu Y. A randomized clinical trial to dismantle components of cognitive processing therapy for posttraumatic stress disorder in female victims of interpersonal violence. Journal of Consulting and Clinical Psychology 2008;76(2):243‐58. [DOI: 10.1037/0022-006X.76.2.243] [PMCID: PMC2967760] [PMID: 18377121] PMC2967760

[CD013456-bib-0064] Rothbaum BO. A controlled study of eye movement desensitization and reprocessing in the treatment of posttraumatic stress disordered sexual assault victims. Bulletin of the Menninger Clinic 1997;61(3):317‐34. [PMID: 9260344]

[CD013456-bib-0065] Leiner AS, Kearns MC, Jackson JL, Astin MC, Rothbaum BO. Avoidant coping and treatment outcome in rape-related posttraumatic stress disorder. Journal of Consulting and Clinical Psychology 2012;80(2):317‐21. [DOI: 10.1037/a0026814] [PMCID: PMC3314118] [PMID: 22229757] PMC3314118

[CD013456-bib-0066] Rothbaum BO, Astin MC, Marsteller F. Prolonged exposure versus eye movement desensitization and reprocessing (EMDR) for PTSD rape victims. Journal of Traumatic Stress 2005;18(6):607‐16. [DOI: 10.1002/jts.20069] [PMID: 16382428]

[CD013456-bib-0067] Schnurr PP, Friedman MJ, Engel CC, Foa EB, Shea MT, Chow BK, et al. Cognitive behavioral therapy for posttraumatic stress disorder in women: a randomized controlled trial. JAMA 2007;297(8):820-30. [DOI: 10.1001/jama.297.8.820] [PMID: 17327524]

[CD013456-bib-0068] Schnurr PP, Friedman MJ, Engel CC, Foa EB, Shea MT, Resick PM, et al. Issues in the design of multisite clinical trials of psychotherapy: VA Cooperative Study No. 494 as an example. Contemporary Clinical Trials 2005;26(6):626-36. [DOI: 10.1016/j.cct.2005.09.001] [PMID: 16236558]

[CD013456-bib-0069] Schnurr PP, Lunney CA, Forshay E, Thurston VL, Chow BK, Resick PA, et al. Sexual function outcomes in women treated for posttraumatic stress disorder. Journal of Women's Health 2009;18(10):1549-57. [DOI: 10.1089/jwh.2008.1165] [PMID: 19788366]

[CD013456-bib-0070] Sikkema KJ, Mulawa MI, Robertson C, Watt MH, Ciya N, Stein DJ, et al. Improving AIDS care after trauma (ImpACT): pilot outcomes of a coping intervention among HIV-infected women with sexual trauma in South Africa. AIDS and Behavior 2018;22(3):1039-52. [DOI: 10.1007/s10461-017-2013-1] [PMCID: PMC5828984] [PMID: 29270789] PMC5828984

[CD013456-bib-0071] Holliday R, Holder N, Monteith LL, Surís A. Decreases in suicide cognitions after cognitive processing therapy among veterans with posttraumatic stress disorder due to military sexual trauma: a preliminary examination. Journal of Nervous and Mental Disease 2018;206(7):575-8. [DOI: 10.1097/NMD.0000000000000840] [PMID: 29905663]

[CD013456-bib-0072] Holliday R, Holder N, Surís A. Reductions in self-blame cognitions predict PTSD improvements with cognitive processing therapy for military sexual trauma-related PTSD. Psychiatry Research 2018;263:181‐4. [DOI: 10.1016/j.psychres.2018.03.007] [PMID: 29573657]

[CD013456-bib-0073] Holliday R, Link-Malcolm J, Morris EE, Surís A. Effects of cognitive processing therapy on PTSD-related negative cognitions in veterans with military sexual trauma. Military Medicine 2014;179(10):1077‐82. [DOI: 10.7205/MILMED-D-13-00309] [PMID: 25269124]

[CD013456-bib-0074] Holliday R, Williams R, Bird J, Mullen K, Surís A. The role of cognitive processing therapy in improving psychosocial functioning, health, and quality of life in veterans with military sexual trauma-related posttraumatic stress disorder. Psychological Services 2015;12(4):428‐34. [DOI: 10.1037/ser0000058] [PMID: 26524285]

[CD013456-bib-0075] Holliday RP, Holder ND, Williamson ML, Suris A. Therapeutic response to cognitive processing therapy in white and Black female veterans with military sexual trauma-related PTSD. Cognitive Behaviour Therapy 2017;46(5):432-46. [DOI: 10.1080/16506073.2017.1312511] [PMID: 28485687]

[CD013456-bib-0076] NCT00371644. Treatment for veterans with military sexual trauma [Manualized treatment for veterans with military sexual trauma]. clinicaltrials.gov/ct2/show/NCT00371644 (first received 31 August 2006).

[CD013456-bib-0077] Surís AM, Link-Malcolm J, Chard K, Ahn C, North C. A randomized clinical trial of cognitive processing therapy for veterans with PTSD related to military sexual trauma. Journal of Traumatic Stress 2013;26(1):28-37. [DOI: 10.1002/jts.21765] [PMID: 23325750]

[CD013456-bib-0078] Gilmore AK, Walsh K, Frazier P, Ledray L, Acierno R, Ruggiero KJ, et al. Prescription opioid misuse after a recent sexual assault: a randomized clinical trial of a video intervention. American Journal on Addictions 2019;28(5):376-81. [DOI: 10.1111/ajad.12922] [PMCID: PMC7354707] [PMID: 31242340] PMC7354707

[CD013456-bib-0079] Gilmore AK, Walsh K, Frazier P, Meredith L, Ledray L, Davis J, et al. Post-sexual assault mental health: a randomized clinical trial of a video-based intervention. Journal of Interpersonal Violence 2021;36(21-2):10614-37. [DOI: 10.1177/0886260519884674] PMC7232869 31709903

[CD013456-bib-0080] NCT01430624. Prevention of post sexual assault stress [Prevention of postrape drug abuse: replication study]. clinicaltrials.gov/ct2/show/NCT01430624 (first received 6 September 2011).

[CD013456-bib-0081] Walsh K, Badour CL, Zuromski KL, Gilmore AK, Kilpatrick DG, Acierno R, et al. A secondary analysis of a brief video intervention on suicidal ideation among recent rape victims. Psychological Services 2021;18(4):703-8. [DOI: 10.1037/ser0000495] [PMCID: PMC8417147] [PMID: 33661694] PMC8417147

[CD013456-bib-0082] Walsh K, Gilmore AK, Frazier P, Ledray L, Acierno R, Ruggiero KJ, et al. A randomized clinical trial examining the effect of video-based prevention of alcohol and marijuana use among recent sexual assault victims. Alcoholism Clinical and Experimental Research 2017;41(12):2163-72. [DOI: 10.1111/acer.13505] [PMCID: PMC5711597] [PMID: 28940320] PMC5711597

[CD013456-bib-0083] Walsh K, Gilmore AK, Schumacher JA, Coffey SF, Frazier PA, Ledray L, et al. Post-sexual assault cigarette smoking: findings from a randomized clinical trial of a video-based intervention. Addictive Behaviors 2020;100:106121. [DOI: 10.1016/j.addbeh.2019.106121] [PMCID: PMC6982466] [PMID: 31622944] PMC6982466

[CD013456-bib-0084] Annan J, Falb K, Kpebo D, Hossain M, Gupta J. Reducing PTSD symptoms through a gender norms and economic empowerment intervention to reduce intimate partner violence: a randomized controlled pilot study in Côte D'Ivoire. Global Mental Health 2017;4:e22. [DOI: 10.1017/gmh.2017.19] [PMCID: PMC5719471] [PMID: 29230318] PMC5719471

[CD013456-bib-0085] Arntz A, Tiesema M, Kindt M. Treatment of PTSD: a comparison of imaginal exposure with and without imagery rescripting. Journal of Behavior Therapy and Experimental Psychiatry 2007;38(4):345-70. [DOI: 10.1016/j.jbtep.2007.10.006] [PMID: 18005935]

[CD013456-bib-0086] Boals A, Murrell AR. I am > trauma: experimentally reducing event centrality and PTSD symptoms in a clinical trial. Journal of Loss and Trauma 2016;21(6):471-83. [DOI: 10.1080/15325024.2015.1117930]

[CD013456-bib-0087] Bragesjö M, Arnberg FK, Särnholm J, Olofsdotter Lauri K, Andersson E. Condensed internet-delivered prolonged exposure provided soon after trauma: a randomised pilot trial. Internet Interventions 2021;23:100358. [DOI: 10.1016/j.invent.2020.100358] [PMCID: PMC7771112] [PMID: 33384946] PMC7771112

[CD013456-bib-0088] Bryant RA, Schafer A, Dawson KS, Anjuri D, Mulili C, Ndogoni L, et al. Effectiveness of a brief behavioural intervention on psychological distress among women with a history of gender-based violence in urban Kenya: a randomised clinical trial. PLoS Medicine 2017;14(8):e1002371. [DOI: 10.1371/journal.pmed.1002371] [PMCID: PMC5557357] [PMID: 28809935] PMC5557357

[CD013456-bib-0089] Cheung DS, Deng W, Tsao SW, Ho RT, Chan CL, Fong DY, et al. Effect of a Qigong intervention on telomerase activity and mental health in Chinese women survivors of intimate partner violence: a randomized clinical trial. JAMA Network Open 2019;2(1):e186967. [DOI: 10.1001/jamanetworkopen.2018.6967] [PMCID: PMC6484539] [PMID: 30646209] PMC6484539

[CD013456-bib-0090] Coffey SF, Schumacher JA, Nosen E, Littlefield AK, Henslee AM, Lappen A, et al. Trauma-focused exposure therapy for chronic posttraumatic stress disorder in alcohol and drug dependent patients: a randomized controlled trial. Psychology of Addictive Behaviors 2016;30(7):778‐90. [DOI: 10.1037/adb0000201] [PMCID: PMC5119896] [PMID: 27786516] PMC5119896

[CD013456-bib-0091] Crespo M, Arinero M. Assessment of the efficacy of a psychological treatment for women victims of violence by their intimate male partner. Spanish Journal of Psychology 2010;13(2):849‐63. [DOI: 10.1017/S113874160000250X] [PMID: 20977033]

[CD013456-bib-0092] Devilly GJ, Spence SH. The relative efficacy and treatment distress of EMDR and a cognitive-behavior trauma treatment protocol in the amelioration of posttraumatic stress disorder. Journal of Anxiety Disorders 1999;13(1-2):131-57. [DOI: 10.1016/s0887-6185(98)00044-9] [PMID: 10225505]

[CD013456-bib-0093] Echeburúa E, De Corral P, Zubizarreta I, Sarasua B. Psychological treatment of chronic posttraumatic stress disorder in victims of sexual aggression. Behavior Modification 1997;21(4):433-56. [DOI: 10.1177/01454455970214003] [PMID: 9337600]

[CD013456-bib-0094] Graham-Bermann SA, Miller-Graff L. Community-based intervention for women exposed to intimate partner violence: a randomized control trial. Journal of Family Psychology 2015;29(4):537-47. [DOI: 10.1037/fam0000091] [PMID: 26030027]

[CD013456-bib-0095] Ha N, Bae SM, Hyun MH. The effect of forgiveness writing therapy on post-traumatic growth in survivors of sexual abuse. Sexual and Relationship Therapy 2019;34(1):10-22. [DOI: 10.1080/14681994.2017.1327712]

[CD013456-bib-0096] Jalal B, Kruger Q, Hinton DE. Culturally adapted CBT (CA-CBT) for traumatised indigenous South Africans (Sepedi): a randomised pilot trial comparing CA-CBT to applied muscle relaxation. Intervention 2020;18(1):61-5. [DOI: 10.4103/INTV.INTV_68_18]

[CD013456-bib-0097] Johnson DM, Zlotnick C, Hoffman L, Palmieri PA, Johnson NL, Holmes SC, et al. A randomized controlled trial comparing HOPE treatment and present-centered therapy in women residing in shelter with PTSD from intimate partner violence. Psychology of Women Quarterly 2020;44(4):539-53. [DOI: 10.1177/0361684320953120] [PMCID: PMC8294703] [PMID: 34305273] PMC8294703

[CD013456-bib-0098] Kanady JC, Talbot LS, Maguen S, Straus LD, Richards A, Ruoff L, et al. Cognitive behavioral therapy for insomnia reduces fear of sleep in individuals with posttraumatic stress disorder. Journal of Clinical Sleep Medicine 2018;14(7):1193-203. [DOI: 10.5664/jcsm.7224] [PMCID: PMC6040781] [PMID: 29991428] PMC6040781

[CD013456-bib-0099] Kip KE, Rosenzweig L, Hernandez DF, Shuman A, Sullivan KL, Long CJ, et al. Randomized controlled trial of accelerated resolution therapy (ART) for symptoms of combat-related post-traumatic stress disorder (PTSD). Military Medicine 2013;178(12):1298-309. [DOI: 10.7205/MILMED-D-13-00298] [PMID: 24306011]

[CD013456-bib-0100] Krupnick JL, Green BL, Stockton P, Miranda J, Krause E, Mete M. Group interpersonal psychotherapy for low-income women with posttraumatic stress disorder. Psychotherapy Research 2008;18(5):497‐507. [DOI: 10.1080/10503300802183678] [PMID: 18816001]

[CD013456-bib-0101] Kubany ES, Hill EE, Owens JA, Iannce-Spencer C, McCaig MA, Tremayne KJ, et al. Cognitive trauma therapy for battered women with PTSD (CTT-BW). Journal of Consulting and Clinical Psychology 2004;72(1):3-18. [DOI: 10.1037/0022-006X.72.1.3] [PMID: 14756610]

[CD013456-bib-0102] Latif M, Khanam SJ. Effectiveness of cognitive behaviour therapy in reducing anxiety, depression and violence in women affected by intimate partner violence: a randomized controlled trial from a low-income country. Journal of Postgraduate Medical Institute 2017;31(4):425‐31. [URL: tinyurl.com/j6cj3zcs]

[CD013456-bib-0103] Lee MR, Cha C. A mobile healing program using virtual reality for sexual violence survivors: a randomized controlled pilot study. Worldviews on Evidence-Based Nursing 2021;18(1):50-9. [DOI: 10.1111/wvn.12478] [PMID: 33245631]

[CD013456-bib-0104] Meffert SM, Neylan TC, McCulloch CE, Blum K, Cohen CR, Bukusi EA, et al. Interpersonal psychotherapy delivered by nonspecialists for depression and posttraumatic stress disorder among Kenyan HIV–positive women affected by gender-based violence: randomized controlled trial. PLoS Medicine 2021;18(1):e1003468. [DOI: 10.1371/journal.pmed.1003468] [PMCID: PMC7799784] [PMID: 33428625] PMC7799784

[CD013456-bib-0105] Patel AR, Weobong B, Patel VH, Singla DR. Psychological treatments for depression among women experiencing intimate partner violence: findings from a randomized controlled trial for behavioral activation in Goa, India. Archives of Women's Mental Health 2019;22(6):779-89. [DOI: 10.1007/s00737-019-00992-2] [PMCID: PMC6841649] [PMID: 31363925] PMC6841649

[CD013456-bib-0106] Rothbaum BO, Kearns MC, Price M, Malcoun E, Davis M, Ressler KJ, et al. Early intervention may prevent the development of posttraumatic stress disorder: a randomized pilot civilian study with modified prolonged exposure. Biological Psychiatry 2012;72(11):957-63. [DOI: 10.1016/j.biopsych.2012.06.002] [PMCID: PMC3467345] [PMID: 22766415] PMC3467345

[CD013456-bib-0107] Sack M, Zehl S, Otti A, Lahmann C, Henningsen P, Kruse J, et al. A comparison of dual attention, eye movements, and exposure only during eye movement desensitization and reprocessing for posttraumatic stress disorder: results from a randomized clinical trial. Psychotherapy and Psychosomatics 2016;85(6):357-65. [DOI: 10.1159/000447671] [PMID: 27744424]

[CD013456-bib-0108] Saftlas AF, Harland KK, Wallis AB, Cavanaugh J, Dickey P, Peek-Asa C. Motivational interviewing and intimate partner violence: a randomized trial. Annals of Epidemiology 2014;24(2):144‐50. [DOI: 10.1016/j.annepidem.2013.10.006] [PMID: 24252714]

[CD013456-bib-0109] Scheck MM, Schaeffer JA, Gillette CS. Brief psychological intervention with traumatized young women: the efficacy of eye movement desensitization and reprocessing. Journal of Traumatic Stress 1998;11(1):25-44. [DOI: 10.1023/A:1024400931106] [PMID: 9479674]

[CD013456-bib-0110] Sharma N, Sharma S. (4098) Effect of homeopathy treatment for post-traumatic stress disorder in battered women: randomized wait-list controlled trial. Global Advances in Health and Medicine 2018;7:278‐9. [DOI: 10.1177/2164956118773837]

[CD013456-bib-0111] Tiwari A, Yuk H, Pang P, Fong DY, Yuen F, Humphreys J, et al. Telephone intervention to improve the mental health of community-dwelling women abused by their intimate partners: a randomised controlled trial. Hong Kong Medical Journal 2012;18(Suppl 6):S14-7. [PMID: 23249846]

[CD013456-bib-0112] Tiwari A, Cheung DS, Deng W, Fong DY, Tsao SW. Effect of a qigong intervention on telomerase activity of Chinese women survivors of intimate partner violence: a single-blind, waitlist, randomised controlled trial. Lancet 2017;390(Special Issue 4):S23. [DOI: 10.1016/S0140-6736(17)33161-6]

[CD013456-bib-0113] Wagman JA, Gray RH, Campbell JC, Thoma M, Ndyanabo A, Ssekasanvu J, et al. Effectiveness of an integrated intimate partner violence and HIV prevention intervention in Rakai, Uganda: analysis of an intervention in an existing cluster randomised cohort. Lancet Global Health 2015;3(1):e23‐33. [DOI: 10.1016/S2214-109X(14)70344-4] [PMCID: PMC4370228] [PMID: 25539966] PMC4370228

[CD013456-bib-0114] Wells SY, Glassman LH, Talkovsky AM, Chatfield MA, Sohn MJ, Morland LA, et al. Examining changes in sexual functioning after cognitive processing therapy in a sample of women trauma survivors. Women's Health Issues 2019;29(1):72-9. [DOI: 10.1016/j.whi.2018.10.003] [PMID: 30455090]

[CD013456-bib-0115] Dutton MA, Dahlgren S, Martinez M, Mete M. The holistic healing arts retreat: an intensive, experiential intervention for survivors of interpersonal trauma. Psychological Trauma: Theory, Research, Practice, and Policy 2021 Dec 20 [Epub ahead of print]. [DOI: 10.1037/tra0001178] 34928687

[CD013456-bib-0116] IRCT20120619010063N8. The effectiveness of mindfulness-based group art therapy (MBAT) on improving psychological symptoms in sexual assault victims [The effectiveness of mindfulness-based group art therapy (MBAT) on depression, anxiety, intrusive thoughts and shame in sexual assault victims]. en.irct.ir/trial/40685 (first received 16 July 2019).

[CD013456-bib-0117] Ehlers A, Wild J, Warnock-Parkes E, Grey N, Murray H, Kerr A, et al. A randomised controlled trial of therapist-assisted online psychological therapies for posttraumatic stress disorder (STOP-PTSD): trial protocol. Trials 2020;21(1):355. [DOI: 10.1186/s13063-020-4176-8] [PMCID: PMC7181498] [PMID: 32326954] PMC7181498

[CD013456-bib-0118] ISRCTN16806208. A randomised controlled trial of therapist-assisted online psychological therapies for post-traumatic stress disorder [A randomised controlled trial of therapist-assisted online psychological therapies for post-traumatic stress disorder]. www.isrctn.com/ISRCTN16806208 (first received 5 January 2018). [DOI: 10.1186/ISRCTN16806208]

[CD013456-bib-0119] NCT02808468. Brief Restructuring Intervention Following Trauma Exposure (BRITE) [Developing a brief early cognitive intervention for PTSD and alcohol misuse]. clinicaltrials.gov/ct2/show/NCT02808468 (first received 21 June 2016).

[CD013456-bib-0120] NCT03019497. Cognitive-behavioral Therapy for treatment of Post-traumatic Stress Disorder and Related Problems (CBT-PTSD-RP) [Towards optimization of traumatic cognitive-behavioral therapy for treatment of post-traumatic stress disorder and related problems]. clinicaltrials.gov/ct2/show/NCT03019497 (first received 12 January 2017).

[CD013456-bib-0121] NCT03429166. Connecting women to care: home-based psychotherapy for women with MST living in rural areas (CWC) [Connecting women to care: home-based psychotherapy for women with MST living in rural areas]. clinicaltrials.gov/ct2/show/NCT03429166 (first received 12 February 2018).

[CD013456-bib-0122] NCT03703258. Tools for Health and Resilience Implemented after Violence Exposure (Project THRIVE) [Preventing risky drinking and PTSD after sexual assault: a web-based intervention]. clinicaltrials.gov/ct2/show/NCT03703258 (first received 11 October 2018).

[CD013456-bib-0123] NCT03794986. Peer online motivational interviewing for sexual and gender minority male survivors. clinicaltrials.gov/ct2/show/NCT03794986 (first received 7 January 2019).

[CD013456-bib-0124] NCT04124380. Understanding and testing recovery processes for PTSD and alcohol use following sexual assault. clinicaltrials.gov/ct2/show/NCT04124380 (first received 11 October 2019).

[CD013456-bib-0125] NCT04582695. Early intervention following sexual assault [Integrated early intervention for alcohol use disorder and posttraumatic stress disorder following sexual assault]. www.clinicaltrials.gov/ct2/show/NCT04582695 (first received 9 October 2020).

[CD013456-bib-0126] American Psychiatric Association. Diagnostic and Statistical Manual of Mental Disorders (DSM-5). 5th edition. Arlington (VA): American Psychiatric Publishing, 2013.

[CD013456-bib-0127] Arroyo K, Lundahl B, Butters R, Vanderloo M, Wood DS. Short-term interventions for survivors of intimate partner violence: a systematic review and meta-analysis. Trauma, Violence, & Abuse 2017;18(2):155–71. [DOI: 10.1177/1524838015602736] [PMID: 26335794]

[CD013456-bib-0128] Basile KC, Hertz MF, Back SE. Intimate Partner Violence and Sexual Violence Victimization Assessment Instruments for Use in Healthcare Settings. 1st edition. Atlanta (GA): Centers for Disease Control and Prevention, National Center for Injury Prevention and Control, 2007.

[CD013456-bib-0129] Beck AT, Ward CH, Mendelson M, Mock J, Erbaugh J. An inventory for measuring depression. Archives of General Psychiatry 1961;4(6):561-71. [DOI: 10.1001/archpsyc.1961.01710120031004] [PMID: 13688369]

[CD013456-bib-0130] Beck AT, Rush A, Shaw B Emergy G. Cognitive Therapy of Depression. New York (NY): Guildford Press, 1979.

[CD013456-bib-0131] Beck AT, Emery G, Greenberg RL. Anxiety Disorders and Phobias: A Cognitive Perspective. New York (NY): Basic Books, 1985.

[CD013456-bib-0132] Beck AT, Steer RA, Garbin MG. Psychometric properties of the Beck Depression Inventory: twenty-five years of evaluation. Clinical Psychology Review 1988;8(1):77-100. [DOI: 10.1016/0272-7358(88)90050-5]

[CD013456-bib-0133] Berman AH, Bergman H, Palmstierna T, Schlyter F. Evaluation of the Drug Use Disorders Identification Test (DUDIT) in criminal justice and detoxification settings and in a Swedish population sample. European Addiction Research 2005;11(1):22-31. [DOI: 10.1159/000081413] [PMID: 15608468]

[CD013456-bib-0134] Bernstein DA, Borkovec TD. Progressive Relaxation Training: A Manual for the Helping Professions. Research Press, 1973.

[CD013456-bib-0135] Bernstein EM, Putnam FW. Development, reliability, and validity of a dissociation scale. Journal of Nervous and Mental Disease 1986;174(12):727-35. [PMID: 3783140] 10.1097/00005053-198612000-00004

[CD013456-bib-0136] Bisson JI, Roberts NP, Andrew M, Cooper R, Lewis C. Psychological therapies for chronic post-traumatic stress disorder (PTSD) in adults. Cochrane Database of Systematic Reviews 2013, Issue 12. Art. No: CD003388. [DOI: 10.1002/14651858.CD003388.pub4] [PMID: 24338345] PMC6991463

[CD013456-bib-0137] Blake DD, Weathers FW, Nagy LM, Kaloupek DG, Klauminzer G, Charney DS, et al. A clinician rating scale for assessing current and lifetime PTSD: the CAPS-1. Behavior Therapist 1990;13:187-8.

[CD013456-bib-0138] Blake DD, Weathers FW, Nagy LM, Kaloupek DG, Gusman FD, Charney DS, et al. The development of a clinician-administered PTSD scale. Journal of Traumatic Stress 1995;8(1):75-90. 10.1007/BF02105408 7712061

[CD013456-bib-0139] Blevins CA, Weathers FW, Davis MT, Witte TK, Domino JL. The Posttraumatic Stress Disorder Checklist for DSM-5 (PCL-5): development and initial psychometric evaluation. Journal of Traumatic Stress 2015;28(6):489-98. [DOI: 10.1002/jts.22059] [PMID: 26606250]

[CD013456-bib-0140] Borenstein M, Hedges LV, Higgins JP, Rothstein HR. Introduction to Meta-Analysis. Chichester (UK): John Wiley & Sons, 2011.

[CD013456-bib-0141] Boudewyns PA. Post-traumatic stress disorder: conceptualization and treatment. In: Hersen M, Eisler RM, Miller PM, editors(s). Progress in Behavior Modification. Vol. 30. Pacific Grove (CA): Brooks/Cole Publishing Co, 1996:165-89. 7567675

[CD013456-bib-0142] Boudewyns PA, Hyer LA. Eye movement desensitization and reprocessing (EMDR) as treatment for post-traumatic stress disorder (PTSD). Clinical Psychology & Psychotherapy 1996;3(3):185-95. [DOI: 10.1002/(SICI)1099-0879(199609)3:3&lt;185::AID-CPP101&gt;3.0.CO;2-0]

[CD013456-bib-0143] Bovin MJ, Marx BP, Weathers FW, Gallagher MW, Rodriguez P, Schnurr PP, et al. Psychometric properties of the PTSD Checklist for Diagnostic and Statistical Manual of Mental Disorders - Fifth Edition (PCL-5) in veterans. Psychological Assessment 2016;28(11):1379-91. [DOI: 10.1037/pas0000254] [PMID: 26653052]

[CD013456-bib-0144] Bradley R, Greene J, Russ E, Dutra L, Westen D. A multidimensional meta-analysis of psychotherapy for PTSD. American Journal of Psychiatry 2005;162(2):214-27. [DOI: 10.1176/appi.ajp.162.2.214] [PMID: 15677582]

[CD013456-bib-0145] Brock RN, Parker RA. Proverbs of Ashes: Violence, Redemptive Suffering, and the Search for What Saves Us. Boston: Beacon Press, 2001.

[CD013456-bib-0146] Brooks R. EuroQol: the current state of play. Health Policy 1996;37(1):53-72. [PMID: 10158943] 10.1016/0168-8510(96)00822-6

[CD013456-bib-0147] Brotto LA, Seal BN, Rellini A. Pilot study of a brief cognitive behavioral versus mindfulness-based intervention for women with sexual distress and a history of childhood sexual abuse. Journal of Sex & Marital Therapy 2012;38(1):1-27. [DOI: 10.1080/0092623X.2011.569636] [PMID: 22268979]

[CD013456-bib-0148] Brown SJ, Khasteganan N, Carter GJ, Brown K, Caswell RJ, Howarth E, et al. Survivor, family and professional experiences of psychosocial interventions for sexual abuse and violence: a qualitative evidence synthesis. Cochrane Database of Systematic Reviews 2020, Issue 6. Art. No: CD013648. [DOI: 10.1002/14651858.CD013648] PMC9531960 36194890

[CD013456-bib-0149] Butler AC, Chapman JE, Forman EM, Beck AT. The empirical status of cognitive-behavioral therapy: a review of meta-analyses. Clinical Psychology Review 2006;26(1):17-31. [DOI: 10.1016/j.cpr.2005.07.003] [PMID: 16199119]

[CD013456-bib-0150] Campbell JC. Health consequences of intimate partner violence. Lancet 2002;359(9314):1331-6. [DOI: 10.1016/S0140-6736(02)08336-8] [PMID: 11965295]

[CD013456-bib-0151] Campbell R, Dworkin E, Cabral G. An ecological model of the impact of sexual assault on women’s mental health. Trauma, Violence, & Abuse 2009;10(3):225–46. [DOI: 10.1177/1524838009334456] [PMID: 19433406]

[CD013456-bib-0152] Carlson EB, Putnam FW. An update on the Dissociative Experiences Scale. Dissociation: Progress in the Dissociative Disorders 1993;6(1):16-27.

[CD013456-bib-0153] Australian Centre for Posttraumatic Mental Health. Australian Guidelines for the Treatment of Acute Stress Disorder & Posttraumatic Stress Disorder. Melbourne, Australia: Phoenix Australia, 2013.

[CD013456-bib-0154] Chang I, Lapham SC, Wanberg KW. Alcohol Use Inventory: screening and assessment of first-time driving-while-impaired offenders. I. Reliability and profiles. Alcohol and Alcoholism 2001;36(2):112-21. [PMID: 11259207] 10.1093/alcalc/36.2.112

[CD013456-bib-0155] Cohen J. Statistical Power Analysis for the Behavioral Sciences. New York (NY): Routledge, 1988.

[CD013456-bib-0156] Cohen JA, Mannarino AP, Knudsen K. Treating sexually abused children: 1 year follow-up of a randomised controlled trial. Child Abuse & Neglect 2005;29(2):135-45. [DOI: 10.1016/j.chiabu.2004.12.005] [PMID: 15734179]

[CD013456-bib-0157] Covidence. Version accessed 5 December 2018. Melbourne, Australia: Veritas Health Innovation, 2018. Available at covidence.org.

[CD013456-bib-0158] Coxell AW, King MB. Male victims of rape and sexual abuse. Sexual and Relationship Therapy 2010;25(4):380-91. [DOI: 10.1080/14681994.2010.518725]

[CD013456-bib-0159] Crown Prosecution Service. Draft guidance on pre-trial therapy. www.cps.gov.uk/publication/draft-guidance-pre-trial-therapy (accessed 11 November 2021).

[CD013456-bib-0160] Cryer L, Beutler L. Group therapy: an alternative treatment approach for rape victims. Journal of Sex & Marital Therapy 1980;6(1):40-6. [DOI: 10.1080/00926238008404244] [PMID: 7381947]

[CD013456-bib-0161] Deblinger E, Stauffer LB, Steer RA. Comparative efficacies of supportive and cognitive behavioral group therapies for young children who have been sexually abused and their nonoffending mothers. Child Maltreatment 2001;6(4):332-43. [DOI: 10.1177/1077559501006004006] [PMID: 11675816]

[CD013456-bib-0162] Decker MR, Peitzmeier S, Olumide A, Acharya R, Ojengbede O, Covarrubias L, et al. Prevalence and health impact of intimate partner violence and non-partner sexual violence among female adolescents aged 15–19 years in vulnerable urban environments: a multi-country study. Journal of Adolescent Health 2014;55(Suppl 6):S58-67. [DOI: 10.1016/j.jadohealth.2014.08.022] [PMID: 25454004]

[CD013456-bib-0163] Deeks JJ, Higgins JP, Altman DG, editor(s). Chapter 10: Analysing data and undertaking meta-analyses. In: Higgins JP, Thomas J, Chandler J, Cumpston M, Li T, Page MJ, Welch VA, editor(s). Cochrane Handbook for Systematic Reviews of Interventions Version 6.2 (updated February 2021). Cochrane, 2021. Available from www.training.cochrane.org/handbook.

[CD013456-bib-0164] Derogatis RL. SCL-90-R. Administration, Scoring & Procedures Manual-II for the R(evised) Version and Other Instruments of the Psychopathology Rating Scales Series. 2nd edition. Towson (MD): Clinical Psychometric Research, 1983.

[CD013456-bib-0165] Dobson KS. Handbook of Cognitive-Behavioral Therapies. 3rd edition. New York (NY): Guilford Press, 2009.

[CD013456-bib-0166] Dossa NI, Zunzunegui MV, Hatem M, Fraser W. Fistula and other adverse reproductive health outcomes among women victims of conflict‐related sexual violence: a population‐based cross‐sectional study. Birth 2014;41(1):5-13. 10.1111/birt.12085 24654632

[CD013456-bib-0167] Earles JL, Vernon LL, Yetz JP. Equine-assisted therapy for anxiety and posttraumatic stress symptoms. Journal of Traumatic Stress 2015;28(2):149-52. [DOI: 10.1002/jts.21990] [PMID: 25782709]

[CD013456-bib-0168] Egger M, Davey Smith G, Schneider M, Minder C. Bias in meta-analysis detected by a simple, graphical test. BMJ 1997;315(7109):629-34. [DOI: 10.1136/bmj.315.7109.629] [PMC2127453] [PMID: 9310563] PMC2127453

[CD013456-bib-0169] Egger M, Davey Smith G, Altman EG. Systematic Reviews in Health Care: Meta‐analysis in Context. 2nd edition. London (UK): BMJ Publishing Group, 2001.

[CD013456-bib-0170] Eisen SV, Culhane MA. Behavior And Symptom Identification Scale (BASIS-32). In: Maruish ME, editors(s). The Use of Psychological Testing for Treatment Planning and Outcomes Assessment. Vol. 1: General Considerations. Mahwah (NJ): Lawrence Erlbaum Associates, 1999:759-90.

[CD013456-bib-0171] Elliott R, Watson JC, Goldman RN, Greenberg LS. Learning Emotion-Focused Therapy: The Process-Experiential Approach to Change. American Psychological Association, 2004.

[CD013456-bib-0172] Ellis A. Rejoinder: Elegant and Inelegant RET. The Counseling Psychologist 1977;7(1):73-82.

[CD013456-bib-0173] Fallot RD, The Spirituality Workgroup. Spirituality and Trauma Recovery: a Group Approach. Unpublished Working Paper. Washington, DC: Community Connections & Lutheran Social Services.

[CD013456-bib-0174] Fernández RS, Bavassi L, Forcato C, Pedreira ME. The dynamic nature of the reconsolidation process and its boundary conditions: evidence based on human tests. Neurobiology of Learning and Memory 2016;130:202-12. 10.1016/j.nlm.2016.03.001 26952269

[CD013456-bib-0175] Finkelhor D, Ormrod RK, Turner HA. Poly-victimization: a neglected component in child victimization. Child Abuse & Neglect 2007;31(1):7-26. [DOI: 10.1016/j.chiabu.2006.06.008] [PMID: 17224181]

[CD013456-bib-0176] Foa EB, Kozak MJ. Emotional processing of fear: exposure to corrective information. Psychological Bulletin 1986;99(1):20-35. [PMID: 2871574]

[CD013456-bib-0177] Foa EB, Rothbaum BO, Riggs DS, Murdock TB. Treatment of posttraumatic stress disorder in rape victims: a comparison between cognitive behavioral procedures and counselling. Journal of Consulting and Clinical Psychology 1991;59(5):715-23. [DOI: 10.1037//0022-006x.59.5.715] [PMID: 1955605]

[CD013456-bib-0178] Foa EB, Riggs DS, Dancu CV, Rothbaum BO. Reliability and validity of a brief instrument for assessing post-traumatic stress disorder. Journal of Traumatic Stress 1993;6(4):459-73. [DOI: 10.1002/jts.2490060405]

[CD013456-bib-0179] Foa EB, Riggs DS. Posttraumatic stress disorder and rape. In: Pynoos RS, editors(s). Posttraumatic Stress Disorder: A Clinical Review. Lutherville (MD): Sidran Press, 1994:133-58.

[CD013456-bib-0180] Foa EB, Rothbaum BO, Molnar C. Cognitive-behavioral therapy of post-traumatic stress disorder. In: Friedman MJ, Charney DS, Deutch AY, editors(s). Neurobiological and Clinical Consequences of Stress: From Normal Adaptation to Post-traumatic Stress Disorder. Pennsylvania: Lippincott Williams & Wilkins Publishers, 1995:483-94.

[CD013456-bib-0181] Foa EB, Rothbaum BO. Treating the Trauma of Rape: Cognitive-Behavioural Therapy for PTSD. New York City: Guildford Press, 1998.

[CD013456-bib-0182] Foa EB, Jaycox LH. Cognitive-behavioral theory and treatment of posttraumatic stress disorder. In: Spiegel D, editors(s). Efficacy and Cost-Effectiveness of Psychotherapy. Washington D.C: American Psychiatric Publishing, 1999:23-61.

[CD013456-bib-0183] Foa EB, Hembree E, Rothbaum B. Prolonged Exposure Therapy for PTSD: Emotional Processing of Traumatic Experiences, Therapist Guide. 1st edition. Oxford: Oxford University Press, 2007.

[CD013456-bib-0184] Foa EB, Keane TM, Friedman MJ, Cohen JA, editor(s). Effective Treatments for PTSD: Practice Guidelines from the International Society for Traumatic Stress Studies. 2nd edition. New York (NY): Guilford Press, 2009.

[CD013456-bib-0185] Foa EB, McLean CP. The efficacy of exposure therapy for anxiety-related disorders and its underlying mechanisms: the case of OCD and PTSD. Annual Review of Clinical Psychology 2016;12:1-28. [DOI: 10.1146/annurev-clinpsy-021815-093533] [PMID: 26565122]

[CD013456-bib-0186] Frazier PA. Perceived control and distress following sexual assault: a longitudinal test of a new model. Journal of Personality and Social Psychology 2003;84(6):1257-69. [PMID: 12793588] 10.1037/0022-3514.84.6.1257

[CD013456-bib-0187] Germain V, Marchand A, Bouchard S, Drouin M, Guay S. Effectiveness of cognitive behavioural therapy administered by videoconference for posttraumatic stress disorder. Cognitive Behaviour Therapy 2009;38(1):42-3. 10.1080/16506070802473494 19235601

[CD013456-bib-0188] Gillies D, Maiocchi L, Bhandari AP, Taylor F, Gray C, O'Brien L. Psychological therapies for children and adolescents exposed to trauma. Cochrane Database of Systematic Reviews 2016, Issue 10. Art. No: CD012371. [DOI: 10.1002/14651858.CD012371] [PMID: 27726123] PMC6457979

[CD013456-bib-0189] Goodman LA, Koss MP, Russo NF. Violence against women: physical and mental health effects. Part I: research findings. Applied and Preventative Psychology 1993;2(2):79-89. [DOI: 10.1016/S0962-1849(05)80114-3]

[CD013456-bib-0190] GRADEpro GDT. Version accessed 5 December 2018. Hamilton (ON): McMaster University (developed by Evidence Prime), 2015. Available at gradepro.org.

[CD013456-bib-0191] Gratz KL. Measurement of deliberate self-harm: preliminary data on the Deliberate Self-Harm Inventory. Journal of Psychopathology and Behavioral Assessment 2001;23(4):253-63. [DOI: 10.1023/A:1012779403943]

[CD013456-bib-0192] Gray R, Budden-Potts D, Bourke F. Reconsolidation of traumatic memories for PTSD: a randomized controlled trial of 74 male veterans. Psychotherapy Research 2019;29(5):621-39. [DOI: 10.1080/10503307.2017.1408973] [PMID: 29241423] 10.1080/10503307.2017.1408973

[CD013456-bib-0193] Greenberg LS, Ford CL, Alden LS, Johnson SM. In-session change in emotionally focused therapy. Journal of Consulting and Clinical Psychology 1993;61(1):78-84. 10.1037//0022-006x.61.1.78 8450111

[CD013456-bib-0194] Guina J, Nahhas RW, Kawalec K, Farnsworth S. Are gender differences in DSM-5 PTSD symptomatology explained by sexual trauma? Journal of Interpersonal Violence 1 November 2016 [Epub ahead of print]. [DOI: 10.1177/0886260516677290] [PMID: 27827321]

[CD013456-bib-0195] Guttierez PM, Osman A, Barrios FX, Kopper BA. Development and initial validation of the Self-Harm Behavior Questionnaire. Journal of Personality Assessment 2001;77(3):475-90. 10.1207/S15327752JPA7703_08 11781034

[CD013456-bib-0196] Hamblen JL, Norman SB, Sonis JH, Phelps AJ, Bisson JI, Nunes VD, et al. A guide to guidelines for the treatment of posttraumatic stress disorder in adults: an update. Psychotherapy 2019;56(3):359-73. [DOI: 10.1037/pst0000231] [PMID: 31282712]

[CD013456-bib-0197] Hameed M, O'Doherty L, Gilchrist G, Tirado-Muñoz J, Taft A, Chondros P, et al. Psychological therapies for women who experience intimate partner violence. Cochrane Database of Systematic Reviews 2020, Issue 7. Art. No: CD013017. [DOI: 10.1002/14651858.CD013017.pub2] [PMID: 32608505] [PMID: PMC7390063] PMC7390063

[CD013456-bib-0198] Hamilton M. A rating scale for depression. Journal of Neurology, Neurosurgery and Psychiatry 1960;23(1):56-62. [DOI: 10.1136/jnnp.23.1.56] [PMC495331] [PMID: 14399272] PMC495331

[CD013456-bib-0199] METAREG: Stata module to perform meta-analysis regression. Harbord R, Higgins J. Boston College, Department of Economics (MA): Statistical Software Components S446201, 2004 (revised 5 January 2009).

[CD013456-bib-0200] Harris M, Anglin J. Trauma Recovery and Empowerment: A Clinician's Guide for Working with Women in Groups. New York: Simon and Schuster, 1998.

[CD013456-bib-0201] Harte CB, Hamilton LD, Meston CM. Predictors of attrition from an expressive writing intervention for sexual abuse survivors. Journal of Child Sexual Abuse 2013;22(7):842-57. [DOI: 10.1080/10538712.2013.830670] [PMID: 24125085]

[CD013456-bib-0202] Hayes SC, Luoma JB, Bond FW, Masuda A, Lillis J. Acceptance and commitment therapy: model, processes and outcomes. Behaviour Research and Therapy 2006;44(1):1-25. [DOI: 10.1016/j.brat.2005.06.006] [PMID: 16300724]

[CD013456-bib-0203] Herman JL. Complex PTSD: a syndrome in survivors of prolonged and repeated trauma. Journal of Traumatic Stress 1992;5(3):377-91. [DOI: 10.1002/jts.2490050305]

[CD013456-bib-0204] Hetrick SE, Purcell R, Garner B, Parslow R. Combined pharmacotherapy and psychological therapies for post traumatic stress disorder (PTSD). Cochrane Database of Systematic Reviews 2010, Issue 7. Art. No: CD007316. [DOI: 10.1002/14651858.CD007316.pub2] [PMID: 20614457] PMC12515543

[CD013456-bib-0205] Higgins JPT, Savovic J, Page MJ, Sterne JAC. Revised Cochrane risk-of-bias tool for randomized trial (ROB 2). Available from https://www.riskofbias.info/welcome/rob-2-0-tool/current-version-of-rob-2.

[CD013456-bib-0206] Higgins JP, Savović J, Page MJ, Elbers RG, Sterne JA. Chapter 8: Assessing risk of bias in a randomized trial. In: Higgins JP, Thomas J, Chandler J, Cumpston M, Li T, Page MJ, Welch VA, editor(s). Cochrane Handbook for Systematic Reviews of Interventions Version 6.2 (updated February 2021). Cochrane, 2021. Available from www.training.cochrane.org/handbook.

[CD013456-bib-0207] Higgins JP, Eldridge S, Li T, editor(s). Chapter 23: Including variants on randomized trials. In: Higgins JP, Thomas J, Chandler J, Cumpston M, Li T, Page MJ, Welch VA, editor(s). Cochrane Handbook for Systematic Reviews of Interventions Version 6.2 (updated February 2021). Cochrane, 2021. Available from www.training.cochrane.org/handbook.

[CD013456-bib-0208] Home Office. The role of the Independent Sexual Violence Adviser: essential elements. bit.ly/2En4iOv (accessed 3 January 2019).

[CD013456-bib-0209] Horowitz M, Wilner N, Alvarez W. Impact of Event Scale: a measure of subjective stress. Psychosomatic Medicine 1979;41(3):209-18. [PMID: 472086] 10.1097/00006842-197905000-00004

[CD013456-bib-0210] Horowitz LM, Rosenberg SE, Baer BA, Ureño G, Villaseñor VS. Inventory of Interpersonal Problems: psychometric properties and clinical applications. Journal of Consulting and Clinical Psychology 1988;56(6):885-92. [PMID: 3204198] 10.1037//0022-006x.56.6.885

[CD013456-bib-0211] Huedo-Medina TB, Sánchez-Meca J, Marín-Martínez F, Botella J. Assessing heterogeneity in meta-analysis: Q statistic or I2 index? Psychological Methods 2006;11(2):193-206. [DOI: 10.1037/1082-989X.11.2.193] [PMID: 16784338]

[CD013456-bib-0212] Institute of Medicine. Treatment of Posttraumatic Stress Disorder: An Assessment of the Evidence. Washington (DC): National Academies Press, 2008. [DOI: 10.17226/11955]

[CD013456-bib-0213] Institute of Medicine. Psychosocial Interventions for Mental and Substance Use Disorders: A Framework for Establishing Evidence-Based Standards. Washington (DC): The National Academies Press, 2015. 26203478

[CD013456-bib-0214] Jakubowski KP, Cundiff JM, Matthews KA. Cumulative childhood adversity and adult cardiometabolic disease: a meta‐analysis. Health Psychology 2018;37(8):701-15. [DOI: 10.1037/hea0000637] [PMCID: PMC6109976] [PMID: 30024227] PMC6109976

[CD013456-bib-0215] Jina R, Thomas LS. Health consequences of sexual violence against women. Best Practice & Research Clinical Obstetrics & Gynaecology 2013;27(1):15-26. [DOI: 10.1016/j.bpobgyn.2012.08.012] 22975432

[CD013456-bib-0216] Jonker I, Imbens A. Christianity and Incest. Tunbridge Wells: Search Press, The Limited, 1992.

[CD013456-bib-0217] Kertz S, Bigda-Peyton J, Bjorgvinsson T. Validity of the Generalized Anxiety Disorder-7 Scale in an acute psychiatric sample. Clinical Psychology & Psychotherapy 2013;20(5):456-64. [DOI: 10.1002/cpp.1802] [PMID: 22593009]

[CD013456-bib-0218] Kilpatrick DG. Rape Aftermath Symptom Test. In: Hersen M, Bellack AS, editors(s). Dictionary of Behavioral Assessment Techniques. Oxford (UK): Pergamon Press, 1988:366-7.

[CD013456-bib-0219] Kitchiner NJ, Lewis C, Roberts NP, Bisson JI. Active duty and ex-serving military personnel with post-traumatic stress disorder treated with psychological therapies: systematic review and meta-analysis. European Journal of Psychotraumatology 2019;10(1):1684226. [DOI: 10.1080/20008198.2019.1684226] [PMCID: PMC6853217] [PMID: 31762951] PMC6853217

[CD013456-bib-0220] Koss MP, Gidycz CA, Wisniewski N. The scope of rape: incidence and prevalence of sexual aggression and victimization in a national sample of higher education students. Journal of Consulting and Clinical Psychology 1987;55(2):162-70. [PMID: 3494755] 10.1037//0022-006x.55.2.162

[CD013456-bib-0221] Kubany ES, Haynes SN, Abueg FR, Manke FP, Brennan JM, Stahura C. Development and validation of the Trauma-Related Guilt Inventory (TRGI). Psychological Assessment 1996;8(4):428-44. [DOI: 10.1037/1040-3590.8.4.428]

[CD013456-bib-0222] Kulka RA, Schlenger WE, Fairbank JA, Hough RL, Jordan BK, Marmar CR, et al. National Vietnam Veterans Readjustment Study (NVVRS): Description, Current Status, and Initial PTSD Prevalence Estimates. Final Report. Washington (DC): Veterans Administration, 1988.

[CD013456-bib-0223] Lee JLC, Nader K, Schiller D. An update on memory reconsolidation updating. Trends in Cognitive Science 2017;21(7):531-45. 10.1016/j.tics.2017.04.006 PMC5605913 28495311

[CD013456-bib-0224] Lefebvre C, Glanville J, Briscoe S, Littlewood A, Marshall C, Metzendorf M-I, et al. Chapter 4: Searching for and selecting studies. In: Higgins JP, Thomas J, Chandler J, Cumpston M, Li T, Page MJ, Welch VA, editor(s). Cochrane Handbook for Systematic Reviews of Interventions Version 6.2 (updated February 2021). Cochrane, 2021. Available from www.training.cochrane.org/handbook.

[CD013456-bib-0225] Lehrer PM, Vaschillo E, Vaschillo B. Resonant frequency biofeedback training to increase cardiac variability: rationale and manual for training. Applied Psychophysiology and Biofeedback 2000;25(3):177-91. 10.1023/a:1009554825745 10999236

[CD013456-bib-0226] Lerman I, Davis B, Huang M, Huang C, Sorkin L, Proudfoot J, et al. Noninvasive vagus nerve stimulation alters neural response and physiological autonomic tone to noxious thermal challenge. PLoS One 2019;14(2):e0201212. [DOI: 10.1371/journal.pone.0201212] [PMC6373934] [PMID: 30759089] PMC6373934

[CD013456-bib-0227] MacDonald G, Higgins JPT, Ramchandani P, Valentine JC, Bronger LP, Klein P, et al. Cognitive-behavioural interventions for children who have been sexually abused. Cochrane Database of Systematic Reviews 2012, Issue 5. Art. No: CD001930. [DOI: 10.1002/14651858.CD001930.pub3] [PMID: 22592679] PMC7061273

[CD013456-bib-0228] Macmillan HL, Wathen CN, Barlow J, Fergusson DM, Leventhal JM, Taussig HN. Interventions to prevent child maltreatment and associated impairment. Lancet 2009;373(9659):250-66. [DOI: 10.1016/S0140-6736(08)61708-0] 19056113

[CD013456-bib-0229] McLellan AT, Luborsky L, Woody GE, O'Brien CP. An improved diagnostic evaluation instrument for substance abuse patients. The Addiction Severity Index. Journal of Nervous and Mental Disease 1980;168(1):26-33. [PMID: 7351540] 10.1097/00005053-198001000-00006

[CD013456-bib-0230] McLellan AT, Kushner H, Metzger D, Peters R, Smith I, Grissom G, et al. The Fifth Edition of the Addiction Severity Index. Journal of Substance Abuse Treatment 1992;9(3):199-213. [PMID: 1334156] 10.1016/0740-5472(92)90062-s

[CD013456-bib-0231] Meichenbaum D. Cognitive-Behavior Modification: An Integrative Approach. New York (NY): Plenum Press, 1977. [ISBN 978-1-4757-9739-8]

[CD013456-bib-0232] Moher D, Liberati A, Tetzlaff J, Altman DG, PRISMA Group. Preferred reporting items for systematic reviews and meta-analyses: the PRISMA statement. PLOS Medicine 2009;6(7):e1000097. [DOI: 10.1371/journal.pmed.1000097] [PMC2707599] [PMID: 19621072] PMC2707599

[CD013456-bib-0233] Murphy D, Ross J, Ashwick R, Armour C, Busuttil W. Exploring optimum cut-off scores to screen for probable posttraumatic stress disorder within a sample of UK treatment-seeking veterans. European Journal of Psychotraumatology 2017;8(1):1398001. [DOI: 10.1080/20008198.2017.1398001] [PMCID: PMC5800736] [PMID: 29435200] PMC5800736

[CD013456-bib-0234] Murray SM, Augustinavicius J, Kaysen D, Rao D, Murray LK, Wachter K, et al. The impact of Cognitive Processing Therapy on stigma among survivors of sexual violence in eastern Democratic Republic of Congo: results from a cluster randomized controlled trial. Conflict and Health 2018;12(1):1-9. [DOI: 10.1186/s13031-018-0142-4] [PMCID: PMC5808396] [PMID: 29449879] PMC5808396

[CD013456-bib-0235] Möller A, Söndergaard HP, Helström L. Tonic immobility during sexual assault - a common reaction predicting post-traumatic stress disorder and severe depression. Acta Obstetricia et Gynecologica Scandinavica 2017;96(8):932-8. [DOI: 10.1111/aogs.13174] [PMID: 28589545]

[CD013456-bib-0236] Nader K, Schafe GE, Le Doux JE. Fear memories require protein synthesis in the amygdala for reconsolidation after retrieval. Nature 2000;406:722-6. 10.1038/35021052 10963596

[CD013456-bib-0237] Nappi CM, Drummon SPA, Thorp SR, McQuaid JR. Effectiveness of imagery rehearsal therapy for the treatment of combat-related nightmares in veterans. Behavior Therapy 2011;41(2):237-44. 10.1016/j.beth.2009.03.003 20412888

[CD013456-bib-0238] National Institute for Health and Care Excellence. Post-traumatic stress disorder. www.nice.org.uk/guidance/ng116 (accessed 3 January 2019).

[CD013456-bib-0239] Norman SB, Haller M, Hamblen JL, Southwick SM, Pietrzak RH. The burden of co-occurring alcohol use disorder and PTSD in US military veterans: comorbidities, functioning, and suicidality. Psychology of Addictive Behaviors 2018;32(2):224-9. [DOI: 10.1037/adb0000348] [PMID: 29553778]

[CD013456-bib-0240] National Sexual Violence Resource Center. Assessing patients for sexual violence: a guide for health care providers. www.nsvrc.org/sites/default/files/Publications_NSVRC_Guides_Assessing-patients-for-sexual-violence.pdf (accessed 3 January 2019).

[CD013456-bib-0241] Office for National Statistics. Nature of sexual assault by rape or penetration, England and Wales: year ending March 2020; 18 March 2021. www.ons.gov.uk/peoplepopulationandcommunity/crimeandjustice/articles/natureofsexualassaultbyrapeorpenetrationenglandandwales/yearendingmarch2020.

[CD013456-bib-0242] Oram S, Khalifeh H, Howard LM. Violence against women and mental health. Lancet. Psychiatry 2017;4(2):159-70. [DOI: 10.1016/S2215-0366(16)30261-9] [PMID: 27856393]

[CD013456-bib-0243] Panish LS, Hai AH. The effectiveness of using neurofeedback in the treatment of post-traumatic stress disorder: a systematic review. Trauma, Violence, & Abuse 2020;21(3):541-50. [DOI: 10.1177/1524838018781103] 29890906

[CD013456-bib-0244] Parcesepe AM, Martin SL, Pollock MD, García-Moreno C. The effectiveness of mental health interventions for adult female survivors of sexual assault: a systematic review. Aggression and Violent Behavior 2015;25(Part A):15-25. [DOI: 10.1016/j.avb.2015.06.004]

[CD013456-bib-0245] Parmar MK, Torri V, Stewart LA. Extracting summary statistics to perform meta-analyses of the published literature for survival endpoints. Statistics in Medicine 1998;17(24):2815-34. [PMID: 9921604] 10.1002/(sici)1097-0258(19981230)17:24<2815::aid-sim110>3.0.co;2-8

[CD013456-bib-0246] Pietrzak RH, Goldstein RB, Southwick SM, Grant BF. Prevalence and Axis I comorbidity of full and partial posttraumatic stress disorder in the United States: results from Wave 2 of the National Epidemiologic Survey on Alcohol and Related Conditions. Journal of Anxiety Disorders 2011;25(3):456-65. [DOI: 10.1016/j.janxdis.2010.11.010] [PMC3051041] [PMID: 21168991] PMC3051041

[CD013456-bib-0247] Pitman RK, Orr SP, Altman B, Longpre RE, Poiré RE, Macklin ML. Emotional processing during eye movement desensitization and reprocessing therapy of Vietnam veterans with chronic posttraumatic stress disorder. Comprehensive Psychiatry 1996;37(6):419-29. [PMID: 8932966] 10.1016/s0010-440x(96)90025-5

[CD013456-bib-0248] Pocock SJ, Hughes MD, Lee RJ. Statistical problems in the reporting of clinical trials. A survey of three medical journals. New England Journal of Medicine 1987;317(7):426-32. [DOI: 10.1056/NEJM198708133170706] [PMID: 3614286]

[CD013456-bib-0249] Pradhan B, Chappuis F, Baral D, Karki P, Rijal S, Hadengue A, et al. The Alcohol Use Disorders Identification Test (AUDIT): validation of a Nepali version for the detection of alcohol use disorders and hazardous drinking in medical settings. Substance Abuse Treatment, Prevention and Policy 2012;7:42. [DOI: 10.1186/1747-597X-7-42] [PMC3508982] [PMID: 23039711] PMC3508982

[CD013456-bib-0250] Pulverman CS, Kilimnik CD, Meston CM. The impact of childhood sexual abuse on women's sexual health: a comprehensive review. Sexual Medicine Reviews 2018;6(2):188-200. [DOI: 10.1016/j.sxmr.2017.12.002] [PMID: 29371141] [URL: www.sciencedirect.com/science/article/pii/S2050052117301476]

[CD013456-bib-0251] Pulverman CS, Creech SK. The impact of sexual trauma on the sexual health of women veterans: a comprehensive review. Trauma, Violence, & Abuse 2021;22(4):656-71. [DOI: 10.1177/1524838019870912] [PMID: 31438778]

[CD013456-bib-0252] Radloff LS. The CES-D scale: a self-report depression scale for research in the general population. Applied Psychological Measurement 1977;1(3):385-401. [DOI: 10.1177/014662167700100306]

[CD013456-bib-0253] Reeves BC, Deeks JJ, Higgins JP, Shea B, Tugwell P, Wells GA. Chapter 24: Including non-randomized studies on intervention effects. In: Higgins JP, Thomas J, Chandler J, Cumpston M, Li T, Page MJ, Welch VA, editor(s). Cochrane Handbook for Systematic Reviews of Interventions Version 6.2 (updated February 2021). Cochrane, 2021. Available from www.training.cochrane.org/handbook.

[CD013456-bib-0254] Regehr C, Alaggia R, Dennis J, Pitts A, Saini M. Interventions to reduce distress in adult victims of rape and sexual violence: a systematic review. Research on Social Work Practice. 2013;23(3):257-65. [DOI: 10.1177/1049731512474103]

[CD013456-bib-0255] Resick PA, Jordan CG, Girelli SA, Hutter CK, Marhoefer-Dvorak S. A comparative outcome study of behavioral group therapy for sexual assault victims. Behavior Therapy 1988;19(3):385-401. [DOI: 10.1016/S0005-7894(88)80011-X]

[CD013456-bib-0256] Resick PA, Schnicke MK. Cognitive processing therapy for sexual assault victims. Journal of Consulting and Clinical Psychology 1992;60(5):748-56. [PMID: 1401390] 10.1037//0022-006x.60.5.748

[CD013456-bib-0257] Resick PA, Schnicke MK. Cognitive Processing Therapy for Sexual Assault Victims: A Treatment Manual. Newbury Park (CA): Sage Publications, 1993. 10.1037//0022-006x.60.5.748 1401390

[CD013456-bib-0258] Resick PA, Monson CM, Chard KM. Cognitive Processing Therapy Veteran/Military Version: Therapist's Manual. Washington, DC: Department of Veterans’ Affairs 2008.

[CD013456-bib-0259] Resick PA, Monson PA, Chard KM. Cognitive Processing Therapy: Veteran/military Version. Washington, DC: Department of Veterans Affairs, 2010.

[CD013456-bib-0260] Resnick HS, Yehuda R, Acierno R. Acute post-rape plasma cortisol, alcohol use, and PTSD symptom profile among recent rape victims. Annals of the New York Academy of Sciences 1997;821(1):433-6. [PMID: 9238223] 10.1111/j.1749-6632.1997.tb48298.x

[CD013456-bib-0261] Review Manager 5 (RevMan 5). Version 5.3. Copenhagen: Nordic Cochrane Centre, The Cochrane Collaboration, 2014.

[CD013456-bib-0262] Review Manager Web (RevMan Web). The Cochrane Collaboration, 2019. Available at revman.cochrane.org.

[CD013456-bib-0263] Roberts NP, Roberts PA, Jones N, Bisson JI. Psychological interventions for post-traumatic stress disorder and comorbid substance use disorder: a systematic review and meta-analysis. Clinical Psychology Review 2015;38:25-38. [DOI: 10.1016/j.cpr.2015.02.007] [PMID: 25792193]

[CD013456-bib-0264] Roberts NP, Roberts PA, Jones N, Bisson JI. Psychological therapies for post-traumatic stress disorder and comorbid substance use disorder. Cochrane Database of Systematic Reviews 2016, Issue 4. Art. No: CD010204. [DOI: 10.1002/14651858.CD010204.pub2] [PMID: 27040448] PMC8782594

[CD013456-bib-0265] Rothbaum BO. A controlled study of eye movement desensitization and reprocessing in the treatment of posttraumatic stress disordered sexual assault victims. Bulletin of the Menninger Clinic 1997;61(3):317-34. [PMID: 9260344]

[CD013456-bib-0266] Rozental A, Castonguay L, Dimidjian S, Lambert M, Shafran R, Andersson G, et al. Negative effects in psychotherapy: commentary and recommendations for future research and clinical practice. British Journal of Psychiatry Open 2018;4(4):307-12. [DOI: 10.1192/bjo.2018.42] [PMC6066991] [PMID: 30083384] PMC6066991

[CD013456-bib-0267] Santa Mina EE, Gallop R, Links P, Heslegrave R, Pringle D, Wekerle C, et al. The Self-Injury Questionnaire: evaluation of the psychometric properties in a clinical population. Journal of Psychiatric and Mental Health Nursing 2006;13(2):221-7. [DOI: 10.1111/j.1365-2850.2006.00944.x] [PMID: 16608478]

[CD013456-bib-0268] Schünemann HJ, Oxman AD, Vist GE, Higgins JPT, Deeks JJ, Glasziou P, et al, Cochrane Applicability and Recommendations Methods Group. Chapter 12: Interpreting results and drawing conclusions. In: Higgins JP, Green S, editor(s). Cochrane Handbook for Systematic Reviews of Interventions Version 5.1.0 (updated March 2011). The Cochrane Collaboration, 2011. Available from handbook.cochrane.org.

[CD013456-bib-0269] Schünemann HJ, Oxman AD, Higgins JPT, Vist GE, Glasziou P, Akl E, et al, Cochrane GRADEing Methods Group and the Cochrane Statistical Methods Group. Chapter 11: Completing ‘Summary of findings’ tables and grading the confidence in or quality of the evidence. In: Higgins JP, Churchill R, Chandler J, Cumpston MS, editor(s). Cochrane Handbook for Systematic Reviews of Interventions Version 5.2.0 (updated June 2017). The Cochrane Collaboration, 2017. Available from www.training.cochrane.org/handbook.

[CD013456-bib-0270] Selzer ML. The Michigan Alcoholism Screening Test: the quest for a new diagnostic instrument. American Journal of Psychiatry 1971;127(12):1653-8. [DOI: 10.1176/ajp.127.12.1653] [PMID: 5565851]

[CD013456-bib-0271] Shapiro F. Eye Movement Desensitization and Reprocessing: Basic Principles, Protocols, and Procedures. New York (NY): Guilford Press, 1995.

[CD013456-bib-0272] Sherman JJ. Effects of psychotherapeutic treatments for PTSD: a meta-analysis of controlled clinical trials. Journal of Traumatic Stress 1998;11(3):413-35. [DOI: 10.1023/A:1024444410595] [PMID: 9690185]

[CD013456-bib-0273] Sikkema KJ, Choi KW, Robertson C, Knettel BA, Ciya N, Knippler ET, et al. Development of a coping intervention to improve traumatic stress and HIV care engagement among South African women with sexual trauma histories. Evaluation and Program Planning 2018;68:148-56. [DOI: org/10.1016/j.evalprogplan.2018.02.007] [PMC5953816] [PMID: 29597104] 10.1016/j.evalprogplan.2018.02.007 PMC5953816

[CD013456-bib-0274] Singh D, Chaudoir SR, Escobar MC, Kalichman S. Stigma, burden, social support, and willingness to care among caregivers of PLWHA in home-based care in South Africa. AIDS Care 2011;23(7):839-45. [DOI: 10.1080/09540121.2010.542122] [PMC3125468] [PMID: 21400316] PMC3125468

[CD013456-bib-0275] Skevington SM, Lotfy M, O'Connell KA, WHOQOL Group. The World Health Organization's WHOQOL-BREF quality of life assessment: psychometric properties and results of the international field trial. A report from the WHOQOL group. Quality of Life Research 2004;13(2):299-310. [DOI: 10.1023/B:QURE.0000018486.91360.00] [PMID: 15085902]

[CD013456-bib-0276] Skinner H. The Drug Abuse Screening Test. Addictive Behaviors 1982;7(4):363-71. [PMID: 7183189] 10.1016/0306-4603(82)90005-3

[CD013456-bib-0277] Spielberger CD, Gorsuch RL, Lushene RE. STAI Manual for the State-Trait Anxiety Inventory. Palo Alto (CA): Consulting Psychologists Press, 1970. [hdl.handle.net/10477/2895]

[CD013456-bib-0278] Spitzer RL, Kroenke K, Williams JB. Validation and utility of a self-report version of PRIME-MD. The PHQ Primary Care Study. JAMA 1999;282(18):1737-44. [DOI: 10.1001/jama.282.18.1737] 10568646

[CD013456-bib-0279] Spitzer RL, Kroenke K, Williams JB, Löwe B. A brief measure for assessing generalized anxiety disorder: the GAD-7. Archives of Internal Medicine 2006;166(10):1092-7. [DOI: 10.1001/archinte.166.10.1092] [PMID: 16717171]

[CD013456-bib-0280] Stata. Version 15. College Station, TX, USA: StataCorp, 2017. Available at www.stata.com.

[CD013456-bib-0281] Steenkamp MM, Litz BT, Hoge CW, Marmar CR. Psychotherapy for military-related PTSD: a review of randomized clinical trials. JAMA 2015;314(5):489-500. [DOI: 10.1001/jama.2015.8370] [PMID: 26241600]

[CD013456-bib-0282] Stefanovics EA, Rosenheck RA, Jones KM, Huang G, Krystal JH. Minimal clinically important differences (MCID) in assessing outcomes of post-traumatic stress disorder. Psychiatry Quarterly 2018;89(1):141-55. [DOI: 10.1007/s11126-017-9522-y] [PMID: 28634644]

[CD013456-bib-0283] Sterne JAC, Savović J, Page MJ, Elbers RG, Blencowe NS, Boutron I, et al. RoB 2: a revised tool for assessing risk of bias in randomised trials. BMJ 2019;366:I4898. [DOI: 10.1136/bmj.l4898] [PMID: 31462531]

[CD013456-bib-0284] Surıs A, Lind L. Military sexual trauma: review of prevalence and associated health consequences in veterans. Trauma, Violence, & Abuse 2008;9(4):250–69. [DOI: 10.1177/1524838008324419] [PMID: 18936282]

[CD013456-bib-0285] Suzuki A, Josselyn SA, Frankland PW, Masushige S, Silva AJ, Kida S. Memory reconsolidation and extinction have distinct temporal and biochemical signatures. Journal of Neuroscience 2004;24(20):4787-95. 10.1523/JNEUROSCI.5491-03.2004 PMC6729467 15152039

[CD013456-bib-0286] Tan G, Dao TK, Farmer L, Sutherland RJ, Gevirtz R. Heart rate variability (HRV) and posttraumatic stress disorder (PTSD): a pilot study. Applied Psychophysiology and Biofeedback 2011;36(1):27-35. 10.1007/s10484-010-9141-y 20680439

[CD013456-bib-0287] Tannock IF. False-positive results in clinical trials: multiple significance tests and the problem of unreported comparisons. Journal of the National Cancer Institute 1996;88(3-4):206-7. [DOI: 10.1093/jnci/88.3-4.206] [PMID: 8632495]

[CD013456-bib-0288] Tewkesbury R. Effects of sexual assaults on men: physical, mental and sexual consequences. International Journal of Men’s Health 2007;6(1):22-35. [DOI: 10.3149/jmh.0601.22]

[CD013456-bib-0289] Thatcher RW. Latest developments in live z-score training: Symptom check list, phase reset, and LORETA zscore biofeedback. Journal of Neurotherapy 2013;17(1):69-87.

[CD013456-bib-0290] Tierney JF, Stewart LA, Ghersi D, Burdett S, Sydes MR. Practical methods for incorporating summary time-to-event data into meta-analysis. Trials 2007;8:16. [DOI: 10.1186/1745-6215-8-16] [PMC1920534] [PMID: 17555582] PMC1920534

[CD013456-bib-0291] Trabold N, McMahon J, Alsobrooks S, Whitney S, Mittal M. A systematic review of intimate partner violence interventions: state of the field and implications for practitioners. Trauma, Violence, & Abuse 2018 Jan 1 [Epub ahead of print]. [DOI: 10.1177/1524838018767934] [PMID: 29649966]

[CD013456-bib-0292] Tylee DS, Gray R, Glatt ST, Bourke F. Evaluation of the reconsolidation of traumatic memories protocol for the treatment of PTSD: a randomized, wait-list-controlled trial. Journal of Military, Veteran and Family Health 2017;3(1):21-33.

[CD013456-bib-0293] Department of Veterans Affairs/Department of Defense. VA/DOD clinical practice guideline for the management of posttraumatic stress disorder and acute stress disorder; 2023. www.healthquality.va.gov/guidelines/MH/ptsd/ (accessed 30 August 2023).

[CD013456-bib-0294] Vaux A, Phillips J, Holly L, Thomson B, Williams D, Stewart D. The Social Support Appraisals (SS-A) Scale: studies of reliability and validity. American Journal of Community Psychology 1986;14(2):195-219. [DOI: 10.1007/BF00911821]

[CD013456-bib-0295] Veronen LJ, Kilpatrick DG. Stress management for rape victims. In: Meichenbaum D, Jaremko ME, editors(s). Stress Reduction and Prevention. New York (NY): Plenum Press, 1983:341-74.

[CD013456-bib-0296] Vickerman KA, Margolin G. Rape treatment outcome research: empirical findings and state of the literature. Clinical Psychology Review 2009;29(5):431-48. [DOI: 10.1016/j.cpr.2009.04.004] [PMC2773678] [PMID: 19442425] PMC2773678

[CD013456-bib-0297] Wachen JS, Jimenez S, Smith K, Resick PA. Long-term functional outcomes of women receiving cognitive processing therapy and prolonged exposure. Psychological Trauma: Theory, Research, Practice, and Policy 2014;6(S1):S58-S65. [DOI: 10.1037/a0035741]

[CD013456-bib-0298] Walby S, Towers J, Francis B. Is violent crime increasing or decreasing? A new methodology to measure repeat attacks making visible the significance of gender and domestic relations. British Journal of Criminology 2016;56(6):1203-34. [DOI: 10.1093/bjc/azv131]

[CD013456-bib-0299] Walker J, Archer J, Davies M. Effects of rape on men: a descriptive analysis. Archives of Sexual Behavior 2005;34(1):69-80. [DOI: 10.1007/s10508-005-1001-0] [PMID: 15772770]

[CD013456-bib-0300] Weare S. “I feel permanently traumatized by it”: physical and emotional impacts reported by men forced to penetrate women in the United Kingdom. Journal of Interpersonal Violence 2021;36(13-14):6621-46. [DOI: 10.1177/0886260518820815] [PMID: 30596303]

[CD013456-bib-0301] Weiss DS, Marmar CR. The Impact of Event Scale - Revised. In: Wilson JP, Keane TM, editors(s). Assessing Psychological Trauma and PTSD. New York (NY): Guilford Press, 1997:399-411. [pp. 399–411]

[CD013456-bib-0302] World Health Organization. Global and Regional Estimates of Violence Against Women: Prevalence and Health Effects of Intimate Partner Violence and Non-Partner Sexual Violence. Geneva, Switzerland: World Health Organization, 2013.

[CD013456-bib-0303] World Health Organization. Responding to Intimate Partner Violence and Sexual Violence Against Women: WHO Clinical and Policy Guidelines. Geneva, Switzerland: World Health Organization, 2013. 24354041

[CD013456-bib-0304] World Health Organization. COVID-19 and violence against women: what the health sector/system can do; April 2020. www.who.int/reproductivehealth/publications/vaw-covid-19/en/ Accessed 30 Aug 2023.

[CD013456-bib-0305] World Health Organization. ICD-11: International Classification of Diseases 11th Revision. icd.who.int/en (accessed 11 November 2021).

[CD013456-bib-0306] Wirtz AL, Poteat TC, Malik M, Glass N. Gender-based violence against transgender people in the United States: a call for research and programming. Trauma, Violence, & Abuse 2018 Feb 13 [Epub ahead of print]. [DOI: 10.1177/1524838018757749] [29439615] 29439615

[CD013456-bib-0307] Wolpe J. Psychotherapy by Reciprocal Inhibition. Redwood City: Stanford University Press, 1958.

[CD013456-bib-0308] Wright ME, Wright BA. Clinical Practice of Hypnotherapy. New York City: Guildford Press, 1987.

[CD013456-bib-0309] Zigmond AS, Snaith RP. The Hospital Anxiety and Depression Scale. Acta Psychiatrica Scandinavica 1983;67(6):361-70. [PMID: 6880820] 10.1111/j.1600-0447.1983.tb09716.x

[CD013456-bib-0310] Zinzow HM, Resnick HS, McCauley JL, Amstadter AB, Ruggiero KJ, Kilpatrick DG. Prevalence and risk of psychiatric disorders as a function of variant rape histories: results from a national survey of women. Social Psychiatry and Psychiatric Epidemiology 2012;47(6):893-902. [DOI: 10.1007/s00127-011-0397-1] [PMCID: PMC4096823] [PMID: 21603967] PMC4096823

[CD013456-bib-0311] Brown SJ, Khasteganan N, Brown K, Hegarty K, Carter GJ, Tarzia L, et al. Psychosocial interventions for survivors of rape and sexual assault experienced during adulthood. Cochrane Database of Systematic Reviews 2019, Issue 11. Art. No: CD013456. [DOI: 10.1002/14651858.CD013456] [PMCID: PMC6836856] PMC10552071 37795783

